# p190A RhoGAP induces *CDH1* expression and cooperates with E-cadherin to activate LATS kinases and suppress tumor cell growth

**DOI:** 10.1038/s41388-020-1385-2

**Published:** 2020-07-08

**Authors:** Hanyue Ouyang, Phi Luong, Morten Frödin, Steen H. Hansen

**Affiliations:** 1 GI Cell Biology Laboratory, Boston Children’s Hospital and Harvard Medical School, Boston, MA 02115 USA; 2 Biotech Research & Innovation Centre (BRIC), University of Copenhagen, 2200 Copenhagen N, Denmark

**Keywords:** Non-small-cell lung cancer, RHO signalling

## Abstract

The *ARHGAP35* gene encoding p190A RhoGAP (p190A) is significantly altered by both mutation and allelic deletion in human cancer, but the functional implications of such alterations are not known. Here, we demonstrate for the first time that p190A is a tumor suppressor using a xenograft mouse model with carcinoma cells harboring defined *ARHGAP35* alterations. In vitro, restoration of p190A expression in carcinoma cells promotes contact inhibition of proliferation (CIP) through activation of LATS kinases and phosphorylation of the proto-oncogenic transcriptional co-activator YAP. In contrast, p190A forms harboring recurrent cancer mutations exhibit loss of function in modulating the Hippo pathway, inducing CIP, as well as attenuated suppression of tumor growth in mice. We determine that p190A promotes mesenchymal to epithelial transition (MET) and elicits expression of a cassette of epithelial adherens junction-associated genes in a cell density-dependent manner. This cassette includes *CDH1* encoding E-cadherin, which amplifies p190A-mediated LATS activation and is necessary for CIP. Oppositely, we establish that p190A is obligatory for E-cadherin to activate LATS kinases and induce CIP. Collectively, this work defines a novel mechanism by which p190A and E-cadherin cooperate in modulating Hippo signaling to suppress tumor cell growth.

## Introduction

Over the past decade large-scale pan-cancer genome sequencing efforts have provided a detailed mutational landscape of human cancer and identified a group of 30–40 of highly significantly mutated genes [1, 2]. With the exception of *ARHGAP35*, all genes in this group were already strongly linked to cancer. The mutational spectrum for *ARHGAP35* is suggestive of a tumor suppressor function [1, 2]. Moreover, the *ARHGAP35* gene is located at Chr. 19q13.32, a region that is frequently deleted in human cancer [3]. Another intriguing discovery from cancer genome analyses is that in lung adenocarcinoma, *ARHGAP35* alterations are found in tumor samples that lack mutations in genes encoding receptor tyrosine kinases (RTKs) or constituents of the RAS–ERK pathway [4]. However, the significance of such findings is difficult to assess, because the role of *ARHGAP35* in cancer is poorly defined.

*ARHGAP35* encodes p190A RhoGAP (p190A), a large GTPase activating protein with functions implicated in cell adhesion, cell migration, cytokinesis, ciliogenesis, entosis, gene transcription, and protein translation [5–12]. Accordingly, *ARHGAP35* is an essential gene, and p190A indeed exerts pivotal functions in development [13, 14]. At the molecular level, the most well-established function of p190A is to promote GTP hydrolysis on Rho GTPases [15]. In addition, motifs in p190A confer scaffolding activities through interactions with p120 RasGAP, TFII-I transcription factors, Rnd proteins, p120-catenin, EIF3 elongation initiation factors, and others [6, 11, 12, 16–20]. Prevailing evidence suggest that both catalytic and scaffolding functions are subject to posttranslational control with protein kinases playing important roles [16, 21–26]. Of note, as suggested by its name, p190A has an ortholog, p190B RhoGAP (p190B) that is encoded by *ARHGAP5* [27]. p190A and p190B exhibit 50% overall sequence identity and highly similar structures. However, *ARHGAP5* is not significantly mutated in human cancer (www.tumorportal.org).

Rho signaling is heavily implicated in control of cell motility [28]. Hence, there is good rationale for suspecting a role for p190A in invasion and metastasis. However, opposing conclusions have been reached in studies examining how p190A modulates motile capacities of oncogenically transformed cells [29–32]. It is well-established that p190A is essential for cell polarity and directional migration [11, 33], but it remains to be shown if perturbation of these functions confers advantages in the context of cancer. Other studies have linked p190A to oncogenic cell transformation and tumor cell growth [31, 34–38]. Generally, this work has centered on the enzymatic activity of p190A and can be difficult to assess due to confounding methodology. This is because expression of either GAP domain alone, dominant active/interfering mutants of Rho GTPases, and/or Rho protein modulating toxins all constitute interventions that exert global effects on Rho signaling, as opposed to specific perturbations of p190A function.

Recently, as result of unbiased transcriptome-based analyses, we established that p190A modulates Hippo signaling to repress expression of a cassette of genes controlled by the proto-oncogenic transcriptional co-activator YAP [39]. We determined that this capacity of p190A is required for contact inhibition of cell proliferation (CIP) in immortalized but non-transformed epithelial cells [39]. In the present work, we have directly tested the significance of *ARHGAP35* alteration in human cancer cells. We have identified NSCLC cell lines with very low expression of p190A, including tumorigenic NCI-H661 (H661) cells with a defined K179* mutation in *ARHGAP35* and loss of heterozygosity (LOH). Using H661 cells in a xenograft nude mouse model, we provide the first direct evidence of p190A acting as a tumor suppressor. It is well established that high cell density activates the canonical Hippo pathway via adherens junctions (AJs) [40, 41]. Here, we determine that wild-type p190A, but not forms harboring recurrent cancer mutations, induces expression of a cassette of genes associated with AJs. This cassette includes *CDH1*, itself a major tumor suppressor gene that encodes E-cadherin, an established modulator of the Hippo pathway [41]. We demonstrate that p190A activates LATS kinases and promotes CIP through induced expression of E-cadherin at high cell density. In turn, p190A is necessary for E-cadherin to activate LATS kinases and promote CIP. Collectively, our results identify a novel mechanism by which p190A and E-cadherin cooperate in a cell density-dependent manner to promote LATS activation and induce CIP.

## Results

### Expression of p190A in carcinoma cells with defined *ARHGAP35* alterations restores Hippo signaling and CIP

**Fig. 1:**
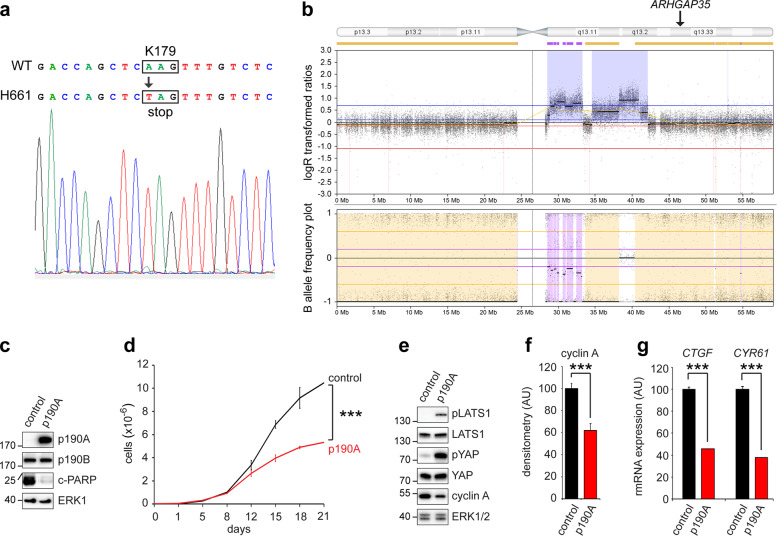
p190A promotes CIP and activates the Hippo pathway in H661 cells with loss-of-function *ARHGAP35* mutation combined with LOH. **a** Sanger sequencing of the *ARHGAP35* exons in H661 cells reveals a A->T transversion at position 535 in exon 1 leading to a K179* mutation. **b** CNA analysis by CytoScan HD assay reveals LOH for most of Chr 19, including the region harboring the *ARHGAP35* gene as indicated. In the top panel showing logR transformed ratios, red lines indicate thresholds for mono- and biallelic deletions, while blue lines mark thresholds for gains and amplifications. Yellow line represents the median of probe values, while solid black lines present estimates of copy number. In the bottom plot showing B allele frequency plot, yellow lines mark thresholds for loss of heterozygosity, while purple lines represent thresholds for allelic imbalance. **c** Western blot of whole cell lysates from control and H661-p190A cells to detect p190A, p190B, cleaved PARP, and ERK1. **d** Growth curves for control and H661-p190A cells. 5 × 10^4^ cells were seeded per well of a 6-well plate and propagated for the number of days indicated with change of medium every 2 days. Cell number was quantified manually. Data are presented as mean ± SD (*n* = 3); *, Student's *t* test, ***p < 0.01. **e** Western blot of whole cell lysates to detect pLATS, LATS, pYAP, and YAP, as well as cyclin A. **f** Quantification by densitometry of cyclin A protein levels detected by western blotting of whole cell lysates prepared from confluent cultures of control and H661-p190A cells. Data are presented as mean ± SD (*n* ≥ 3); pairwise Student's *t* test with p190A(wt), ***p < 0.01. **g** Transcript levels for the bona fide YAP target genes *CTGF* and *CYR61*, as determined by qPCR. Data are presented as mean ± SD (*n* = 3); Student's *t* test, *** *p* < 0.01.

In this work, we aimed to test directly a role for p190A to suppress oncogenic capacities in human cancer cells. *ARHGAP35* mutations are found in ~4% of NSCLC, mainly tumors characterized by the absence of oncogenic driver mutations in the RTK–RAS–ERK pathway [4]. We therefore mined the Cancer Cell Line Encyclopedia (CCLE) [42], to identify cell lines with very low expression of p190A and defined loss-of-function alteration in *ARHGAP35*. We noted a that the *ARHGAP35* gene in H661 cells harbors an A->T transversion resulting in a K179* mutation. We verified the presence of this mutation by Sanger sequencing of genomic DNA from H661 cells (Fig. 1a). Furthermore, we confirmed that H661 cells exhibit LOH for the *ARHGAP35* gene (Fig. 1b).

Next, we examined cellular levels of p190A in pooled populations of H661 cells transduced with empty control vector (control H661 cells) or with p190A expression vector (H661-p190A cells). Western blotting of whole cell lysates failed to reveal any endogenous p190A protein in control H661 cells, while full-length p190A protein was readily detectable in H661-p190A cells (Fig. 1c). Of note, cellular levels of p190B were unaffected by expression of p190A (Fig. 1c). We then tested the effects of p190A expression on cell proliferation. At low cell density, there was no discernable difference in growth rates between H661-p190A and control cells (Fig. 1d). However, while control cells continued to proliferate at high cell density, H661-p190A cells underwent CIP, as evidenced by significant reductions in growth rate and cyclin A levels (Fig. 1d–f). Expression of p190A did not appear to impact cell viability or result in increased apoptosis, which might have influenced differences in saturation densities between H661-p190A and control cells. In contrast, as determined by PARP-cleavage, apoptosis was readily detectable in control H661 cells grown at high cell density, but not in H661-p190A cells (Fig. 1c). Consistent with a pivotal role of Hippo signaling in controlling CIP [40], expression of p190A potently activated LATS kinases and strongly promoted phosphorylation of the Hippo-transducer YAP (Fig. 1e). Moreover, expression of p190A significantly reduced expression levels of the bona fide YAP-target genes *CTGF* and *CYR61* (Fig. 1g). These data are consistent with p190A activating the Hippo pathway and promoting CIP in H661 cells.

### p190A suppresses tumorigenesis in a xenograft nude mouse model

**Fig. 2:**
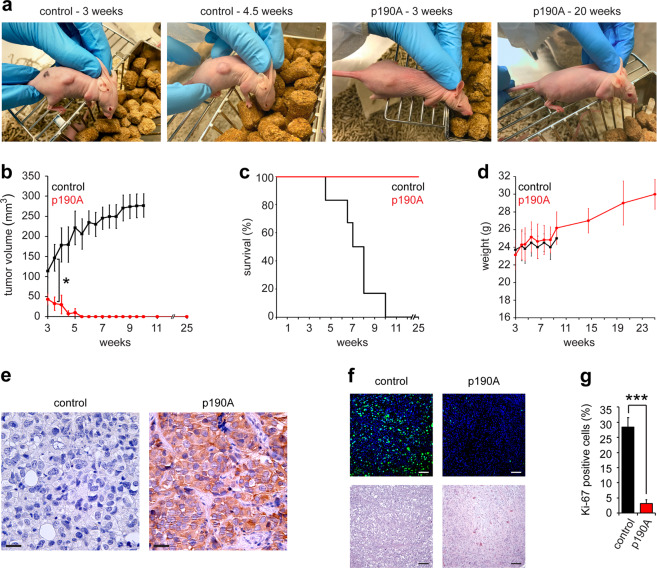
p190A suppresses tumorigenesis in a xenograft mouse model. **a** Images show mice injected with control or H661-p190A cells after 3 weeks and the same mice after 4.5 weeks and 20 weeks, respectively. **b** Cumulative xenograft tumor volume in mice injected with control or H661-p190A cells. Data are presented as mean ± SD (*n* = 6 mice in each group); Student's *t* test, *p < 0.05. **c** Kaplan–Meier survival plot for mice injected with control or H661-p190A cells; log-rank test, **p < 0.025. Mice were sacrificed when tumors measured ≥8-mm along the longest axis. **d** Weight of mice injected with control or H661-p190A cells. Data are presented as mean ± SD. **e** Immunohistochemistry to detect p190A in tumors from mice injected with control or H661-p190A cells. Scale bar 20 µm. **f** Top panels show Ki-67 staining (green) of tumors from mice injected with control and H661-p190A cells, respectively. Nuclei are labeled with Hoechst 33342 (blue). Bottom panels show corresponding adjacent H&E stained sections. Scale bar 100 µm. **g** Quantification of Ki-67 positive nuclei from **f**. Data are presented as mean ± SD; Student's *t* test, ***p < 0.01.

To date, no formal evidence exists of a role *ARHGAP35* as suppressor of tumorigenesis. H661 cells are tumorigenic in nude mice [43]. To test directly if p190A can inhibit tumorigenesis, we implanted 5 × 10^6^ H661-p190A or control cells in the right flank of athymic mice. While control cell tumors grew rapidly, H661-p190A cell tumors were significantly smaller and regressed below detectable size within 5 weeks (Fig. 2a, b). Primary control H661 cell tumors reached maximum size within 4.5–10 weeks (Fig. 2c and Supplementary Fig. S1a, b). Two mice injected with control H661 cells harbored large abdominal metastases (Supplementary Fig. S1c). In contrast, no mice implanted with H661-p190A cell tumors showed any signs of tumors or other disease past 5 weeks and were sacrificed 25 weeks after injection (Fig. 2c). At this time, careful systemic examination failed to reveal any evidence of tumor tissue (Supplementary Fig. S1d). Moreover, mice inoculated with H661-p190A cells continued to gain weight during this time (Fig. 2d).

Histology of tumors from mice implanted with control H661 cells showed a morphology consistent with large cell lung carcinoma (Supplementary Fig. S1e), i.e., the cell type from which H661 cells are derived. To verify the presence of p190A protein in H661-p190A cell tumors, we set up a second cohort where all mice were sacrificed after 3 weeks. Immunohistochemistry revealed strong p190A staining in small tumors in mice inoculated with H661-p190A cells (Fig. 2e). Such staining was completely absent from control H661 cell tumors (Fig. 2e). Ki-67 staining on this set tumors revealed that almost 30% of control H661 cells were Ki-67 positive (Fig. 2f, g). In contrast, only 3% of H661-p190A cells showed staining for Ki-67, consistent with a largely quiescent tumor cell population (Fig. 2f, g). In these samples, we furthermore performed labeling for cleaved caspase-3 in order obtain an estimate of apoptotic cell death (Supplementary Fig. S1f). There was a 60% reduction in caspase-3 staining in H661-p190A cell tumors relative to controls (Supplementary Fig. S1g). However, the ratio of Ki-67/caspase-3 positive cells is nearly fourfold higher in control than H661-p190A cell tumors, which likely explains why the former grow and the latter regress.

### Recurrent *ARHGAP35* mutations in human cancer perturb p190A function

**Fig. 3:**
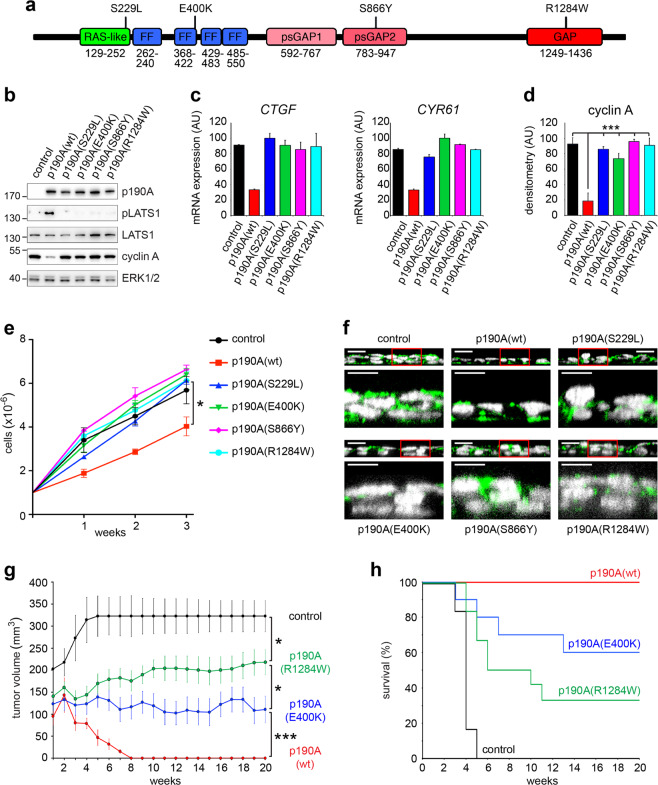
Naturally recurring *ARHGAP35* mutations in human cancer perturb the capacity of p190A to activate the Hippo pathway, promote CIP, and suppress tumorigenesis. **a** Cartoon depicting the p190A(S229L), p190A(E400K), p190A(S866Y), and p190A(R1284W) mutations located within the Ras-like domain, FF motifs, pseudo-GAP and catalytic GAP domains, respectively. **b** Western blotting of whole cell lysates to detect p190A protein, as well as LATS activation and cyclin A in confluent cultures of H661 cells transduced with expression vectors encoding wild type and mutant p190A forms. **c** Transcript levels, as determined by qPCR, for the Hippo signature genes CTGF and CYR61 in H661 cells expressing wild type and mutant p190A forms. **d** Quantification by densitometry of cyclin A protein levels detected by western blotting of whole cell lysates prepared from confluent cultures of H661 cells expressing wild type or mutant p190A forms. Data are presented as mean ± SD (*n* ≥ 3); pairwise Student's *t* test with p190A(wt), ***p < 0.01. **e** Cell number of control H661 cells, as well as H661 cells expressing wild-type or mutant p190A forms 1–3 weeks after plating 1 × 10^6^ cells per well of 6-well dish (1.25 × 10^5^ cells/cm^2^). Data are presented as mean ± SD (*n* = 3); pairwise Student's *t* test with p190A(wt), *p < 0.05. **f** Confocal microscopy of H661 cells with or without expression of wild type or mutant forms of p190A. Cells were stained with fluorescent phalloidin and DRAQ5 to detect polymerized actin (green) and nuclei (white), respectively. Scale bars are 10 µm in length. **g** Cumulative xenograft tumor volume in mice injected with control cells, H661-p190A cells or H661 cells expressing the recurrent p190A mutations E400K or R1284W. Data are presented as mean ± SD; Student's *t* test, *p < 0.05, ***p < 0.01. **h** Kaplan–Meier survival plot for mice injected with control cells, H661-p190A cells, or H661 cells expressing p190A(E400K) or p190A(R1284W); log-rank test results are shown in Supplementary Fig. S2f. Mice were sacrificed when tumors measured ≥8-mm along the longest axis.

The spectrum of *ARHGAP35* mutations in cancer is strongly suggestive of a role as tumor suppressor. Out of 10,953 patients presently in the TCGA database, 422 patients have 458 *ARHGAP35* mutations in tumor samples of which 103 mutations are recurrent; defined as three or more tumors with mutation of a given residue, excluding multiple samples from the same individual (cbioportal.org). To test the significance of individual *ARHGAP35* alterations in cancer, we selected three recurrent mutations: S229L in the Ras-like domain; E400K in the second FF motif; S866Y in the second pseudoGAP domain; as well as R1284W, which targets the arginine-finger of the catalytically active GAP domain (Fig. 3a). We generated pooled populations of H661 cells expressing each of these mutants and compared the effects on Hippo signaling and CIP relative to that of wild type p190A. Strikingly, while expressed at equal levels to wild type (Fig. 3b), all four mutant p190A forms failed to activate LATS-kinases and repress transcription of the YAP-target genes *CTGF* and *CYR61* (Fig. 3c). Likewise, they all failed to promote CIP, as evidenced by cyclin A levels (Fig. 3d) and quantification of cell number (Fig. 3e). Remarkably, whereas control H661 cells exhibit extensive multilayering when cultured on permeable supports, H661-p190A cells form a monolayer (Fig. 3f). In contrast, H661 cells expressing each of the four recurrent cancer mutations studied exhibited cell multilayering similar to control H661 cells (Fig. 3f). Quantification of area of cell multilayering in cultures of control cells, as well as cultures of H661 cells expressing wild-type or mutant p190A forms, supports these conclusions (Supplementary Figs. S2a, b).

To explore the role of recurrent *ARHGAP35* mutations in tumor suppression, we injected groups of 10–12 athymic mice with 5 × 10^6^ H661 cells expressing either p190A(E400K), p190(R1284W), p190A(wt), or empty vector control. Again, inoculation of control H661 cells led to aggressively growing tumors, while injection of p190A(wt) expressing H661 cells resulted in significantly smaller tumors that completely regressed (Fig. 3g). Tumors in mice injected with H661 cells expressing p190A(E400K) or p190A(R1284W) showed intermediate responses with significantly higher tumor burdens than mice injected with p190A(wt), but also significantly smaller than mice inoculated with control H661 cells (Fig. 3g). Tumors were significantly larger in mice injected with cells expressing p190(R1284W) relative to p190A(E400K) (Fig. 3g). We verified that p190A(E400K) and p190A(R1284W) forms were present in tumor cells when mice injected with cells expressing the respective mutants were sacrificed (Supplementary Fig. S3a). Moreover, levels of Ki-67 staining were not significantly different in tumors from mice injected with p190A(E400K) or p190A(R1284W) (Supplementary Fig. S3b, c). Neither was there was there a significant difference in survival (Supplementary Fig. S3d), although the data suggest that with greater numbers of mice, the p190A(R1284W) mutant would be associated with higher mortality than the p190A(E400K) mutant (Fig. 3h). Results of log-rank testing for survival support this notion (Supplementary Fig. S3d).

### Wild-type p190A, but not recurrent mutant forms, induces expression of AJ-associated genes

**Fig. 4:**
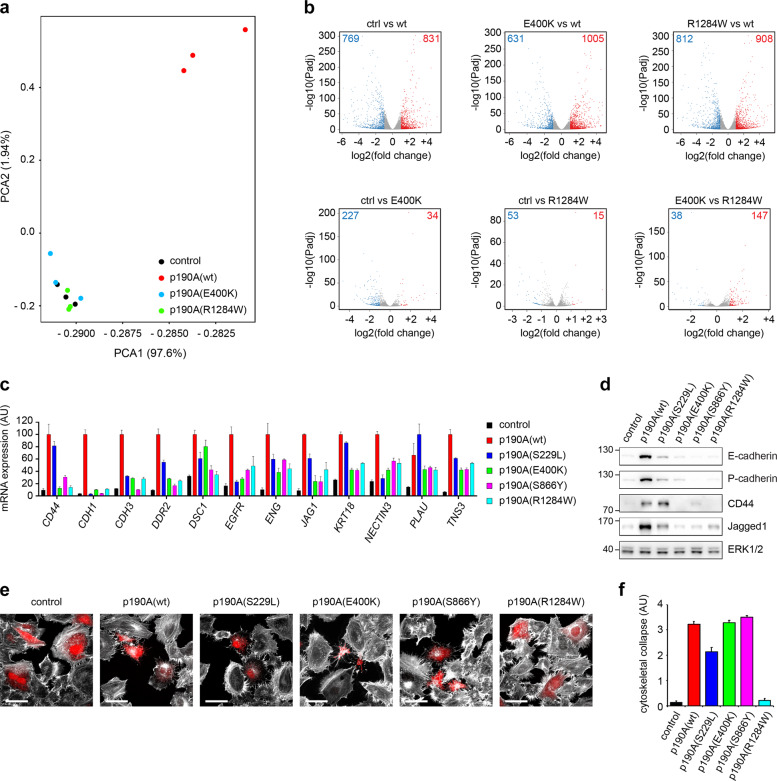
Wild type p190A, but not cancer mutant forms, induces expression of a cassette of AJ-associated genes. **a** Principle component analysis (PCA) for RNA-seq gene expression in control H661 cells, as well as cells expressing p190A(wt), p190A(E400K), or p190A(R1284W). Points represent individual biological replicates for each cell line. **b** Volcano plots of pairwise comparisons of differentially expressed genes in control, p190A(wt), p190A(E400K), and p190A(R1284W) cells. **c** Expression of AJ-associated genes in control H661 cells and H661 cells expressing wild type or mutant forms of p190A, as determined by qPCR analysis. **d** Protein levels of E-cadherin (encoded by *CDH1*), P-cadherin (encoded by *CDH3*), CD44 and Jagged1 (encoded by *JAG1*) in control H661 cells, H661-p190A cells, and cells expressing mutant p190A forms as determined by western blotting of whole cell lysates. **e** Cell morphology of HeLa cells transiently transfected with expression vectors encoding wild type and mutant p190A forms and co-expressing mCherry (red). Cells were stained with fluorescent phalloidin to detect polymerized actin (white). Scale bar 20 µm. **f** Quantification of cytoskeletal collapse in samples shown in **e**.

We next analyzed transcriptomes for control and H661-p190A cells, as well as H661 cells expressing p190A(E400K) and p190A(R1284W). Differentially expressed genes (DEGs) for each comparison are listed in Supplementary Tables S1a–f. A principle component analysis revealed strikingly different gene expression patterns between control H661 and H661-p190A cells (Fig. 4a). In contrast, both p190A(E400K) and p190A(R1284W) mutants formed clusters that overlap with control H661 cells (Fig. 4a). Moreover, as evident from volcano plots, the number of significantly up- or down-regulated genes in H661-p190A cells relative to control cells, as well as p190A(E400K) or p190A(R1284W) expressing H661 cells, were quite similar (Fig. 4b).

Next, we queried DEGs in the Panther gene ontology database [44]. This analysis revealed a significantly induced cassette of genes associated with the AJ in H661-p190A cells relative to control, p190A(E400K) or p190A(R1284W) expressing H661 cells. This cassette comprised the following genes: *CD44*, *CDH1*, *CDH3*, *DDR2*, *DSC1*, *EGFR*, *ENG*, *JAG1*, *KRT18*, *NECTIN3*, *PLAU*, and *TNS3*. Using qPCR analyses, we validated that each of these genes were significantly upregulated in H661-p190A cells relative to control H661 cells, as well as H661 cells expressing the p190A(S229L), p190A(E400K), p190A(S866Y), or p190A(R1284W) recurrent cancer mutations (Fig. 4c). *CD44*, *KRT18*, and *PLAU* transcripts in p190A(S229L) expressing cells represented exceptions (Fig. 4c). We moreover verified that protein levels for E-cadherin and P-cadherin, encoded by *CDH1* and *CDH3*, respectively, as well as CD44 and Jagged1 proteins were elevated in H661-p190A cells relative to control cells and H661 cells expressing the aforementioned p190A cancer mutations with exception of CD44 protein in p190A(S229L) expressing cells (Fig. 4d).

We furthermore tested the effect of recurrent *ARHGAP35* mutations on GAP activity using a validated assay to quantify cytoskeletal collapse [45]. Plasmids encoding wild type or mutant p190A were transfected into HeLa cells that were labeled to detect polymerized actin. In this assay, loss of actin stress fibers and cell rounding serves as a proxy for catalytic activity. As expected, the p190A(R1284W) mutant was devoid of GAP activity, whereas both p190A(E400K) and p190A(S866Y) mutants retained GAP function equivalent to that of wild-type p190A (Fig. 4e, f). The Ras-like domain mutant p190A(S229L) exhibited an intermediate effect (Fig. 4e, f), consistent with a requirement for this domain for catalytic function [46]. Taken together, these data show that there is no simple relationship between GAP activity and tumor suppressor function of p190A forms harboring recurrent cancer mutations with both enzymatic and scaffolding domains exerting pivotal functions.

### Cell density and E-cadherin expression modulate p190A-regulated gene transcription

**Fig. 5:**
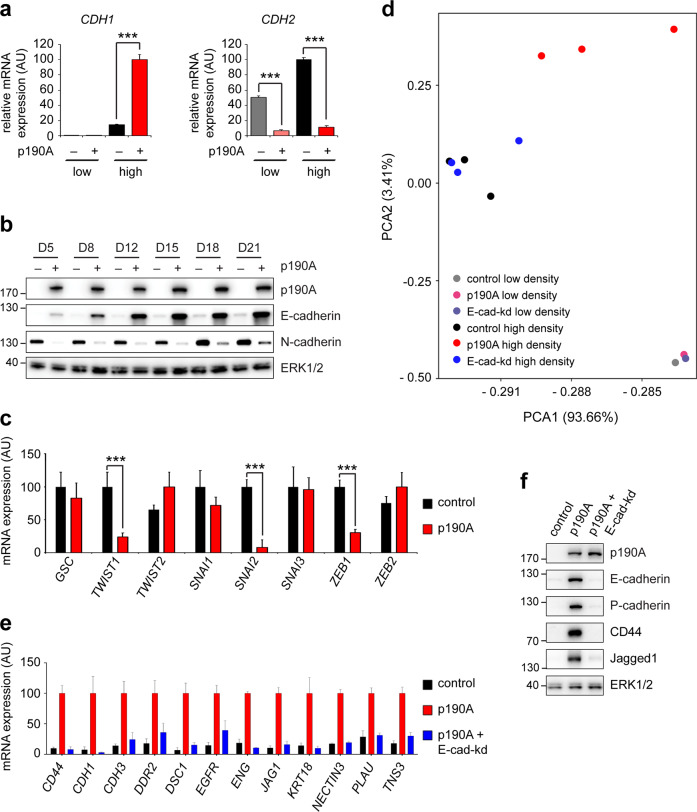
p190A-induced expression of AJ-associated genes is dependent on both E-cadherin and high cell density. **a** *CDH1* (encoding E-cadherin) and *CDH2* (encoding N-cadherin) transcript levels, as determined by qPCR, in control and H661-p190A cells, cultured at low (2 × 10^4^ cells/cm^2^) or high (>2 × 10^5^ cells/cm^2^) density. **b** E-cadherin and N-cadherin protein levels in control and H661-p190A cells various times after seeding at low density (2 × 10^4^ cells/cm^2^) and propagated until reaching saturation density for H661-p190A cells (~5 × 10^5^ cells/cm^2^ on day 21). **c** Expression of EMT-inducing transcription factors in control H661 and H661-p190A cells, as determined by qPCR. **d** Principle component analysis (PCA) for RNA-seq gene expression in control H661 cells and H661-p190A(wt) cells, without or with E-cadherin knockdown, cultured at low (2 × 10^4^ cells/cm^2^) or high (2 × 10^5^ cells/cm^2^) density. Points represent individual biological replicates for each cell line. **e** Expression of AJ-associated genes in control H661 cells and H661-p190A(wt) cells, without or with E-cadherin knockdown, cultured at high density (>2 × 10^5^ cells/cm^2^), as quantified by qPCR analysis. **f** Protein levels of P-cadherin, CD44, and Jagged1, as determined by western blotting of whole cell lysates, in control H661 cells or H661-p190A cells without or with E-cadherin knockdown cultured at high density (>2 × 10^5^ cells/cm^2^).

Among the genes in the AJ cassette defined above, we focused our efforts on *CDH1* encoding E-cadherin, because it is a major cancer gene, its expression is known to activate the Hippo pathway, and because E-cadherin is a key constituent of AJs [41]. E-cadherin levels have been shown to be modulated by cell density in human cancer cells [47]. Moreover, we noted from our transcriptomic data that N-cadherin, encoded by *CDH2*, was regulated in an opposite manner of E-cadherin (Supplementary Tables S1a–f), thus suggesting a that p190A promotes mesenchymal-to-epithelial transition (MET) in H661 cells. To test this possibility directly, we performed qPCR analyses for *CDH1* and *CDH2* transcripts in control H661 and H661-p190A cells cultured at low versus high cell density. *CDH1* transcript levels were significantly higher in H661-p190A cells than in control H661 cells cultured at high cell density (Fig. 5a). In comparison, *CDH1* transcript was virtually undetectable in cells cultured at low density, irrespective of p190A expression (Fig. 5a). In contrast, *CDH2* transcript levels were high in control H661 cells and significantly lower in H661-p190A cells at both low and high cell density (Fig. 5a). To determine if protein and transcript levels correlated, we seeded cells at low density and propagated these until they reached high density. From day 5 to 21, we harvested cells to prepare whole cell lysates for detection of E- and N-cadherin by western blotting. E- and N-cadherin levels were reciprocal between control and H661-p190A cells at all times, but only E-cadherin was more strongly induced by high cell density (Fig. 5b). Moreover, we examined the effect of p190A on expression of the Gsc, Twist, Slug, Snail, and Zeb transcription factors that are established regulators of EMT [48]. Consistent with their roles to promote EMT [49–51], *TWIST1*, *SNAI2*, and *ZEB1* transcripts were significantly downmodulated in confluent H661-p190A cells relative to control H661 cells (Fig. 5c). Taken together these data demonstrate that p190A promotes MET in H661 cells with high cell density serving as a requirement for induced expression of E-cadherin.

To test if E-cadherin modulates gene expression in H661-p190A cells, we conducted transcriptome analyses of control, as well as H661-p190A cells with or without depletion of E-cadherin, cultured at both low and high cell density. To this end, we knocked down E-cadherin in H661-p190A cells using a validated shRNA [52]. Strikingly, E-cadherin markedly contributed to alterations in gene expression, but only at high cell density (Fig. 5d and Supplementary Tables S2a–f). At low cell density, there was virtually no difference in the transcriptomes of control H661 and H661-p190A cells and also no effect of E-cadherin depletion. Thus, p190A controls gene expression in a manner that is entirely dependent on cell density and strongly influenced by E-cadherin levels. To this end, ~50% of p190A-regulated genes were entirely dependent on E-cadherin expression (Supplementary Fig. S4 and Supplementary Tables S2a–f). Notably, this included all genes in the AJ cassette, as determined by qPCR analysis (Fig. 5e). Again, we confirmed that protein and mRNA levels for P-cadherin, CD44, and Jagged1 were regulated in a coordinate fashion (Fig. 5f). In addition, we determined that in NSCLC NCI-H226 (H226) cells, p190A activates the canonical Hippo pathway, induces E-cadherin expression, and promotes CIP when grown at high cell density (Supplementary Fig. S5a–d). This result indicates that our findings are not restricted to H661 cells.

### E-cadherin is necessary but not sufficient for p190A-induced CIP

**Fig. 6:**
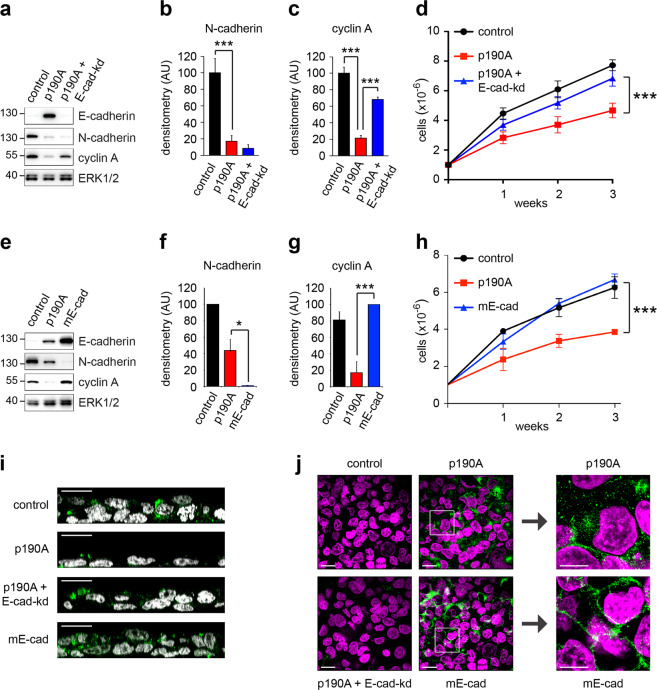
E-cadherin is required, but N-cadherin dispensable, for p190A-induced CIP. **a** Western blotting to detect E- and N-cadherin, as well as cyclin A and ERK1/2 in whole cell lysates from control H661 cells and H661-p190A cells with or without E-cadherin knockdown. **b** Quantification by densitometry of N-cadherin protein levels from experiments as shown in **a**. Data are presented as mean ± SD (*n* = 3). Student's *t* test, ***p < 0.01. **c** Quantification by densitometry of cyclin A protein levels from experiments as shown in **a**. Data are presented as mean ± SD (*n* = 3). Student's *t* test, ***p < 0.01. **d** Number of control H661 cells, as well as H661-p190A cells with or without E-cadherin knockdown 1–3 weeks after plating 1 × 10^6^ cells per well of 6-well dish. **e** Western blotting to detect E- and N-cadherin, as well as cyclin A and ERK1/2 in whole cell lysates from control H661 cells and H661-p190A cells, as well as control H661 cells transduced with mouse E-cadherin expression vector (mE-cad). **f** Quantification by densitometry of N-cadherin protein levels from experiments as shown in **e**. Data are presented as mean ± SD (*n* = 3). Student's *t* test, *** *p* < 0.01. **g** Quantification by densitometry of cyclin A protein levels from experiments as shown in **e**. Data are presented as mean ± SD (*n* = 3). Student's *t* test, *** *p* < 0.01. **h** Cell number of control H661 cells, H661-p190A cells, or H661 cells expressing mE-cad 1–3 weeks after plating 1 × 10^6^ cells per well of six-well dish. **i** Fluorescence microscopy of control, H661-p190A cells without and with E-cadherin knockdown, as well as H661 cells expressing mE-cad labeled with Alexa^488^-phalloidin (green) to visualize polymerized actin. Nuclei were labeled with DRAQ5 (white). **j** Immunofluorescence microscopy to detect E-cadherin (green) in control, H661-p190A cells without and with E-cadherin knockdown, as well as mE-cad cells. Nuclei were stained with DRAQ5 (purple). Scale bar 20 µm. Insets magnified from p190A and mE-cad panels as indicated. Scale bar 10 µm.

Subsequently, we tested the functional significance of E-cadherin to p190A-regulated CIP and MET. Depletion of E-cadherin from H661-p190A cells failed to restore N-cadherin levels (Fig. 6a, b). However, knockdown of E-cadherin in H661-p190A cells perturbed CIP, as demonstrated by significantly elevated cyclin A levels and enhanced cell proliferation (Fig. 6c, d). Next, we transduced H661 cells with a mouse E-cadherin expression construct to test if E-cadherin in the absence of p190A would be sufficient to induce CIP (Fig. 6e). While exogenous E-cadherin was sufficient to repress expression of N-cadherin in H661 cells (Fig. 6f), it did not reduce cyclin A levels (Fig. 6g), and cell proliferation was not significantly different from control H661 cells (Fig. 6h). It was moreover apparent that depletion of E-cadherin from H661-p190A cells promotes cell multilayering, and that expression of exogenous E-cadherin in control H661 cells was insufficient to attenuate cell multilayering (Fig. 6i).

We furthermore examined the subcellular localization of E-cadherin in control H661 cells and in H661-p190A cells without or with depletion of E-cadherin, as well as in H661 cells expressing mouse E-cadherin. In H661-p190A cells, we observed E-cadherin staining at areas of cell–cell contact, but also intracellular staining with a pattern suggestive of vesicular localization (Fig. 6j). No such staining was observed in control H661 cells or H661-p190A cells with knockdown of E-cadherin. H661 cells expressing mouse E-cadherin showed stronger staining than H661-p190A cells with E-cadherin predominantly localized to adherens junctions (Fig. 6j, inset). Collectively, these experiments demonstrate that p190A does not promote CIP merely by inducing E-cadherin expression. Instead, co-expression of p190A and E-cadherin is required for CIP. Moreover, that while p190A controls a MET switch, downmodulation of N-cadherin per se is not sufficient to induce CIP.

### p190A and E-cadherin cooperate to promote LATS-dependent CIP

**Fig. 7:**
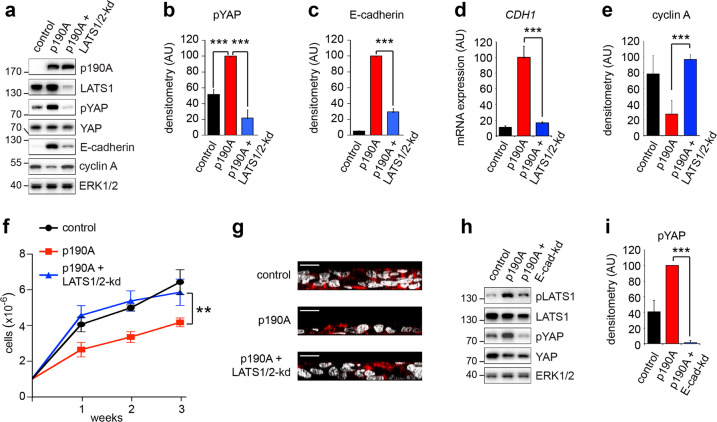
p190A cooperates with induced E-cadherin to activate the Hippo pathway and promote CIP. **a** Western blots of whole cell lysates from control and H661-p190A cells without and with LATS1/2 knockdown to detect LATS1, pYAP(S127), E-cadherin, and cyclin A. **b** Quantification by densitometry of pYAP(S127) levels from experiments as shown in **a**. Data are presented as mean ± SD (*n* = 3). Student's *t* test, ***p < 0.01. **c** Quantification by densitometry of E-cadherin protein levels from experiments as shown in **a**. Data are presented as mean ± SD (*n* = 3). Student's *t* test, ***p < 0.01. **d** Quantification by qPCR of *CDH1* transcript control and H661-p190A cells without and with LATS1/2 knockdown. Data are presented as mean ± SD (*n* = 3). Student's *t* test, ***p < 0.01. **e** Quantification by densitometry of cyclin A protein levels from experiments as shown in **a**. Data are presented as mean ± SD (*n* = 3). Student's *t* test, ***p < 0.01. **f** Cell number of control H661 cells, as well as H661-p190A cells with or without LATS1/2 knockdown 1–3 weeks after plating 1 × 10^6^ cells per well of six-well dish. The data are presented as mean ± SD (*n* = 3). Student's *t* test, **p < 0.025. **g** Confocal microscopy of control and H661-p190A cells with or without LATS1/2 knockdown labeled with fluorescent phalloidin (red) and DRAQ5 (white) to detect polymerized actin and nuclei, respectively. **h** Western blotting of whole cell lysates from control and H661-p190A cells with or without E-cadherin knockdown to detect pLATS1(S909) and pYAP(S127). **i** Quantification by densitometry of pYAP(S127) levels from experiments as shown in **h**. The data are presented as mean ± SD (*n* = 3). Student's *t* test, ***p < 0.01.

To order p190A and E-cadherin relative to the Hippo pathway, we generated H661-p190A cells with concomitant depletion of LATS1 and LATS2 using a validated dual shRNA construct [53]. In H661-p190A cells with LATS1/2-kd, the phospho-YAP level was significantly reduced relative to H661-p190A cells transduced with empty vector (Fig. 7a, b). Depletion of LATS1/2 from control H661 cells did not substantially impact E-cadherin or cyclin A levels (Supplementary Fig. S6a). These results clearly establish that p190A activates canonical Hippo signaling, for which LATS-dependency represents the operational definition [54, 55]. Of note, inhibition of Rho signaling in control H661 cells by incubation with Clostridium botulinum C3 toxin or treatment with the ROCK inhibitor Y-27632 was not sufficient to activate the canonical Hippo pathway or induce E-cadherin expression (Supplementary Fig. 6b, c). Thus, the activation of LATS kinases by p190A cannot be explained merely by enzymatic activity toward Rho GTPases but must require additional functions, as for instance compartmentalization of Rho signaling.

Both E-cadherin protein and *CDH1* transcript levels were diminished upon LATS1/2-kd in comparison with H661-p190A cells without depletion of LATS1/2 (Fig. 7c, d). In addition, cyclin A levels and cell proliferation were significantly higher in H661-p190A cells with LATS1/2-kd than without (Fig. 7e, f). Accordingly, H661-p190A cells with LATS1/2-kd exhibited cell multilayering similar to control H661 cells (Fig. 7g and Supplementary Fig. S6d). Next, we examined the effect of E-cadherin depletion from H661-p190A cells on the Hippo pathway. We found that knockdown of E-cadherin attenuated activation of LATS kinases and potently inhibited phosphorylation of YAP in H661-p190A cells (Fig. 7h, i). We furthermore performed calcium switch assays, which revealed that LATS activation and induction of E-cadherin expression in H661-p190A cells coincide (Supplementary Fig. S6e).

**Fig. 8:**
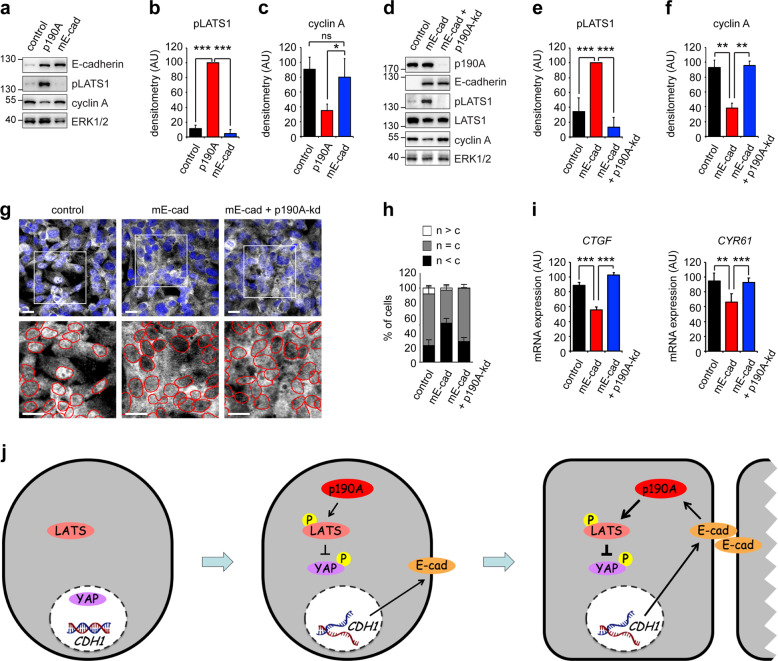
p190A is necessary for E-cadherin to activate the Hippo pathway. **a** Western blots of whole cell lysates from control H661 cells with or without mouse E-cadherin, as well as from H661-p190A cells to detect E-cadherin, pLATS1 and cyclin A. **b** Quantification by densitometry of pLATS levels from experiments as shown in **a**. Data are presented as mean ± SD (*n* = 3). Student's *t* test, ***p < 0.01. **c** Quantification by densitometry of cyclin A protein levels from experiments as shown in **a**. Data are presented as mean ± SD (*n* = 3). Student's *t* test, *p < 0.05, ns: not significant. **d** Western blots of whole cell lysates from MDA-MB-231 cells with or without mouse E-cadherin and with or without p190A knockdown to detect p190A, E-cadherin, pLATS1, LATS and cyclin A. **e** Quantification by densitometry of pLATS levels from experiments as shown in **d**. Data are presented as mean ± SD (*n* = 3). Student's *t* test, ***p < 0.01. **f** Quantification by densitometry of cyclin A protein levels from experiments as shown in **d**. Data are presented as mean ± SD (*n* = 3). Student's *t* test, **p < 0.025. **g** Top panels show confocal microscopy of MDA-MB-231 cells with or without mouse E-cadherin and with or without p190A knockdown. Cells were immunolabeled to detect YAP (white) and labeled with DRAQ5 to detect nuclei (blue), respectively. Scale bars represent 20 µm. Bottom panels show magnifications of the boxed areas in top panels. Red lines outline perimeters from nuclei manually traced from the DRAQ5 labeling. Scale bars represent 20 µm. **h** Quantification of YAP staining in nuclei relative to cytoplasm of cells as exemplified in **g**; n nucleus, c cytoplasm. Intensity of YAP staining was determined to be predominantly nuclear (white), cytoplasmic (black), or equally nuclear and cytoplasmic (gray). **i** Transcript levels for the YAP target genes *CTGF* and *CYR61*, as determined by qPCR. Data are presented as mean ± SD (*n* = 3); Student's *t* test, **p < 0.025, ***p < 0.01. **j** Model illustrating a proposed feed forward cycle by which p190A by activation of the Hippo pathway promotes *CDH1* transcription. In turn, induced expression of E-cadherin at high cell density enhances p190A-mediated LATS activation to promote CIP.

Finally, we tested if p190A is necessary for the capacity of exogenous E-cadherin to activate the Hippo pathway [41]. In the absence of p190A, exogenous expression of E-cadherin was not sufficient to activate LATS1 or significantly impact cyclin A levels in H661 cells (Fig. 8a–c). In contrast, expression of E-cadherin in MDA-MB-231 cells with readily detectable levels of endogenous p190A strongly activated LATS1 and downmodulated cyclin A levels (Fig. 8d–f). Strikingly, using a validated shRNA construct [39], knockdown of p190A in MDA-MB-231 cells abrogated activation of LATS1 kinase (Fig. 8d–f). Furthermore, depletion of p190A from MDA-MB-231 cells reduced nuclear translocation of YAP (Fig. 8g, h), as well as attenuated transcription of the YAP-target genes *CTGF* and *CYR61* (Fig. 8i). Taken together, these results demonstrate that p190A establishes a feed-forward cycle in which transcription of the *CDH1* gene is LATS- and cell density-dependent. In turn, induced E-cadherin cooperates with p190A to activate LATS1 and promote CIP. This novel tumor suppressor mechanism for *ARHGAP35* is consistent with the model proposed in Fig. 8j.

## Discussion

Among highly significantly mutated genes in human cancer, *ARHGAP35* encoding p190A is unusual in that its role in cancer is poorly understood. The mutation spectrum and copy number alterations are suggestive of a tumor suppressor function, but it has been difficult to verify due to lack of co-mutation data and relevant model systems [1–3]. In previous work, we established that p190A and its ortholog p190B activate LATS kinases and repress the Hippo transducer YAP to promote CIP in non-transformed epithelial cells [39]. However, the relevance of these findings to the tumor suppressor function of p190A in human cancer was not clear. First, loss of CIP upon depleting p190A from non-oncogenically transformed cells is evidently quite different from restoring CIP in human cancer cells with numerous mutations of which the vast majority are of unknown significance. Second, in our previous study, we were unable to separate the function of p190A from that of p190B encoded by the *ARHGAP5* gene, which is not significantly mutated in human cancer. Finally, no insight was gained into the mechanism by which p190A activated LATS kinases to promote CIP [39].

Here, we aimed to directly test a role for p190A in cancer. To this end, we were aided by a recent study from others demonstrating that *ARHGAP35* mutations are found in a subset of lung adenocarcinomas lacking oncogenic driver mutations in the RTK–RAS–ERK pathway [4]. With this information at hand, we surveyed the CCLE database for cell lines matching these criteria. While such cell lines are rare, we noted that the H661 cell line exhibits very low levels of p190A transcript and confirmed the presence of a K179* mutation combined with LOH. Next, we determined that reconstitution of p190A expression is sufficient to activate the Hippo pathway and restore CIP in vitro, as well as repress tumorigenesis in athymic mice, thus providing conclusive evidence of a tumor suppressor function for *ARHGAP35*. It is established that YAP can functionally substitute for oncogenic RAS in cancer [56, 57]. Based on our current findings, one can hypothesize that in a subset of cancers, deregulated Hippo signaling due to loss of p190A function is sufficient to bypass CIP in the absence of oncogenic driver mutations in the RTK–RAS–RK pathway. Consistent with this hypothesis, mutations in *LATS1* were also significantly enriched among lung adenocarcinomas lacking oncogenic driver mutations in the RTK–RAS–ERK pathway [4].

YAP and TAZ serve established functions to promote EMT [58–60]. As revealed by a comprehensive transcriptome analyses and validated by qPCR and western blotting, we demonstrate here that p190A modulates transcription of a cassette of AJ-associated genes. This includes E-cadherin, which is required but not sufficient for inducing expression of other members of the AJ cassette. Expression of p190A moreover promotes an N-cadherin to E-cadherin switch associated with MET. However, p190A-induced E-cadherin expression and down-modulation of N-cadherin are uncoupled, as knockdown of E-cadherin in H661-p190A cells fails to restore N-cadherin expression. At higher protein levels of E-cadherin, such as those obtained by expression of exogenous E-cadherin, N-cadherin expression is indeed repressed, but such cells fail to activate LATS kinases and undergo CIP. Thus, these results reveal that p190A is not only required to induce transcription of *CDH1* at high cell density. Expression of p190A is also obligatory for E-cadherin-mediated activation of LATS kinases and CIP. Thus, both p190A and E-cadherin are necessary, but neither sufficient to efficiently activate the canonical Hippo pathway at high cell density and induce CIP. Evidently, more work is necessary to identify the precise mechanism by which p190A and E-cadherin cooperate, but it seems probable that key constituents in this pathway operate in the context of cell–cell interaction.

Finally, we establish here that naturally occurring recurrent cancer mutations in *ARHGAP35* profoundly impact Hippo signaling, CIP and tumor suppressor capacity. Intriguingly, these mutants include p190A(R1284W) with no enzymatic activity, as well as p190A(E400K) and p190A(S866Y) with seemingly intact GAP function. Hence, there is no simple relationship between enzymatic activity and cell signaling. One explanation to account for these findings is that scaffolding function is required for proper subcellular targeting of the GAP domain. An alternative possibility is that hydrolysis of GTP-bound Rho protein bound to the GAP domain elicits a change in confirmation to modulate scaffolding functions of p190A. Either of these scenarios are consistent with our present findings. Moreover, host factors are also likely to play a role, as the effect of recurrent cancer mutations in p190A showed greater penetrance in vitro vs in vivo. Testing of additional cancer mutations, recurrent as well as unique, will help to identify regions of p190A that are important for tumor suppressor capacity. Such efforts will also aid in interpreting the significance of individual *ARHGAP35* mutations in tumor samples. Our work demonstrates that p190A exerts tumor suppressor function, at least in part through effects on the Hippo pathway. YAP/TAZ signaling, primarily the interaction with TEAD family transcription factors [61], is actively being pursued as a drug target [62–64]. Thus, present and future investigations into the tumor suppressor function of p190A may directly impact future personalized targeted therapy for malignancies with *ARHGAP35* alteration.

## Materials and methods

### Reagents

The following reagents were used for this work: Blasticidin (EMD Millipore): Calyculin A (Cell Signaling Technologies); Hoechst 33342 (Life Technologies); DMEM (Corning); DMEM/F12 1:1 (Gibco); DRAQ5 (Biostatus); fetal bovine serum (FBS; Atlanta Biologicals); Fast SYBR green master mix reagent (Applied Biosystems); FluorSave™ (EMD Millipore); Hoechst 33342 (Invitrogen); goat serum (Gibco); Omnifect (Transomic technologies); PageRuler™ prestained protein ladder 10–180 kD (Thermo Fisher); phalloidin/Alexa^488^ and phalloidin/Alexa^594^ (Invitrogen); Polybrene (Santa Cruz Biotechnology); Puromycin (Sigma Aldrich); Trypsin-EDTA, 0.25% (Gibco).

### Antibodies

Antibodies used for this study were as follows:

**Table:** 

Antigen	Species	Company	Cat. no.
Caspase-3 (N17)	rabbit monoclonal	Cell Signaling Technologies	9661S
CD44	rabbit monoclonal	Cell Signaling Technologies	37259S
Cyclin A	rabbit polyclonal	Santa Cruz Biotechnologies	sc-751
E-Cadherin	mouse monoclonal	BD Biosciences	610182
E-Cadherin	rabbit polyclonal	Cell Signaling Technologies	3195S
ERK1	mouse monoclonal	Santa Cruz Biotechnologies	sc-271269
ERK1/2	rabbit polyclonal	Sigma Aldrich	M5670
Jagged1	rabbit monoclonal	Cell Signaling Technologies	70109T
Ki-67	rabbit polyclonal	Thermo Fisher Scientific	RM9106S
LATS1	rabbit monoclonal	Cell Signaling Technologies	3477S
pLATS1(S909)	rabbit monoclonal	Cell Signaling Technologies	9157S
N-cadherin	mouse monoclonal	Cell Signaling Technologies	14215S
p190A	mouse monoclonal	BD Biosciences	610150
p190B	mouse monoclonal	BD Biosciences	611613
PARP	mouse monoclonal	BD Biosciences	611039
p-Cadherin	rabbit monoclonal	Cell Signaling Technologies	2189T
YAP1	rabbit polyclonal	Cell Signaling Technologies	4912S
pYAP1(S127)	rabbit polyclonal	Cell Signaling Technologies	4911S

Secondary antibodies obtained from Invitrogen were the following: goat anti-mouse/Alexa^488^; goat anti-mouse/Alexa^555^; goat anti-rabbit/Alexa^488^; goat anti-mouse/HRP; goat anti-rabbit/HRP. Donkey anti-rabbit/Alexa^488^ secondary antibody was purchased from Jackson ImmunoResearch Lab.

### Plasmid constructs

All p190A expression constructs were synthesized with an N-terminal Myc-tag by Gene Oracle, Inc. and cloned into the lentiviral vector pUltra-hot from Addgene, plasmid #24130. The entire cDNAs for wild-type or mutant p190A forms were verified by Sanger sequencing. Validated pLKO.1 puro shRNA E-cadherin lentiviral plasmid was purchased from Addgene, plasmid #18801. Validated pLenti-EmGFP-LATS2/1-kd plasmid to deplete LATS1 and LATS2 together was likewise obtained from Addgene, plasmid #52085. Lentiviral expression construct encoding mouse E-cadherin was similarly purchased from Addgene, plasmid #18804. Lentiviral pZIP vectors encoding shRNAs targeting human p190A were purchased from transOMIC technologies Inc; cat. no. TRHS1000-35 (*ARHGAP35*/p190A) and validated previously [39].

### Cell culture, transfection, transduction, and selection

NCI-H226 and NCI-H661 cells were purchased from ATCC. NCI-H661 cells were propagated in DMEM/F12 1:1 supplemented with 10% FBS. NCI-H226 cells were cultured in RPMI 1640 supplemented with 10% FBS. 293T, HeLa, and MDA-MB-231 cells were from lab stock and grown in DMEM with 10% FCS. All transfections were performed using Omnifect according to the manufacturer’s instructions. For lentiviral transduction, one 100-mm dish with 70% confluent 239T cells was transfected with 2 µg each of VSV-G and PAX2 encoding plasmids, as well as 2-µg transfer vector. The culture medium was replaced after 24 h and the medium containing lentiviral particles harvested after 48 h. Following filtration through a 0.45-µm filter, the medium was supplemented with 6–8 µg/ml polybrene and added to NCI-H226, NCI-H661 or MDA-MB-231 cells. In some instances, transduced cells were enriched either by FACS sorting on a BD FacsMelody machine, or by drug selection with 10 µg/ml blasticidin, or 2 µg/ml puromycin for 10 days. Finally, selected cells were expanded to generate frozen stock.

### Confocal microscopy

Samples were rinsed once with PBS and fixed in 3.7% formalin containing 10% methanol for 10 min at room temperature. After rinsing three times with PBS, samples were incubated 30 min in PBS containing 10% normal goat serum, 0.2% fish skin gelatin, and 0.1% Triton X-100 (PBS-NGS). Samples were then incubated with primary antibodies diluted in PBS-NGS for 1 h, rinsed extensively with PBS containing 0.1% Triton X-100 for 30 min, and then incubated with secondary antibodies diluted in PBS-NGS for 40 min. Following further extensive rinsing for 30 min, samples were stained for 15 min DRAQ5 diluted 1:300 in PBS to detect nuclei and, when relevant, phalloidin/Alexa^488^ or phalloidin/Alexa^594^ to visualize polymerized actin. After final rinsing in PBS, samples were mounted with FluorSave.

### Cytoskeletal collapse assay

For these experiments, 2 × 10^4^ HeLa cells were seeded into each well of six well dishes containing collagen type I-coated coverslips. Cells were transfected with pUltra-Hot expression vectors encoding wild-type or mutant p190A forms or using Omnifect according to the manufacturer’s instructions. Approximately 24 h later, cells were fixed with 3.7% formalin containing 10% methanol for 10 min at room temperature and stained with phalloidin/Alexa^488^ to detect polymerized actin. Samples were evaluated by confocal microscopy. Transfected cells were easily identified, because the pUltra-Hot vector co-expresses mCherry. Experiments were carried out three times independently, and images of minimum of 60 cells were systematically sampled from each condition. Each cell was scored on a 0–2 scale for loss of actin stress fibers and, similarly, on a scale from 0 to 2 for cell rounding thus yielding a composite score ranging from 0 to 4. Finally, an average score and standard deviation for each condition was calculated.

### Xenograft tumorigenesis in nude mice

For these experiments 6–7 weeks old female outbred homozygous nude Foxn1^nu^/Foxn1^nu^ were obtained from The Jackson Laboratory Cat. no. 007850) and acclimatized for several days. Next, mice were injected with 100 µl Matrigel containing 5 × 10^6^ control or H661-p190A cells in the right flank. At this time, mice were furthermore ear-tagged. Mice were then followed for up to 25 weeks after injection, during which weight and period tumor size (length × width) were measured 1–2 times per week and the condition of mice recorded. Upon tumors reaching maximum length of ≥8-mm, mice were euthanized by CO_2_ asphyxiation per institutional guidelines and tumors were removed for histology. Moreover, abdominal and thoracic cavities were accessed by midline incision. and carefully inspected for any signs of metastases. In addition, the skin on the back of mice was removed to assess local invasion and penetrance of the body wall. These procedures were conducted according to BCH IACUC-approved protocol #3319.

### Histology

Tumor samples were fixed in 10% formalin overnight, dehydrated and embedded in Tissue Prep 2 paraffin. Samples were sectioned with a rotary microtome, and H&E staining was performed in an Autostainer. For Ki-67, antigen retrieval was performed by boiling the slides for 10 min in 10 mM sodium citrate buffer in a pressure cooker. The sections were blocked with 5% normal donkey serum (Jackson ImmunoResearch Lab Inc, West Grove PA) for an hour at room temperature. Sections were then incubated with rabbit anti-Ki-67 antibody overnight at 4 °C. Next, sections were washed in TBS/TBST and incubated with Alexa^488^-conjugated donkey anti-rabbit secondary antibody diluted 1:300. Finally, sections were counter-stained with Hoechst 33342, washed with TBS/TBST and mounted in Prolong Gold anti-fade mounting media (Invitrogen). Quantification of Ki-67 and Hoechst 33342 staining was performed on systematically sampled images using Cell Profiler to define and count particles representative of Ki-67 and Hoechst 33342 positive nuclei, respectively. Staining to detect cleaved caspase-3(Asp175) and p190A was performed using similar methods, except that mouse anti-p190A antibody followed by HRP-conjugated goat anti-rabbit/mouse secondary antibody were used for immunolabeling followed by H&E staining.

### Real-time qPCR

For real-time qPCR analyses, total RNA was extracted from cell pellets using Qiagen RNeasy Mini and QIAshredder kits according to the manufacturer’s instructions. cDNA was synthetized using Bio-Rad iScript cDNA Synthesis Kit according to the manufacturer’s instructions. qRT-PCR was performed with the One Step plus Sequence Detection System using Fast SYBR green master mix reagent. Gene expression levels were normalized to the two “housekeeping” genes *HPRT1* and *RPS18*. Primer sequences for relevant genes were as follows:

**Table:** 

Gene	Forward primer (5′-3′)	Reverse primer (5′-3′)
*CD44*	GAGATGCTGTAGCGACCATT	GACACCATGGACAAGTTTTGG
*CDH1*	GTCACTGACACCAACGATAATCCT	TTTCAGTGTGGTGATTACGACGTTA
*CDH2*	CCTCCAGAGTTTACTGCCATGAC	GTAGGATCTCCGCCACTGATTC
*CDH3*	CAGGTGCTGAACATCACGGACA	CTTCAGGGACAAGACCACTGTG
*CTGF*	GAAGCTGACCTGGAAGAGAACA	CGTCGGTACATACTCCACAGAA
*CYR61*	GAGTGGGTCTGTGACGAGGAT	GGTTGTATAGGATGCGAGGCT
*DDR2*	AACGAGAGTGCCACCAATGGCT	ACTCACTGGCTTCAGAGCGGAA
*DSC1*	CAGAGTCAAGATGGCTTCCCAG	GTTCTCAAGTCGCCAGTGTGTTG
*EGFR*	ACCAATACCTATTCCGTTACACA	CCCGTAATTATGTGGTGACAGA
*ENG*	CGGTGGTCAATATCCTGTCGAG	AGGAAGTGTGGGCTGAGGTAGA
*HPRT1*	TTGCTTTCCTTGGTCAGGCA	ATCCAACACTTCGTGGGGTC
*JAG1*	TGCTACAACCGTGCCAGTGACT	TCAGGTGTGTCGTTGGAAGCCA
*KRT18*	GCTGGAAGATGGCGAGGACTTT	TGGTCTCAGACACCACTTTGCC
*NECTIN3*	ATTCCCGCTTGGAAATGCCCAG	GCTGCTACTGTTTCATTTCCTCC
*PLAU*	GGCTTAACTCCAACACGCAAGG	CCTCCTTGGAACGGATCTTCAG
*RPS18*	CTTTGCCATCACTGCCATTAAG	TCCATCCTTTACATCCTTCTGTC
*TNS3*	CAGTCAGCACAAAGGAGGACGT	GCAAAAGCCTGCTGAAAGGAGG

### Genome-wide mRNA expression profiling

Total RNA was isolated from 1 × 10^6^ NCI-H661 cells per sample using the RNeasy kit (Qiagen). The integrity of samples was verified on an Agilent Technologies 2100 Bioanalyzer using the Agilent RNA 6000 Nano kit with an RNA integrity number (RIN) above 9.5 as threshold for acceptance. At this stage, samples were shipped to BGI Genomics Co. Ltd for further processing. Total RNA was subjected to Oligo dT selection for enrichment of mRNA followed by reverse transcription and second strand synthesis. Following cDNA synthesis and library preparation, samples were analyzed on the BGISEQ-500 platform with 50 bp end sequencing and a minimum of 20 M clean reads per sample. Sequence reads were filtered with SOAPnuke software to remove read with adapters, unknown bases, and low-quality reads. Then, genome mapping of filtered reads to reference genome GRCh38 was performed using HISAT2 and Bowtie2 software [65, 66]. Next, gene expression levels were calculated with RSEM [67]. Finally, differentially expressed genes were detected with NOIseq [68].

### Copy number alteration analysis

Purified DNA was processed by CytoScan HD assay (Thermo Fisher) according to the manufacturer´s instructions. The.CEL files from the CytoScan arrays were imported into NEXUS v10.0 (BioDiscovery) and used for the analysis and visualization of copy number alterations (CNAs) using NCBI Build 37 as reference. The samples were pre-processed by systematic correction (Quadratic), probes were re-centered by median and applying mean of Combine Replicates Between Arrays. Subsequently, data were processed by SNP-FASST2 Segmentation with significance threshold of 1.0E-8 and max contiguous probe spacing of 1000-bp with minimum of three probes per segment. Copy number call, including LOH, was assessed by visual inspection.

### Statistical analyses

Student's *t* tests (unpaired, two-tailed, unequal variance) and log-rank tests were performed as described previously [69]. In all figures *, ** and *** denote *p* < 0.05, 0.025, and 0.01, respectively.

## Supplementary information

Supplementary information
