# Supplementary material for: p190A RhoGAP induces CDH1 expression and cooperates with E-cadherin to activate LATS kinases and suppress tumor cell growth
Source: Oncogene. 2020 Jul 8;39(33):5570–87. doi: 10.1038/s41388-020-1385-2 (PMC7426264; doi:10.1038/s41388-020-1385-2)
Supplement: Supplementary file 1 — Supplementary information [file 41388_2020_1385_MOESM1_ESM.pdf]

Supplementary Figure S1

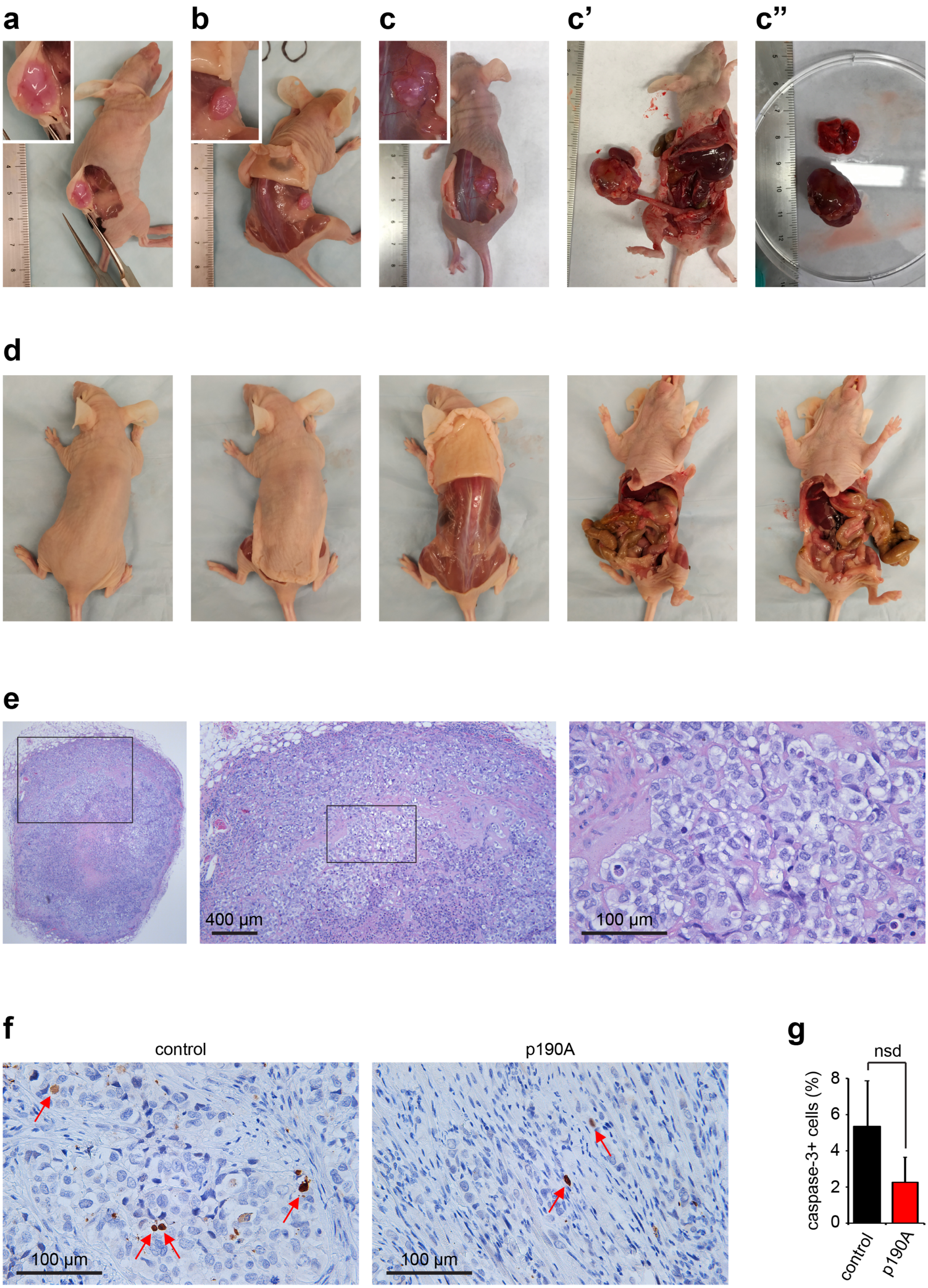

Figure S1: Validation of p190A tumor suppressor function in nude mice. (**a-c**): H661 cells form tumors and metastases in nude mice. (**a-b**): Examples of control cell tumors in mice without signs of local invasion or metastasis. (**c**): Example of a mouse injected with control cells in which primary tumor has invaded through the back musculature and into the abdominal cavity. (**c'**): large intrabdominal metastases from the same mouse as shown in **c**, which was found to be adherent to both right kidney and intestines. (**c"**): Size comparison between primary tumor (top) and metastasis (bottom) from the same mouse as shown in **c**. (**d**): Example of mouse injected with H661-p190A cells and sacrificed after 25 weeks with no signs of tumor or clinical manifestations of disease. The five images illustrate how each mouse injected with H661-p190A cells was scrutinized for evidence of tumor growth or other abnormalities. (**e**): Representative histology (H&E) of tumor from mice injected with control H661 cells. Tumor cell morphology is consistent with large cell lung cancer from which the H661 cell line is derived. (**f**): Representative images from cleaved caspase-3 staining of control and H661-p190A cell tumors. Red arrows point to examples of positively stained cells. (**g**): Quantification of cleaved caspase-3 positive cells from (**f**). Data are presented as mean ± SD; Student *t* test.

Supplementary Figure S2

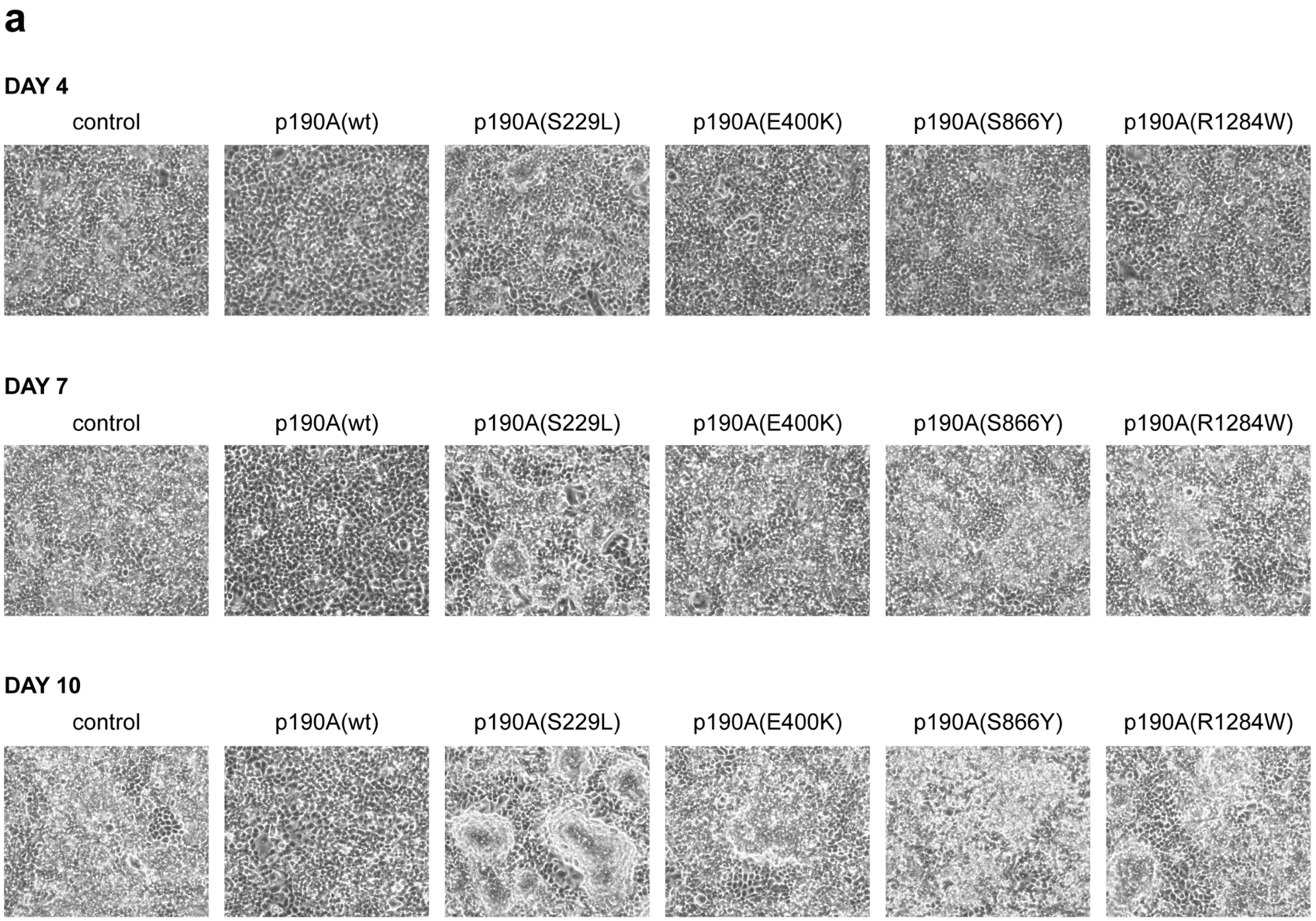

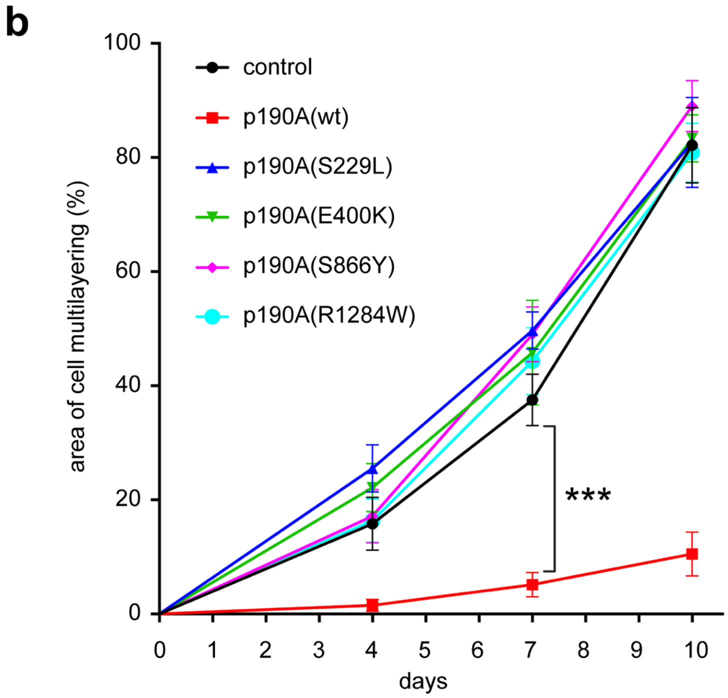

Figure S2: Determination of area of cell multilayering control cells as well as H661 cells expressing p190A(wt), p190A(S229L), p190A(E400K), p190A(S866Y), and p190A(R1284W). (**a**): Representative phase images sampled on days 4, 7 and 10 after plating $1x10^6$ cells per well of 6-well dishes. (**b**): Quantification of area of cell multilayering. Data are presented as mean ± SD (n=3); Student *t* test, *** p<0.01.

Supplementary Figure S3

a

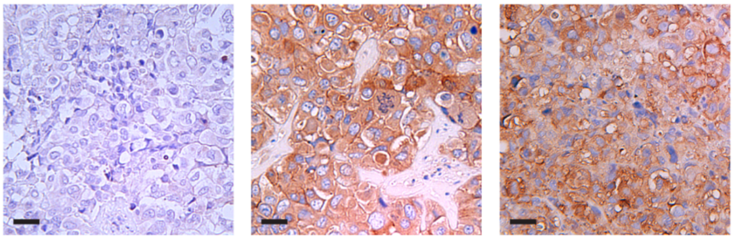

b

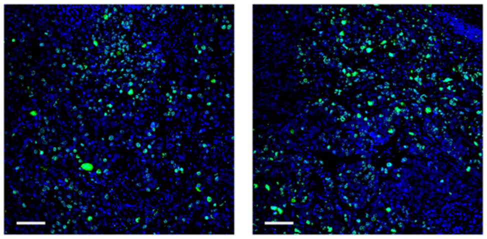

c

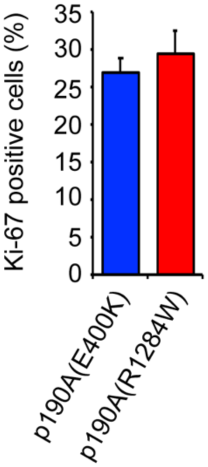

d

	control	p190A(wt)	p190A(E400K)	p190A(R1284W)
control	X	p<0.001	p<0.01	p<0.05
p190A(wt)	X	X	p<0.05	p<0.001
p190A (E400K)	X	X	X	n.s.d.
p190A (R1284W)	X	X	X	X

Figure S3: Effects of recurrent *ARHGAP35* mutations on tumor suppression. (**a**): Immunohistochemistry to detect p190A in tumors from mice injected with control or H661 cells expressing p190A(E400K) or p190A(R1284W). Scale bar 20 µm. In contrast to Fig. 2E, these samples we obtained when the mice were sacrificed after tumors had reached a size of 8-mm in maximum length. (**b**): Ki-67 (green) and Hoechst 33342 (blue) staining of tumors from mice injected with H661 cells expressing p190A(E400K) and p190A(R1284W), respectively. Scale bar 100µm. (**c**): Quantification of Ki-67 positive nuclei from (**b**). Data are presented as mean ± SEM. The difference in percent Ki-67 positive nuclei between in H661 cells expressing p190A(E400K) or p190A(R1284W) is not significant. (**d**): Log-rank test results for Kaplan-Meier survival plot for mice injected with control, H661-p190A, or H661 cells expressing p190A(E400K) or p190A(R1284W), as shown in Fig. 3h. Of note, two E400K mice were excluded due to the appearance of what we perceived to be minute skin metastases, which is a criterium for euthanasia in our IACUC approved protocol. However, because the processes were very small and spread out and we considered it unrealistic to perform histology. For this reason, they were censured from the analysis.

Supplementary Figure S4

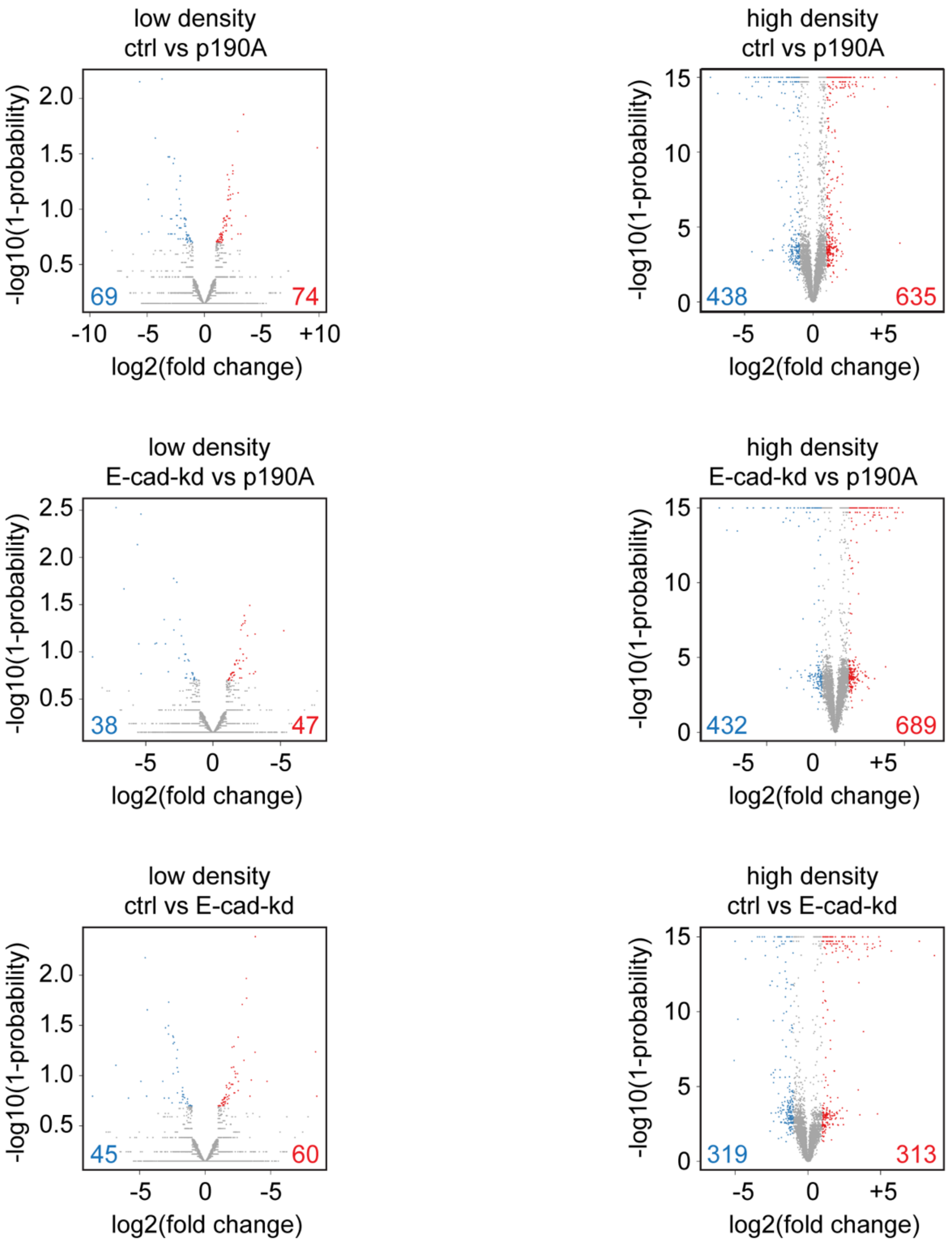

Figure S4: Regulation of gene expression by p190A and E-cadherin. Volcano plots of pairwise comparisons of differentially expressed genes in control H661 and H661-p190A cells with or without E-cadherin knockdown cultured at low and high cell density.

Supplementary Figure S5

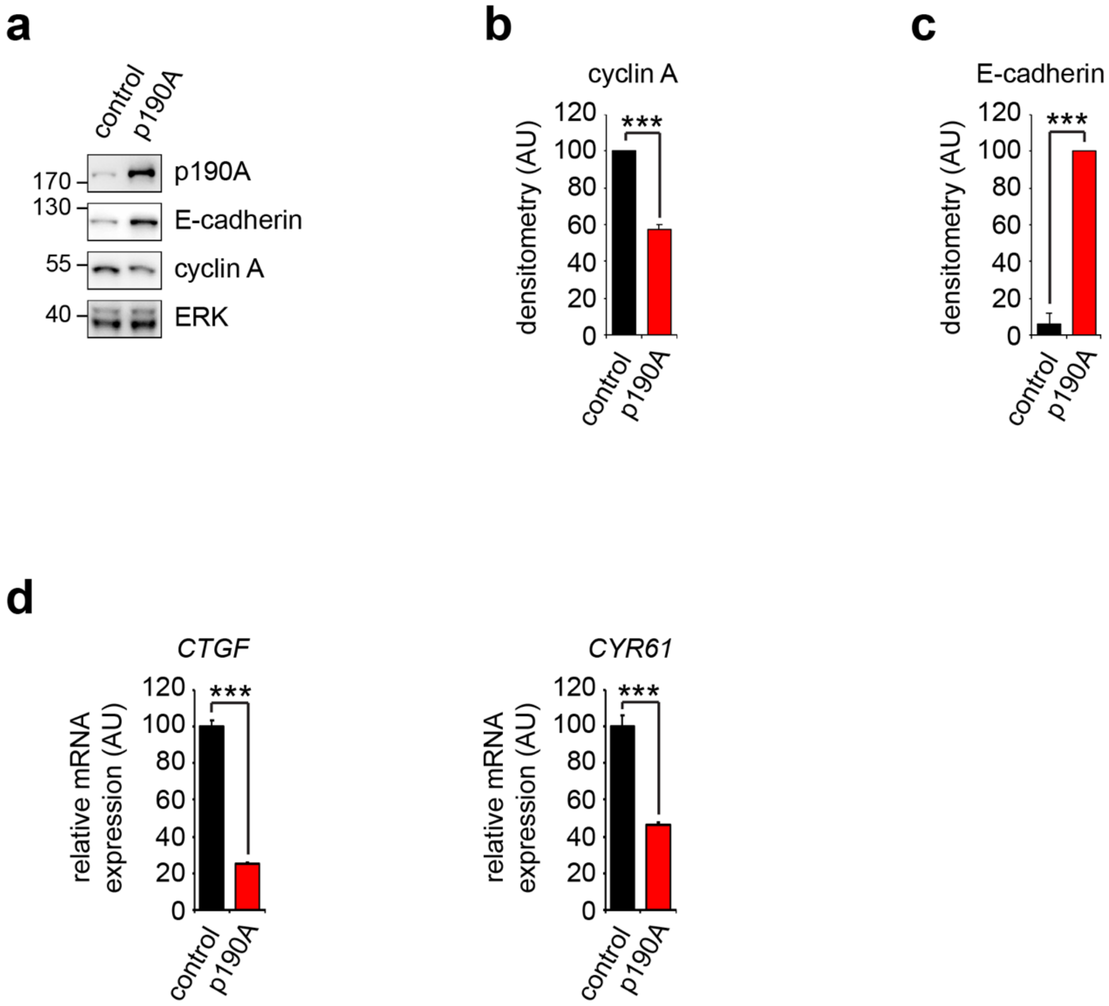

Figure S5: p190A activates the Hippo pathway, induces E-cadherin expression and promotes CIP in H226 cells cultured at high cell density. (**a**): Western blots of whole cell lysates to detect E-cadherin and cyclin A in H226 cells with or without exogenous expression of p190A. (**b**): Quantification by densitometry of cyclin A levels from experiments as shown in **a**. Data are presented as mean ± SD (n=3). Student *t* test, *** $p<0.01$. (**c**): Quantification by densitometry of E-cadherin levels from experiments as shown in **a**. Data are presented as mean ± SD (n=3). Student *t* test, *** $p<0.01$. (**d**): qPCR analyses to quantify transcript levels for YAP target genes *CTGF* and *CYR61* in NCI-H226 cells cultured at high cell density. Data are presented as mean ± SD (n=3). Student *t* test, *** $p<0.01$; ** $p<0.025$.

Supplementary Figure S6

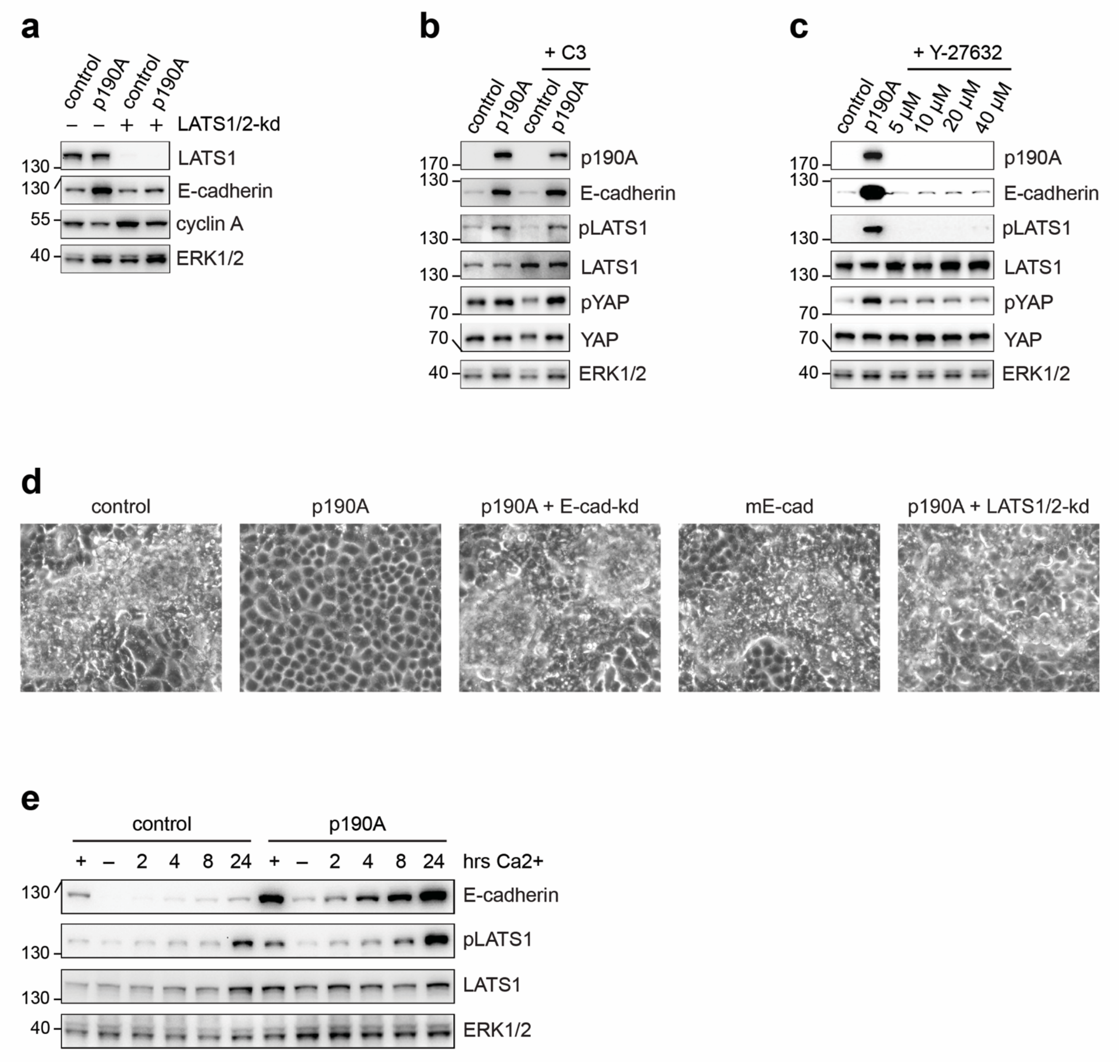

Figure S6: Role of LATS1/2 and Rho signaling in modulation of E-cadherin expression and CIP by p190A. (**a**): Western blotting of whole cell lysates from control and H661-p190A cells without and with LATS1/2 knockdown to detect LATS1, E-cadherin, and cyclin A. (**b**): Western blotting of whole cell lysates from control and H661-p190A cells with or without treatment of Clostridium botulinum C3 toxin for 18 hours. (**c**): Western blotting of whole cell lysates from control and H661-p190A cells incubated with or without indicated concentrations of ROCK inhibitor Y-27632 for 7 days. (**d**): Phase images of control H661 cells with or without mouse E-cadherin expression, as well as H661-p190A cells with or without E-cadherin depletion or LATS1/2 knockdown. (**e**): Calcium switch assay performed for control H661 and H661-p190A cells followed by western blotting of whole cell lysates to detect E-cadherin, pLATS1, total LATS1, and cyclin A.

Supplementary Tables

Table S1a: ctrl vs p190A(WT)

Table S1b: p190A(E400K) vs p190A(WT)

Table S1c: p190A(R1284W) vs p190A(WT)

Table S1d: ctrl vs p190A(E400K)

Table S1e: ctrl vs p190A(R1284W)

Table S1f: p190A(E400K) vs p190A(R1284W)

Table S2a: ld ctrl vs ld p190A

Table S2b: ld p190A w E-cad-kd vs ld p190A

Table S2c: ld ctrl vs ld p190A w E-cad-kd

Table S2d: hd ctrl vs hd p190A

Table S2e: hd p190A w E-cad-kd vs hd p190A

Table S2f: hd ctrl vs hd p190A w E-cad-kd

Table S1a

Gene ID	Gene symbol	ctrl Expression	p190A Expression	log2FoldChange (p190A/ctrl)
4071	TM4SF1	4.901367058	190.2880715	5.278857124
93649	MYOCD	1.725662755	41.17431145	4.576522071
56892	TCIM	47.56853204	1124.240696	4.56279963
6588	SLN	18.67300524	384.4076282	4.363610926
94240	EPSTI1	13.97177481	281.1844014	4.330929363
26289	AK5	10.5322342	180.8737779	4.102099856
2846	LPAR4	5.091392925	83.76205294	4.040164488
6641	SNTB1	1.996838552	32.11307773	4.007371342
4222	MEOX1	306.9174885	4822.139873	3.973750739
3815	KIT	8.494936665	132.8585717	3.967144308
9411	ARHGAP29	4.060213958	63.40481528	3.964964751
11098	PRSS23	447.3360996	6873.216289	3.941554267
5999	RGS4	11.92423639	178.0720716	3.900492478
5796	PTPRK	29.20882443	435.8373549	3.899313653
51702	PADI3	39.52459085	497.4808749	3.653818631
55024	BANK1	5.691455413	70.38533449	3.628405331
2890	GRIA1	2.96919614	36.07098986	3.60269471
127254	ERICH3	1.587933683	18.70459001	3.558169776
8660	IRS2	7.036544089	81.26282886	3.529656641
221981	THSD7A	21.00739623	228.3686518	3.442395363
84419	C15orf48	7.021905903	73.30703001	3.384016988
55170	PRMT6	37.13484041	373.3401465	3.329645371
644019	CBWD6	1.184350401	11.5918032	3.290937122
728118	NUTM2A	9.74697454	94.18083777	3.272407175
57282	SLC4A10	1.569226047	15.15458132	3.271628903
140706	CCM2L	251.063096	2398.444275	3.255977034
3777	KCNK3	11.73680349	111.7268151	3.250864032
4629	MYH11	40.19541309	381.4381231	3.24634626
10335	MRVI1	1.198713898	11.27737628	3.233872188
84166	NLRC5	3.931494229	36.08015562	3.198055919
960	CD44	36.45522846	331.3805483	3.184291272
117154	DACH2	13.20244373	118.8914795	3.170768429
11309	SLCO2B1	1.589011314	14.17030775	3.156669789
286	ANK1	148.2658621	1289.916972	3.121019843
1015	CDH17	2.873011182	24.68376157	3.102926751
4110	MAGEA11	2.580351882	22.07404945	3.09671159
116441	TM4SF18	1.558183667	13.23943339	3.086904178
50964	SOST	3.245464661	26.92654413	3.052532128
165	AEBP1	44.5552959	352.0383175	2.98206364
167838	TXLNB	30.33876173	231.6684306	2.932827353

728130	NUTM2D	7.993684968	60.71086015	2.925021991
51286	CEND1	50.61677333	383.3998623	2.921162371
10873	ME3	15.79333631	119.3146099	2.917382835
338596	ST8SIA6	4.254619668	31.88178888	2.905630505
246	ALOX15	17.74850993	130.6729067	2.880190235
3106	HLA-B	86.55225468	632.4924218	2.869404887
4005	LMO2	10.36645292	74.99583112	2.854888068
1800	DPEP1	145.1711266	1033.477371	2.831680353
23544	SEZ6L	14.43741755	101.5102494	2.813740789
1794	DOCK2	1.803986468	12.63561296	2.808235229
3751	KCND2	3.854313254	26.65284707	2.789743916
4907	NT5E	147.6728238	1021.028824	2.789547338
3013	HIST1H2AD	1.167261964	8.032039485	2.782637985
1828	DSG1	12.05807818	81.00337833	2.74798209
203859	ANO5	14.78685273	99.0224625	2.743440809
89927	C16orf45	176.8232267	1180.760268	2.739336383
169792	GLIS3	5.577548247	37.07660147	2.732806016
59277	NTN4	170.719383	1127.293191	2.723164014
22822	PHLDA1	585.749784	3848.947316	2.716107502
166979	CDC20B	3.188024661	20.75104134	2.702449042
105372978	LINC01638	4.939191572	31.81017061	2.687141278
22882	ZHX2	26.19427036	168.639646	2.686620562
339761	CYP27C1	28.32893757	180.1795977	2.669087257
55531	ELMOD1	9.952792195	62.98580432	2.661853485
347365	ITIH6	4.267870019	26.95727872	2.659086723
51809	GALNT7	18.71126602	117.6620464	2.652669953
388662	SLC6A17	23.13384248	145.4672067	2.652617138
776	CACNA1D	1.082728649	6.795264571	2.649857999
4916	NTRK3	28.16876486	175.6015736	2.640137562
147409	DSG4	4.568116908	28.25902553	2.629040239
8829	NRP1	2.189839683	13.53861641	2.62818315
344901	OSTN	4.913919191	30.22417511	2.620756927
2303	FOXC2	5.158118883	31.6150615	2.615695097
2857	GPR34	2.122730048	12.97556034	2.611804024
7062	TCHH	1.065885934	6.466183116	2.600861308
4939	OAS2	2.832687862	17.16605804	2.599315238
6236	RRAD	14.98862239	90.45544925	2.593339626
800	CALD1	67.49083739	406.3842768	2.590081025
7042	TGFB2	818.2488501	4926.011013	2.589808278
11010	GLIPR1	84.20677799	504.7595944	2.58358816
117581	TWIST2	1.40883405	8.434187363	2.581747388
2254	FGF9	4.155318764	24.72379465	2.572869129
84709	MGARP	36.96889676	218.4860571	2.56315732

140862	ISM1	3.580440787	21.03239575	2.55440408
59352	LGR6	1.436670978	8.41043684	2.549451038
999	CDH1	356.1502182	2064.225368	2.535042711
7058	THBS2	13.41329065	77.67213119	2.533733838
51232	CRIM1	830.2240541	4768.913747	2.522088053
154	ADRB2	1.408709979	8.077004719	2.519445758
115701	ALPK2	9.730200483	55.7259857	2.517808794
8707	B3GALT2	6.562649878	37.49502907	2.514348971
639	PRDM1	24.76381028	140.598096	2.505271841
182	JAG1	1037.045487	5879.280143	2.503160347
23316	CUX2	31.33694031	176.9809344	2.497657723
3306	HSPA2	1595.287419	8994.977006	2.49530322
5028	P2RY1	28.19390753	157.9541071	2.486050104
4608	MYBPH	18.85995964	105.553099	2.484570443
4135	MAP6	112.7299551	628.8926118	2.479942761
8605	PLA2G4C	25.45710617	141.6092921	2.4757756
10046	MAMLD1	63.70474963	352.3343385	2.467472242
9235	IL32	12.73836516	70.34702753	2.46530933
3489	IGFBP6	82.98879464	455.2813725	2.455769976
55450	CAMK2N1	88.08561973	481.2387537	2.449774408
3239	HOXD13	9.963377371	54.09087574	2.440678482
80326	WNT10A	222.347032	1206.477558	2.439916027
1381	CRABP1	126.9707601	685.5709431	2.432809665
11096	ADAMTS5	1.083312727	5.819296435	2.425394964
85463	ZC3H12C	2.854820779	15.23030997	2.415473221
4940	OAS3	176.1974919	934.3928158	2.406835793
91683	SYT12	75.09117108	398.202431	2.406786832
1435	CSF1	395.6428029	2094.652066	2.404440205
2300	FOXL1	4.129078372	21.85481493	2.404059455
5915	RARB	4.141342894	21.84696392	2.399262237
131544	CRYBG3	6.876899181	36.25277687	2.398261406
84630	TTBK1	17.31388882	91.18480321	2.396863605
7164	TPD52L1	112.9219764	593.8383596	2.394744004
3167	HMX2	1.363967427	7.158633624	2.391875053
7262	PHLDA2	17.31531079	90.54129167	2.386527606
1839	HBEGF	511.268394	2664.626588	2.381780624
130576	LYPD6B	30.66942406	159.4118277	2.377885696
161145	TMEM229B	13.97089194	72.61374291	2.37781849
6422	SFRP1	41.32827094	214.6831559	2.377008089
10568	SLC34A2	568.4423198	2917.832237	2.359811066
399939	TRIM49D1	1.066698935	5.45150129	2.353500541
9211	LGI1	46.06107809	234.7798341	2.349688413
5992	RFX4	4.210131008	21.4146112	2.346658453

5698	PSMB9	31.74949752	161.3736588	2.345599441
83593	RASSF5	21.72085446	110.1642961	2.342503964
4773	NFATC2	170.5649263	864.3382035	2.341274918
1901	S1PR1	1.352544833	6.838440083	2.337990853
54885	TBC1D8B	4.111863526	20.54694572	2.321059668
29943	PADI1	8.135556991	40.52206011	2.316394495
55466	DNAJA4	30.84451327	153.5797933	2.315902626
9394	HS6ST1	1815.737695	8945.405886	2.300591141
340156	MYLK4	3.778993495	18.57420175	2.29722627
51332	SPTBN5	10.92430997	53.64136095	2.295803686
80099	C7orf69	3.057487957	15.01208303	2.295705453
5581	PRKCE	2.723710204	13.36244348	2.294538728
79722	ANKRD55	0.996633338	4.883843148	2.292882128
28996	HIPK2	215.683824	1051.744856	2.285794877
84871	AGBL4	2.07925362	10.07883844	2.277191733
389257	LRRC14B	7.575989609	36.72158034	2.27712189
8638	OASL	25.18469933	121.7196112	2.272942218
2909	ARHGAP35	3201.932358	15442.91183	2.269930069
84541	KBTBD8	2.712206438	13.03108243	2.264418028
8061	FOSL1	35.8579872	171.7981487	2.260348077
3236	HOXD10	22.3589304	106.9799422	2.258417249
55806	HR	117.0683628	553.0514952	2.240062571
3237	HOXD11	38.89055376	183.2750699	2.236518873
153	ADRB1	2.706427558	12.70720295	2.231184828
2247	FGF2	596.6363891	2789.732815	2.225203079
388585	HES5	154.7483294	722.1658293	2.222406323
3371	TNC	1.678368456	7.828094434	2.221601691
11240	PADI2	274.9566022	1281.561874	2.2206273
6999	TDO2	15.54892413	72.29978708	2.217176638
169355	IDO2	0.989797028	4.597104155	2.215520738
388849	CCDC188	4.084928889	18.94539994	2.213464727
79836	LONRF3	21.66806026	99.44502825	2.198329237
2983	GUCY1B1	112.6814845	516.8796823	2.197578019
347902	AMIGO2	39.20957547	179.6839239	2.196183411
54809	SAMD9	2.428263812	11.08229418	2.190259496
2313	FLI1	2.42076758	11.02503194	2.187246359
23569	PADI4	1.657531276	7.518963184	2.181497645
57158	JPH2	40.62067375	184.0474658	2.179791811
9619	ABCG1	79.06895287	357.6465291	2.177351217
132671	SPATA18	1.316797358	5.910891866	2.166342482
162494	RHBDL3	473.1925121	2112.300578	2.158315994
387849	REP15	2.421678299	10.73063109	2.147655795
133396	IL31RA	6.503087617	28.67998034	2.140847269

131578	LRRC15	10.47573942	46.16648426	2.139793792
219621	CABCOCO1	1.646601178	7.213874124	2.131283086
79819	WDR78	9.129321238	39.92292174	2.128637802
3965	LGALS9	10.17763223	44.43578952	2.126320156
57522	SRGAP1	662.993276	2876.532496	2.117264625
90627	STARD13	4.380473087	18.96734079	2.114358835
55824	PAG1	1207.373416	5175.24852	2.099756202
80144	FRAS1	0.930243318	3.980893292	2.097412173
168667	BMPER	5.375333516	22.9236314	2.092409428
84969	TOX2	58.59327914	249.636278	2.09102051
5654	HTRA1	14.26526679	60.75005145	2.090378902
644538	SMIM10	9.882872528	42.08254549	2.090219635
2312	FLG	2.071044926	8.800057431	2.08715409
7056	THBD	4.012031784	17.03837793	2.086383056
388021	TMEM179	29.09534937	122.9620797	2.079352996
80243	PREX2	91.31146992	385.6855438	2.078557074
1734	DIO2	2.705333118	11.40378991	2.075635212
1012	CDH13	2.712154007	11.41394598	2.073286633
825	CAPN3	11.13962993	46.80896892	2.071083681
340061	TMEM173	29.4106997	122.6816959	2.060507003
83697	SLC4A9	3.706535611	15.35245484	2.050326082
5155	PDGFB	131.0337659	540.7401005	2.044996723
9148	NEURL1	24.13880405	99.54962115	2.044061625
90293	KLHL13	11.31493543	46.59273895	2.04187679
7043	TGFB3	77.00114493	316.7578838	2.040428725
85352	SHISAL1	11.8126455	48.40766183	2.034903312
145376	PPP1R36	20.95452454	85.69771759	2.031994995
100287482	SMKR1	15.20092169	62.10709466	2.030599278
54625	PARP14	100.3562097	409.9112199	2.030181591
144535	CFAP54	14.9804777	61.17202102	2.029788311
6710	SPTB	30.44550097	124.2957493	2.029476003
60506	NYX	2.331836401	9.485300664	2.024226931
728392	LOC728392	149.830421	608.7827493	2.022596906
1830	DSG3	16.30234208	65.32388209	2.002531286
23551	RASD2	27.0508965	108.3193765	2.001543028
11043	MID2	138.319088	553.1047994	1.999552599
5079	PAX5	1.97609989	7.89476751	1.998240905
647310	TEX22	42.97494741	170.2051149	1.985706614
3754	KCNF1	3.329058355	13.10282138	1.976691431
9478	CABP1	43.97771973	172.2281766	1.969476481
861	RUNX1	20.21739317	78.92817699	1.964943439
1141	CHRNB2	45.2286564	174.4613378	1.947598314
58489	ABHD17C	5.070695789	19.54259562	1.946366468

1840	DTX1	110.3757031	422.473777	1.936439168
5950	RBP4	6.080220279	23.14816087	1.928702078
26115	TANC2	1618.617575	6160.218064	1.928219256
2274	FHL2	6.032821952	22.94157556	1.927059565
90102	PHLDB2	13.8197682	52.35932862	1.921713181
1812	DRD1	0.892290168	3.35694597	1.91156447
80833	APOL3	1.934319889	7.267271056	1.909587316
24	ABCA4	6.376481506	23.92861274	1.907904279
64919	BCL11B	1.243782325	4.663748425	1.906755947
7424	VEGFC	10.83440848	40.59034047	1.905516052
2171	FABP5	81.01405355	300.4121435	1.890699032
59269	HIVEP3	26.41181206	97.23783806	1.880334532
5507	PPP1R3C	71.20128436	261.8642271	1.878843818
130574	LYPD6	9.363419629	34.41899494	1.87809755
254778	C8orf46	15.85079905	58.20354168	1.876551374
8357	HIST1H3H	39.86138814	146.240293	1.875277009
84141	EVA1A	4.658615301	17.08109694	1.874427521
2104	ESRRG	190.191775	696.7242995	1.873133024
55509	BATF3	36.49644246	133.3487406	1.869376452
347732	CATSPER3	2.264572818	8.248099624	1.864822828
144501	KRT80	3.622850668	13.15971454	1.860930949
4103	MAGEA4	14.09504523	51.14779846	1.859484036
84072	HORMAD1	29.34236584	106.1663186	1.855269039
4994	OR3A1	4.283740647	15.46846256	1.852386769
627	BDNF	37.43769984	135.1194972	1.851672161
5457	POU4F1	27.3163746	98.57039318	1.851388357
8651	SOCS1	53.97659316	194.6925593	1.850791923
54935	DUSP23	21.67488763	77.82826084	1.844269605
2669	GEM	19.87348299	71.33148628	1.843694295
140733	MACROD2	38.21118886	136.7971468	1.83997109
100505767	SPDYE18	0.995054545	3.559845262	1.838967016
10391	CORO2B	3.394737602	12.09852113	1.833458739
22998	LIMCH1	39.42649193	140.1855949	1.830100856
91624	NEXN	248.2497083	881.8025216	1.82866358
28513	CDH19	16.80631835	59.62479101	1.826908587
57822	GRHL3	20.21710648	71.32469824	1.818825208
102724428	SIK1B	318.0536558	1121.902288	1.818604956
54453	RIN2	77.34118427	271.696639	1.812687958
25884	CHRDL2	2.263098512	7.927163528	1.808505351
3235	HOXD9	73.61543574	256.9038832	1.803148489
330	BIRC3	71.5291674	249.4028781	1.80187456
23708	GSPT2	100.6546192	350.8778171	1.80155536
84627	ZNF469	12.39097514	42.95820978	1.793644144

5140	PDE3B	1.249139731	4.327662749	1.792653207
284076	TTLL6	170.8296604	591.6305836	1.792138147
55567	DNAH3	158.956853	550.1103187	1.791085749
11023	VAX1	14.24769693	49.30592514	1.791032293
8435	SOAT2	1.546590918	5.344095849	1.788854237
2006	ELN	23.5804806	81.23476978	1.784504233
25858	CATSPERZ	3.921099035	13.5076195	1.78444346
2878	GPX3	60.45488394	207.9874506	1.782565683
56912	IFT46	80.64395766	276.564557	1.777977934
57460	PPM1H	5.95003595	20.40130632	1.777691243
101928841	LOC101928841	17.12775661	58.37042377	1.768901342
7475	WNT6	180.2754607	613.8572351	1.767700138
653149	NBPF6	19.98850282	68.03880199	1.767187323
146	ADRA1D	19.15658459	65.18848009	1.766776671
266727	MDGA1	48.88216397	166.0893162	1.764579216
3755	KCNG1	165.0765668	559.7317739	1.761600307
378948	RBMY1B	0.82359854	2.774049759	1.751980488
4211	MEIS1	8.676763856	29.19477614	1.750481278
64065	PERP	50.54881296	169.9329686	1.749216657
642968	FAM163B	2.266716528	7.619218972	1.749039137
54738	FEV	14.64359162	49.19440607	1.748224829
26230	TIAM2	84.09732318	281.9683229	1.74540131
3620	IDO1	24.03121368	80.47411802	1.743615348
116496	FAM129A	422.4680339	1414.075973	1.742945544
257019	FRMD3	185.1811331	618.9018314	1.740773471
7104	TM4SF4	0.832134737	2.777601929	1.738950805
943	TNFRSF8	7.976390348	26.60602673	1.73794516
10761	PLAC1	55.32568193	184.4100781	1.736896269
2766	GMPR	19.17730495	63.83993072	1.735059098
127294	MYOM3	102.5474322	341.208446	1.734361991
2048	EPHB2	259.0126065	861.0487831	1.733072659
53335	BCL11A	142.7601641	473.6530538	1.730237221
221061	FAM171A1	135.082054	447.9719838	1.729572487
1001	CDH3	3721.145932	12337.28911	1.729206549
8038	ADAM12	1.215718981	4.016807034	1.724239374
5409	PNMT	4.586981837	15.14983832	1.723685297
2863	GPR39	11.36510771	37.39862598	1.71837391
9568	GABBR2	22.51900206	74.06788508	1.717705248
8372	HYAL3	18.83962776	61.86994027	1.715468181
198437	LKAAEAR1	26.315954	86.3576223	1.714385826
56145	PCDHA3	8.007371191	26.26084478	1.713512736
144347	RFLNA	74.95774394	245.2962286	1.710375616
684	BST2	13.76574441	44.87493789	1.704827314

23670	TMEM2	43.86648197	142.8359904	1.703168627
6783	SULT1E1	0.84779832	2.755006295	1.700262602
164633	CABP7	9.311361115	30.22687143	1.698767685
84532	ACSS1	59.66027896	192.8250833	1.692450105
23600	AMACR	606.2222122	1954.389547	1.688799433
9573	GDF3	5.623520225	18.12057677	1.688083456
24141	LAMP5	182.2721371	586.8728651	1.686953963
51764	GNG13	19.82188096	63.56770736	1.681200186
134	ADORA1	20.86559942	66.80502967	1.678830359
102724265	LOC102724265	1.171067858	3.749306371	1.678799043
26030	PLEKHG3	251.1058909	803.6794956	1.678324401
84665	MYPN	23.6380784	75.55101257	1.676338331
118881	COMTD1	161.0223531	512.4906259	1.670264639
643008	SMIM5	4.656491505	14.72814126	1.661260118
146664	MGAT5B	284.3561585	897.6591591	1.658468708
26499	PLEK2	7.606937878	24.00760597	1.658103817
64135	IFIH1	36.3892753	114.8298491	1.657912483
50651	SLC45A1	119.0457271	375.557691	1.657518704
27344	PCSK1N	748.1571258	2359.947853	1.657341784
9645	MICAL2	74.26668714	233.1020576	1.65017461
10083	USH1C	6.939566963	21.74021817	1.647448874
1823	DSC1	106.3607435	333.1107251	1.647036038
23220	DTX4	29.30714665	91.69325463	1.645563093
27190	IL17B	1.515502002	4.738664798	1.644684854
9882	TBC1D4	0.794711732	2.483408797	1.643818217
64759	TNS3	3088.88607	9651.716211	1.643698837
10788	IQGAP2	3.586815912	11.20404068	1.643243518
6372	CXCL6	0.792749847	2.473282316	1.641489328
8787	RGS9	48.80843306	152.2679951	1.641410395
6273	S100A2	0.798113343	2.486495303	1.639448158
8360	HIST1H4D	0.798113343	2.486495303	1.639448158
339977	LRRC66	5.605588771	17.44132739	1.637572025
7401	CLRN1	0.79621759	2.476746622	1.637211635
122402	TDRD9	0.795160269	2.471315827	1.635961816
8714	ABCC3	9.993763474	30.89124343	1.628097965
80003	PCNX2	2.169994818	6.696867355	1.625794795
81706	PPP1R14C	91.74640755	282.7257115	1.623679516
56147	PCDHA1	46.60755503	143.4752416	1.622166066
84456	L3MBTL3	26.85402125	82.66319049	1.622106907
2334	AFF2	611.5851693	1882.285686	1.621860284
57168	ASPHD2	23.31474341	71.69425156	1.620614896
4330	MN1	1254.779292	3851.281043	1.617904781
100130705	LOC100130705	21.56754046	66.15398688	1.616966443

1991	ELANE	44.08039662	135.1967495	1.616851356
54463	RETREG1	23.53853459	72.06076262	1.614189413
3604	TNFRSF9	43.42693757	132.9321021	1.614027423
2022	ENG	210.3142998	643.7728394	1.614004766
4300	MLLT3	6.254601314	19.1390774	1.613531455
91050	CCDC149	62.67489472	191.0557105	1.608033807
360	AQP3	13.70519789	41.73301228	1.606465898
80830	APOL6	55.21754605	167.9789728	1.605081973
203111	ERICH5	29.25935242	88.84067731	1.602322553
51289	RXFP3	0.811261316	2.46214683	1.601678198
83666	PARP9	116.7038148	354.0700159	1.601182955
85444	LRRCC1	30.57774807	92.74394422	1.600770921
8970	HIST1H2BJ	15.37786589	46.62144252	1.600138341
5328	PLAU	274.708101	832.6931254	1.599885458
1303	COL12A1	173.3091766	524.3714094	1.597240981
2615	LRRC32	13.04508482	39.43756516	1.596064159
23678	SGK3	96.88416963	292.8486211	1.59582224
728	C5AR1	3.674793467	11.10381507	1.595320372
3918	LAMC2	8.273444593	24.99614204	1.595145427
286133	SCARA5	8.608769492	25.97773445	1.593396674
26064	RAI14	180.2008038	543.733827	1.593295139
79690	GAL3ST4	95.23582874	287.0740707	1.59184669
221662	RBM24	320.4766448	964.5830495	1.589686236
4938	OAS1	1.477574194	4.443328765	1.588410317
55068	ENOX1	3.537573014	10.63712116	1.588275921
161436	EML5	9.042518096	27.17417114	1.587439546
6563	SLC14A1	1.561632223	4.692218672	1.587215522
122773	KLHDC1	29.53973251	88.49688777	1.582969958
4137	MAPT	803.037077	2401.006742	1.580100949
3199	HOXA2	41.37110584	123.5619635	1.578539276
25945	NECTIN3	49.48195809	147.5111284	1.575849301
389692	MAFA	49.81785448	148.3091548	1.57387286
5139	PDE3A	1067.776322	3175.39213	1.5723253
55231	CCDC87	8.965320819	26.65002318	1.571709672
374973	TEX38	1.821569247	5.410153321	1.57048764
8329	HIST1H2AI	14.77469342	43.8691258	1.570077763
9806	SPOCK2	7.229656703	21.46176776	1.569769865
9540	TP53I3	429.014686	1273.201703	1.569362052
56134	PCDHAC2	91.54820457	271.2293889	1.566910012
862	RUNX1T1	291.9515419	864.6643834	1.566411333
80231	CXorf21	4.233471795	12.53641168	1.566211279
51315	KRCC1	97.86254512	289.7665578	1.566062393
494143	CHAC2	28.16688599	83.31889007	1.564643546

29118	DDX25	15.33901529	45.29757517	1.562227953
117531	TMC1	17.02590367	50.2633785	1.561776273
2342	FNTB	151.3003025	446.6136195	1.561612375
8347	HIST1H2BC	30.91116334	91.14885526	1.560096587
3233	HOXD4	7.605490017	22.4167957	1.559466963
695	BTK	11.97115366	35.16286911	1.5544906
91584	PLXNA4	3.955575981	11.57917913	1.549573292
4254	KITLG	56.39273417	165.0648368	1.549451623
3875	KRT18	884.2991372	2582.341365	1.54607334
84913	ATOH8	21.1484989	61.60011504	1.542377779
190	NR0B1	28.3412563	82.48171578	1.541170633
9052	GPRC5A	41.09663921	119.6020863	1.541150232
3959	LGALS3BP	845.6009758	2449.622903	1.53451073
3363	HTR7	13.31253613	38.50062441	1.532096403
150221	RIMBP3C	11.96897355	34.5126217	1.527824636
2621	GAS6	353.8461505	1020.143128	1.52757745
11346	SYNPO	365.5682567	1050.621389	1.523030153
24138	IFIT5	103.6783642	297.8764452	1.522599184
5988	RFPL1	6.56127654	18.84990365	1.522508716
492	ATP2B3	3.495541489	10.02062746	1.519384985
11341	SCRG1	3.838793313	10.96459631	1.514127906
3205	HOXA9	13.08442533	37.3712644	1.514078814
140876	RIPOR3	547.6660487	1561.479951	1.511545694
8991	SELENBP1	35.58598504	101.0287952	1.505385472
100170229	SRRM5	3.59458559	10.20361643	1.505183212
8365	HIST1H4H	6.867402594	19.47952203	1.50412183
55332	DRAM1	188.8719244	535.2573962	1.502824564
64127	NOD2	2.839657209	8.045865866	1.502530902
3983	ABLIM1	12.6036257	35.55745679	1.496313326
1277	COL1A1	7072.12124	19933.28135	1.494964308
115207	KCTD12	19.72475101	55.44132204	1.490954571
5739	PTGIR	5.19284269	14.57800099	1.489196477
256130	TMEM196	4.233230503	11.87883307	1.488562167
50632	CALY	601.2170783	1686.389382	1.487979792
83857	TMTC1	195.4115328	547.985819	1.487622944
1747	DLX3	21.08877554	59.0087055	1.484452478
4855	NOTCH4	46.7511755	130.8005959	1.484294568
200958	MUC20	26.81668584	74.80911834	1.480083176
548596	CKMT1A	346.394813	966.1820224	1.479877682
80216	ALPK1	97.50693448	271.9454142	1.479740369
84002	B3GNT5	2.876374955	8.021947638	1.479700796
55638	SYBU	88.27058827	245.6593082	1.476654188
115708	TRMT61A	151.06282	420.3217337	1.476345432

83468	GLT8D2	183.1508455	508.8075209	1.474087635
84674	CARD6	5.860822508	16.2511465	1.47136645
283652	SLC24A5	4.990020513	13.82242118	1.469892693
55715	DOK4	1528.299327	4228.238074	1.468129479
29116	MYLIP	7.896627378	21.75110234	1.461779998
57571	CARNS1	118.307594	325.5379259	1.46028295
84074	QRICH2	55.45361322	152.3651593	1.458179675
219595	FOLH1B	32.76784528	89.82748102	1.454876069
255231	MCOLN2	10.58593142	28.98857694	1.4533363
387723	C10orf143	4.86213093	13.27171757	1.448694442
1769	DNAH8	20.69750104	56.48907752	1.44851535
1244	ABCC2	20.37918553	55.50192831	1.445441502
10150	MBNL2	534.7988748	1456.080845	1.445022124
6583	SLC22A4	17.02055007	46.32326467	1.444459269
283417	DPY19L2	51.7451206	140.2880755	1.438897652
100129654	TCF24	32.21105328	87.29701115	1.438376423
55520	ELAC1	22.04334492	59.73504694	1.438234459
124925	SEZ6	28.74235194	77.82895183	1.437128808
11247	NXPH4	22.72627302	61.42477451	1.434459547
57094	CPA6	51.48283732	138.971577	1.432626377
1308	COL17A1	3.121231929	8.423158825	1.432245806
203190	LGI3	5.527157506	14.89913042	1.4306185
151742	PPM1L	25.82879335	69.59690026	1.430042806
148545	NBPF4	108.4583851	291.84482	1.428059867
283576	ZDHHC22	32.61801727	87.74838797	1.427703533
25878	MXRA5	5.181882915	13.92852595	1.426494263
860	RUNX2	79.10305265	212.6096777	1.426401992
10257	ABCC4	909.1024105	2441.396319	1.425191783
3215	HOXB5	99.43858955	266.6943839	1.423309707
11077	HSF2BP	25.17975962	67.52554284	1.423168822
1847	DUSP5	58.26761933	156.2142105	1.422759426
139716	GAB3	5.906103136	15.80416406	1.420026273
91768	CABLES1	1074.730581	2871.411885	1.417785248
390205	LRRC10B	19.68736192	52.57023004	1.416976243
83698	CALN1	114.3159671	304.9240606	1.415423067
1159	CKMT1B	147.0872307	392.1617	1.414776638
339145	FAM92B	28.52402892	76.0371875	1.414527398
4643	MYO1E	36.58943989	97.45471452	1.413304649
120939	TMEM52B	3.508202571	9.33997354	1.412686409
5920	RARRES3	26.84532792	71.41415268	1.411538985
26507	CNNM1	612.3480567	1626.09024	1.408983508
9423	NTN1	343.1341856	910.7788988	1.408328002
152485	ZNF827	9.570613808	25.36333913	1.406061332

153571	C5orf38	24.1265505	63.93461153	1.405975497
347731	LRRTM3	38.00263764	100.6247434	1.404813644
3202	HOXA5	26.47752461	70.09250044	1.40449184
3437	IFIT3	21.08323223	55.77844294	1.403611602
4921	DDR2	271.4152374	716.0876116	1.399634392
7462	LAT2	9.897405895	26.07904283	1.39776857
2346	FOLH1	360.3446363	949.0140973	1.397052147
57596	BEGAIN	238.4840607	627.524303	1.395778488
57699	CPNE5	13.27099554	34.90464662	1.395142505
55107	ANO1	10.29335571	27.06270348	1.394592578
961	CD47	10.28888174	26.99712876	1.39171979
57125	PLXDC1	105.7767098	277.5105371	1.391520546
84460	ZMAT1	90.32840495	236.961713	1.391402337
2257	FGF12	461.6915481	1210.787419	1.390944362
54947	LPCAT2	149.4554152	391.0880915	1.387778436
771	CA12	327.2277672	856.2023882	1.387656685
216	ALDH1A1	10175.26991	26561.99493	1.384296441
151636	DTX3L	233.4286286	609.1343518	1.383778957
4060	LUM	16.01127453	41.69312982	1.380721522
114881	OSBPL7	27.12192366	70.61997861	1.380616877
3669	ISG20	15.65747402	40.74667545	1.379830868
23780	APOL2	181.4874346	470.9725725	1.37577338
51226	COPZ2	16.99427859	44.04930309	1.374070074
8819	SAP30	265.807818	688.2445563	1.372537756
5359	PLSCR1	14.27223405	36.91403502	1.370958266
8029	CUBN	538.1302015	1390.873979	1.369964527
1281	COL3A1	58765.12208	151880.9025	1.369908423
51458	RHCG	4.143332703	10.70084725	1.368861452
83595	SOX7	32.17246504	83.06911857	1.368485767
27129	HSPB7	103.8248025	267.6037407	1.365947155
60680	CELF5	128.7931396	331.8953414	1.365672632
1007	CDH9	6.167857168	15.89398337	1.365639478
124857	WFIKKN2	6.838547667	17.60889312	1.364542354
84465	MEGF11	23.1273988	59.42769585	1.361532434
94032	CAMK2N2	19.98987033	51.25305187	1.358368799
5731	PTGER1	241.0255176	617.5682141	1.357412605
60529	ALX4	551.2823579	1412.143645	1.357023511
7594	ZNF43	34.22146894	87.49752147	1.354340462
56521	DNAJC12	15.94926146	40.76227463	1.353744939
11197	WIF1	4.454028575	11.37815314	1.353083685
58191	CXCL16	301.4251483	768.1862735	1.349656397
3204	HOXA7	244.5729977	622.6319393	1.348114454
6374	CXCL5	5.848168853	14.88730328	1.348025573

23371	TNS2	293.9864936	747.6669095	1.346645807
3707	ITPKB	102.0459272	259.4698448	1.346348277
8701	DNAH11	436.1997015	1108.771106	1.345900879
283008	NUTM2E	61.99736292	157.5766552	1.345775061
84632	AFAP1L2	7.162475251	18.19692899	1.34516484
284086	NEK8	78.0808924	198.2318939	1.344147652
144811	LACC1	6.53710043	16.57211965	1.342035375
2042	EPHA3	2622.237193	6647.115121	1.34193015
283130	SLC25A45	105.4412654	266.7259802	1.338918767
79922	MRM1	79.77664174	201.6802819	1.338031741
83849	SYT15	56.82394131	143.5013956	1.336493962
84168	ANTXR1	10.86160909	27.39249083	1.334542611
64116	SLC39A8	142.6010351	359.5757806	1.334311397
79132	DHX58	76.45597528	192.5961407	1.332877632
375519	GJB7	114.5167946	288.0036647	1.330527975
6337	SCNN1A	8.828324844	22.18824803	1.329584137
286207	CFAP157	9.859387673	24.75104233	1.327919327
1602	DACH1	391.9325148	983.2233806	1.326913958
85439	STON2	5.841096906	14.61975512	1.323607922
100996928	C7orf55-LUC7L2	212.0976158	530.0469286	1.321391693
282973	JAKMIP3	10.19831972	25.46573601	1.320225942
57134	MAN1C1	162.1729566	404.8947426	1.32001365
124936	CYB5D2	184.0393924	459.1569554	1.318972801
2812	GP1BB	177.0917039	441.0031718	1.316292403
1280	COL2A1	12.57498987	31.268253	1.314141378
84152	PPP1R1B	26.05945528	64.72481885	1.312512095
168507	PKD1L1	23.06132075	57.22445001	1.311156552
55930	MYO5C	195.5797707	484.5123818	1.308776378
11219	TREX2	7.141017652	17.62350124	1.303298981
3207	HOXA11	32.47663033	80.04055001	1.30132913
166929	SGMS2	74.49148982	183.5165531	1.300762678
9087	TMSB4Y	33.12048349	81.39179047	1.297159552
59283	CACNG8	180.4284118	443.3003579	1.296857992
57562	CEP126	116.546963	286.1959871	1.296092032
79983	POF1B	3380.69221	8297.46903	1.295352665
5787	PTPRB	75.02165527	183.9848002	1.294207584
1647	GADD45A	9.838335354	24.11343557	1.293351077
2780	GNAT2	6.116478064	14.97944603	1.292211194
288	ANK3	38.62007473	94.57499696	1.292107869
5271	SERPINB8	14.9406351	36.56113428	1.291069353
1956	EGFR	185.2992025	453.4325216	1.291031197
55970	GNG12	170.9325201	418.024441	1.290160399
2537	IFI6	864.1041708	2109.879623	1.28788354

7771	ZNF112	62.52975702	152.6749261	1.287848331
23052	ENDOD1	27.75390901	67.6891836	1.286234334
84660	CCDC62	10.19000606	24.83846043	1.285420843
10566	AKAP3	5.090730316	12.39555813	1.283878688
6421	SFPQ	2447.583494	5956.732908	1.283163197
440686	HIST2H3PS2	22.73180397	55.23696923	1.280921984
9734	HDAC9	4262.065729	10352.19256	1.280311611
7409	VAV1	7.802485651	18.95007798	1.280198081
7205	TRIP6	23.44521315	56.87366621	1.278467411
6352	CCL5	31.4896154	76.38186884	1.278354082
5029	P2RY2	28.73893645	69.66421477	1.277411088
283212	KLHL35	54.38248732	131.819972	1.277354926
857	CAV1	637.4659324	1544.657764	1.27686708
79971	WLS	10.50709767	25.44621696	1.276086974
375057	STUM	45.67760101	110.543718	1.275058254
8839	WISP2	19.32357194	46.74877866	1.274566874
375759	C9orf50	6.19208098	14.96226782	1.272832616
8336	HIST1H2AM	15.56486968	37.57394276	1.271439013
467	ATF3	144.9077716	349.6460045	1.270760048
51435	SCARA3	102.8729096	247.9851822	1.269390804
729920	ISPD	41.22739462	99.35066509	1.268926333
4675	NAP1L3	14.31799337	34.49068133	1.268377311
79899	PRR5L	5.416948673	13.04022538	1.267416478
392490	FLJ44635	5.516814546	13.27170793	1.266446653
84225	ZMYND15	6.783024059	16.30341996	1.265174115
284306	ZNF547	12.91129453	31.02495418	1.264795422
3234	HOXD8	72.35648699	173.4762203	1.261543645
5205	ATP8B1	47.3469909	113.5145784	1.261532947
79815	NIPAL2	88.11056139	211.0373077	1.260111201
1607	DGKB	21.67286627	51.89975654	1.259837806
153572	IRX2	43.67577199	104.5381857	1.259124918
342132	ZNF774	34.565724	82.72123839	1.25891564
10929	SRSF8	57.78349319	137.9889827	1.255823757
7399	USH2A	707.7313597	1680.798658	1.247873163
91608	RASL10B	206.1702192	489.5277937	1.247554819
85301	COL27A1	36.53474123	86.72373724	1.24715794
54898	ELOVL2	25.351545	60.15584668	1.246631291
158866	ZDHHC15	198.516722	471.0522925	1.246626688
128434	VSTM2L	499.5627244	1184.728518	1.245818764
408	ARRB1	1185.482327	2805.277359	1.242669262
25805	BAMBI	41.21617296	97.49427104	1.242106893
84875	PARP10	60.19834392	141.9480743	1.237567574
3090	HIC1	24.68688008	58.17760219	1.236719316

100133941	CD24	157.6751432	371.3836991	1.23595525
64782	AEN	350.4324604	823.7601768	1.233087966
3958	LGALS3	93.2895025	219.2601351	1.232856877
53342	IL17D	53.33609125	125.3475048	1.232749272
6840	SVIL	961.9180024	2259.618796	1.232093583
6752	SSTR2	25.0869598	58.89221501	1.23113929
83445	GSG1	45.62588908	107.0036992	1.229736096
219670	ENKUR	334.2272595	782.8042185	1.227822126
9263	STK17A	251.2651635	588.3955214	1.227575602
2296	FOXC1	2461.959622	5763.859102	1.227227969
222183	SRRM3	291.200214	681.5452891	1.226798113
80217	CFAP43	10.50610904	24.53445137	1.223580548
100505741	SPATA1	5.738421342	13.39247456	1.222696749
8343	HIST1H2BF	10.80694067	25.20445183	1.221720409
494513	PJVK	14.58649383	33.99473838	1.220678324
9469	CHST3	697.1340271	1623.403608	1.219513773
497190	CLEC18B	14.28706692	33.24423364	1.218394353
55089	SLC38A4	279.5572021	650.2579697	1.217868663
151195	CCNYL1	2989.653293	6950.235782	1.217083735
9725	TMEM63A	292.4869392	679.3474878	1.215777502
387104	SOGA3	217.4280374	504.9628211	1.215639182
83982	IFI27L2	322.2481402	748.0037459	1.214873462
23466	CBX6	3766.394844	8741.247604	1.214654954
54505	DHX29	1171.127652	2711.302173	1.211087572
57381	RHOJ	16.55058107	38.27047931	1.209350101
399979	SNX19	205.3896244	474.7561658	1.208823434
7691	ZNF132	11.17142923	25.81576034	1.208438319
7306	TYRP1	718.8019941	1661.047044	1.208426617
53637	S1PR5	686.1028623	1584.152039	1.207214014
83853	ROPN1L	57.11178946	131.7458492	1.205897016
284098	PIGW	138.7387663	319.7343572	1.204502817
114827	FHAD1	11.48598823	26.45762269	1.203808448
79930	DOK3	20.60501392	47.42553968	1.202668752
64582	GPR135	8.50477724	19.56799693	1.202150729
9498	SLC4A8	103.0672431	236.9938493	1.20126373
1806	DPYD	1054.805512	2424.554564	1.200742705
50509	COL5A3	75.62154531	173.782694	1.200415184
161394	SAMD15	15.54177162	35.67548892	1.198782234
100288332	NPIPA5	427.4525288	978.4070534	1.194670596
6441	SFTPD	20.97244919	47.99413706	1.194362827
374882	TMEM205	908.8604053	2078.543613	1.193442391
348801	LNP1	32.13408229	73.43992396	1.192460294
155185	AMZ1	10.16924426	23.22230179	1.191298511

137872	ADHFE1	7.876600042	17.93604143	1.18721659
221	ALDH3B1	302.6712765	687.9764681	1.184607449
51481	VCX3A	13.92007533	31.58253494	1.181959954
60677	CELF6	15.97401046	36.23409507	1.1816213
440689	HIST2H2BF	9.509005641	21.56573634	1.181374585
9542	NRG2	189.8804402	430.5977406	1.18124945
112849	L3HYPDH	117.7092247	266.8242711	1.180662518
54855	FAM46C	20.00615263	45.34707223	1.180565658
84675	TRIM55	19.93355598	45.1141537	1.178381024
144100	PLEKHA7	77.3537885	175.0240824	1.178009586
154214	RNF217	70.66147946	159.7956023	1.177231844
126820	WDR63	8.804734831	19.89131125	1.175786922
29952	DPP7	18.55070073	41.8878453	1.175057991
8501	SLC43A1	9.13571322	20.61723355	1.174261494
5621	PRNP	519.7564908	1172.588874	1.173789499
285600	KIAA0825	17.26622523	38.94542731	1.173501234
84631	SLITRK2	316.4902546	713.8487151	1.173457281
55106	SLFN12	33.45652657	75.46002046	1.173424818
130752	MDH1B	15.5308916	35.02911513	1.17341389
729288	ZNF286B	29.72799854	67.02329701	1.172840322
160857	CCDC122	60.81750232	136.977502	1.171380483
5175	PECAM1	6.394453464	14.39508434	1.17068328
100528064	NEDD8-MDP1	80.54396417	181.1612092	1.169425689
4493	MT1E	108.2790095	243.5110302	1.169233527
57540	DISP3	80.3487765	180.6840326	1.169121058
5753	PTK6	74.02694883	166.3602059	1.168187904
161357	MDGA2	22.58736661	50.75367871	1.167996314
133383	SETD9	112.3065102	252.0879738	1.166485734
101928601	MEI4	10.7969352	24.22873479	1.16609722
4318	MMP9	38.24859823	85.77461078	1.165143799
8741	TNFSF13	19.31015485	43.18818874	1.161277079
141	ADPRH	208.5898162	466.1600357	1.160156603
401494	HACD4	13.48821224	30.14341494	1.160143726
4862	NPAS2	9.507596539	21.23525039	1.159308532
150082	LCA5L	13.48344267	30.09948931	1.158550109
56112	PCDHGA3	527.8610518	1177.783662	1.157844441
4995	OR3A2	33.16799732	73.97853336	1.157314794
7582	ZNF33B	108.4674276	241.8426704	1.156806943
2641	GCG	201.2789311	448.6507942	1.156396799
84698	CAPS2	87.88827341	195.7583065	1.155330936
4647	MYO7A	120.2261158	266.9003152	1.150550693
1583	CYP11A1	12.89586684	28.62075177	1.150152815
79800	CARF	41.17188361	91.36621566	1.150001347

3770	KCNJ14	11.50117218	25.51337586	1.1494729
5327	PLAT	397.3142242	881.1717148	1.149142742
255374	MBLAC1	43.87956839	97.22405035	1.147763903
169611	OLFML2A	1019.531322	2256.697718	1.146307085
8407	TAGLN2	1126.213715	2492.161543	1.145916962
5224	PGAM2	37.12428506	82.14046979	1.145729956
10892	MALT1	247.4555886	547.0756003	1.144570588
11146	GLMN	286.3791413	632.1543873	1.142350526
7957	EPM2A	36.79155661	81.20327098	1.142163126
5971	RELB	173.4915553	382.8571359	1.141940706
1311	COMP	122.6833115	270.1552015	1.138849445
11103	KRR1	713.6155899	1569.469066	1.137057556
1415	CRYBB2	18.58798554	40.86493931	1.136493164
101059918	GOLGA8R	50.83202046	111.7398254	1.136333991
124056	NOXO1	10.43399509	22.93612906	1.136330267
55117	SLC6A15	1644.347508	3614.516988	1.136287648
6282	S100A11	1144.662422	2514.775825	1.135507611
10325	RRAGB	111.7215861	245.4177935	1.135331892
5332	PLCB4	164.0565929	360.1829856	1.134536461
79642	ARSJ	548.9904746	1204.138788	1.133148663
401027	C2orf66	27.11677981	59.43264278	1.13206967
159371	SLC35G1	93.80886061	205.1996544	1.129232199
116443	GRIN3A	9.432093136	20.60454927	1.127313035
683	BST1	44.86739162	97.91726009	1.125895875
28951	TRIB2	317.2996744	692.4182196	1.125797646
51065	RPS27L	312.7518892	682.0548294	1.124869121
1050	CEBPA	97.86010641	213.0837741	1.122627982
57727	NCOA5	845.99601	1841.24482	1.121958702
51375	SNX7	1051.096904	2287.589085	1.121932244
54482	TRMT13	200.1977428	435.5263188	1.121334195
100125288	ZGLP1	7.390396269	16.06431986	1.120136273
11182	SLC2A6	172.4501163	374.4290858	1.118513407
9289	ADGRG1	216.7816938	470.2954416	1.117324416
3908	LAMA2	4339.871258	9408.815545	1.116360871
7041	TGFB1I1	613.5644344	1329.172753	1.115241861
286097	MICU3	49.94341951	108.0673116	1.113563692
79884	MAP9	224.0972821	484.6274124	1.11275086
8764	TNFRSF14	13.46964003	29.12302207	1.112448774
388323	GLTPD2	18.91275537	40.86404477	1.111472448
55500	ETNK1	504.9349989	1089.829497	1.10993286
54839	LRRC49	37.80736616	81.50352781	1.108195159
143941	TTC36	16.53806662	35.65064761	1.108137698
166614	DCLK2	65.80068621	141.6494512	1.106150478

8969	HIST1H2AG	87.48869307	188.1676682	1.104850277
148932	MOB3C	299.5720725	643.8936376	1.103919259
92154	MTSS1L	463.8410449	996.1000376	1.10266015
400954	EML6	23.02942267	49.41848391	1.101572507
56937	PMEPA1	13.21744973	28.3554048	1.101179913
60598	KCNK15	120.2554678	257.671187	1.099428731
56137	PCDHA12	76.76346389	164.395619	1.098680134
1466	CSRP2	119.842712	256.6348251	1.098574779
4502	MT2A	168.2537582	359.1235487	1.093841528
1767	DNAH5	70.90921689	151.3054506	1.093418891
219348	PLAC9	10.75525299	22.93404089	1.092449113
3198	HOXA1	57.42447242	122.3896533	1.091743998
6604	SMARCD3	959.3322615	2044.261243	1.091477095
2159	F10	33.38706714	71.08311195	1.090217476
2906	GRIN2D	488.3433868	1039.284524	1.089622809
388677	NOTCH2NL	257.6753423	548.2653055	1.089319694
55924	FAM212B	57.47142395	122.2530978	1.088954322
50944	SHANK1	127.5596772	270.1992214	1.082851165
284307	ZIK1	200.5570693	424.7295065	1.082531518
91807	MYLK3	192.7104547	407.7336831	1.081192302
6398	SECTM1	140.0138192	295.8324406	1.079211038
2113	ETS1	916.8531534	1934.464359	1.077171558
340526	RTL5	717.5176083	1512.469869	1.07582026
954	ENTPD2	643.0098894	1355.101162	1.075487726
817	CAMK2D	1804.752587	3803.309311	1.075454201
116835	HSPA12B	30.71713696	64.72425991	1.07526281
10039	PARP3	139.4566535	293.3419478	1.072766624
100293516	ZNF587B	34.06113452	71.6379328	1.072597218
81831	NETO2	1154.740334	2428.112701	1.072266916
5805	PTS	135.0514386	283.8651011	1.071696484
23362	PSD3	865.4832741	1818.125816	1.070874195
65983	GRAMD2B	125.199118	262.8041294	1.069763545
5721	PSME2	1183.090534	2483.252701	1.069670603
389432	SAMD5	86.00711801	180.3955818	1.068636037
85369	STRIP1	806.4563007	1690.855171	1.068084826
341019	DCDC1	36.40576617	76.29619574	1.067444152
3675	ITGA3	531.5994336	1111.850233	1.064550996
93183	PIGM	171.7740106	359.1506917	1.064077521
2149	F2R	400.0985768	836.3777463	1.063799179
8814	CDKL1	60.49512369	126.4499402	1.063675593
110599564	EEF1AKMT4	43.95190218	91.79445436	1.062481393
389799	CFAP77	11.76974021	24.56686667	1.061631387
4054	LTBP3	1689.692229	3521.9486	1.059613365

3572	IL6ST	3269.895051	6815.105263	1.059491608
115399	LRRC56	20.56355156	42.84561722	1.05905818
54487	DGCR8	324.6614375	675.7547472	1.057563707
7993	UBXN8	117.7347687	244.5542184	1.05461392
6041	RNASEL	13.78076711	28.56100258	1.051390425
6890	TAP1	474.1782937	981.8951141	1.050139302
23213	SULF1	664.7311446	1375.711369	1.049334962
4234	METTL1	163.3422029	337.0838931	1.045210103
4636	MYL5	26.65895437	54.94889485	1.04347027
5272	SERPINB9	442.2104432	911.1586095	1.042969116
79966	SCD5	681.0550656	1403.155763	1.042831816
388407	C17orf82	38.81600025	79.90998202	1.041724265
145946	SPATA8	13.07094068	26.90665089	1.041599857
283948	NHLRC4	37.40879855	76.99416692	1.041371519
10553	HTATIP2	117.3840334	241.5932586	1.041344012
284695	ZNF326	315.2440739	648.419044	1.040457213
4313	MMP2	90.81093428	186.6805078	1.039633373
112817	HOGA1	12.14369272	24.92696981	1.037500324
169834	ZNF883	26.97951075	55.35919416	1.036958756
11230	PRAF2	357.7090765	733.7440691	1.036490211
55859	BEX1	22.9445362	47.01453131	1.034956091
100037417	DDTL	72.17584933	147.8750826	1.03479089
80258	EFHC2	20.1774856	41.33749999	1.034704734
343099	CCDC18	126.8448268	259.8357334	1.034535166
163154	PRR22	22.23038116	45.49957965	1.033320532
84696	ABHD1	39.07944236	79.96430438	1.032946252
9147	NEMF	979.3260405	2003.747398	1.032839495
131583	FAM43A	14.76582983	30.17867039	1.031266807
79745	CLIP4	163.279187	333.6078905	1.030812507
85452	CFAP74	61.46150001	125.5065925	1.030008265
284338	PRR19	39.76801482	81.20426742	1.029946999
85236	HIST1H2BK	2254.318094	4602.543919	1.029740388
221711	SYCP2L	15.76980638	32.1448782	1.027423937
26095	PTPN20	112.0197073	228.2883518	1.027104686
1153	CIRBP	1074.64207	2189.1725	1.026529416
3196	TLX2	206.9324834	421.4886541	1.026333665
8082	SSPN	59.7179848	121.4660754	1.024316049
10587	TXNRD2	524.848949	1067.461619	1.024210016
79148	MMP28	16.45469555	33.41528312	1.022008764
23732	FRRS1L	308.7033731	626.5706322	1.021255904
8370	HIST2H4A	24.22110246	49.15483842	1.021068898
554313	HIST2H4B	24.22110246	49.15483842	1.021068898
644	BLVRA	393.388886	797.8100976	1.020089184

84190	METTL25	98.86407589	199.9064606	1.015806807
363	AQP6	19.14734015	38.69393665	1.014963519
10610	ST6GALNAC2	200.5297132	405.0920637	1.014433799
4482	MSRA	31.11367263	62.83380048	1.013992144
55783	CMTR2	164.9670208	332.52293	1.01127619
81606	LBH	28.27942225	56.96583614	1.010344312
51776	MAP3K20	423.528665	852.1921345	1.008719119
112937	GLB1L3	16.78770231	33.75945813	1.007886962
25876	SPEF1	14.80827852	29.76988597	1.007449761
10384	BTN3A3	120.0554599	240.9424884	1.004987809
10763	NES	2736.356541	5489.505151	1.004419882
54865	GPATCH4	274.7594204	551.1782809	1.004350094
123099	DEGS2	191.7815566	384.448353	1.003325814
115416	MALSU1	939.2367328	1878.714053	1.000184763
4884	NPTX1	857.7273489	1715.478896	1.00002035
89832	CHRFAM7A	579.3083463	289.3496934	-1.001517335
367	AR	271.5986326	135.634776	-1.001749092
133418	EMB	1551.471808	774.7572286	-1.001821266
1643	DDB2	1963.120626	980.1470023	-1.002078778
8434	RECK	740.3977132	369.4159211	-1.003054394
84952	CGNL1	849.2388014	423.2250405	-1.004745301
644596	SMIM10L2B	78.92361661	39.28999795	-1.006294975
10886	NPFFR2	152.1451878	75.57178182	-1.009529159
11123	RCAN3	935.8757505	464.8064414	-1.009686944
90625	ERVH48-1	28.31987159	14.0574952	-1.01047517
127833	SYT2	103.6257884	51.42408146	-1.010867054
27255	CNTN6	1226.440218	608.4627844	-1.011235982
65249	ZSWIM4	1135.071885	563.0304244	-1.011498879
9917	FAM20B	2118.881406	1049.840052	-1.013133299
18	ABAT	3099.681975	1535.174786	-1.013717282
6303	SAT1	1982.864372	981.479134	-1.014556499
93099	DMKN	147.5051576	73.00346944	-1.014728466
284273	ZADH2	213.4781432	105.6428394	-1.014893385
79958	DENND1C	166.0146856	82.12586956	-1.015402222
4440	MSI1	1720.0772	849.3216915	-1.018090315
3911	LAMA5	9234.709293	4558.77455	-1.018420482
80816	ASXL3	249.1963962	122.921699	-1.019543592
1013	CDH15	76.318485	37.60908401	-1.020951364
83463	MXD3	214.7910989	105.8378096	-1.021079099
6496	SIX3	9516.211256	4687.13206	-1.021681856
89122	TRIM4	269.2013653	132.376746	-1.024036014
2770	GNAI1	96.4533844	47.42895442	-1.024063797
6606	SMN1	939.3608344	461.9038889	-1.024086751

80817	CEP44	1984.411965	975.6823577	-1.024228114
9306	SOCS6	1496.21219	735.5928086	-1.024335509
2125	EVPL	125.1975944	61.52338172	-1.025000132
29097	CNIH4	1854.016937	910.2519829	-1.02631654
54814	QPCTL	1311.766481	643.567471	-1.027347604
79990	PLEKHH3	1478.281132	725.0674411	-1.027733563
55274	PHF10	356.8273539	174.8932183	-1.028751867
55366	LGR4	4692.305499	2298.806694	-1.029411789
342346	C16orf96	54.04399644	26.47633215	-1.029431088
7545	ZIC1	1340.049651	656.3800649	-1.029683127
27293	SMPDL3B	573.3702868	280.5968748	-1.030968203
100129543	ZNF730	134.5834009	65.85797696	-1.031070383
92610	TIFA	74.93865243	36.67047849	-1.031090944
481	ATP1B1	141.5155596	69.09082853	-1.034394567
151306	GPBAR1	41.55916056	20.2642816	-1.036227484
113451	AZIN2	50.31215391	24.52450557	-1.036682903
7903	ST8SIA4	45.61427022	22.22136605	-1.037537727
9444	QKI	4394.174285	2140.215927	-1.037835735
4800	NFYA	1372.582259	668.125663	-1.038701233
55435	AP1AR	518.3826517	252.2918538	-1.038923813
57716	PRX	2605.337808	1265.406016	-1.041870085
89846	FGD3	398.3527055	193.2143069	-1.043844448
285761	DCBLD1	86.37928207	41.88779505	-1.044155382
4324	MMP15	2505.93792	1212.211903	-1.047708761
1005	CDH7	3382.640738	1635.754956	-1.048193319
348262	MCRIP1	2533.835122	1224.963742	-1.048583604
347688	TUBB8	143.6974597	69.45266737	-1.04893255
3157	HMGCS1	5770.761574	2789.097109	-1.04896356
9620	CELSR1	7016.559233	3386.887994	-1.050803461
55259	CASC1	25.02531982	12.06111008	-1.053025811
83999	KREMEN1	1209.35734	582.4750196	-1.05397251
375387	NRROS	258.098419	124.2657951	-1.054492064
80237	ELL3	189.2877749	91.07429873	-1.055465351
4948	OCA2	125.1867814	60.20692068	-1.056080997
1718	DHCR24	2253.735295	1083.426562	-1.056716712
646643	SBK2	40.16492119	19.30523868	-1.056943655
283248	RCOR2	1399.185713	672.4995616	-1.056982231
79816	TLE6	681.0308107	326.874477	-1.058983336
10420	TESK2	856.9232855	411.2183979	-1.059261243
7163	TPD52	4330.840445	2077.285515	-1.059947499
196394	AMN1	148.1044641	71.02940868	-1.060126746
6938	TCF12	3178.831318	1524.39884	-1.060256048
23025	UNC13A	593.1926397	283.9847612	-1.062687181

3038	HAS3	1113.337757	532.5455189	-1.063914585
256933	NPB	41.11808999	19.6451843	-1.065597548
7468	NSD2	3185.460001	1519.159514	-1.068228359
5884	RAD17	1126.893498	537.2138311	-1.068782819
55197	RPRD1A	1626.713795	775.0909214	-1.069522985
4605	MYBL2	5242.890063	2497.571766	-1.069836161
158326	FREM1	19.90736902	9.478707126	-1.070540368
7008	TEF	686.3505079	326.5990035	-1.071425136
5865	RAB3B	896.5452049	424.9933414	-1.07693609
57519	STARD9	314.2510856	148.8208603	-1.078340965
131616	TMEM42	217.9460851	103.205596	-1.078450091
401	PHOX2A	42.85064028	20.28516475	-1.078891742
643314	KIAA0754	133.6778174	63.25994342	-1.079395912
11156	PTP4A3	1860.993358	879.8330926	-1.080771136
10793	ZNF273	357.1233125	168.7645936	-1.081410053
28984	RGCC	261.8975735	123.7092881	-1.082048871
348751	FTCDNL1	36.80902331	17.36431568	-1.083933914
9783	RIMS3	1169.091569	551.4377355	-1.084118032
284254	DYNAP	36.15197351	17.02259076	-1.086623779
5657	PRTN3	621.3678561	292.5393605	-1.086816863
54852	PAQR5	68.1386623	32.07402011	-1.087068434
4438	MSH4	20.21001366	9.507382899	-1.087950127
58526	MID1IP1	676.9352281	318.3814369	-1.088261575
84614	ZBTB37	50.91537118	23.93567954	-1.088938502
1373	CPS1	9325.353772	4381.752831	-1.089650351
728498	GOLGA8H	255.5030905	119.942132	-1.091002219
3600	IL15	465.246628	218.1281713	-1.092819588
158038	LINGO2	351.0548462	164.3803994	-1.094658161
5106	PCK2	1390.262239	650.9828639	-1.094665565
222643	UNC5CL	59.63646877	27.91454438	-1.095177826
9414	TJP2	5427.928426	2539.40247	-1.095912631
152503	SH3D19	1364.734638	638.0758246	-1.096820678
9467	SH3BP5	334.5499814	156.2726122	-1.098156805
55876	GSDMB	28.01688428	13.07507939	-1.099476818
10493	VAT1	7861.261265	3665.384633	-1.1007962
8553	BHLHE40	1050.141643	488.7097869	-1.10353403
160622	GRASP	235.7676984	109.6926389	-1.10389936
285533	RNF175	18.26152588	8.487061156	-1.105470339
8673	VAMP8	31.94872566	14.81934845	-1.108276368
6658	SOX3	12289.64766	5695.586231	-1.109527307
8905	AP1S2	696.8892596	322.9383199	-1.109670779
55814	BDP1	1530.549522	707.3525836	-1.113548307
2201	FBN2	16.24140089	7.504502035	-1.113847827

9920	KBTBD11	81.63793313	37.71555851	-1.114079867
8702	B4GALT4	752.5458272	346.6161132	-1.118440716
29944	PNMA3	77.54661611	35.70875391	-1.118786037
144195	SLC2A14	178.0794647	81.97161383	-1.119324854
8622	PDE8B	40.52866978	18.64917714	-1.119830849
10620	ARID3B	2365.208452	1086.259956	-1.122597938
79629	OCEL1	944.9710239	433.7030552	-1.123562486
374907	B3GNT8	24.97036514	11.45689894	-1.124000318
27165	GLS2	211.5022864	97.00388951	-1.124558759
2202	EFEMP1	230.3702854	105.5759418	-1.125673524
6721	SREBF2	8576.400381	3925.326896	-1.127559454
3064	HTT	2582.482431	1181.76469	-1.127815737
57333	RCN3	1266.890123	579.5357019	-1.12832196
1271	CNTFR	1018.411547	465.6991161	-1.128850634
900	CCNG1	3174.014142	1449.618605	-1.13063518
2590	GALNT2	5275.223974	2405.148818	-1.133106185
90355	C5orf30	283.8760916	129.3766735	-1.133683824
23235	SIK2	3640.043007	1656.209418	-1.136070391
338761	C1QL4	1717.789778	781.3519694	-1.136509012
6909	TBX2	702.7443994	319.4518591	-1.137401515
3321	IGSF3	208.0704931	94.35477632	-1.140904933
5309	PITX3	65.80554702	29.81943847	-1.141956109
85407	NKD1	65.36054006	29.61700747	-1.141994027
57167	SALL4	3182.617902	1441.588162	-1.142554891
226	ALDOA	44471.53037	20115.61592	-1.14456614
3673	ITGA2	23.97323487	10.80536678	-1.149676551
1307	COL16A1	183.1648868	82.5556965	-1.149703289
8565	YARS	3474.698621	1566.031699	-1.149774435
222389	BEND7	22.57457822	10.17424864	-1.149776776
145501	ISM2	32.06198828	14.44024009	-1.150769165
54836	BSPRY	17.54109908	7.894940574	-1.151738834
3998	LMAN1	5047.899154	2263.166632	-1.157340278
79144	PPDPF	27982.27723	12541.60036	-1.157791922
8310	ACOX3	159.5806342	71.46643574	-1.158947842
11217	AKAP2	2929.356427	1311.214508	-1.15968002
51778	MYOZ2	236.076108	105.5718683	-1.16102659
7456	WIPF1	678.7920517	303.2207668	-1.162601108
51673	TPPP3	21.25613993	9.495088445	-1.162626286
339456	TMEM52	281.0485319	125.5263842	-1.162828644
3159	HMGA1	9453.167563	4218.891839	-1.163933727
1628	DBP	2035.724396	908.3690291	-1.164191834
83881	MIXL1	91.61893272	40.65036642	-1.1723774
5783	PTPN13	1056.59034	468.6206435	-1.172923711

23216	TBC1D1	398.6825615	176.5775138	-1.174938867
94031	HTRA3	325.0509001	143.9207016	-1.175391524
26261	FBXO24	85.29484559	37.72697249	-1.17686223
57715	SEMA4G	918.3274202	405.6582267	-1.178743877
221303	FAM162B	58.00186716	25.59909684	-1.180006432
5420	PODXL	297.0788055	131.047896	-1.180751492
54800	KLHL24	2140.559961	942.5447329	-1.183355254
357	SHROOM2	195.4888553	85.91938155	-1.186030849
2824	GPM6B	17.19531463	7.550894121	-1.187296121
132720	FAM241A	38.1007708	16.71725222	-1.18848245
340485	ACER2	47.86637767	20.9967343	-1.188847675
6619	SNAPC3	1361.203283	596.1948991	-1.191026599
57393	CLTRN	42.09562128	18.43635188	-1.191116965
654254	ZNF732	41.42663076	18.12824256	-1.19231942
79792	GSDMD	51.22587393	22.38484336	-1.194350469
51092	SIDT2	4774.408996	2085.643514	-1.194829571
79925	SPEF2	69.13840422	30.19472077	-1.195190974
115362	GBP5	754.3034756	329.4152517	-1.195237721
9622	KLK4	16.53111113	7.21901865	-1.195309062
5651	TMPRSS15	18.8687055	8.232362919	-1.19661696
3753	KCNE1	50.14377823	21.86791687	-1.197254908
8864	PER2	197.5061814	86.06662616	-1.198371985
10683	DLL3	2656.320528	1156.952551	-1.199099544
3373	HYAL1	111.6298561	48.48986607	-1.202967763
10000	AKT3	554.9958045	240.5911972	-1.205893008
154091	SLC2A12	55.25148269	23.94024259	-1.206575409
1122	CHML	771.6522249	333.6701958	-1.20952796
83854	ANGPTL6	60.0306989	25.91659548	-1.211824253
2966	GTF2H2	1118.646927	482.0220965	-1.21458357
1145	CHRNE	63.99997953	27.5693869	-1.215004259
9509	ADAMTS2	168.9960511	72.76465538	-1.215679783
115330	GPR146	70.12577984	30.18465412	-1.21612964
3781	KCNN2	464.6674826	199.8661876	-1.217164264
401720	FIGNL2	948.0669668	407.7804614	-1.217196317
57462	MYORG	957.6128318	411.6407251	-1.218056764
347735	SERINC2	510.813311	219.4613189	-1.21882944
115727	RASGRP4	393.9332891	169.0807419	-1.220238989
971	CD72	16.1810394	6.944401	-1.22038212
5757	PTMA	47579.16071	20386.79869	-1.222694574
55344	PLCXD1	10922.04263	4677.403682	-1.223462842
5837	PYGM	1624.134591	695.4102424	-1.223734972
8626	TP63	1792.573434	767.2475239	-1.224268232
10675	CSPG5	1009.995003	431.8835999	-1.225633716

2065	ERBB3	334.4546873	142.8567092	-1.227241971
4664	NAB1	185.8135169	79.31107904	-1.228261137
114793	FMNL2	1229.988968	524.0596828	-1.230842348
4601	MXI1	1215.021468	517.6156382	-1.231028695
5144	PDE4D	762.814234	324.8447876	-1.231581208
93	ACVR2B	1116.806802	475.2848302	-1.232515373
4615	MYD88	74.13271707	31.54225953	-1.232824378
285489	DOK7	53.5403842	22.73906977	-1.235454255
283659	PRTG	3233.031721	1372.704875	-1.235866176
22936	ELL2	479.6013885	203.602191	-1.236082749
158219	TTC39B	88.96912542	37.74963841	-1.236841945
79412	KREMEN2	2916.744503	1235.122397	-1.239705
1000	CDH2	940.4780579	398.1409322	-1.240115085
8490	RGS5	5732.400111	2424.266188	-1.241591193
65124	SOWAHC	1590.662072	672.1861592	-1.242694633
10110	SGK2	14.13055418	5.964839034	-1.244262936
79041	TMEM38A	626.0702055	264.2090762	-1.244644418
7378	UPP1	5492.776111	2317.719086	-1.24482977
4638	MYLK	129.8187867	54.75072192	-1.245549286
23038	WDTC1	3690.745863	1554.705156	-1.247271395
85442	KNDC1	192.5563328	81.02734326	-1.248799828
3730	ANOS1	110.9377451	46.64603354	-1.249923993
54587	MXRA8	250.0632659	105.0327611	-1.251453748
3613	IMPA2	1118.876751	469.2115469	-1.253740704
10218	ANGPTL7	14.16343648	5.937278065	-1.254297763
346689	KLRG2	134.5419021	56.39060719	-1.254528777
4323	MMP14	54.9240034	23.0043216	-1.255531875
1021	CDK6	982.8585634	411.6075733	-1.255714296
23150	FRMD4B	240.1778552	100.5224244	-1.256585767
51361	HOOK1	161.7077457	67.64326059	-1.257370676
6445	SGCG	73.83558238	30.86304377	-1.258435888
340371	NRBP2	2218.16722	925.0361649	-1.261786454
7855	FZD5	6484.321819	2693.918487	-1.267249496
604	BCL6	118.5612825	49.23031876	-1.268013972
3164	NR4A1	1670.289511	693.4745129	-1.268183421
55753	OGDHL	747.9922437	309.8526244	-1.271441123
3156	HMGCR	1751.684791	725.2260227	-1.272240593
220001	VWCE	44.4955939	18.40018821	-1.27394196
83543	AIF1L	540.8863245	223.2560887	-1.2766259
389125	MUSTN1	15.16095291	6.257230004	-1.276764393
57685	CACHD1	137.2138302	56.43272561	-1.281821966
8728	ADAM19	51.41686934	21.12913708	-1.283007922
8749	ADAM18	539.942662	220.9708808	-1.288949946

81618	ITM2C	6549.566479	2674.311744	-1.292231767
11248	NXPH3	100.4952122	41.02468032	-1.292562774
9717	SEC14L5	115.316902	46.97448901	-1.295654611
10859	LILRB1	11.43436733	4.654549058	-1.296663234
55314	TMEM144	689.075091	280.3133721	-1.297620638
1028	CDKN1C	470.4404165	191.2618219	-1.298463087
126668	TDRD10	34.03523786	13.82427473	-1.299825398
158405	KIAA1958	708.8368393	287.8681693	-1.300045311
1649	DDIT3	389.1030136	157.7052955	-1.30292105
317649	EIF4E3	11644.42269	4717.615379	-1.303509408
205860	TRIML2	17.36021517	7.031783425	-1.303822287
9456	HOMER1	592.3761466	239.5929772	-1.305927927
7697	ZNF138	410.3430546	165.9049856	-1.306473293
6405	SEMA3F	438.6458635	177.2441455	-1.307318689
203447	NRK	14.80013394	5.967317119	-1.310455879
3050	HBZ	16.47626854	6.640078352	-1.311117375
26140	TTLL3	73.85386646	29.7603231	-1.311283263
1132	CHRM4	319.859101	128.1846354	-1.319213186
7101	NR2E1	4494.41358	1798.226858	-1.321557851
55636	CHD7	2115.259016	843.3021572	-1.326712784
730098	LOC730098	99.02174869	39.458232	-1.327419112
25816	TNFAIP8	187.5483619	74.583667	-1.330331026
131405	TRIM71	733.4637124	291.2780058	-1.332328822
3398	ID2	5758.139595	2285.816425	-1.33289322
10087	COL4A3BP	1663.786116	659.9171865	-1.334113087
5864	RAB3A	324.8961751	128.519847	-1.33798759
3685	ITGAV	38364.57678	15173.5389	-1.338217238
344758	GPR149	16.80941983	6.633976599	-1.341324104
5230	PGK1	45998.02218	18132.15162	-1.3430217
4094	MAF	55.25317152	21.7325597	-1.346199169
7373	COL14A1	364.5173271	143.3645068	-1.346299499
1112	FOXN3	1370.644119	538.7924532	-1.347052483
2069	EREG	11.05545711	4.34193249	-1.348349477
64946	CENPH	733.6236339	287.8989945	-1.349477366
10434	LYPLA1	6541.447084	2567.038491	-1.349504893
1832	DSP	752.2672188	295.0965017	-1.350058406
1272	CNTN1	16.13733579	6.317855111	-1.352895658
84628	NTNG2	69.754714	27.29921061	-1.353431482
7179	TPTE	829.1628637	323.6008404	-1.357440148
282809	POC1B	1187.067232	463.142734	-1.357872863
22902	RUFY3	1689.647222	658.730383	-1.358962061
116173	CMTM5	11.58375932	4.494192857	-1.365969592
51554	ACKR4	27.2442062	10.55865692	-1.367523123

783	CACNB2	28.22085009	10.93615344	-1.367656057
4661	MYT1	231.2411117	89.56686311	-1.368360928
134121	C5orf49	18.84107676	7.290096355	-1.369871629
6662	SOX9	92.62726623	35.82140427	-1.37061504
56605	ERO1B	150.5561578	58.15967657	-1.372210564
84034	EMILIN2	1954.609977	754.9334796	-1.372459328
284422	SMIM24	96.38487606	37.15362525	-1.3753038
55843	ARHGAP15	2276.491601	877.2930745	-1.375681351
26355	FAM162A	1649.9694	635.6949781	-1.376032672
431707	LHX8	12.09166315	4.658474672	-1.37608314
335	APOA1	59.2225891	22.81043224	-1.376453779
3060	HCRT	12.32165417	4.73718745	-1.379093284
146540	ZNF785	59.92328484	23.02895551	-1.379667733
388588	SMIM1	238.2421918	91.54812291	-1.379826721
7634	ZNF80	847.6638078	325.2799667	-1.381810218
25914	RTTN	1069.074593	410.1579011	-1.382111193
55093	WDYHV1	230.9444374	88.25809131	-1.387745345
56154	TEX15	278.8794246	106.5511831	-1.388094886
230	ALDOC	508.456244	194.2196728	-1.388434285
79924	ADM2	171.592656	65.3611414	-1.392482725
375033	PEAR1	15.77261075	6.000887993	-1.394173574
388581	C1QTNF12	54.07531845	20.56037538	-1.395103651
2245	FGD1	8790.83188	3340.498704	-1.395936195
1382	CRABP2	30551.1575	11604.88548	-1.396494755
55203	LGI2	112.544545	42.73170621	-1.397117304
81563	C1orf21	2970.958876	1126.282954	-1.399359317
64283	ARHGEF28	16.74987046	6.340370878	-1.4015108
54532	USP53	4039.753737	1528.959945	-1.401716737
23090	ZNF423	177.1564561	67.03158255	-1.402111142
8577	TMEFF1	169.4942884	64.11542668	-1.402493231
11045	UPK1A	1116.61518	421.3246567	-1.406127822
54361	WNT4	31.60416849	11.91566405	-1.407255504
2911	GRM1	854.693335	322.0164014	-1.408272701
120071	LARGE2	402.5520515	151.5971859	-1.408932368
9060	PAPSS2	719.2477062	270.4646553	-1.411048642
6515	SLC2A3	7758.013662	2909.841975	-1.41474651
9262	STK17B	856.694247	321.3009648	-1.414855088
6866	TAC3	17.79367897	6.656553217	-1.418517585
27134	TJP3	903.6386494	337.6621755	-1.420165398
54845	ESRP1	107.4454719	40.14530644	-1.420301453
53616	ADAM22	195.1511263	72.88403411	-1.42091707
51168	MYO15A	41.66359343	15.55520107	-1.421390232
1821	DRP2	44.40092398	16.53947346	-1.424676392

10507	SEMA4D	610.6220539	227.3118051	-1.425607086
148229	ATP8B3	97.89494957	36.42624649	-1.426256091
80303	EFHD1	432.2097006	160.4088123	-1.429978052
5137	PDE1C	18.81512799	6.979321354	-1.430734438
2118	ETV4	1983.173863	735.4261099	-1.43115886
5172	SLC26A4	41.02513395	15.21274101	-1.431227925
79956	ERMP1	785.4205128	291.0241022	-1.432326637
2695	GIP	136.4781035	50.56869481	-1.432353056
4237	MFAP2	384.514821	142.1367733	-1.435759353
348738	C2orf48	12.71805231	4.691081949	-1.438885139
22843	PPM1E	357.2903003	131.5888076	-1.441059964
114800	CCDC85A	8.346058668	3.073594234	-1.441168344
9497	SLC4A7	369.085497	135.6165696	-1.444421591
23550	PSD4	299.7426565	110.0125682	-1.446056058
643246	MAP1LC3B2	63.22653896	23.13853462	-1.450232748
84561	SLC12A8	29.91095119	10.94522125	-1.450372672
100533952	RBAK-RBAKDN	77.80960848	28.34710056	-1.456747142
22875	ENPP4	115.0842729	41.92293482	-1.456879071
8313	AXIN2	244.4122375	88.97558147	-1.45783516
402778	IFITM10	97.67855109	35.54427763	-1.458424485
4008	LMO7	24.81947361	9.030909481	-1.458529328
7710	ZNF154	93.28717565	33.93671922	-1.458831664
7098	TLR3	260.4834323	94.70140394	-1.459733896
6340	SCNN1G	1966.826995	714.501623	-1.460860868
23414	ZFPM2	965.5730486	350.1113784	-1.463571458
23671	TMEFF2	39.36267257	14.25660516	-1.465197695
140738	TMEM37	64.66521089	23.38979929	-1.467110286
83401	ELOVL3	40.32457325	14.55555071	-1.470089841
81848	SPRY4	2272.074594	820.0014729	-1.470311794
23338	JADE2	4802.540815	1732.305232	-1.47110472
5214	PFKP	1865.203584	668.0714573	-1.48125878
401024	FSIP2	34.29978571	12.28031539	-1.48185195
9749	PHACTR2	457.0999105	163.4884488	-1.48332083
6331	SCN5A	106.0342759	37.89422966	-1.484480611
130888	FBXO36	318.4260729	113.5537317	-1.487583352
7137	TNNI3	25.4472442	9.073854636	-1.487721976
126393	HSPB6	37.30869528	13.30010475	-1.4880743
5163	PDK1	1768.37754	629.5357047	-1.490066207
5456	POU3F4	13.36805135	4.755241158	-1.491198768
51027	BOLA1	83.45615738	29.67362005	-1.491837555
2264	FGFR4	753.8760142	267.2380154	-1.496202021
27443	CECR2	2349.691464	832.8311973	-1.496375313
54106	TLR9	35.19320055	12.43867116	-1.500464354

8497	PPFIA4	392.7849288	138.8062275	-1.500667279
440	ASNS	2530.547269	893.5104506	-1.501892915
5454	POU3F2	4029.186833	1421.991179	-1.502576189
10461	MERTK	1213.047009	426.9926442	-1.506352338
64388	GREM2	6.946207171	2.434540228	-1.5125761
90649	ZNF486	428.2092721	149.8057889	-1.51522266
2119	ETV5	1333.675607	464.8289915	-1.520635841
90634	N4BP2L1	315.1761018	109.4019064	-1.526520269
4255	MGMT	430.5412452	149.334211	-1.527606742
1184	CLCN5	1801.798988	624.0589491	-1.529683852
83604	TMEM47	5001.16355	1731.565131	-1.530187131
579	NKX3-2	106.7899464	36.86558705	-1.534429197
55630	SLC39A4	524.8996152	180.4093241	-1.540767637
196385	DNAH10	93.86536549	32.08977796	-1.548479122
138715	ARID3C	11.00361528	3.756087062	-1.550675196
10874	NMU	8.24555565	2.813378798	-1.551312904
147798	TMC4	199.4919491	68.00896068	-1.552533775
147407	SLC25A52	6.258127888	2.129867113	-1.55496772
441234	ZNF716	44.21569059	15.03621751	-1.556116732
613037	NPIPB13	88.19821355	29.97551238	-1.55696502
11170	FAM107A	19.76255129	6.707117682	-1.559004386
7087	ICAM5	1689.671342	572.8273568	-1.560570356
7857	SCG2	99.27798172	33.59956334	-1.563031303
64123	ADGRL4	15.02330017	5.081394427	-1.563905407
165215	FAM171B	6310.686284	2133.789405	-1.56437911
134429	STARD4	13.28538906	4.490855355	-1.564778317
103344718	HOTS	251.7671957	85.03647897	-1.565936551
345895	RSPH4A	148.1389688	49.99273106	-1.567160952
6261	RYR1	298.2509942	100.5752353	-1.568251833
138255	C9orf135	292.5983319	98.48341786	-1.570968808
7015	TERT	252.0558842	84.7116746	-1.573110919
5806	PTX3	2.274703512	0.760886259	-1.5799258
641700	ECSCR	2.274703512	0.760886259	-1.5799258
343172	OR2T8	2.275743754	0.761097975	-1.580184034
100529241	HSPE1-MOB4	472.2083389	157.9064566	-1.580353356
5077	PAX3	79.62068617	26.54036491	-1.584955098
59353	TMEM35A	3101.670502	1033.195583	-1.585932053
5577	PRKAR2B	17.34981572	5.772624723	-1.587620995
23127	COLGALT2	11691.55821	3881.543087	-1.590765013
728340	GTF2H2C	413.2780507	137.1605867	-1.591246763
56944	OLFML3	14.35291194	4.762902965	-1.591430401
8436	CAVIN2	87.49669932	29.02148054	-1.592107474
57644	MYH7B	20.40727845	6.741744508	-1.597889935

126969	SLC44A3	66.24616744	21.85851759	-1.599641429
257407	C2orf72	257.47261	84.73009021	-1.603472656
8693	GALNT4	1474.692826	484.6380162	-1.605434994
8987	STBD1	760.5790765	249.1554952	-1.610051858
2902	GRIN1	64.14881612	20.98382091	-1.61214524
8174	MADCAM1	93.22475522	30.47465632	-1.613103153
25854	FAM149A	656.4791071	213.5512202	-1.620166956
3075	CFH	6.607512197	2.136258871	-1.6290207
664	BNIP3	16371.78789	5289.80349	-1.629925847
169166	SNX31	107.3322765	34.65922479	-1.63077269
9508	ADAMTS3	3.565935346	1.148681155	-1.634302148
93986	FOXP2	1488.15415	478.7007938	-1.636327872
112399	EGLN3	7.881659633	2.531992819	-1.638226135
53826	FXYD6	26.12513457	8.392151002	-1.638325927
4613	MYCN	2.807484541	0.899981027	-1.641311586
26051	PPP1R16B	78.43032802	25.13979283	-1.641438873
83643	CCDC3	53.79062305	17.23790163	-1.641770534
440836	ODF3B	308.7382242	98.58957019	-1.646877174
50515	CHST11	1547.473377	492.7613477	-1.650953589
10098	TSPAN5	30.75933388	9.788145968	-1.65191674
287	ANK2	7884.100186	2507.072996	-1.652942107
57576	KIF17	500.4204191	158.9780267	-1.654313282
55885	LMO3	8.917455387	2.831737595	-1.65494451
3109	HLA-DMB	92.24780172	29.05097656	-1.666927871
115111	SLC26A7	14.27426769	4.490706759	-1.66840231
116844	LRG1	168.7147902	53.03477714	-1.66957584
100129083	LOC100129083	12.2472736	3.84262872	-1.672295129
8654	PDE5A	130.9044399	41.05446252	-1.672903077
79789	CLMN	52.06257631	16.29186909	-1.676094578
25837	RAB26	4.864360948	1.520927931	-1.677298492
645121	CCNI2	79.858563	24.81730797	-1.686100483
8482	SEMA7A	53.74427154	16.68408777	-1.687638183
8711	TNK1	24.07822832	7.460137102	-1.690455192
7380	UPK3A	2.575835226	0.795190096	-1.695668615
116135	LRRC3B	2.578794684	0.795739249	-1.69632925
2827	GPR3	45.66984004	14.02119525	-1.703632398
29969	MDFIC	3125.261409	957.1938195	-1.707093881
7694	ZNF135	15.67774642	4.796492202	-1.708666579
2047	EPHB1	122.9090487	37.56932711	-1.709963951
29953	TRHDE	5.921879251	1.809504724	-1.7104602
56171	DNAH7	42.8795361	13.06955729	-1.714079025
117166	WFIKKN1	232.4246	70.7972054	-1.714998454
347442	DCAF8L2	22.65000938	6.845842129	-1.72621172

149483	CCDC17	41.5208737	12.51691365	-1.729957927
49	ACR	15.23507442	4.59263216	-1.730003404
10253	SPRY2	2370.012607	712.5175362	-1.733897307
222223	KIAA1324L	598.5445924	178.9789823	-1.741668554
83878	USHBP1	42.58272324	12.72051519	-1.743111113
80125	CCDC33	2.866174678	0.85432064	-1.746276995
27285	TEKT2	2.865048436	0.850686312	-1.751860383
151835	CPNE9	13.98039783	4.142708267	-1.754759281
4922	NTS	31.82193006	9.394722306	-1.760098917
126326	GIPC3	17.60616732	5.191744619	-1.76178956
56099	PCDHGB7	218.4406395	64.39107906	-1.762308554
10256	CNKSR1	12.17843677	3.582960373	-1.765104967
338645	LUZP2	1265.936246	371.8804588	-1.767293905
54551	MAGEL2	51.0590933	14.98788297	-1.768371301
10673	TNFSF13B	6.241472464	1.828855673	-1.770945197
57188	ADAMTSL3	97.18117503	28.46097798	-1.77169164
6319	SCD	37581.56419	11000.66547	-1.772434315
8537	BCAS1	108.6019396	31.73239369	-1.775021611
7291	TWIST1	124.5263626	36.35404423	-1.776263426
779	CACNA1S	5.215010776	1.521626036	-1.777056397
57369	GJD2	51.69663817	15.0780134	-1.777624106
79012	CAMKV	30.39027237	8.849540127	-1.779935214
169026	SLC30A8	2541.748821	737.9571268	-1.784212561
154141	MBOAT1	1745.53655	506.4322837	-1.785229285
340578	DCAF12L2	12.23349293	3.539764252	-1.789111198
100131390	SP9	7.55693673	2.180640346	-1.793049699
171024	SYNPO2	2165.621486	624.1603311	-1.794792533
11075	STMN2	826.153389	238.0061646	-1.795410725
586	BCAT1	3682.353866	1058.943842	-1.798002191
54549	SDK2	406.3216219	116.6593945	-1.800319646
3166	HMX1	10.46217651	2.987285918	-1.808275784
7570	ZNF22	21.92444393	6.233223132	-1.814489989
9473	THEMIS2	322.3719865	91.44687855	-1.817720551
27345	KCNMB4	222.6903649	62.9617227	-1.822492218
5024	P2RX3	10.22345742	2.882989573	-1.826245652
340120	ANKRD34B	10.23495387	2.879633774	-1.82954735
400935	IL17REL	16.27594818	4.55831523	-1.83616899
23452	ANGPTL2	61.81779378	17.29962121	-1.837281717
402573	C7orf61	8.159836665	2.274370232	-1.843073152
80312	TET1	3226.468844	896.8681388	-1.8469883
126014	OSCAR	293.7293977	80.57144291	-1.866147166
6098	ROS1	56.64530908	15.48472804	-1.871110443
10215	OLIG2	4.488137246	1.225586641	-1.872644317

9844	ELMO1	179.1351944	48.88732274	-1.873516504
256691	MAMDC2	1201.200882	327.7782989	-1.873685193
130367	SGPP2	146.4783183	39.96244918	-1.873970223
27019	DNAI1	483.1536487	130.8403035	-1.884675046
254122	SNX32	3.459202786	0.93404157	-1.888880926
10409	BASP1	251.4469605	67.84309899	-1.889980137
1462	VCAN	8.213069477	2.211291082	-1.893032556
60675	PROK2	70.53192319	18.96619643	-1.894845996
1010	CDH12	61.03736875	16.37192108	-1.898469153
7145	TNS1	8.488076617	2.271372433	-1.901873397
4885	NPTX2	224.4439414	60.01426688	-1.902977742
1848	DUSP6	7541.789835	2013.864185	-1.904940556
91522	COL23A1	222.6349868	59.31605641	-1.908185739
8633	UNC5C	341.7791909	90.917039	-1.910441958
487	ATP2A1	113.3667759	30.11755791	-1.912321197
152273	FGD5	72.86825647	19.32277898	-1.914987875
8277	TKTL1	7.184682563	1.896086817	-1.921899393
5125	PCSK5	11.42886788	3.00692237	-1.926322973
84889	SLC7A3	2742.810065	715.1188194	-1.939399846
5789	PTPRD	74.03612781	19.22446742	-1.945285811
51557	LGSN	96.10552529	24.92014236	-1.947307066
80709	AKNA	714.2641644	184.4002741	-1.953616941
79152	FA2H	2684.031565	690.6819078	-1.9583083
646457	C19orf67	4.832348455	1.2410242	-1.961193241
3488	IGFBP5	3.492642547	0.896845935	-1.96138692
285141	ERICH2	44.57566797	11.4438997	-1.961677659
3046	HBE1	3.502719843	0.888020597	-1.979810558
152078	PQLC2L	9.129911376	2.308724238	-1.983504993
92565	FANK1	6.450517891	1.617864397	-1.995324303
4131	MAP1B	3190.068575	799.1513017	-1.99704686
2	A2M	33.00545017	8.263164979	-1.997937898
10630	PDPN	7.80681489	1.944199613	-2.005557713
84101	USP44	1525.900378	378.9211986	-2.009691017
11320	MGAT4A	38.47245896	9.522317303	-2.014441434
55760	DHX32	312.2007239	77.19352834	-2.015922076
6691	SPINK2	65.10025383	16.07870233	-2.017512193
81031	SLC2A10	1024.410507	252.549013	-2.020158647
710	SERPING1	4368.724048	1076.744711	-2.020535744
5333	PLCD1	1960.455203	482.6729606	-2.022070762
135932	TMEM139	23.96418408	5.898387259	-2.02248737
152831	KLB	101.4691446	24.94090262	-2.024455507
91752	ZNF804A	6.480570793	1.590694127	-2.02646444
206338	LVRN	36.44785651	8.840254109	-2.043674228

55603	FAM46A	49.22188999	11.81537777	-2.058634297
221914	GPC2	127.9885444	30.60669226	-2.064095646
407977	TNFSF12-TNFSF13	83.37433152	19.92291391	-2.065174619
414332	LCN10	19.78658241	4.725927429	-2.065853067
388553	BHMG1	6.783668808	1.612884545	-2.072422567
6480	ST6GAL1	1032.587513	244.7453715	-2.076910576
3352	HTR1D	152.48648	35.74674739	-2.092797452
1295	COL8A1	770.1412858	179.7300136	-2.099291791
6781	STC1	4.115463482	0.955421935	-2.106845012
54768	HYDIN	7.110031456	1.633553193	-2.121842509
10716	TBR1	7.116774929	1.634452922	-2.122415788
286046	XKR6	4.117548292	0.945067945	-2.12329561
341405	ANKRD33	11.77438971	2.666256308	-2.142764905
3270	HRC	300.369109	67.48703014	-2.154054276
8447	DOC2B	112.8910488	25.34725228	-2.15502983
84891	ZSCAN10	11.808361	2.639168883	-2.161653154
55601	DDX60	53.26628443	11.85443212	-2.167796097
4504	MT3	9.073324946	2.018723532	-2.168187983
7148	TNXB	189.1862601	42.02835388	-2.170372458
9886	RHOBTB1	275.4140162	61.05230758	-2.173484252
11174	ADAMTS6	94.76945651	21.00643822	-2.1735906
79094	CHAC1	313.9292538	69.34926271	-2.178487024
4828	NMB	10.49203198	2.287591325	-2.197392866
8076	MFAP5	673.4546233	146.3059118	-2.202592676
26052	DNM3	45.54367659	9.863907295	-2.207019614
28999	KLF15	25.97887288	5.603916324	-2.21283152
284111	SLC13A5	6.107727064	1.317287649	-2.213065181
1285	COL4A3	6.103827502	1.301669344	-2.229351176
5652	PRSS8	40.40080425	8.610300503	-2.230248519
494470	RNF165	100.5008842	21.40550087	-2.231154696
121256	TMEM132D	6.408356417	1.363245659	-2.232908828
1268	CNR1	24.95505494	5.280804817	-2.240502356
168620	BHLHA15	23.84903402	5.029921061	-2.245323169
84634	KISS1R	538.5796034	113.1982652	-2.250307745
4857	NOVA1	209.9458546	44.0280789	-2.253521501
131540	ZDHHC19	124.7221099	25.96440541	-2.264110146
64220	STRA6	161.4519781	33.54651609	-2.266870266
28316	CDH20	8.08040576	1.673736961	-2.271354924
10826	FAXDC2	777.2868018	160.4135534	-2.276650979
146429	SLC22A31	115.2119893	23.69848466	-2.281424138
1004	CDH6	42939.58395	8787.260191	-2.288822895
799	CALCR	1111.90826	226.6324081	-2.294611676
153579	BTNL9	24.5880906	4.995227505	-2.299337415

391059	FRRS1	78.52318703	15.85317884	-2.308346574
1593	CYP27A1	26.60759524	5.331269136	-2.319287207
84898	PLXDC2	927.7623764	185.7209091	-2.320619094
4990	SIX6	5.047951117	1.008568002	-2.323389579
22837	COBLL1	951.0159044	189.9366099	-2.32395146
460	ASTN1	100.4625383	19.82440658	-2.341308045
79727	LIN28A	152.9776082	30.08432241	-2.346236728
4038	LRP4	3941.280223	766.5627689	-2.362188493
64218	SEMA4A	15.77065618	3.065475995	-2.363059676
2898	GRIK2	20.56006213	3.992845953	-2.364355306
6328	SCN3A	62.98774164	12.2241359	-2.365338599
283710	LOC283710	12.72135728	2.461132558	-2.369858336
2348	FOLR1	102.6864336	19.81973294	-2.373236165
10251	SPRY3	1583.746207	305.651235	-2.373382862
8224	SYN3	7.071780113	1.352179894	-2.386786316
81578	COL21A1	81.21470908	15.50404537	-2.389096345
4692	NDN	203.6356381	38.806463	-2.391621218
10144	FAM13A	21.49063437	4.089976162	-2.39354373
1404	HAPLN1	118.5156134	22.52552594	-2.395444437
83592	AKR1E2	51.21589476	9.596571484	-2.416000639
402665	IGLON5	76.13448562	14.26122963	-2.416451701
91351	DDX60L	207.4067865	38.60398144	-2.425641548
8128	ST8SIA2	5.401221845	1.002510616	-2.429668291
136288	C7orf57	3585.224296	662.0777648	-2.436990793
130399	ACVR1C	12.79338625	2.361134499	-2.437846048
51050	PI15	1102.567076	203.1561251	-2.440205659
5210	PFKFB4	1542.017886	282.6749688	-2.447603458
7075	TIE1	34.94494211	6.368992072	-2.455946675
3816	KLK1	38.65519949	7.027753168	-2.459527057
92162	TMEM88	2235.478209	405.0597448	-2.464376862
22925	PLA2R1	9.41328836	1.701241189	-2.468111101
4897	NRCAM	89.30783582	15.89959325	-2.489796904
2028	ENPEP	56.22938726	9.974123482	-2.495062357
6857	SYT1	1388.001589	241.9667865	-2.520128284
1482	NKX2-5	53.89209356	9.299834254	-2.534796723
1946	EFNA5	69.33749526	11.96243731	-2.535124358
56979	PRDM9	663.0849816	113.044221	-2.552306538
50863	NTM	605.85544	103.1892921	-2.55368033
254173	TTLL10	6.060434018	1.028203989	-2.559294602
10194	TSHZ1	235.9499465	39.80184606	-2.567573593
51305	KCNK9	46.45012457	7.724244428	-2.588216742
54514	DDX4	18.45299263	3.066000898	-2.589424781
22865	SLITRK3	257.8922439	42.76327555	-2.592324116

23327	NEDD4L	91.97907711	15.21202359	-2.59609364
100271846	ERVV-2	72.69733917	11.94651308	-2.605312972
80059	LRRTM4	32.95302093	5.407793632	-2.607298726
6549	SLC9A2	11.01586469	1.805854657	-2.608829055
3174	HNF4G	26.8677489	4.397287551	-2.611189669
9940	DLEC1	32.95060369	5.391073627	-2.611660379
9315	NREP	2262.440707	366.2588151	-2.626944596
51309	ARMCX1	57.89794041	9.323531169	-2.634563663
23349	PHF24	19.45499649	3.109504438	-2.645384138
8433	UTF1	21.71349964	3.468982784	-2.646007683
80309	SPHKAP	6.695656858	1.059717661	-2.659545653
164832	LONRF2	27.90192657	4.410774137	-2.661260949
727909	GOLGA8Q	6.984948491	1.093457808	-2.675351922
4118	MAL	165.3948409	25.73865184	-2.68390584
255488	RNF144B	69.01301409	10.69118403	-2.690446805
7837	PXDN	71.28511911	11.01281031	-2.694418273
2043	EPHA4	9.323006859	1.438228907	-2.696502017
10468	FST	2359.525628	362.0447138	-2.70425705
7180	CRISP2	7.009235003	1.074936561	-2.705005476
5168	ENPP2	7.0181079	1.073080886	-2.709323302
10017	BCL2L10	7.033905183	1.065552783	-2.722723827
653220	XAGE1A	13.75548926	2.074347981	-2.729277617
653067	XAGE1B	13.75548926	2.074347981	-2.729277617
131149	OTOL1	60.60592405	9.012168611	-2.749512609
57172	CAMK1G	14.6924061	2.182708317	-2.750879421
2566	GABRG2	466.9770848	67.49631478	-2.790471117
1296	COL8A2	81.15431079	11.63274586	-2.802476052
5797	PTPRM	703.3675276	99.44592985	-2.822294501
388531	RGS9BP	84.17630985	11.69331921	-2.847729761
7425	VGF	2227.665518	305.7771583	-2.864980084
79875	THSD4	25.49563224	3.475520036	-2.874949347
56479	KCNQ5	79.09416597	10.75809202	-2.87814905
7078	TIMP3	380.274591	51.6857533	-2.87920297
9743	ARHGAP32	459.1288726	61.88257217	-2.89129409
339453	TMEM240	21.11566469	2.806671186	-2.911381701
2619	GAS1	21.09301485	2.790826099	-2.918001181
83482	SCRT1	158.039463	20.86611846	-2.92105069
729408	GAGE2D	8.344860312	1.101479142	-2.921445724
148113	CILP2	160.27564	20.88986326	-2.939680216
79632	FAM184A	85.08913301	11.08201236	-2.940755011
9427	ECEL1	499.8420522	64.87070705	-2.945833213
220382	FAM181B	223.1396238	28.74003109	-2.956813194
5325	PLAGL1	833.1558486	106.9282681	-2.961943087

57502	NLGN4X	11.36241245	1.455893481	-2.964292467
10351	ABCA8	40.26429993	5.155756638	-2.965245177
1730	DIAPH2	16.71978835	2.136214399	-2.968428231
2122	MECOM	166.0497535	20.82483771	-2.995238424
144568	A2ML1	174.5349859	21.86588147	-2.996762843
642475	MROH6	133.3614894	16.62873388	-3.003591891
167410	LIX1	1212.827001	150.7920904	-3.007741117
79729	SH3D21	316.1144111	38.95691768	-3.020495368
63982	ANO3	31.5139937	3.853312819	-3.031821379
7103	TSPAN8	23.11274767	2.818804404	-3.035533501
1299	COL9A3	251.7909934	30.42544999	-3.048876173
27123	DKK2	83.82574563	10.04671507	-3.060669544
23022	PALLD	1040.973994	123.7872531	-3.071999361
3872	KRT17	9.645876027	1.142205631	-3.078089867
7020	TFAP2A	507.175759	59.64577004	-3.087994058
22797	TFEC	21.09543751	2.475181742	-3.091324644
79953	SYNDIG1	9.985742159	1.140031703	-3.130795711
57554	LRRC7	9.991086846	1.14045228	-3.131035544
81704	DOCK8	22.37999597	2.551396068	-3.132850997
3948	LDHC	13.32161358	1.494927076	-3.155621824
2888	GRB14	174.8858988	18.94568953	-3.206472415
5443	POMC	65.94075649	7.126335688	-3.209938089
7412	VCAM1	35.88402915	3.855900385	-3.218202203
116984	ARAP2	17.37952252	1.839225559	-3.240218122
79827	CLMP	73.93975472	7.806069312	-3.24368208
4045	LSAMP	37.25811142	3.842013103	-3.277620203
9510	ADAMTS1	22.00961082	2.218352562	-3.310573059
79772	MCTP1	588.9686378	58.99786657	-3.319456122
6326	SCN2A	81.05646105	8.110364754	-3.321088484
124602	KIF19	15.66247756	1.523986267	-3.361390635
10085	EDIL3	19.69419591	1.893177103	-3.378889231
56884	FSTL5	109.0909087	10.42883742	-3.386880633
151254	C2CD6	27.98868926	2.624179693	-3.414905507
148198	ZNF98	30.80276501	2.885151651	-3.416340803
5087	PBX1	92.17103885	8.507319456	-3.437536977
203328	SUSD3	24.07937607	2.193586145	-3.456434741
3485	IGFBP2	1164.825159	103.0392255	-3.498847863
2904	GRIN2B	13.64061263	1.20377861	-3.502266449
10417	SPON2	40.22165485	3.539595268	-3.506316129
9627	SNCAIP	14.57699402	1.246013705	-3.548301404
58494	JAM2	317.8127571	26.54404313	-3.581716996
3912	LAMB1	2076.952607	172.1017342	-3.593134756
26011	TENM4	15.24481006	1.258589903	-3.598437996

30010	NXPH1	47.93510051	3.893525525	-3.621933468
4345	CD200	14.98331085	1.216857045	-3.622124853
10840	ALDH1L1	1810.023356	143.606092	-3.655819457
339479	BRINP3	510.1035669	40.46479761	-3.656050904
448831	FRG2	70.8881731	5.568348372	-3.670223572
91851	CHRDL1	472.5916522	37.08690502	-3.671612364
26166	RGS22	15.64857578	1.221837235	-3.678907343
83690	CRISPLD1	4912.690736	380.1312212	-3.691943994
5156	PDGFRA	3134.125365	235.4705046	-3.734444626
7001	PRDX2	210.8325902	15.72851125	-3.744643867
794	CALB2	62.80499135	4.566628055	-3.781678026
79674	VEPH1	32.70009884	2.310676488	-3.822907805
441521	CT45A5	67.1547085	4.721150324	-3.830278232
22871	NLGN1	95.76845834	6.565003925	-3.866682799
100288801	FRG2C	247.1715517	16.43005137	-3.91110381
100529144	CORO7-PAM16	50.49292065	3.234243139	-3.964581084
4593	MUSK	20.97175125	1.302347358	-4.009261143
55103	RALGPS2	196.5027165	11.87404465	-4.048665909
4772	NFATC1	149.1547677	8.890851684	-4.068344658
25928	SOSTDC1	302.5569811	17.74901694	-4.091395848
282890	ZNF311	33.4646434	1.957220422	-4.095759495
1286	COL4A4	39.51587111	2.302428801	-4.101203763
22999	RIMS1	452.5275261	24.68860438	-4.196088361
56776	FMN2	134.6244024	7.240886043	-4.216629885
28954	REM1	25.3288036	1.310980652	-4.272060634
441581	FRG2B	64.12692279	3.31773497	-4.27265963
760	CA2	185.1841691	9.560777892	-4.275688957
8784	TNFRSF18	53.28083053	2.66637517	-4.320664878
345557	PLCXD3	106.246262	5.290648217	-4.32782378
100529239	RPS10-NUDT3	29.86537838	1.430788353	-4.383591815
1902	LPAR1	103.5541204	4.67443758	-4.469448356
170825	GSX2	808.9700867	36.47188916	-4.471229522
11030	RBPMS	126.6822009	5.680359573	-4.47908777
2045	EPHA7	165.8864467	7.311851408	-4.503815457
114907	FBXO32	155.79763	6.643069217	-4.55167953
1535	CYBA	287.7782549	12.24897168	-4.55422504
285313	IGSF10	42.0417067	1.740807366	-4.593992766
64849	SLC13A3	421.2864204	16.87891577	-4.641507274
2173	FABP7	101.1230671	4.015979134	-4.654216543
6335	SCN9A	257.3167505	9.934899307	-4.694896219
9074	CLDN6	611.379512	23.10238627	-4.725954425
221002	RASGEF1A	362.5452814	12.63556564	-4.842599043
143662	MUC15	383.4474065	13.28748231	-4.850889036

80034	CSRNP3	671.0211838	22.83209732	-4.877223018
26002	MOXD1	474.9352612	15.89557515	-4.901033748
29951	PDZRN4	241.7530907	6.716624039	-5.169654247
8492	PRSS12	153.6930957	3.782944427	-5.344398964
429	ASCL1	88.94690745	2.155613262	-5.366774173
6332	SCN7A	173.5081638	3.501290451	-5.630972989
9388	LIPG	234.6016801	4.498073528	-5.70476229
118427	OLFM3	145.8850053	2.541010357	-5.843285538
10752	CHL1	818.0007857	14.09849559	-5.858489099

Table S1b

Gene ID	Gene symbol	E400K Expression	p190A Expression	log2FoldChange (p190A/E400K)
55170	PRMT6	14.2895141	375.0521739	4.714062539
2641	GCG	17.37080227	450.1394333	4.695635662
221981	THSD7A	8.975398207	229.3801903	4.675621045
6641	SNTB1	1.353630817	32.24872423	4.574335865
56892	TCIM	48.80295729	1129.682245	4.532804649
344901	OSTN	1.331526176	30.23816252	4.505217779
169792	GLIS3	1.677301573	37.12359753	4.468122515
51481	VCX3A	1.429755852	31.37032455	4.455559739
94240	EPSTI1	13.22814535	282.585642	4.417005457
5796	PTPRK	21.35931588	437.9892131	4.357957994
5999	RGS4	9.214371795	178.9129802	4.279228438
7058	THBS2	4.048053524	77.99325651	4.26804912
117154	DACH2	6.202736201	119.3780733	4.266489296
960	CD44	17.58263903	332.9025227	4.242876273
4071	TM4SF1	10.47738361	191.356997	4.190916255
4608	MYBPH	6.181304382	105.9466884	4.099283375
2846	LPAR4	5.12145018	84.13434332	4.03807054
127254	ERICH3	1.251155181	18.79980249	3.909384861
1293	COL6A3	1.238894822	18.16426843	3.873977644
4747	NEFL	2.300438686	33.5288191	3.865420762
11098	PRSS23	485.1754628	6905.827128	3.831235725
93649	MYOCD	2.935823553	41.51924569	3.821943065
130576	LYPD6B	11.5372505	160.0238217	3.793915331
190	NR0B1	6.045668626	82.53123239	3.770966374
24141	LAMP5	44.52262953	589.4695525	3.72680659
3777	KCNK3	8.743319239	112.2249714	3.682068842
57282	SLC4A10	1.239164535	15.2290729	3.619388453
168667	BMPER	1.885332444	22.81649425	3.597186295
5028	P2RY1	13.14209806	158.5522346	3.592690693
4222	MEOX1	413.9826072	4844.138867	3.548598162
51702	PADI3	43.75301799	499.927427	3.514264239
25878	MXRA5	1.206283958	13.58861268	3.493756711
7056	THBD	1.522910271	16.913795	3.473297551
6422	SFRP1	19.85390864	215.569702	3.44065946
90293	KLHL13	4.309186417	46.71458918	3.438385764
55024	BANK1	6.589701896	70.84463754	3.426373548
26289	AK5	17.17297329	181.801163	3.404149677
140706	CCM2L	227.8527332	2409.651719	3.402651059
1828	DSG1	7.79974507	81.26320656	3.381103417
79836	LONRF3	9.672628509	99.77176899	3.366651758

10568	SLC34A2	287.1410106	2931.821147	3.351965794
1381	CRABP1	68.63315145	688.6672124	3.326829485
800	CALD1	40.81094729	408.2749977	3.322513118
182	JAG1	597.0604768	5906.260217	3.306295944
8660	IRS2	8.293795214	81.71656124	3.300524164
84630	TTBK1	9.308057282	91.53094837	3.297707634
347902	AMIGO2	18.48506792	180.3575138	3.286427278
130574	LYPD6	3.540654799	34.33964728	3.277787123
3815	KIT	13.77279559	133.5659442	3.277658874
140862	ISM1	2.203867369	21.18145468	3.264692364
8707	B3GALT2	3.924566877	37.60507704	3.260322103
203859	ANO5	10.43869067	99.43177511	3.251766197
51286	CEND1	40.71727251	385.3054126	3.242289623
24	ABCA4	2.533813004	23.84990911	3.234601806
84072	HORMAD1	11.33729286	106.4366538	3.230846963
165	AEBP1	38.05819985	353.7368197	3.216397165
51232	CRIM1	521.0951479	4789.766484	3.200336595
1825	DSC3	67.28582423	616.350954	3.195377569
9211	LGI1	26.62982918	235.6969275	3.145817873
3754	KCNF1	1.47204905	12.99656565	3.142232792
2857	GPR34	1.478606115	13.00903118	3.137203835
4005	LMO2	8.580886579	75.42524278	3.135848815
167838	TXLNB	26.65528588	232.7011502	3.125984781
4110	MAGEA11	2.552663237	22.21648919	3.121555723
84166	NLRC5	4.192926759	36.32410498	3.114897716
2300	FOXL1	2.525416477	21.87282705	3.114546466
6588	SLN	44.72975996	386.4162921	3.110849001
4629	MYH11	44.84777758	383.2013062	3.094994079
2303	FOXC2	3.824791767	31.75538591	3.053548192
2863	GPR39	4.578732074	37.27511261	3.025192658
3620	IDO1	9.998341881	80.74670591	3.013642642
286	ANK1	160.7212731	1295.800037	3.011210302
83857	TMTC1	69.75403825	550.250368	2.979739559
55466	DNAJA4	19.70686744	154.3013315	2.96898014
3199	HOXA2	15.98106743	123.8560445	2.954228599
7042	TGFB2	643.0041731	4948.564394	2.944110046
347365	ITIH6	3.5289749	27.13021678	2.942579502
388585	HES5	95.94997922	725.0179844	2.917662383
3489	IGFBP6	60.59438134	457.3634078	2.916085015
64135	IFIH1	15.3868732	115.1406559	2.903625342
124925	SEZ6	10.47091693	77.90751146	2.895374648
10046	MAMLD1	48.11098855	353.9993841	2.879308502
7424	VEGFC	5.544887293	40.65046038	2.87404165

339761	CYP27C1	25.14396049	181.0957054	2.848468519
1839	HBEGF	371.866427	2677.003131	2.847762418
9411	ARHGAP29	8.882405362	63.94280653	2.84775975
80099	C7orf69	2.107994698	15.02653521	2.833569249
1794	DOCK2	1.78611784	12.71579598	2.831722603
999	CDH1	294.7136157	2073.743541	2.814851868
4883	NPR3	1.06627917	7.497566619	2.813837226
26064	RAI14	77.68070423	546.1180408	2.813584631
131578	LRRC15	6.592565598	46.20066054	2.809001552
22822	PHLDA1	552.1127243	3867.068377	2.808205519
2890	GRIA1	5.195022934	36.37494031	2.807742857
59277	NTN4	163.6874757	1132.466719	2.790452807
54935	DUSP23	11.30212709	78.04348553	2.787683896
9394	HS6ST1	1317.965033	8988.19614	2.769719513
25945	NECTIN3	21.69993151	147.960614	2.769450798
11010	GLIPR1	74.94743678	507.4024438	2.759179424
6374	CXCL5	2.156915697	14.60237442	2.759161283
147409	DSG4	4.253461037	28.38025061	2.738178185
166979	CDC20B	3.133686823	20.9019997	2.737708061
2254	FGF9	3.769677882	24.89232688	2.723187941
2983	GUCY1B1	79.16793839	518.9656217	2.712650784
338596	ST8SIA6	4.89634169	32.0727192	2.711570529
89927	C16orf45	181.5907351	1186.274623	2.707675531
1012	CDH13	1.754066601	11.41460004	2.702104877
1435	CSF1	324.6148491	2104.369495	2.696587142
2766	GMPR	9.879844307	63.95999183	2.694609542
5787	PTPRB	28.50612448	184.4115154	2.693584929
54625	PARP14	63.95087577	411.6830906	2.686498172
5915	RARB	3.438275766	21.97806137	2.67630697
23708	GSPT2	55.15884988	352.3513989	2.675350661
5654	HTRA1	9.574769989	60.99959078	2.671489829
7504	XK	2.468197003	15.68010443	2.667405713
80243	PREX2	61.03840262	387.273182	2.665562488
80003	PCNX2	1.030380871	6.526941126	2.663229311
23057	NMNAT2	5.856894023	36.97102437	2.658187322
3306	HSPA2	1433.848768	9037.829626	2.656083494
11023	VAX1	7.858718325	49.47466612	2.654324023
1823	DSC1	53.17373545	334.407455	2.652821286
9148	NEURL1	16.03103371	99.98948955	2.640908997
2313	FLI1	1.775293092	11.05886308	2.639073944
117581	TWIST2	1.372939168	8.514310343	2.632621973
55796	MBNL3	65.26574078	404.4523417	2.631571916
861	RUNX1	12.92069286	79.13705696	2.614669978

131544	CRYBG3	5.947455753	36.33445774	2.610993837
10873	ME3	19.67197451	120.0178797	2.609035672
4921	DDR2	118.0050919	719.1571634	2.607457976
1244	ABCC2	9.169267738	55.47870744	2.597055747
3592	IL12A	2.061039278	12.44396831	2.594002721
11043	MID2	92.04820063	555.500061	2.593325645
3236	HOXD10	17.94482672	107.4695139	2.582287568
27063	ANKRD1	14.909269	89.28296002	2.582175333
246	ALOX15	21.99359216	131.380375	2.578594629
84871	AGBL4	1.703940507	10.16841858	2.577148455
6586	SLIT3	1.082169143	6.455619263	2.576629484
4773	NFATC2	145.644247	868.3006132	2.575745887
54885	TBC1D8B	3.491759904	20.60259138	2.560799544
84709	MGARP	37.50425162	219.5530691	2.549443643
771	CA12	147.3081213	859.6972166	2.544991665
642778	NPIPA3	1.393099036	8.086795122	2.537270238
116496	FAM129A	244.8743033	1419.986407	2.535763826
7045	TGFBI	7.981231121	46.172876	2.53236239
8605	PLA2G4C	24.69501445	142.2889534	2.526531945
4135	MAP6	110.0331755	631.9463799	2.521863583
286133	SCARA5	4.497564327	25.80840605	2.520625221
79971	WLS	4.412828437	25.23953142	2.515909562
84168	ANTXR1	4.764017583	27.20685677	2.513719647
4940	OAS3	164.9085663	938.8951047	2.509297644
90102	PHLDB2	9.238659032	52.46770222	2.505674241
63895	PIEZO2	0.989519272	5.580727356	2.495653455
57158	JPH2	32.85755664	184.8676543	2.492195715
8829	NRP1	2.440173735	13.69496254	2.488589546
84141	EVA1A	3.068043724	17.07135949	2.476187004
3237	HOXD11	33.17918688	184.0380102	2.471653327
27129	HSPB7	48.41223338	268.5126932	2.471546733
57822	GRHL3	12.90506199	71.51767106	2.470362685
1602	DACH1	179.6750771	986.8295409	2.457410598
55190	NUDT11	3.716818844	20.35262421	2.453074545
11096	ADAMTS5	1.072129369	5.864124332	2.451436692
22882	ZHX2	31.08428125	169.5681452	2.447608048
2903	GRIN2A	1.695824966	9.17770507	2.436146176
79899	PRR5L	2.369126534	12.78219929	2.431708927
288	ANK3	17.61489691	94.71691904	2.426826122
91683	SYT12	74.54215969	400.0717328	2.424130174
5396	PRRX1	9.825112623	52.50703901	2.417964991
153572	IRX2	19.60046743	104.733207	2.417758974
1800	DPEP1	194.8287293	1038.458171	2.414164769

2615	LRRC32	7.460643436	39.45059794	2.402675202
3623	INHA	1.674721687	8.847787475	2.401395371
2861	GPR37	0.999203685	5.276488704	2.40072749
79937	CNTNAP3	4.484816339	23.59965775	2.395645127
50964	SOST	5.165048841	27.15232837	2.394222021
80326	WNT10A	230.7987403	1212.249338	2.392979211
1901	S1PR1	1.317543177	6.907509289	2.390315357
6710	SPTB	24.01120358	124.8929474	2.378912385
84867	PTPN5	1.701605296	8.83546423	2.37640951
388697	HRNR	0.957373936	4.965504108	2.374785756
220963	SLC16A9	0.957373936	4.965504108	2.374785756
639	PRDM1	27.33038842	141.3302899	2.370492829
54738	FEV	9.573366685	49.3961971	2.367301702
4741	NEFM	1.013587411	5.217122766	2.363783871
79148	MMP28	6.440342348	33.12722411	2.362808034
341640	FREM2	10.78619999	55.23749367	2.356461173
55567	DNAH3	109.3054641	552.4030555	2.337355778
162494	RHBDL3	424.0386174	2122.056216	2.323195313
3198	HOXA1	24.58458721	122.6724155	2.31898484
7062	TCHH	1.322719536	6.58518292	2.315716325
55450	CAMK2N1	97.10790768	483.3456267	2.315394501
9478	CABP1	34.78979463	173.0249711	2.314244196
54453	RIN2	54.91163034	272.9523532	2.313465484
144448	TSPAN19	2.044846671	10.13718606	2.309592661
3778	KCNMA1	2.33058686	11.49204577	2.301870456
59352	LGR6	1.72670139	8.502791502	2.299917952
57094	CPA6	28.35032052	139.3939294	2.297730782
23072	HECW1	1.628382046	7.926415005	2.283229285
2104	ESRRG	143.7537037	699.4214949	2.282563008
9619	ABCG1	73.91818525	359.5170832	2.282059082
4313	MMP2	38.51321541	187.10481	2.280421165
5698	PSMB9	33.40207716	162.0981862	2.278858221
154	ADRB2	1.68593902	8.174556027	2.277588023
4907	NT5E	211.8039746	1025.877935	2.276057514
57549	IGSF9	1.697754674	8.18543507	2.269431096
5920	RARRES3	14.85162153	71.6027656	2.269394855
8638	OASL	25.38760857	122.3558534	2.268886711
54463	RETREG1	14.98321899	72.14184077	2.267488631
153	ADRB1	2.66364173	12.80924934	2.26571398
105372978	LINC01638	6.70333597	32.22648552	2.265295715
5457	POU4F1	20.58692139	98.96873033	2.265244668
169355	IDO2	0.968773966	4.646612612	2.261947372
85463	ZC3H12C	3.195324383	15.30140162	2.259629507

728130	NUTM2D	12.68525542	60.67776436	2.258015363
83593	RASSF5	23.2231446	110.7874885	2.254159721
25884	CHRDL2	1.660604653	7.905722991	2.25118876
5332	PLCB4	75.93179582	361.058709	2.249457408
153571	C5orf38	13.47953441	64.04189381	2.248245305
3239	HOXD13	11.50445053	54.46459902	2.243126729
388021	TMEM179	26.30861388	123.5588321	2.231590995
23670	TMEM2	30.64316112	143.4571135	2.226982471
1991	ELANE	28.99412387	135.6578303	2.226139874
24138	IFIT5	63.92521061	298.9440123	2.225418401
28513	CDH19	12.7993563	59.8307053	2.224814813
23544	SEZ6L	21.88724113	102.286478	2.224453416
60506	NYX	2.034417916	9.497656048	2.222955439
728118	NUTM2A	20.32918718	94.47666274	2.216405471
83697	SLC4A9	3.334388032	15.45293137	2.212386629
1141	CHRNB2	37.86217694	175.1020862	2.209367004
57522	SRGAP1	626.3161252	2889.07061	2.205642536
23569	PADI4	1.647534069	7.565592393	2.199144752
4994	OR3A1	3.379123673	15.44134129	2.192077018
23316	CUX2	38.99149647	177.8047798	2.189062677
115207	KCTD12	12.18593494	55.4785484	2.186713097
1007	CDH9	3.451323973	15.64453251	2.180436739
80896	NPL	5.010124084	22.70242894	2.179928421
144811	LACC1	3.641738349	16.50140283	2.179889501
57168	ASPHD2	15.89156576	71.95794105	2.178892629
84159	ARID5B	145.8470157	657.3692218	2.172248044
548596	CKMT1A	217.229347	970.4198091	2.159389981
80144	FRAS1	0.903473634	4.032243875	2.158028493
1159	CKMT1B	88.52267934	393.1874736	2.151098337
1015	CDH17	5.670388565	25.03689819	2.142536334
6999	TDO2	16.46905848	72.69702299	2.142138206
6252	RTN1	0.909970944	4.012894505	2.140750845
388662	SLC6A17	33.19229918	146.0598552	2.137639234
84627	ZNF469	9.794257366	43.04318751	2.135776907
8651	SOCS1	44.50396317	195.517733	2.13529374
6662	SOX9	7.779652708	34.12327623	2.13297851
90627	STARD13	4.370612617	19.0726982	2.125601537
340061	TMEM173	28.19579004	122.9844141	2.124923821
340156	MYLK4	4.331493973	18.78769022	2.116851096
5507	PPP1R3C	60.95146418	263.046675	2.109586031
100528021	ST20-MTHFS	2.855874151	12.30906062	2.107716354
23052	ENDOD1	15.78081151	67.82577146	2.103662156
64065	PERP	39.71015809	170.6536396	2.103491174

10344	CCL26	0.995936933	4.272890997	2.101086221
2150	F2RL1	206.2410683	884.2891596	2.100186561
8038	ADAM12	0.934086685	3.988523263	2.094226346
23657	SLC7A11	64.11358106	273.6818895	2.093798072
8435	SOAT2	1.250282289	5.329267634	2.091683423
84675	TRIM55	10.54122749	44.9021675	2.090742214
4103	MAGEA4	12.0652441	51.36108203	2.089818493
3106	HLA-B	149.4605784	635.1440253	2.087318764
51809	GALNT7	27.91521324	118.3860281	2.084375342
9586	CREB5	1.634951892	6.93302529	2.084236837
64116	SLC39A8	85.16027695	361.0785975	2.084060362
55806	HR	131.5469471	555.7174403	2.078773751
220382	FAM181B	6.319181959	26.69459552	2.078737976
6236	RRAD	21.55110611	91.01461953	2.078336384
8714	ABCC3	7.339398681	30.96207786	2.076768521
1558	CYP2C8	1.581696194	6.668960644	2.075989416
79822	ARHGAP28	41.30216026	173.911837	2.074066983
943	TNFRSF8	6.33891114	26.67638397	2.07325617
10788	IQGAP2	2.65720097	11.17201344	2.071909963
3973	LHCGR	33.60085503	141.1369445	2.070525832
26115	TANC2	1474.924653	6183.922273	2.06788093
102724265	LOC102724265	0.887867526	3.720910092	2.06723919
53335	BCL11A	113.5269319	475.7410282	2.067141863
9573	GDF3	4.332876606	18.14586945	2.066244128
641517	DEFB109B	0.88608177	3.709928955	2.065879814
100131137	BSPH1	1.915450808	7.964307747	2.055864993
54947	LPCAT2	94.59964115	392.8238931	2.053976067
54809	SAMD9	2.700454023	11.20761346	2.053205214
51334	PRR16	215.2085043	891.0157398	2.049715828
9900	SV2A	57.65587896	238.6550809	2.049387424
3707	ITPKB	63.22884785	260.4933949	2.042591955
3908	LAMA2	2292.96867	9446.332739	2.042537712
23017	FAIM2	2.640577257	10.86772987	2.041125354
388849	CCDC188	4.655818346	19.134375	2.039060103
2919	CXCL1	0.903764495	3.711439154	2.037959931
159686	CFAP58	2.590428223	10.62158395	2.035736411
2977	GUCY1A2	2.01286943	8.227227877	2.031152812
3751	KCND2	6.667020067	26.99306907	2.017475044
5583	PRKCH	4.325641529	17.45857342	2.01294966
5950	RBP4	5.757488512	23.23307802	2.012668769
79983	POF1B	2066.692597	8335.697401	2.01197909
10761	PLAC1	45.97359744	185.0716817	2.009206695
3625	INHBB	1.571821281	6.324312909	2.008471561

116441	TM4SF18	3.363628374	13.52427181	2.007460691
64759	TNS3	2411.166818	9690.16289	2.006789449
152485	ZNF827	6.314383597	25.36587745	2.006175255
284306	ZNF547	7.739564122	30.94665165	1.999459098
9947	MAGEC1	0.856821855	3.425186207	1.999115236
134	ADORA1	16.79798956	67.05746339	1.997109334
23551	RASD2	27.26966372	108.8578216	1.997076257
26230	TIAM2	71.16800737	283.2696243	1.992875158
3371	TNC	1.987195332	7.887320737	1.988801621
219670	ENKUR	198.280175	786.2386087	1.987426773
56243	KIAA1217	3.596845752	14.23524459	1.984663084
100133941	CD24	94.22451815	372.891972	1.98458332
11107	PRDM5	0.862144014	3.406841654	1.98243411
3238	HOXD12	0.994505795	3.928785488	1.982031716
387849	REP15	2.737794437	10.80848149	1.981077817
161145	TMEM229B	18.51638957	73.06422993	1.980362457
5359	PLSCR1	9.381858069	36.90153499	1.975735249
11230	PRAF2	187.4154905	736.4341881	1.974316402
4330	MN1	988.3831697	3867.28924	1.96818032
1812	DRD1	0.871456732	3.397784436	1.963093387
2312	FLG	2.29884777	8.945762535	1.960293528
116372	LYPD1	1.678783411	6.528074759	1.959241467
132671	SPATA18	1.555228606	6.041633619	1.957812038
168507	PKD1L1	14.77152381	57.31694939	1.956143166
1840	DTX1	109.3895743	424.4473934	1.956110511
64919	BCL11B	1.216062667	4.71620384	1.955408498
51435	SCARA3	64.22616045	248.7790703	1.95363216
284546	C1orf185	0.882068497	3.412961959	1.952061736
3965	LGALS9	11.5648399	44.60977163	1.947614469
2188	FANCF	9.999605704	38.56453497	1.9473316
10586	MAB21L2	1.56159315	6.012201299	1.944874684
6041	RNASEL	7.361366388	28.34113385	1.944851994
140733	MACROD2	35.78525095	137.3939388	1.940881358
29943	PADI1	10.65148613	40.87472677	1.940154353
1747	DLX3	15.42166079	59.17074659	1.939925958
29116	MYLIP	5.675220376	21.71533682	1.93596601
11240	PADI2	337.4421963	1287.867337	1.932271694
144535	CFAP54	16.1586468	61.58094642	1.930177655
91523	PCED1B	1.230131078	4.687064063	1.929872462
2159	F10	18.80158999	71.10708355	1.919138615
2346	FOLH1	252.3772518	953.4238008	1.917535763
219595	FOLH1B	23.8163989	89.73548026	1.913723231
2202	EFEMP1	27.68416419	104.1921368	1.912113533

392255	GDF6	0.828958451	3.11968369	1.912028062
64478	CSMD1	105.2642344	396.0202365	1.911558817
5996	RGS1	6.376480079	23.97616969	1.910769042
133396	IL31RA	7.723930741	28.93549634	1.905433258
4675	NAP1L3	9.205555312	34.32967729	1.898879638
9052	GPRC5A	32.30954118	120.0530897	1.893640364
102724428	SIK1B	303.6183622	1127.619955	1.892949964
3235	HOXD9	69.4997934	258.029845	1.89245735
54714	CNGB3	2.241694936	8.310610742	1.890364543
4938	OAS1	1.191375731	4.412157414	1.888855787
23213	SULF1	373.2166323	1381.845312	1.88851094
6000	RGS7	0.906560887	3.355743249	1.888156514
283537	SLC46A3	0.91001368	3.365227684	1.88674398
85352	SHISAL1	13.16345463	48.62897835	1.885278121
10998	SLC27A5	2.247755166	8.297599722	1.884209163
51458	RHCG	2.890416743	10.66409431	1.883412022
6387	CXCL12	1.192277392	4.398148192	1.883176287
84631	SLITRK2	194.682684	716.6600591	1.880164383
3204	HOXA7	169.9828168	625.3102032	1.879183143
55553	SOX6	4.944407148	18.16503658	1.877294819
28996	HIPK2	288.0160705	1056.812687	1.875498474
1001	CDH3	3378.83297	12396.04595	1.875283069
57144	PAK5	0.845858155	3.102613652	1.8749964
6095	RORA	0.843878043	3.091741704	1.873313375
8501	SLC43A1	5.624653674	20.49311979	1.865303458
100287482	SMKR1	17.15916678	62.42531943	1.863151799
146	ADRA1D	18.06452025	65.54560579	1.859340124
2669	GEM	19.8406817	71.66460015	1.852799057
5155	PDGFB	150.5007729	543.2660412	1.851887973
2247	FGF2	778.0849994	2802.176146	1.848547975
7262	PHLDA2	25.44858431	91.17169861	1.841000652
1280	COL2A1	8.714687716	31.19735662	1.839902921
2149	F2R	234.7157923	839.9177755	1.839333188
10335	MRVI1	3.256561226	11.63075659	1.836523693
9915	ARNT2	1.228823602	4.387731938	1.836197558
54437	SEMA5B	2.530167686	9.032892927	1.835955105
91050	CCDC149	53.83394822	191.8737032	1.833568858
387882	C12orf75	162.9133044	579.8435984	1.831559386
389257	LRRC14B	10.43202648	37.07294831	1.829347417
5271	SERPINB8	10.3158323	36.63224215	1.828253781
57540	DISP3	51.0973581	181.2347263	1.82653881
1466	CSRP2	72.64859475	257.5849917	1.82604174
3433	IFIT2	2.902259942	10.28802375	1.825717232

283576	ZDHHC22	24.90121412	88.05159976	1.82213313
55231	CCDC87	7.581490541	26.77621734	1.820398747
145376	PPP1R36	24.4582452	86.19655809	1.817309364
5327	PLAT	251.314304	885.094109	1.816338073
11219	TREX2	4.973690635	17.49849251	1.81484196
27344	PCSK1N	673.4929878	2369.359606	1.814762348
89797	NAV2	0.801821709	2.819880541	1.814280663
378948	RBMY1B	0.800794422	2.814481798	1.813365489
4499	MT1M	1.167026085	4.100078959	1.812814886
51332	SPTBN5	15.41074561	54.08144364	1.811196999
115701	ALPK2	16.0759239	56.32827441	1.808957623
347731	LRRTM3	28.87894416	100.9975476	1.80623036
56912	IFT46	79.49784975	277.7897877	1.805005819
58504	ARHGAP22	2.866955747	10.01622331	1.804747092
2274	FHL2	6.619621163	23.12485382	1.804623686
2006	ELN	23.42935944	81.69125808	1.80186419
5328	PLAU	239.9491597	836.4809186	1.80160387
221662	RBM24	278.3212079	969.4243858	1.800377529
3755	KCNG1	161.6716333	562.3005723	1.798274948
5992	RFX4	6.274531959	21.80079991	1.796801319
5139	PDE3A	917.7975892	3188.295447	1.796537402
132332	TMEM155	1.181728421	4.104654714	1.796362347
282973	JAKMIP3	7.318373936	25.4148582	1.796077143
54212	SNTG1	11.03291403	38.2761513	1.794631885
7104	TM4SF4	0.812295887	2.815832393	1.793484219
330	BIRC3	72.30743774	250.609924	1.793227586
3207	HOXA11	23.17620771	80.19787081	1.790919415
9829	DNAJC6	0.809186027	2.799664162	1.790710463
8347	HIST1H2BC	26.47083145	91.55357477	1.790212707
84632	AFAP1L2	5.253546303	18.15549327	1.789042608
8787	RGS9	44.28962864	153.0057953	1.788545492
6546	SLC8A1	50.22566543	173.4718429	1.788204833
3202	HOXA5	20.35026396	70.10743427	1.78451993
84419	C15orf48	21.48301816	73.95776153	1.783504867
387104	SOGA3	147.2788041	506.6081539	1.782320481
3887	KRT81	1.554790728	5.346271684	1.781812744
387723	C10orf143	3.869186908	13.29853786	1.781165306
4939	OAS2	5.06495157	17.3794428	1.778761449
79656	BEND5	0.816198998	2.799247555	1.778046235
9645	MICAL2	68.38497393	234.0878566	1.775298831
26030	PLEKHG3	236.2032553	807.483633	1.77340417
389692	MAFA	43.614631	149.0337471	1.772754962
7475	WNT6	180.7295522	616.757249	1.770870338

63950	DMRTA2	51.39734647	175.3158998	1.77019106
51315	KRCC1	85.57768342	291.1003745	1.766210164
8329	HIST1H2AI	12.98854647	44.14459238	1.764996732
4300	MLLT3	5.64792203	19.1419687	1.760947138
6446	SGK1	1.882540481	6.372734335	1.759231631
11077	HSF2BP	20.1110742	67.77328094	1.752726472
203111	ERICH5	26.48678864	89.23991824	1.752416256
9843	HEPH	1.214781472	4.082883344	1.748891538
84465	MEGF11	17.77575767	59.64286959	1.746438617
9568	GABBR2	22.17947737	74.36926014	1.745481049
3437	IFIT3	16.70907042	55.96010313	1.743767147
79884	MAP9	145.608368	486.7872221	1.74119803
55531	ELMOD1	19.03076566	63.60228333	1.740744953
254778	C8orf46	17.50497359	58.46543445	1.739819051
55068	ENOX1	3.199289255	10.68183885	1.739336684
1830	DSG3	19.7869456	65.77997419	1.733099514
11309	SLCO2B1	4.364434918	14.50214326	1.732399352
203430	RTL3	1.143456718	3.796793602	1.731379816
127294	MYOM3	103.2838889	342.7725277	1.730636258
1607	DGKB	15.6978991	52.0046838	1.728070074
9180	OSMR	27.4834052	90.89738048	1.725677953
10083	USH1C	6.602203265	21.82440855	1.724923095
81693	AMN	2.146491224	7.094861375	1.724794222
84066	TEX35	1.146583695	3.785499882	1.723142154
1303	COL12A1	159.817911	526.573035	1.720204543
84779	NAA11	1.224351231	4.029475918	1.718574726
26507	CNNM1	497.033339	1633.428669	1.716488924
4488	MSX2	8.637704244	28.36472497	1.715378051
3959	LGALS3BP	749.820255	2460.522326	1.714347904
55930	MYO5C	148.3548821	486.3215641	1.712858159
715	C1R	6.267147409	20.5316638	1.71196971
84913	ATOH8	18.88417484	61.74752016	1.709203453
10150	MBNL2	447.3325522	1462.670724	1.709185375
776	CACNA1D	2.170161804	7.089016083	1.707782791
8676	STX11	82.01371927	267.8307956	1.707384684
146664	MGAT5B	276.7166464	901.8989685	1.704556396
7164	TPD52L1	183.3432104	596.7569887	1.702596716
857	CAV1	476.8761209	1551.740506	1.702200871
84969	TOX2	77.17292877	250.8729644	1.700790242
644538	SMIM10	13.0608288	42.43260657	1.699926855
6372	CXCL6	0.772944486	2.508965813	1.698656107
8343	HIST1H2BF	7.717338422	25.03947166	1.698028844
83468	GLT8D2	157.7040141	511.1646102	1.696568575

284293	HMSD	1.47741914	4.78236005	1.694643577
23362	PSD3	565.1729419	1825.11624	1.691224049
57381	RHOJ	11.92628814	38.3381325	1.684634965
6695	SPOCK1	2.196552172	7.056586521	1.683729712
27190	IL17B	1.491990223	4.790686409	1.682994298
64127	NOD2	2.514176859	8.069975128	1.682478088
84460	ZMAT1	74.10126295	237.8424348	1.682436101
400954	EML6	15.43653898	49.45127552	1.679658411
8061	FOSL1	54.03171262	172.8115228	1.677321101
84541	KBTBD8	4.173546764	13.31433661	1.673634706
29118	DDX25	14.27446971	45.52621072	1.673260233
257169	C9orf43	2.843295116	9.067198608	1.673093037
3013	HIST1H2AD	2.609484128	8.314768068	1.671911394
83666	PARP9	111.8666258	355.6052561	1.668496961
221935	SDK1	36.26842464	115.1679693	1.666953542
6282	S100A11	796.0943714	2525.279725	1.665431836
26499	PLEK2	7.603490212	24.11693579	1.665312902
157638	FAM84B	69.53940114	220.1453685	1.662553945
55332	DRAM1	170.2074291	537.7484695	1.659637506
55117	SLC6A15	1149.842678	3631.025523	1.658940586
3399	ID3	1100.444812	3474.522511	1.658727933
862	RUNX1T1	275.2048923	868.8199292	1.658551079
8357	HIST1H3H	46.64276606	147.1036971	1.657108253
221061	FAM171A1	142.9006068	450.2001239	1.655554411
11221	DUSP10	0.79412942	2.500547773	1.654798118
117531	TMC1	16.04130096	50.41740978	1.652130851
2920	CXCL2	0.790737265	2.484661837	1.651779194
59269	HIVEP3	31.20690997	97.85822692	1.648827631
102723475	KCNE1B	14.26850752	44.73783925	1.648661141
84456	L3MBTL3	26.51675555	83.00301119	1.64625941
9435	CHST2	12.02467663	37.63712079	1.64615817
60529	ALX4	454.1382591	1418.161617	1.642818467
3233	HOXD4	7.220030445	22.53568109	1.642134227
51289	RXFP3	0.798348083	2.490243747	1.641197153
58489	ABHD17C	6.327321688	19.73147458	1.640831925
6768	ST14	16.64936299	51.74994629	1.636090385
124976	SPNS2	24.69960001	76.75189933	1.635714774
81706	PPP1R14C	91.45583752	283.9830779	1.634657801
3196	TLX2	136.2237422	422.8664474	1.634223923
51776	MAP3K20	275.7319522	855.8529392	1.634096462
627	BDNF	43.92113292	135.8687307	1.629226294
91624	NEXN	287.4654353	885.9684965	1.623866902
3875	KRT18	847.2999824	2593.314703	1.613852546

170689	ADAMTS15	113.5435455	347.1089627	1.61214292
643008	SMIM5	4.882247025	14.90625822	1.610300959
23705	CADM1	1315.948602	4015.349147	1.609422298
7481	WNT11	8.575348965	26.15644119	1.608898977
283417	DPY19L2	46.2309946	140.9465327	1.608215682
100129654	TCF24	28.76417472	87.67111762	1.607828561
25780	RASGRP3	2.362123826	7.192943527	1.606497683
85450	ITPRIP	89.85422149	273.3453806	1.605066804
23600	AMACR	645.6864332	1963.649649	1.60463193
91768	CABLES1	951.4024083	2884.712571	1.600299996
1277	COL1A1	6625.41895	20028.17199	1.59594716
961	CD47	8.976597225	27.05429696	1.591617183
57526	PCDH19	3.794014981	11.41586012	1.589242283
23158	TBC1D9	15.94650022	47.90914676	1.58706129
7462	LAT2	8.694069338	26.03371136	1.58227749
80833	APOL3	2.483629409	7.430696147	1.581047457
166614	DCLK2	47.56832937	142.2307154	1.580159793
2048	EPHB2	289.3917198	865.1523264	1.579930525
28951	TRIB2	232.7126217	695.4802634	1.579460113
2342	FNTB	151.0284909	451.0643766	1.578512618
266727	MDGA1	56.00493597	166.9722356	1.57598234
56833	SLAMF8	3.486621354	10.38027027	1.573942406
151742	PPM1L	23.48905621	69.87883729	1.572866856
55715	DOK4	1428.982275	4248.27282	1.571888396
140876	RIPOR3	528.3809764	1568.681063	1.569901629
79132	DHX58	65.14185982	193.3928753	1.569877831
355	FAS	2.835057639	8.406438771	1.568116693
684	BST2	15.27817509	45.2185861	1.565443652
2878	GPX3	70.61394071	208.99261	1.565426994
4643	MYO1E	33.09601231	97.92207228	1.56497669
286319	TUSC1	50.6553455	149.630336	1.562616272
3167	HMX2	2.504427925	7.39290146	1.561659591
114881	OSBPL7	24.05013411	70.90288412	1.559799374
50651	SLC45A1	128.0541898	377.3315429	1.55907825
64073	C19orf33	22.37634805	65.88705752	1.558020499
57596	BEGAIN	214.1705155	630.5272505	1.557798843
390205	LRRC10B	17.95543088	52.80827317	1.556343692
120939	TMEM52B	3.187490718	9.371402627	1.555843853
53342	IL17D	42.8562199	125.8253342	1.553845921
8029	CUBN	476.2140778	1396.44954	1.552081268
2296	FOXC1	1976.521217	5790.224169	1.550655761
64072	CDH23	390.2190791	1142.218644	1.549482615
362	AQP5	7.57840546	22.16845001	1.548541668

27328	PCDH11X	2.526536752	7.38526783	1.547489076
5988	RFPL1	6.500478478	18.98761399	1.546440806
114795	TMEM132B	3.118933981	9.101758141	1.545092235
219699	UNC5B	85.68429856	250.0106157	1.544886591
10475	TRIM38	4.809331982	14.02916227	1.544520442
3983	ABLIM1	12.25580482	35.72077533	1.543298168
342132	ZNF774	28.49003835	83.0311342	1.543194842
2736	GLI2	243.290282	708.578792	1.542249591
7594	ZNF43	30.18198623	87.8783151	1.541819459
89796	NAV1	123.5650273	359.6599718	1.54136313
55106	SLFN12	25.95112424	75.5165648	1.540996099
7205	TRIP6	19.68193542	57.10397582	1.536719101
23220	DTX4	31.80442797	92.25280006	1.536365062
100130705	LOC100130705	22.98308243	66.64222067	1.535864174
2770	GNAI1	15.93479221	46.17892277	1.535054312
59	ACTA2	8.562022212	24.80672529	1.534707816
727832	GOLGA6L6	6.103257696	17.6717142	1.53379058
729920	ISPD	34.47560923	99.74949747	1.532733527
4137	MAPT	837.066171	2412.322102	1.527008975
133	ADM	60.39057117	174.0143735	1.526811254
55089	SLC38A4	226.7069308	653.181386	1.526655181
360	AQP3	14.59611119	42.00492063	1.524974294
55970	GNG12	145.9596545	419.9439044	1.524626986
131583	FAM43A	10.5133554	30.16222594	1.520519714
10257	ABCC4	854.9501301	2452.026866	1.520062612
8365	HIST1H4H	6.839880814	19.57939977	1.517293446
6840	SVIL	792.9626756	2268.985747	1.516722681
2621	GAS6	358.2508776	1024.993491	1.516572604
8839	WISP2	16.38994115	46.79906272	1.513668962
3918	LAMC2	8.839078488	25.20953283	1.512001508
339977	LRRC66	6.1778241	17.61542113	1.511668267
23678	SGK3	108.3925316	308.8163867	1.510483952
3958	LGALS3	77.30734872	220.2517339	1.51047591
118881	COMTD1	180.8074785	514.7275824	1.509354742
4855	NOTCH4	46.19679998	131.444272	1.508586449
23231	SEL1L3	489.1215064	1391.3207	1.508190195
84333	PCGF5	177.4439827	504.6013957	1.507780544
85444	LRRCC1	32.79684082	93.22332024	1.507134044
79690	GAL3ST4	101.6331281	288.5205476	1.505303331
1956	EGFR	160.4322813	455.389803	1.505137525
124936	CYB5D2	162.6148422	461.2668935	1.504142808
8970	HIST1H2BJ	16.5600837	46.94663859	1.503311899
3680	ITGA9	11.18699984	31.49522993	1.493310162

55520	ELAC1	21.32363752	60.01249303	1.4928093
57125	PLXDC1	99.12263254	278.7719537	1.491799015
5376	PMP22	391.9816226	1101.06003	1.490035204
94032	CAMK2N2	18.32951247	51.41621875	1.488055102
26095	PTPN20	81.96470282	229.144939	1.483185754
151636	DTX3L	218.9936228	611.7590472	1.482074674
1829	DSG2	593.2496353	1653.096992	1.47846016
339145	FAM92B	27.43003913	76.41081226	1.478020117
10566	AKAP3	4.459439098	12.40436183	1.475913348
51375	SNX7	826.8753363	2297.800929	1.474512072
79819	WDR78	14.53877994	40.3966844	1.474330681
22998	LIMCH1	50.887746	141.0269654	1.470580847
1803	DPP4	7.114689904	19.67346843	1.467378544
340562	SATL1	3.077775275	8.496816973	1.465034592
10202	DHRS2	28.34055804	78.13179502	1.463041594
79642	ARSJ	439.7091492	1209.257026	1.459499463
115584	SLC5A11	3.086285519	8.459625594	1.454722279
56147	PCDHA1	52.46543124	143.7607668	1.45423094
8022	LHX3	3.096377972	8.481971551	1.453818054
65983	GRAMD2B	96.34314913	263.8241554	1.453322675
55509	BATF3	49.00286025	134.1277545	1.452669934
8321	FZD1	94.0812355	257.315763	1.451560927
100170229	SRRM5	3.798210582	10.38271529	1.450791988
399979	SNX19	174.3870017	476.6862181	1.450747405
83648	FAM167A	3.102990216	8.476713184	1.449845825
3234	HOXD8	63.78560541	174.2084902	1.449512146
2334	AFF2	694.8553116	1890.492779	1.443977834
2706	GJB2	4.450468191	12.09691682	1.442610369
8407	TAGLN2	922.1171303	2502.001385	1.440060665
7399	USH2A	622.207725	1687.002624	1.438994006
9540	TP53I3	472.1109474	1279.259562	1.438111175
57562	CEP126	106.1214975	287.5021792	1.437855953
139411	PTCHD1	58.28565648	157.7319481	1.436262104
219348	PLAC9	8.478791755	22.9278834	1.43517258
57631	LRCH2	187.2115988	505.2993319	1.432468452
11278	KLF12	4.205387356	11.34671078	1.431963548
114827	FHAD1	9.852061979	26.49737752	1.427351972
80217	CFAP43	9.156055522	24.61109234	1.426510575
84674	CARD6	6.121034066	16.38507698	1.420535149
2113	ETS1	726.4797468	1943.3063	1.41951883
131368	ZPLD1	4.166173944	11.08853087	1.412273253
10391	CORO2B	4.613306481	12.27594307	1.411960816
85315	PAQR8	154.4204065	410.8965448	1.411911783

5205	ATP8B1	42.87662738	114.0620668	1.411555745
9289	ADGRG1	177.9460367	472.2839161	1.408214603
5621	PRNP	444.8396817	1178.148301	1.405163759
11346	SYNPO	399.2618597	1055.656481	1.402733279
3176	HNMT	6.799941343	17.96351547	1.401475507
4060	LUM	15.89521357	41.97101314	1.400800888
4916	NTRK3	66.98907596	176.8671496	1.400668357
2022	ENG	245.7122347	646.9781474	1.396747288
5029	P2RY2	26.64725109	69.96592159	1.392665684
150	ADRA2A	79.83984597	209.4094004	1.391145364
408	ARRB1	1074.529434	2818.321401	1.391131143
23780	APOL2	180.5964209	473.193031	1.389659525
1281	COL3A1	58240.7918	152583.3422	1.389495595
1016	CDH18	7.868651249	20.61079179	1.389211656
4880	NPPC	10.78847852	28.2432187	1.388413095
6404	SELPLG	5.394709631	14.10886196	1.386984409
4995	OR3A2	28.43990323	74.22708921	1.384029239
80258	EFHC2	15.87607078	41.41369019	1.383253861
57348	TTYH1	310.8180253	810.2941757	1.382375598
9734	HDAC9	3990.361922	10398.1804	1.381739582
9118	INA	1161.681188	3022.43373	1.379496517
2747	GLUD2	5.781201395	15.03853522	1.379222817
55349	CHDH	12.87405477	33.43599849	1.37693569
9469	CHST3	628.5671398	1630.939728	1.37556471
1906	EDN1	5.068605028	13.11683774	1.3717593
9542	NRG2	167.2808946	432.5718454	1.370667086
3709	ITPR2	502.6587609	1298.819576	1.369549797
7318	UBA7	4.059442348	10.475041	1.367602429
4884	NPTX1	668.8672141	1723.346569	1.365421125
9806	SPOCK2	8.424610244	21.69473678	1.364663234
50632	CALY	657.6628323	1692.840072	1.36402564
644	BLVRA	311.6641548	800.8856662	1.361604063
141	ADPRH	182.4309405	468.22962	1.359865769
84163	GTF2IRD2	64.32339176	164.983812	1.358909089
10892	MALT1	214.3929478	549.428776	1.357675022
3206	HOXA10	160.0202189	409.1863127	1.354503683
51700	CYB5R2	18.54352094	47.33554739	1.352008805
50509	COL5A3	68.42534764	174.5523626	1.35105712
2100	ESR2	6.058403817	15.45072441	1.350664833
441430	ANKRD20A2	5.594957761	14.26586458	1.35036804
2329	FMO4	5.784213753	14.73562452	1.349115433
25858	CATSPERZ	5.408083539	13.76570908	1.347889583
79815	NIPAL2	83.26993886	211.9218391	1.347664599

282969	FUOM	13.90579393	35.3832917	1.347382152
414152	C10orf105	24.93409975	63.43652496	1.347193627
83445	GSG1	42.22606926	107.386075	1.346601067
6737	TRIM21	42.63085858	108.217918	1.343969374
54149	C21orf91	11.14746124	28.24484806	1.341272556
58191	CXCL16	304.833047	771.7071388	1.340034138
114821	ZBED9	65.38548986	165.3990379	1.338908425
6583	SLC22A4	18.5120225	46.68282278	1.334429276
9464	HAND2	328.6910897	827.830402	1.332602885
51473	DCDC2	11.85727688	29.8021582	1.32964409
84074	QRICH2	60.93092473	153.1379149	1.329584978
59336	PRDM13	8.792729341	21.96871055	1.321067228
115708	TRMT61A	169.0613523	422.3110471	1.320759092
170692	ADAMTS18	199.4617928	498.177166	1.320546474
90139	TSPAN18	165.6049352	413.5111064	1.320180412
467	ATF3	140.7322131	351.2368719	1.319491709
3675	ITGA3	447.9266842	1116.936067	1.31821209
64067	NPAS3	566.9611734	1413.099985	1.317541703
8372	HYAL3	25.02047579	62.32115961	1.316612854
81606	LBH	22.94960919	57.13713963	1.315959229
25840	METTL7A	103.7144475	258.085588	1.315232704
51226	COPZ2	17.83669145	44.35461323	1.314236131
9659	PDE4DIP	341.5121697	849.2110079	1.314186083
8358	HIST1H3B	18.40657434	45.76677604	1.314079517
80830	APOL6	68.0245111	168.9670529	1.312615373
387885	CFAP73	18.15947016	45.05212383	1.310873009
8382	NME5	7.417295276	18.39424211	1.310289128
11247	NXPH4	24.92186886	61.80085103	1.310214446
1050	CEBPA	86.32961724	213.8442018	1.308632593
817	CAMK2D	1542.980214	3820.728962	1.308128356
11182	SLC2A6	152.2539082	376.1215502	1.304719708
2914	GRM4	6.771721973	16.70736506	1.302889576
80216	ALPK1	110.9139779	273.3249615	1.301176029
389432	SAMD5	73.75287039	181.1634875	1.296521113
4644	MYO5A	437.6713238	1074.397542	1.295608143
122773	KLHDC1	36.33203376	89.08428422	1.293928816
401027	C2orf66	24.35496709	59.68788336	1.29322206
388115	CCDC9B	30.76245864	75.24830005	1.290488177
3201	HOXA4	86.53887446	211.6489498	1.290253068
6752	SSTR2	24.27780492	59.21078814	1.286222071
284086	NEK8	81.73397561	199.3121734	1.286022013
10855	HPSE	76.02376258	185.1509564	1.284179668
4211	MEIS1	12.14441743	29.53353111	1.282060575

2257	FGF12	500.3189124	1216.392617	1.281689072
257106	ARHGAP30	7.426619791	18.01711047	1.278590028
117245	HRASLS5	170.3153652	413.0888632	1.278243571
1415	CRYBB2	16.90972947	41.00894195	1.278084943
83844	USP26	78.33918739	189.8132782	1.27677485
57493	HEG1	210.7062072	510.4321206	1.276486303
11167	FSTL1	1189.486843	2878.866758	1.275161705
84978	FRMD5	22.56187865	54.54483601	1.273555416
139716	GAB3	6.565865439	15.87127115	1.273360591
10610	ST6GALNAC2	168.3376405	406.8640585	1.273189041
401494	HACD4	12.50905054	30.23325085	1.273163824
163175	LGI4	10.75093711	25.97644356	1.272741506
154214	RNF217	66.47178896	160.4843699	1.271618709
2537	IFI6	877.9407228	2118.684324	1.270973208
9498	SLC4A8	98.70889437	238.1119388	1.270387965
283212	KLHL35	54.908089	132.3800104	1.269594683
1847	DUSP5	65.12058908	156.9271548	1.268909364
198437	LKAAEAR1	36.0963684	86.96276954	1.26854419
8819	SAP30	286.9498989	691.2913761	1.268495062
84875	PARP10	59.26659957	142.6235728	1.26692126
284098	PIGW	133.545301	321.2320519	1.266286635
1767	DNAH5	63.14508283	151.8443719	1.265851137
10763	NES	2293.121901	5512.801478	1.265472599
10178	TENM1	7.776609484	18.67986533	1.264270856
161247	FITM1	6.035190598	14.49039644	1.263625828
56937	PMEPA1	11.86123401	28.45310128	1.262331798
22801	ITGA11	81.21789286	194.632091	1.260880099
363	AQP6	16.21589154	38.74642493	1.256654857
5971	RELB	161.189809	384.5514676	1.254416164
91010	FMNL3	750.2963522	1788.486777	1.253207002
1995	ELAVL3	299.432259	713.1514189	1.25197877
375057	STUM	46.61111265	110.99667	1.251770538
3215	HOXB5	112.4642589	267.6896133	1.251094575
8991	SELENBP1	42.73656752	101.6631951	1.250254533
23732	FRRS1L	264.7734862	629.0870058	1.248500901
1026	CDKN1A	304.6815223	722.4796458	1.245654934
56134	PCDHAC2	115.1765081	272.7054059	1.24349681
375519	GJB7	122.2314973	289.2713918	1.242807557
5307	PITX1	89.79458354	212.4324107	1.242303566
101928841	LOC101928841	24.90550637	58.88119259	1.241342148
51440	HPCAL4	93.62434815	221.3083952	1.241102506
5954	RCN1	2798.294293	6605.93128	1.239214265
647310	TEX22	72.54568786	171.2489948	1.239133752

9379	NRXN2	65.60558398	154.8350809	1.23884186
153769	SH3RF2	230.5952837	543.777137	1.237652488
203190	LGI3	6.40380728	15.09056908	1.236645416
64167	ERAP2	19.54253933	46.00634526	1.235214912
3693	ITGB5	164.2206517	386.4438026	1.234623064
50801	KCNK4	23.19743371	54.58619275	1.234570865
147646	C19orf84	20.54231544	48.30422358	1.233550535
7409	VAV1	8.108591254	19.05332187	1.232519353
954	ENTPD2	579.6067006	1361.281479	1.231819232
341019	DCDC1	32.64257983	76.61498619	1.230871535
128434	VSTM2L	509.2066975	1190.222959	1.224908552
163782	KANK4	320.9359922	748.3283167	1.221385774
56521	DNAJC12	17.59251315	40.92590934	1.218052882
340526	RTL5	653.1423147	1519.137353	1.217783034
6495	SIX1	74.29670925	172.6683653	1.216633572
11103	KRR1	679.1163383	1576.860767	1.215324633
91608	RASL10B	211.9949414	491.8561913	1.214206723
83849	SYT15	62.23381213	144.2971396	1.213272175
51065	RPS27L	296.4204756	685.1172889	1.208705885
124857	WFIKKN2	7.724723785	17.78660887	1.203236226
3572	IL6ST	2971.904155	6841.846424	1.202998132
6595	SMARCA2	81.07095394	186.5585455	1.202371421
89801	PPP1R3F	53.95157528	124.1434909	1.202271631
100506564	THEGL	9.413712026	21.65682316	1.201986004
160857	CCDC122	59.89037492	137.6414458	1.200518883
25805	BAMBI	42.69938871	98.01628279	1.198806018
80854	SETD7	178.1926796	408.6706346	1.197500511
112849	L3HYPDH	116.8232757	267.9171205	1.197459032
5409	PNMT	6.730009299	15.4274919	1.196823134
85301	COL27A1	38.04428324	87.20248187	1.196689514
29970	SCHIP1	639.8804678	1466.156988	1.196165253
286749	STON1-GTF2A1L	54.96234385	125.8778837	1.195509395
9249	DHRS3	13.57412414	31.01502794	1.192108312
164633	CABP7	13.41113102	30.62023226	1.191054315
50507	NOX4	14.1175539	32.23277472	1.191038247
9241	NOG	178.3683618	406.9330274	1.189931638
91461	PKDCC	155.8286444	355.418718	1.189559209
143941	TTC36	15.75739013	35.90983072	1.188350248
84532	ACSS1	85.15067773	193.9721704	1.187759763
53637	S1PR5	698.7502999	1591.538269	1.187572945
79922	MRM1	89.14624386	202.7081815	1.185158402
7128	TNFAIP3	30.54680356	69.4493472	1.18493971
8323	FZD6	224.3775713	509.5956568	1.18342451

494143	CHAC2	36.97833995	83.86702955	1.181423299
197335	WDR90	39.66572871	89.79516338	1.178744686
101928601	MEI4	10.76660762	24.33514803	1.176477798
83853	ROPN1L	58.54899872	132.0850376	1.173750646
2812	GP1BB	196.3139552	442.7533265	1.173340414
4013	VWA5A	15.46735711	34.87066974	1.172787367
54505	DHX29	1208.63669	2723.733698	1.172205014
8745	ADAM23	1087.912443	2444.619754	1.168047629
3977	LIFR	20.81714507	46.75105037	1.167226553
245806	VGLL2	39.72408896	89.15459817	1.166295074
5731	PTGER1	276.469798	620.4291281	1.166144533
6441	SFTPD	21.51918745	48.2877114	1.166032485
56112	PCDHGA3	527.1108052	1182.621573	1.165810329
284723	SLC25A34	16.53942467	37.09681208	1.165386163
51764	GNG13	28.58684114	64.11163381	1.165234964
3897	L1CAM	2329.202557	5222.735568	1.164969553
8814	CDKL1	56.65520958	126.9784039	1.164302622
255231	MCOLN2	13.07815254	29.29683453	1.163586036
7043	TGFB3	142.3744689	318.5566117	1.161859321
23283	CSTF2T	102.95372	230.125774	1.160426614
23641	LDOC1	171.4243653	382.9991136	1.159768872
200558	APLF	54.98167581	122.7981932	1.159266549
51316	PLAC8	106.7210762	238.2306781	1.158514087
84698	CAPS2	88.1229706	196.4739896	1.156748299
4753	NELL2	4386.063647	9772.056771	1.155735498
54898	ELOVL2	27.18294503	60.5292614	1.154930979
7306	TYRP1	750.0405039	1668.607632	1.153604337
3669	ISG20	18.47570912	41.07099574	1.152490187
9423	NTN1	413.3343756	914.940409	1.14636843
56896	DPYSL5	243.304569	538.0763578	1.145047506
4482	MSRA	28.57315088	63.16689352	1.144508487
57216	VANGL2	118.8793744	262.6937617	1.143883512
2762	GMDS	702.7681573	1552.599567	1.143565061
728392	LOC728392	277.1971157	611.853635	1.142274333
7168	TPM1	3318.892634	7313.979117	1.139954548
8969	HIST1H2AG	85.79556895	189.0679794	1.139930004
122060	SLAIN1	51.0898684	112.5033841	1.138859273
6376	CX3CL1	19.77545369	43.49179774	1.137032551
1583	CYP11A1	13.12040249	28.8457393	1.136546262
115416	MALSU1	858.9555808	1886.664643	1.135182573
9263	STK17A	269.4281062	591.42556	1.134296237
130752	MDH1B	16.07038081	35.26913771	1.134002188
10325	RRAGB	112.3654788	246.5715793	1.133807644

8612	PLPP2	33.91859488	74.28567048	1.13100754
59284	CACNG7	975.1745478	2135.341497	1.130734436
283948	NHLRC4	35.3211114	77.30815884	1.13008994
9107	MTMR6	344.8298098	754.4348654	1.129511853
255374	MBLAC1	44.69447442	97.69777523	1.128229228
948	CD36	19.11939393	41.7913102	1.128166198
4647	MYO7A	122.6650994	268.0584411	1.127822733
283130	SLC25A45	123.0540473	268.140006	1.123694374
9881	TRANK1	82.45143929	179.5871588	1.123067612
4862	NPAS2	9.802723421	21.33423389	1.121915781
6536	SLC6A9	126.0683267	274.2966875	1.121531339
2116	ETV2	8.65229554	18.82398777	1.121417439
5805	PTS	131.0699858	285.0103789	1.120677103
8788	DLK1	180.4360256	392.3449225	1.120635116
8828	NRP2	63.85639509	138.5219457	1.117211542
162394	SLFN5	40.63817629	87.9262891	1.113458921
55506	H2AFY2	282.7933233	611.7978729	1.113307032
1565	CYP2D6	10.69130262	23.09416244	1.111090584
51296	SLC15A3	11.43455327	24.6865046	1.110322575
93183	PIGM	167.1692276	360.8035561	1.109904256
23466	CBX6	4073.219271	8781.617713	1.108317251
7771	ZNF112	71.28483866	153.5575176	1.107111971
29103	DNAJC15	762.8165302	1642.7697	1.106722231
57514	ARHGAP31	98.66855525	212.482628	1.106682606
143689	PIWIL4	22.4657336	48.3701147	1.106389786
54855	FAM46C	21.21129397	45.6474383	1.105701266
91978	TPGS1	149.141192	320.8627754	1.105277649
115811	IQCD	151.2783157	325.3472767	1.104775272
60598	KCNK15	120.4307053	258.9049388	1.104219215
55638	SYBU	114.9526471	247.0693352	1.103876274
27124	INPP5J	178.4346672	383.345048	1.103247607
23371	TNS2	350.1021428	751.2884277	1.101590988
5288	PIK3C2G	27.18303504	58.31889027	1.101256722
1130	LYST	1093.348253	2342.658616	1.099393732
100528064	NEDD8-MDP1	84.53703421	181.1081132	1.099195772
242	ALOX12B	29.23592444	62.57680796	1.097885859
440689	HIST2H2BF	10.13578789	21.68738127	1.097397621
6352	CCL5	35.86782898	76.73021306	1.097104337
10343	PKDREJ	10.03672297	21.46986653	1.097024922
9638	FEZ1	199.4317066	426.4730578	1.096559809
116443	GRIN3A	9.696298295	20.7260597	1.09593988
10929	SRSF8	64.96839745	138.6747364	1.093894959
10148	EBI3	300.3588533	641.0929282	1.093846306

6094	ROM1	13.40861682	28.61197641	1.093458735
91807	MYLK3	192.0580629	409.7043248	1.09304059
9454	HOMER3	256.0037663	546.1100584	1.093026694
84152	PPP1R1B	30.54715691	65.13895796	1.092482529
55924	FAM212B	57.76931055	122.9982098	1.090262136
284307	ZIK1	200.4505973	426.7698041	1.090211386
151963	MB21D2	286.3580636	608.3715927	1.08713256
9331	B4GALT6	300.7241817	638.8770453	1.087097423
7552	ZNF711	1261.926115	2680.164013	1.086693845
2171	FABP5	142.2839975	302.1539417	1.086510349
54972	TMEM132A	2159.121963	4585.037822	1.086488895
23624	CBLC	31.89599814	67.71144854	1.086024357
9884	LRRC37A	45.40761759	96.2875933	1.084415574
11013	TMSB15A	9.023303058	19.10238103	1.082024929
5721	PSME2	1178.210143	2493.475891	1.081561376
59283	CACNG8	210.6015922	445.5428423	1.081047821
7957	EPM2A	38.62786256	81.66843894	1.080136802
79739	TTLL7	190.5259248	402.707418	1.079744729
83698	CALN1	145.1413047	306.4459293	1.078174397
3363	HTR7	18.44819692	38.90017836	1.076296952
5979	RET	37.01027291	77.95015831	1.074626179
6337	SCNN1A	10.66108138	22.45174398	1.074473731
220323	OAF	21.14114203	44.43588312	1.071671848
8336	HIST1H2AM	18.05910319	37.89971347	1.069460691
23237	ARC	13.36095395	28.03678191	1.069297747
101927322	LOC101927322	20.42086732	42.81536714	1.068084555
6890	TAP1	470.6200611	986.5404154	1.067815335
83595	SOX7	39.92987524	83.65278881	1.066945073
10516	FBLN5	13.36528169	27.99781241	1.066823863
5272	SERPINB9	437.4066646	915.5236069	1.065621884
8871	SYNJ2	88.91402858	185.8928341	1.063988191
10922	FASTK	898.071437	1874.838791	1.061864437
2891	GRIA2	128.5752626	268.1391543	1.0603688
158135	TTLL11	26.5815891	55.26515378	1.055942753
57605	PITPNM2	145.0000286	301.2425145	1.054872208
3725	JUN	793.0360031	1647.342431	1.054682207
7041	TGFB1I1	643.8061827	1335.44071	1.052617589
51363	CHST15	71.67827497	148.6161469	1.051983048
825	CAPN3	22.87360203	47.39948342	1.051187763
4920	ROR2	346.8143307	718.0143327	1.04984913
9725	TMEM63A	329.7807996	682.6273043	1.049590717
59271	EVA1C	115.6975785	239.4436947	1.049327775
81831	NETO2	1178.840327	2439.579	1.049263882

130132	RFTN2	79.9959488	165.5021535	1.048851144
10625	IVNS1ABP	1738.947631	3594.339091	1.047512034
6421	SFPQ	2896.102638	5984.387407	1.047090841
3800	KIF5C	12.11229473	25.01872602	1.046536112
161436	EML5	13.3867615	27.63835447	1.045864734
132430	PABPC4L	64.40647631	132.8401549	1.044413641
55604	CARMIL1	15.13282169	31.20222255	1.043967777
163702	IFNLR1	94.51073608	194.8329695	1.043687701
10587	TXNRD2	521.9271733	1072.577039	1.039160854
8935	SKAP2	403.1998125	828.3390634	1.038726458
284695	ZNF326	317.1942223	651.5127928	1.038427015
133383	SETD9	123.2843786	253.1724413	1.038130364
6509	SLC1A4	133.4772255	273.906577	1.037090305
22807	IKZF2	66.76271844	136.8431909	1.035409046
25797	QPCT	14.33337391	29.36276369	1.034609521
79962	DNAJC22	144.2496221	295.3953694	1.034079671
169611	OLFML2A	1107.846234	2266.640544	1.032797966
83982	IFI27L2	367.7275858	751.2006003	1.030560809
1187	CLCNKA	39.22710358	80.05575849	1.029154366
216	ALDH1A1	13078.14877	26689.29367	1.029102785
10628	TXNIP	8048.609174	16425.03808	1.029085308
4493	MT1E	119.9752267	244.6545816	1.028009761
9830	TRIM14	432.4692647	881.5491176	1.02744335
51635	DHRS7	338.6796753	689.7246885	1.026099198
25789	TMEM59L	844.5550905	1719.471801	1.025702018
166929	SGMS2	90.66493668	184.4795625	1.024844374
28	ABO	39.20419431	79.73369622	1.024181539
91947	ARRDC4	315.3647672	641.3188606	1.024020346
56660	KCNK12	24.77651392	50.36417567	1.023424688
9159	PCSK7	235.2873016	478.2606048	1.023373498
51196	PLCE1	187.7591025	381.3184515	1.022113492
146691	TOM1L2	3990.092741	8101.105113	1.021696448
3663	IRF5	125.7462132	255.2277428	1.021270202
10808	HSPH1	1168.692313	2372.022265	1.021222396
57571	CARNS1	161.3544714	327.3559709	1.020626736
221	ALDH3B1	340.7750715	691.3595706	1.020616437
6330	SCN4B	12.69724406	25.75414703	1.020289366
100289187	TMEM225B	16.09211015	32.61988397	1.019398132
169834	ZNF883	27.49487479	55.70786074	1.018718199
153129	SLC38A9	573.0846343	1161.071394	1.018636566
374882	TMEM205	1031.182231	2088.387661	1.01809023
9570	GOSR2	454.7546405	919.2626285	1.015388731
651746	ANKRD33B	3516.444798	7107.830016	1.01529161

102724951	LOC102724951	16.31709518	32.97303336	1.014902367
102724219	LOC102724219	16.31709518	32.97303336	1.014902367
102724843	LOC102724843	16.31709518	32.97303336	1.014902367
4054	LTBP3	1751.166831	3538.492802	1.014818451
26519	TIMM10	125.4403318	253.3603807	1.01418966
4067	LYN	184.8791481	372.6707367	1.011319021
51762	RAB8B	847.7501563	1708.600164	1.011103775
257019	FRMD3	308.9017002	622.2822521	1.010421289
285600	KIAA0825	19.48099043	39.2397846	1.010250095
258010	SVIP	12.0664062	24.30141147	1.010044055
54937	SOHLH2	261.2325884	525.7477159	1.009035795
8706	B3GALNT1	84.34983849	169.7394757	1.008864915
7779	SLC30A1	743.4312326	1494.984619	1.007859438
79762	C1orf115	43.91953566	88.26437671	1.006968486
64838	FNDC4	169.1604093	339.8551308	1.006527947
2752	GLUL	26.18085623	52.57869202	1.005965973
6785	ELOVL4	237.1023379	475.9032573	1.005158441
9928	KIF14	439.2797829	219.2983667	-1.002245036
9514	GAL3ST1	77.3203139	38.5409836	-1.004454104
10493	VAT1	7392.45354	3684.140089	-1.00472535
55972	SLC25A40	420.5073169	209.4294682	-1.00566645
79816	TLE6	660.9511638	328.6108695	-1.008163477
1592	CYP26A1	1088.72	541.2002214	-1.008398632
4948	OCA2	121.6909019	60.45875926	-1.009198033
55314	TMEM144	566.9848181	281.4802913	-1.010276194
284254	DYNAP	34.33152179	17.03429024	-1.011091971
9754	STARD8	253.6524618	125.8182181	-1.011512327
401251	SAPCD1	61.56798643	30.51549408	-1.012638437
81578	COL21A1	30.36270929	15.04803064	-1.012725838
8987	STBD1	504.8001199	249.9915872	-1.013832707
644596	SMIM10L2B	79.71760132	39.44271424	-1.015139467
132720	FAM241A	33.71178149	16.67102683	-1.015909901
65010	SLC26A6	703.7332718	347.6567718	-1.017365032
26084	ARHGEF26	1805.30209	891.8438178	-1.017377282
1745	DLX1	1390.842754	685.4466745	-1.020842982
84900	RNFT2	75.33676604	37.12116139	-1.021112256
4286	MITF	1528.213653	752.1144828	-1.022822072
1718	DHCR24	2214.599801	1089.125356	-1.023875999
6303	SAT1	2009.403729	987.9284721	-1.024288962
59341	TRPV4	38.04223433	18.69279351	-1.025119796
10365	KLF2	1926.652399	946.0102079	-1.026168652
84561	SLC12A8	21.95916535	10.77811372	-1.026718505
23593	HEBP2	424.5379759	208.3541195	-1.026855988

26037	SIPA1L1	5748.354459	2821.013546	-1.026935432
5309	PITX3	60.89881598	29.88235623	-1.02712027
6920	TCEA3	3039.199333	1491.001879	-1.027409225
23338	JADE2	3548.739785	1740.8334	-1.027528649
4671	NAIP	388.7622695	190.6576509	-1.027903782
401	PHOX2A	41.41612676	20.30140826	-1.028612831
2596	GAP43	6419.739239	3142.007032	-1.030828289
10841	FTCD	30.23808931	14.79579321	-1.031179939
8971	H1FX	8184.484634	4003.262855	-1.031715228
8724	SNX3	12281.76888	6000.838901	-1.033282254
22843	PPM1E	270.443371	132.0763522	-1.033954355
7644	ZNF91	924.8629916	451.5951049	-1.034209814
6619	SNAPC3	1229.95532	599.3242668	-1.037197213
144132	DNHD1	328.4285743	160.0176665	-1.037348459
340485	ACER2	43.08133719	20.9834549	-1.037810794
10409	BASP1	139.0617841	67.64504505	-1.039669837
10420	TESK2	851.1590475	413.3754386	-1.041976068
85442	KNDC1	167.5631019	81.33030385	-1.042839588
79144	PPDPF	26000.96517	12612.30804	-1.043732867
4237	MFAP2	295.1910727	142.8523591	-1.04712423
221120	ALKBH3	165.5662687	80.09816843	-1.04756762
119	ADD2	33.97960091	16.43728289	-1.04769707
340371	NRBP2	1924.602508	930.2397502	-1.048886019
4664	NAB1	165.044899	79.61950673	-1.051664712
5837	PYGM	1450.411227	699.0807826	-1.052930916
5864	RAB3A	267.9451597	129.1170775	-1.053257925
8447	DOC2B	52.07084541	25.09092298	-1.053310288
65266	WNK4	349.8931152	168.3005666	-1.055874244
1789	DNMT3B	1497.208598	720.0288825	-1.056148555
118	ADD1	5101.832057	2447.453374	-1.059734034
11004	KIF2C	2116.079512	1014.845247	-1.06013409
604	BCL6	102.8334042	49.31509595	-1.060207737
64946	CENPH	603.2609586	289.2218035	-1.060605902
2731	GLDC	569.3941494	272.9317319	-1.060887533
286336	FAM78A	332.8347532	159.5072752	-1.061183854
27293	SMPDL3B	588.8138485	282.1656539	-1.061269214
4615	MYD88	65.84034293	31.55040616	-1.061313272
23038	WDTC1	3263.148977	1562.312456	-1.062581839
2028	ENPEP	19.86705949	9.50921183	-1.062980682
84695	LOXL3	510.2449476	244.0935829	-1.063755624
55814	BDP1	1491.941654	713.1399483	-1.064933989
2035	EPB41	2139.788963	1022.398306	-1.065511166
113457	TUBA3D	21.26117715	10.14449988	-1.067523733

50863	NTM	216.0173541	102.9955358	-1.068565411
6331	SCN5A	79.55757779	37.92947101	-1.068680101
4897	NRCAM	32.35013032	15.42237893	-1.068746203
57654	UVSSA	215.2539751	102.6050475	-1.068938177
146212	KCTD19	71.26114696	33.93888349	-1.0701766
51027	BOLA1	62.1523924	29.59789469	-1.070315366
1958	EGR1	788.9593605	375.0522517	-1.072859385
221301	CALHM4	45.75513734	21.7342112	-1.073966
23216	TBC1D1	373.9389649	177.4073152	-1.075737311
9509	ADAMTS2	153.9481736	73.02043591	-1.076072565
389421	LIN28B	2948.437777	1398.060784	-1.076523661
2769	GNA15	43.04939714	20.39850487	-1.077529622
55840	EAF2	17.898156	8.479989833	-1.077676518
57715	SEMA4G	861.0553978	407.837034	-1.078113273
79629	OCEL1	922.0548637	436.4284889	-1.079107316
100129543	ZNF730	140.0363352	66.27960155	-1.079164377
4131	MAP1B	1696.778577	802.6624914	-1.079932924
3600	IL15	463.7599442	219.238506	-1.080877009
115330	GPR146	63.90265723	30.20510699	-1.081083427
8092	ALX1	221.4478109	104.6390636	-1.0815452
10461	MERTK	908.2889694	428.8439054	-1.082698742
10227	MFSD10	1158.233808	546.3023738	-1.084154916
10434	LYPLA1	5475.342844	2579.681585	-1.085756301
196385	DNAH10	68.39650554	32.21044413	-1.086394065
79925	SPEF2	64.25499646	30.25206077	-1.08677522
1746	DLX2	1082.236643	508.9613336	-1.088388033
92610	TIFA	78.60045154	36.92215054	-1.090051015
1821	DRP2	35.00349499	16.41153236	-1.092789027
4902	NRTN	293.4344558	137.5583919	-1.09299413
6002	RGS12	474.530733	222.2521443	-1.094304188
23533	PIK3R5	31.63268502	14.80872112	-1.094968964
2065	ERBB3	306.9320374	143.6144029	-1.095718799
56123	PCDHB13	96.04866953	44.88698486	-1.097468439
55502	HES6	368.3592872	172.0219579	-1.098520886
10675	CSPG5	929.5923253	433.9385441	-1.09910742
80303	EFHD1	345.6444157	161.1113521	-1.101230465
202915	TMEM184A	231.123756	107.6895203	-1.101787692
64403	CDH24	2175.550853	1013.417156	-1.102152582
389816	LRRC26	403.3530523	187.8658791	-1.10234011
57167	SALL4	3115.20559	1450.73988	-1.102538513
226	ALDOA	43471.54964	20229.9738	-1.103577073
9759	HDAC4	354.5503252	164.9032046	-1.104370984
57639	CCDC146	113.9303472	52.9878847	-1.104417643

239	ALOX12	21.22678132	9.86619806	-1.105319474
1043	CD52	17.55855474	8.158562508	-1.105787215
7525	YES1	11099.61951	5152.267926	-1.1072307
8673	VAMP8	32.3693079	15.01865716	-1.107870694
55765	C1orf106	92.99538917	43.13998859	-1.108133392
100130771	EFCAB10	66.06962473	30.64766009	-1.10821022
2825	GPR1	884.7776061	410.4175359	-1.108222497
79041	TMEM38A	572.5267859	265.5335072	-1.108449268
23327	NEDD4L	31.94356296	14.81037075	-1.108917482
26355	FAM162A	1379.882931	639.1698599	-1.110274589
347688	TUBB8	150.8008339	69.81014974	-1.111135696
160622	GRASP	238.4225211	110.3248941	-1.111762155
283659	PRTG	2986.532369	1381.842662	-1.111878
27019	DNAI1	283.3716075	131.0947348	-1.112085471
333929	SNAI3	31.27245742	14.45087207	-1.113736034
23022	PALLD	267.7624149	123.6406284	-1.114800575
22902	RUFY3	1434.684291	662.1772499	-1.115443949
2125	EVPL	134.1143294	61.85279468	-1.116552703
81563	C1orf21	2457.503506	1131.874163	-1.118479901
84667	HES7	1790.710045	823.8974851	-1.119995009
1028	CDKN1C	417.72089	192.1902098	-1.120004447
1382	CRABP2	25416.46138	11675.10211	-1.122328017
100128071	FAM229A	22.88174944	10.5066991	-1.122887871
399474	TMEM200B	2265.34444	1036.72988	-1.127690377
7305	TYROBP	605.8004881	277.2432413	-1.12769045
56992	KIF15	570.8357758	260.7911347	-1.130180928
7101	NR2E1	3962.876173	1808.227038	-1.131972057
151112	ZSWIM2	28.22498871	12.86615854	-1.133391633
6909	TBX2	705.0679595	321.1164097	-1.13466593
11156	PTP4A3	1944.054052	884.9079268	-1.135469074
116969	ART5	21.76619609	9.90424733	-1.135970054
25798	BRI3	3057.650846	1390.839173	-1.136468067
8626	TP63	1696.832982	771.2111468	-1.13764676
54532	USP53	3385.168143	1538.236494	-1.137950169
55344	PLCXD1	10363.46287	4705.407819	-1.139114476
6480	ST6GAL1	541.8502179	245.5865207	-1.141662728
79915	ATAD5	765.3833753	346.7902337	-1.142119295
5077	PAX3	58.41429269	26.45336211	-1.142870314
1021	CDK6	917.0560959	414.9982867	-1.143904605
125228	FAM210A	3360.071378	1518.490509	-1.145853989
283229	CRACR2B	532.0160632	239.9437806	-1.148773387
83881	MIXL1	90.67062008	40.80703943	-1.151817105
6490	PMEL	156.1810832	70.23063159	-1.153047409

100533952	RBAK-RBAKDN	63.14862576	28.37840232	-1.153957969
335	APOA1	50.69728523	22.78227144	-1.153996901
53826	FXYD6	18.44529915	8.282158726	-1.15517443
342897	NCCRP1	1094.100109	490.7149854	-1.156787515
3781	KCNN2	447.8625524	200.8412514	-1.157000422
51092	SIDT2	4680.323197	2098.28902	-1.157394748
152503	SH3D19	1433.520899	641.9263603	-1.159083227
9355	LHX2	3686.311332	1646.077831	-1.163145369
3159	HMGA1	9527.900277	4240.740817	-1.167842002
342346	C16orf96	59.85345656	26.62695135	-1.168547309
23127	COLGALT2	8804.292785	3904.128169	-1.173206706
783	CACNB2	24.54294189	10.86769149	-1.175262675
55197	RPRD1A	1763.363406	779.8421701	-1.17707575
285489	DOK7	51.44369587	22.73954596	-1.177790846
728498	GOLGA8H	272.881981	120.6097031	-1.177931156
6606	SMN1	1046.366318	462.019485	-1.179362406
9497	SLC4A7	309.3634932	136.4450032	-1.180983398
22915	MMRN1	1243.374148	547.2073295	-1.184101029
54800	KLHL24	2154.129051	947.821761	-1.184416993
8565	YARS	3581.217576	1574.623988	-1.18544281
220001	VWCE	42.00759053	18.45077221	-1.186968841
1815	DRD4	52.18960427	22.91070985	-1.187740304
401024	FSIP2	27.85268814	12.2235205	-1.188156717
10620	ARID3B	2491.099626	1092.393041	-1.189290691
8786	RGS11	35.98433987	15.7744259	-1.189781692
643853	TMPPE	126.3129728	55.35315654	-1.190265322
10203	CALCRL	403.1130056	176.5558343	-1.191059834
3491	CYR61	14466.90468	6331.028532	-1.192244475
146439	BICDL2	19.50808007	8.530647525	-1.193344639
287	ANK2	5770.768147	2523.361389	-1.193416529
7468	NSD2	3497.554282	1528.482334	-1.194246571
158405	KIAA1958	664.5090871	290.0830817	-1.195822772
126393	HSPB6	30.22920722	13.19075121	-1.196416416
5172	SLC26A4	34.95248274	15.17015808	-1.204158818
199223	TTC21A	96.03829268	41.49717907	-1.210596489
971	CD72	16.11725971	6.959045978	-1.21164503
5163	PDK1	1469.420193	632.5276219	-1.216046618
1122	CHML	780.9075082	335.7460849	-1.217781107
8654	PDE5A	95.61970279	41.04700097	-1.220031104
8864	PER2	201.9952586	86.62903165	-1.221398933
55603	FAM46A	26.88457482	11.52767123	-1.22167756
54507	ADAMTSL4	2781.543408	1192.49545	-1.221901858
4922	NTS	21.51915417	9.212239966	-1.223997475

221416	C6orf223	19.19194453	8.20744143	-1.225496439
5230	PGK1	42614.33113	18219.29128	-1.225871847
7378	UPP1	5469.743961	2331.21168	-1.230393292
7373	COL14A1	338.4846371	144.24381	-1.230580946
27134	TJP3	799.2480664	339.3254342	-1.235973777
3670	ISL1	76.24380271	32.32288954	-1.238063901
55355	HJURP	801.6506147	339.6280866	-1.239017829
11045	UPK1A	1001.829286	423.701621	-1.241516138
2264	FGFR4	635.045662	268.4265427	-1.242332995
25854	FAM149A	508.1286176	214.6339621	-1.243315343
4885	NPTX2	142.061776	59.94181484	-1.244883757
55843	ARHGAP15	2095.982965	882.8942483	-1.247314442
54020	SLC37A1	462.7872212	194.8746765	-1.247802401
6658	SOX3	13613.72986	5727.499546	-1.249085043
55093	WDYHV1	211.1469741	88.68829509	-1.251431954
9467	SH3BP5	374.4121836	157.2486364	-1.251579876
79789	CLMN	38.59880841	16.17369758	-1.25490677
151306	GPBAR1	48.72919828	20.41608119	-1.255080515
57615	ZNF492	493.3705529	206.6299944	-1.255621919
339456	TMEM52	301.8485647	126.3380766	-1.256535427
115362	GBP5	792.3829052	331.3915461	-1.257660957
7349	UCN	61.72698466	25.77097577	-1.260154155
140738	TMEM37	56.0758963	23.40540863	-1.260538822
3064	HTT	2857.142758	1191.657523	-1.261603452
120071	LARGE2	365.1329198	152.2837226	-1.261660003
79924	ADM2	157.9940417	65.7158949	-1.265555886
91522	COL23A1	143.0408243	59.32435116	-1.269730633
730098	LOC730098	95.88001782	39.74450693	-1.270474697
79956	ERMP1	706.5069445	292.5160739	-1.272187838
389072	PLEKHM3	1749.37075	723.6505204	-1.273471041
7020	TFAP2A	143.4237251	59.28321138	-1.274588188
56979	PRDM9	274.6445879	113.1548647	-1.279267253
25914	RTTN	1003.825184	412.9235077	-1.281561587
135932	TMEM139	13.75817736	5.646943607	-1.28474723
345895	RSPH4A	122.2133905	50.10519796	-1.286370182
81618	ITM2C	6577.380145	2688.51179	-1.290705256
51168	MYO15A	38.19009254	15.57483056	-1.293981948
9920	KBTBD11	93.64640906	38.05465273	-1.29915082
148229	ATP8B3	89.80482749	36.49126912	-1.299241673
158326	FREM1	23.5220835	9.554279933	-1.299796802
8482	SEMA7A	40.65612868	16.46489017	-1.304079958
8497	PPFIA4	345.1253703	139.6056768	-1.305762924
440	ASNS	2222.871431	898.2437199	-1.307245657

93	ACVR2B	1187.186178	478.8249497	-1.309975967
51704	GPRC5B	1931.749198	778.544971	-1.311055518
10087	COL4A3BP	1650.049293	664.4633507	-1.312247591
56479	KCNQ5	25.75682657	10.36971041	-1.312579248
8577	TMEFF1	160.4379636	64.41435452	-1.31656143
83543	AIF1L	559.0924996	224.42104	-1.316879053
84699	CREB3L3	43.63737162	17.50148116	-1.318087184
27165	GLS2	243.6820988	97.62697884	-1.319648481
2695	GIP	127.1057593	50.86246555	-1.321356099
7697	ZNF138	418.4982043	166.8198637	-1.326930348
131405	TRIM71	736.986047	293.4290532	-1.328625582
18	ABAT	3884.851483	1546.458453	-1.328891371
84628	NTNG2	68.82320426	27.37291697	-1.33014588
1112	FOXN3	1367.460896	542.525112	-1.333737756
8204	NRIP1	2030.757494	802.5837158	-1.339294178
7634	ZNF80	827.9001521	327.0943986	-1.33974973
55636	CHD7	2149.192475	848.3048597	-1.34113996
3613	IMPA2	1196.556776	472.2328935	-1.341318413
138255	C9orf135	250.775384	98.94801064	-1.341653134
5454	POU3F2	3640.850652	1429.939659	-1.348321294
388588	SMIM1	235.0006279	92.20262664	-1.349784856
730394	GTF2H2C_2	1212.714395	475.6423547	-1.350290729
222223	KIAA1324L	459.0746216	179.9537629	-1.35110241
1649	DDIT3	405.5376264	158.6371466	-1.354105142
27123	DKK2	24.51373337	9.584533699	-1.35481007
9060	PAPSS2	696.8491848	272.0658561	-1.356890543
3398	ID2	5886.218808	2297.928621	-1.357007187
4323	MMP14	59.4061987	23.1489143	-1.359668944
6405	SEMA3F	457.6472559	178.2168379	-1.360602381
90649	ZNF486	386.4508181	150.4889196	-1.360627554
60675	PROK2	48.33183112	18.80618919	-1.361766118
7179	TPTE	836.8624328	325.4435509	-1.36258316
54587	MXRA8	271.6112584	105.5807453	-1.363196526
10793	ZNF273	438.0326231	169.9704313	-1.365754529
8553	BHLHE40	1269.418861	491.6526004	-1.368457005
54361	WNT4	30.87731115	11.91275997	-1.374039428
147798	TMC4	178.2220974	68.31805468	-1.383337424
6319	SCD	28897.37997	11066.21904	-1.38477631
257407	C2orf72	222.4693151	85.17522137	-1.385100664
6261	RYR1	264.2184859	101.0177314	-1.38712286
7545	ZIC1	1727.8093	660.2180517	-1.387929504
9886	RHOBTB1	159.7562835	61.04077097	-1.388027587
11148	HHLA2	175.9854643	67.18086276	-1.389334045

717	C2	40.88121549	15.57817906	-1.391911486
146540	ZNF785	60.73920642	23.12748812	-1.393019476
8711	TNK1	19.38721628	7.356912073	-1.397933414
100506243	KRBOX1	28.83475362	10.92076118	-1.40073528
55803	ADAP2	1791.462427	678.3136024	-1.401113457
114793	FMNL2	1398.668881	527.8481683	-1.405859547
56154	TEX15	285.5413028	107.7173855	-1.406448323
91851	CHRDL1	97.61231818	36.71994044	-1.410499501
83401	ELOVL3	39.11535991	14.70225195	-1.41169809
114132	SIGLEC11	18.69118492	7.023960744	-1.412001344
10507	SEMA4D	608.0549893	228.4353437	-1.412415916
7087	ICAM5	1533.726884	575.721487	-1.413598638
4692	NDN	102.9920874	38.65096256	-1.413957256
55435	AP1AR	677.929078	254.0347927	-1.41610825
4661	MYT1	241.3363313	90.15401624	-1.42058145
83643	CCDC3	46.23792686	17.22793919	-1.424326577
8537	BCAS1	85.19321402	31.70267393	-1.426133991
151835	CPNE9	10.96680417	4.07001053	-1.43003874
402778	IFITM10	96.26908028	35.68252429	-1.431854828
158219	TTC39B	102.6763981	38.00174946	-1.433966851
56099	PCDHGB7	174.9098047	64.73564869	-1.433978862
3685	ITGAV	41365.6981	15293.25793	-1.435539153
27345	KCNMB4	171.6764647	63.26517791	-1.44020873
317649	EIF4E3	12933.29275	4743.907093	-1.446941964
4585	MUC4	15.64730425	5.737363048	-1.447454412
143872	ARHGAP42	79.5089161	29.09888264	-1.450152896
2118	ETV4	2020.651137	739.5027423	-1.450192861
401720	FIGNL2	1121.727419	410.3701163	-1.450724561
79412	KREMEN2	3403.764231	1242.648009	-1.453713411
55313	CPPED1	291.3511114	106.2843587	-1.454829515
85474	LBX2	17.40293196	6.340499966	-1.456661874
7015	TERT	233.6113297	85.101323	-1.456856778
8313	AXIN2	245.6597757	89.47553575	-1.457096468
79792	GSDMD	61.72559237	22.47252585	-1.457706488
94031	HTRA3	398.0367238	144.8930737	-1.457912911
23235	SIK2	4593.10975	1671.139234	-1.458639317
26140	TTLL3	84.08657016	30.59211048	-1.458715761
3352	HTR1D	98.31825999	35.72543073	-1.460507978
93426	SYCE1	95.15079135	34.43365971	-1.466396132
2902	GRIN1	58.23575139	21.01727408	-1.470329542
2119	ETV5	1302.220697	467.4529672	-1.478080852
5144	PDE4D	915.0913865	326.8782644	-1.485162378
728215	FAM155A	66.72133224	23.82703447	-1.485548692

158038	LINGO2	464.3692962	165.5151603	-1.488309219
3164	NR4A1	1959.917038	697.7990677	-1.489909012
26050	SLITRK5	2927.138391	1039.283727	-1.493901389
26025	PCDHGA12	9.144576607	3.23337239	-1.499876702
8744	TNFSF9	202.7750605	71.64606438	-1.500920862
25928	SOSTDC1	48.86210825	17.26312971	-1.501022072
11217	AKAP2	3741.152883	1320.382801	-1.502526671
645121	CCNI2	70.24404484	24.70481615	-1.507583602
143630	UBQLNL	9.658423725	3.393521928	-1.509004422
51778	MYOZ2	304.6485709	106.9115389	-1.510728402
27443	CECR2	2394.938473	840.026088	-1.511482555
6515	SLC2A3	8346.977086	2926.110871	-1.512269376
57644	MYH7B	19.3244538	6.769747083	-1.513253796
282809	POC1B	1333.62714	465.7895374	-1.51760523
84889	SLC7A3	2061.446458	718.9040019	-1.519785951
7291	TWIST1	104.3564777	36.37850817	-1.520361869
1295	COL8A1	519.1448122	180.9449002	-1.520586578
55630	SLC39A4	522.453425	181.8436679	-1.522603742
57685	CACHD1	164.2706241	57.00914743	-1.52680918
348738	C2orf48	13.68172783	4.721469497	-1.534942581
8310	ACOX3	210.1169258	71.98454634	-1.545433255
51673	TPPP3	28.37315119	9.71655853	-1.546009062
9717	SEC14L5	138.236166	47.24886818	-1.548783434
10256	CNKSR1	9.972953877	3.408500674	-1.54888361
59353	TMEM35A	3040.298016	1038.688878	-1.549449162
57586	SYT13	10.97400375	3.744647726	-1.551188064
8174	MADCAM1	89.85140174	30.51585172	-1.557982153
84034	EMILIN2	2242.966376	759.5740642	-1.562145443
80059	LRRTM4	15.00852386	5.071773627	-1.565219831
2245	FGD1	9963.826132	3365.330874	-1.565951492
23090	ZNF423	199.7197578	67.41624298	-1.566808927
220979	C10orf25	7.258013639	2.444471633	-1.570052104
8490	RGS5	7249.008	2438.167279	-1.571986471
83604	TMEM47	5184.593683	1741.85645	-1.573605196
222643	UNC5CL	84.35057903	28.28689187	-1.576264308
51309	ARMCX1	27.07753684	9.03100333	-1.584138325
402573	C7orf61	6.539775199	2.180175583	-1.584796716
4038	LRP4	2314.397826	770.1711114	-1.587385958
57576	KIF17	481.3024218	159.7634207	-1.591006555
8277	TKTL1	5.285780648	1.751795078	-1.593282536
93349	SP140L	308.8591863	102.0017577	-1.598355228
440836	ODF3B	300.5948923	99.15872316	-1.600008896
400935	IL17REL	13.60104784	4.481143273	-1.601779044

90625	ERVH48-1	43.48779467	14.32226553	-1.60235083
2966	GTF2H2	1475.975916	485.0586088	-1.605438199
84519	ACRBP	13.5799123	4.459573046	-1.606496663
154790	CLEC2L	40.05491875	13.07145569	-1.615559603
7980	TFPI2	2457.710839	801.7306467	-1.616125657
23414	ZFPM2	1082.997087	353.2381715	-1.616316206
89832	CHRFAM7A	896.6014032	292.3730963	-1.616656194
7710	ZNF154	104.9416888	34.14850331	-1.619693659
1645	AKR1C1	5.819488267	1.890652408	-1.622008144
149483	CCDC17	38.92950558	12.63790182	-1.623107059
6545	SLC7A4	2.281178483	0.739614398	-1.624934116
339390	CLEC4G	2.281178483	0.739614398	-1.624934116
8128	ST8SIA2	2.28537841	0.740519034	-1.625824342
93273	LEMD1	2.28537841	0.740519034	-1.625824342
2904	GRIN2B	2.787584837	0.900249187	-1.630619416
130888	FBXO36	354.037198	114.2442123	-1.63177987
221914	GPC2	94.8066741	30.53874705	-1.634347752
1184	CLCN5	1955.820296	629.1248932	-1.636355466
49	ACR	14.19986005	4.553617923	-1.640791561
83854	ANGPTL6	82.11975583	26.20356077	-1.647966466
256949	KANK3	1542.907967	490.1925237	-1.654231623
152831	KLB	78.44396109	24.90815943	-1.655043969
9456	HOMER1	762.3551902	241.3985462	-1.659046335
81848	SPRY4	2630.088935	825.8659863	-1.671131986
80312	TET1	2880.68087	904.4483005	-1.6712999
26051	PPP1R16B	80.72705759	25.27043751	-1.675601666
7694	ZNF135	15.30878787	4.787934037	-1.676884875
83878	USHBP1	40.77121761	12.75011754	-1.677040496
7135	TNNI1	7.916073915	2.475330142	-1.677164129
728780	ANKDD1B	8.929529559	2.789886	-1.678377999
7137	TNNI3	29.29076621	9.139190022	-1.680307718
1441	CSF3R	3.584940047	1.110395226	-1.690875721
100137049	PLA2G4B	78.38818139	24.20532604	-1.695311628
149461	CLDN19	10.47243398	3.228984892	-1.697444194
1139	CHRNA7	615.8175181	189.4940266	-1.700350537
9262	STK17B	1051.691406	323.5565236	-1.700621772
126969	SLC44A3	72.02859798	21.99433817	-1.711437633
165215	FAM171B	7161.961351	2185.355198	-1.712486945
167410	LIX1	497.584119	150.9491188	-1.720878106
168620	BHLHA15	15.92970495	4.831346435	-1.721222335
285141	ERICH2	37.77626204	11.4213791	-1.725743091
169026	SLC30A8	2457.538396	742.2871313	-1.727164694
9473	THEMIS2	304.2503833	91.84938582	-1.727917101

460	ASTN1	65.78664653	19.84681267	-1.728887439
2827	GPR3	46.76914362	14.08573798	-1.731321861
284422	SMIM24	124.7425145	37.48610342	-1.734525472
63982	ANO3	12.6429655	3.786292642	-1.739477072
56944	OLFML3	15.96618204	4.780610657	-1.739752546
414332	LCN10	15.45025456	4.624627881	-1.740221418
389125	MUSTN1	21.64754096	6.476820288	-1.740845533
22925	PLA2R1	4.911970309	1.464110295	-1.7462776
1010	CDH12	55.17108352	16.39676913	-1.750500745
54549	SDK2	394.751611	117.2953134	-1.750799783
664	BNIP3	17920.3426	5321.247555	-1.751761791
2348	FOLR1	67.44471875	19.83467853	-1.765680463
10253	SPRY2	2438.977102	716.6825662	-1.766870048
407977	TNFSF12-TNFSF13	68.51218366	20.10207322	-1.769016267
9068	ANGPTL1	2.85525476	0.834234809	-1.775094059
81031	SLC2A10	871.9176437	254.2340473	-1.778034622
8693	GALNT4	1705.263128	490.895081	-1.796507754
388581	C1QTNF12	72.49727659	20.80069668	-1.801294951
51554	ACKR4	37.43182984	10.73699033	-1.801675925
284111	SLC13A5	4.183403034	1.197402007	-1.804769403
3912	LAMB1	610.7264078	172.2776337	-1.825790816
79152	FA2H	2465.667879	694.8466627	-1.827211937
586	BCAT1	3784.72482	1065.477654	-1.828688073
4481	MSR1	6.540054935	1.839690123	-1.829839975
7857	SCG2	120.6967801	33.85515562	-1.833939733
1356	CP	29.30148859	8.2034652	-1.836668611
254122	SNX32	2.888423952	0.80796365	-1.837920218
171024	SYNPO2	2250.9276	629.2353685	-1.838847984
11320	MGAT4A	33.74494691	9.428529698	-1.839566767
6445	SGCG	112.3028596	31.23401713	-1.846204627
5325	PLAGL1	386.3141858	107.3763417	-1.847098497
494470	RNF165	77.50244935	21.52442374	-1.848267292
107080638	TBC1D7-LOC10013	13.90375244	3.859337215	-1.849049288
7570	ZNF22	22.52794675	6.246480565	-1.850600358
147664	ERVV-1	18.62323297	5.163497495	-1.850683033
729085	FAM198A	14.44095893	3.96527107	-1.864675147
7145	TNS1	8.153939638	2.227086825	-1.872339473
10144	FAM13A	14.48310993	3.93336272	-1.880536285
51050	PI15	757.8969675	204.237131	-1.891756557
84101	USP44	1416.252472	381.3536151	-1.892877193
441234	ZNF716	56.44017072	15.19648563	-1.89298463
3050	HBZ	25.60539315	6.872590712	-1.897521761
728340	GTF2H2C	503.6417451	134.9454445	-1.900021593

80709	AKNA	694.5245544	185.7515863	-1.902651169
642475	MROH6	61.50033292	16.4124613	-1.905802611
64283	ARHGEF28	24.29878311	6.482784616	-1.906198518
5333	PLCD1	1833.511314	485.5209807	-1.917003623
26166	RGS22	3.450929093	0.912499651	-1.919088919
255022	CALHM1	3.185915636	0.841164407	-1.921248354
146429	SLC22A31	91.38081262	23.70521108	-1.946687033
79094	CHAC1	271.3190143	69.56870772	-1.96347973
84000	TMPRSS13	6.139346828	1.571064972	-1.966342329
5210	PFKFB4	1117.537682	283.869908	-1.97702165
116844	LRG1	211.2599868	53.46333925	-1.982397688
154141	MBOAT1	2015.351052	509.870982	-1.982827024
255488	RNF144B	41.96167423	10.59567073	-1.985597325
50515	CHST11	1977.338105	496.1299369	-1.99476966
5652	PRSS8	34.24234799	8.582828602	-1.996256537
3485	IGFBP2	413.8016608	103.1590895	-2.004068491
29969	MDFIC	3873.808963	963.933499	-2.006747284
7855	FZD5	11003.54511	2714.407495	-2.019259181
5797	PTPRM	408.2651966	99.84244788	-2.031781377
283767	GOLGA6L1	4.843670939	1.180531816	-2.036663933
93986	FOXP2	1994.487596	482.2249977	-2.048239807
148113	CILP2	85.68115421	20.71356969	-2.048401712
8224	SYN3	5.400916279	1.298492497	-2.056366507
283710	LOC283710	9.477952059	2.269328621	-2.062309823
646457	C19orf67	5.412034554	1.291954024	-2.066616319
5617	PRL	3.805283112	0.907600778	-2.067874043
23349	PHF24	12.18981972	2.89142821	-2.075822602
401335	C7orf65	51.71409466	12.25985376	-2.07661577
5443	POMC	29.89486983	6.967051203	-2.101277858
1373	CPS1	19087.90071	4416.084978	-2.111818605
4345	CD200	4.102757504	0.946061314	-2.116588293
100131378	C11orf91	5.463484159	1.254910412	-2.122236902
51557	LGSN	110.3433811	25.26010394	-2.127067612
117166	WFIKKN1	312.3765836	71.48740281	-2.127525366
1848	DUSP6	8950.097413	2026.283109	-2.143067625
8433	UTF1	15.02414043	3.387041925	-2.149184703
3109	HLA-DMB	131.0338659	29.37532311	-2.157263101
4255	MGMT	673.7378042	151.034171	-2.157312261
341405	ANKRD33	12.04684214	2.694200063	-2.16072613
710	SERPING1	4870.986614	1082.877482	-2.169343995
164832	LONRF2	19.48046642	4.325396143	-2.171124047
3060	HCRT	22.49889662	4.949762875	-2.184422933
2047	EPHB1	175.7590032	38.07287653	-2.206763113

338645	LUZP2	1735.322823	375.06354	-2.209997143
54106	TLR9	58.70594295	12.6649547	-2.212664642
9427	ECEL1	301.540579	64.97548352	-2.214384792
83592	AKR1E2	44.95530797	9.62552347	-2.223554561
2888	GRB14	88.28391595	18.83308255	-2.228881468
84634	KISS1R	537.9706005	113.8400974	-2.240518532
126014	OSCAR	384.7847959	81.15416184	-2.245314807
1285	COL4A3	6.099065805	1.281042255	-2.251270218
25893	TRIM58	145.6903387	30.41451184	-2.260073456
3816	KLK1	34.40984077	7.11014091	-2.274871159
3174	HNF4G	21.17238127	4.285286381	-2.304720013
11174	ADAMTS6	105.4199035	21.22494009	-2.31231489
4118	MAL	128.217698	25.78150592	-2.314186972
131540	ZDHHC19	132.466268	26.23992222	-2.335789681
7148	TNXB	215.1730565	42.33484235	-2.345580016
6866	TAC3	35.57916377	6.959627405	-2.353950627
256691	MAMDC2	1710.366476	329.9090715	-2.374165128
5156	PDGFRA	1240.333626	236.9227211	-2.388239764
28999	KLF15	29.89956953	5.630926302	-2.40868054
83690	CRISPLD1	2028.798988	381.3653912	-2.4113801
54514	DDX4	16.39223527	3.069351296	-2.417006915
10826	FAXDC2	864.514647	161.5895195	-2.419556777
4828	NMB	12.44364339	2.321925164	-2.422015576
388531	RGS9BP	62.11285967	11.56921381	-2.424601161
1268	CNR1	28.85257431	5.320688165	-2.439015288
391059	FRRS1	86.55551138	15.9319142	-2.441706071
134526	ACOT12	10.76808542	1.979831926	-2.443311894
8633	UNC5C	499.14829	91.70985552	-2.444319799
254173	TTLL10	6.231284692	1.140244717	-2.450186146
10194	TSHZ1	220.2411304	39.96906775	-2.462128091
1004	CDH6	49021.59523	8849.698368	-2.469717244
56776	FMN2	38.90524742	7.016291744	-2.471184112
79632	FAM184A	61.33699107	11.04180268	-2.473781671
1299	COL9A3	169.5822715	30.46437834	-2.476790149
6326	SCN2A	44.33112271	7.944809804	-2.480235313
79727	LIN28A	169.3954407	30.31421057	-2.482328887
136288	C7orf57	3732.060719	665.7774048	-2.486860644
9940	DLEC1	30.15187419	5.343939974	-2.496271973
55753	OGDHL	1781.050844	312.4300961	-2.511123364
6857	SYT1	1403.471629	244.1383218	-2.523229226
9743	ARHGAP32	362.0003396	62.51341025	-2.533753438
2566	GABRG2	393.1650536	67.8711749	-2.534264201
7075	TIE1	37.51562906	6.388202542	-2.554009789

1593	CYP27A1	31.78836313	5.38944502	-2.560290106
64218	SEMA4A	18.09194135	3.045701624	-2.570500706
25837	RAB26	10.3692516	1.724291753	-2.588235965
10351	ABCA8	30.50423479	5.055394877	-2.59311385
10468	FST	2228.846104	364.2522236	-2.613287321
4857	NOVA1	275.3039147	44.30176852	-2.635588927
3948	LDHC	9.303069447	1.456391915	-2.675308159
487	ATP2A1	197.0591188	30.52287437	-2.690665777
391195	OR2T33	7.273402181	1.126073959	-2.691328767
1296	COL8A2	75.82812068	11.63014997	-2.704863268
114907	FBXO32	42.23025749	6.394571985	-2.723357342
79674	VEPH1	14.57866446	2.206181044	-2.72423547
3270	HRC	451.4644338	68.21651688	-2.726419339
100271846	ERVV-2	79.78225937	12.05432319	-2.726517329
4593	MUSK	7.014473884	1.058586832	-2.728195287
6335	SCN9A	64.68895891	9.703318946	-2.736969295
9315	NREP	2488.176704	368.6369735	-2.754816263
339453	TMEM240	19.25447477	2.85138402	-2.755459512
30010	NXPH1	26.33902473	3.857879784	-2.771321834
8076	MFAP5	1019.575094	147.378923	-2.790365916
148198	ZNF98	20.268057	2.911854901	-2.799197419
124602	KIF19	9.693658542	1.384439909	-2.80773883
10251	SPRY3	2170.309004	308.6101979	-2.814042824
51441	YTHDF2	13528.02443	1893.931942	-2.836494776
402665	IGLON5	106.6511999	14.42043856	-2.88671325
799	CALCR	1758.937234	228.7589083	-2.942804172
79772	MCTP1	461.6590972	59.46935832	-2.956609502
79729	SH3D21	307.8639869	39.16816704	-2.974539593
7425	VGF	2488.891048	308.8660398	-3.010449919
1902	LPAR1	36.12119141	4.448113475	-3.021579981
151254	C2CD6	21.00395102	2.492582819	-3.074947391
22871	NLGN1	56.8849405	6.529911444	-3.122911438
81704	DOCK8	22.92708484	2.584064593	-3.149338891
83482	SCRT1	190.636697	20.99283247	-3.182857119
4772	NFATC1	80.13807917	8.767725252	-3.192213435
56171	DNAH7	125.0642918	13.46028862	-3.215888682
345557	PLCXD3	49.83513586	5.174184717	-3.267759798
7001	PRDX2	153.3306044	15.70826475	-3.28704996
56884	FSTL5	102.643851	10.41647405	-3.300708287
203328	SUSD3	21.67783302	2.148624236	-3.334735445
9074	CLDN6	235.4504142	23.1140625	-3.348580507
92162	TMEM88	4208.409752	409.9812722	-3.359645265
1286	COL4A4	24.49589179	2.339738685	-3.388120499

51305	KCNK9	55.79123916	5.274798473	-3.402850714
10417	SPON2	37.79567796	3.497703037	-3.433741556
79827	CLMP	85.7312771	7.92371967	-3.435571889
7078	TIMP3	569.756993	52.12781749	-3.450221364
11030	RBPMS	61.27984952	5.515154887	-3.473939446
55103	RALGPS2	133.4711517	11.81602347	-3.497711452
760	CA2	110.2932028	9.57544599	-3.525860389
343172	OR2T8	13.93998066	1.193507276	-3.545949292
29951	PDZRN4	77.0464859	6.532897482	-3.559934251
144568	A2ML1	262.7817558	22.07099901	-3.57364128
28954	REM1	13.94788356	1.169876917	-3.575617569
282890	ZNF311	22.63604776	1.866530845	-3.600190832
339479	BRINP3	507.2543293	40.66983346	-3.640678285
10752	CHL1	180.5791143	14.0782133	-3.681094887
6332	SCN7A	45.31741421	3.362434279	-3.752487569
22865	SLITRK3	592.9595155	43.27448246	-3.776345137
10840	ALDH1L1	2016.718529	144.6653264	-3.801218661
26052	DNM3	142.553666	10.16687158	-3.809557416
118427	OLFM3	33.32516467	2.266629201	-3.8779917
1103	CHAT	61.0195207	4.095188665	-3.897269025
9388	LIPG	63.88853478	4.277211165	-3.900814714
5592	PRKG1	120.6112968	7.915820453	-3.929482341
22999	RIMS1	380.8072381	24.91003478	-3.934261959
429	ASCL1	29.97497225	1.929842838	-3.957203151
448831	FRG2	86.71840345	5.579540673	-3.958119931
7103	TSPAN8	47.40394853	2.987412295	-3.98803897
8492	PRSS12	58.45820735	3.641338751	-4.004864724
170825	GSX2	636.3717092	36.44586421	-4.126042684
6572	SLC18A3	243.9413377	13.79191832	-4.144639214
100288801	FRG2C	298.8493681	16.88311515	-4.145765463
131149	OTOL1	166.3426121	9.221782988	-4.172968266
431707	LHX8	97.78464412	5.270589142	-4.213571786
6549	SLC9A2	39.31763064	2.018252944	-4.283997483
285313	IGSF10	33.96173251	1.678518974	-4.338649307
8784	TNFRSF18	58.46151498	2.66985593	-4.452653417
26002	MOXD1	357.4701958	15.90226933	-4.490518397
1535	CYBA	288.1563684	12.30049581	-4.55006353
441581	FRG2B	84.27153625	3.367559778	-4.645269965
143662	MUC15	343.3001351	13.41619579	-4.677422871
221002	RASGEF1A	344.9660137	12.63924346	-4.770472217
64849	SLC13A3	488.6825091	17.05925637	-4.840270804
130399	ACVR1C	83.29241732	2.728542298	-4.931982849
80034	CSRNP3	795.7814112	23.36679576	-5.089840381

2045	EPHA7		277.7957988	7.491108758	-5.212701703
57554	LRRC7		108.2933499	1.570469771	-6.107604669

Table S1c

Gene ID	Gene symbol	R1284W Expression	p190A Expression	log2FoldChange (p190A/R1284W)
5796	PTPRK	9.622883035	435.8236442	5.501131464
6588	SLN	11.24391928	384.4510199	5.0955829
3815	KIT	4.11052808	132.8412022	5.014235125
4071	TM4SF1	6.141090961	190.420862	4.954552762
6422	SFRP1	8.473188474	214.4467344	4.661570576
5999	RGS4	7.183660249	178.117048	4.631962677
117154	DACH2	4.801526138	118.836604	4.629342441
2846	LPAR4	3.395225704	83.76955252	4.624846578
169792	GLIS3	1.643019763	36.96621049	4.491785417
130576	LYPD6B	7.098229915	159.3283506	4.488399885
56892	TCIM	54.53259845	1124.293918	4.365756529
26289	AK5	9.116180474	180.9329575	4.310881927
94240	EPSTI1	14.21951517	281.2496851	4.305907301
11098	PRSS23	360.4902319	6872.94622	4.252896598
4222	MEOX1	262.1784525	4822.138748	4.201052137
130574	LYPD6	1.929494885	34.08873314	4.14299986
2303	FOXC2	1.916824684	31.48774836	4.038000299
960	CD44	20.24547129	331.3772152	4.032803277
7058	THBS2	4.99435186	77.70115768	3.959566723
90627	STARD13	1.223038735	18.75045823	3.938383852
10873	ME3	8.382012299	119.3198516	3.83139364
4629	MYH11	26.97270384	381.4899821	3.822073118
4110	MAGEA11	1.581499552	22.06764231	3.802567449
344901	OSTN	2.218860465	30.20691948	3.766988215
221981	THSD7A	16.91093432	228.377559	3.755392619
1828	DSG1	6.024485642	80.95915725	3.748284295
115701	ALPK2	4.290803625	55.7424045	3.699455455
57381	RHOJ	2.912986649	37.74316383	3.695644368
4916	NTRK3	14.12034392	175.5041223	3.635657784
3777	KCNK3	9.010209487	111.7510883	3.632584422
140706	CCM2L	194.1186325	2398.308724	3.627006875
127254	ERICH3	1.52513043	18.76441725	3.620994953
3983	ABLIM1	2.901612075	35.13848508	3.598125435
9411	ARHGAP29	5.28816463	63.53285406	3.586663834
51702	PADI3	42.82155791	497.5435806	3.53841371
25878	MXRA5	1.175144677	13.55120797	3.527511172
1381	CRABP1	59.62247677	685.5205799	3.523271765
8829	NRP1	1.179905115	13.53075568	3.519499663
55024	BANK1	6.26519967	70.50909883	3.492377048
388662	SLC6A17	13.04772388	145.3572123	3.477732595

1794	DOCK2	1.149266554	12.6168064	3.456561426
5654	HTRA1	5.581769082	60.65558463	3.441846136
257019	FRMD3	59.13770203	618.7864875	3.387291603
286	ANK1	124.2133363	1289.924911	3.376395102
8660	IRS2	7.945165348	81.33188018	3.355671817
57282	SLC4A10	1.508424087	15.21671542	3.334542983
11010	GLIPR1	50.26714573	504.9286009	3.328391721
6641	SNTB1	3.218308297	32.24206275	3.324569606
100528021	ST20-MTHFS	1.218389649	12.18718127	3.322316992
2300	FOXL1	2.178131761	21.78644575	3.322267719
93649	MYOCD	4.197861034	41.40946372	3.302234206
147409	DSG4	2.89271026	28.23481252	3.286981319
4921	DDR2	73.39903402	715.8085964	3.285740888
84419	C15orf48	7.612978128	73.37961496	3.268846496
23544	SEZ6L	10.62690987	101.6455433	3.25775291
1823	DSC1	34.79617346	332.7647175	3.257501908
203859	ANO5	10.37377909	99.04276248	3.255110002
50964	SOST	2.860664728	26.97590081	3.237248809
7056	THBD	1.818392118	16.88076952	3.214645431
4005	LMO2	8.248104725	75.06515626	3.186008837
168667	BMPER	2.50490054	22.76685514	3.184109796
2857	GPR34	1.45537225	12.95978407	3.154581569
5915	RARB	2.485566795	21.81916289	3.133948974
89927	C16orf45	136.0247592	1180.715645	3.117720378
10568	SLC34A2	340.1484584	2917.521178	3.100506673
1293	COL6A3	2.133656392	18.18317383	3.091204275
4608	MYBPH	12.49587663	105.5704006	3.078681374
90102	PHLDB2	6.209063992	52.23197248	3.072485481
131578	LRRC15	5.532626218	46.04965142	3.057153871
6236	RRAD	10.93391524	90.4635738	3.048526895
3620	IDO1	9.983395379	80.40063131	3.009604361
51232	CRIM1	593.5708391	4768.335055	3.005993491
1012	CDH13	1.421073232	11.34479852	2.996978179
3778	KCNMA1	1.429629098	11.32448967	2.985733228
2983	GUCY1B1	65.53849518	516.7736153	2.97911796
1015	CDH17	3.151127557	24.75688398	2.973889681
79836	LONRF3	12.75947194	99.22265124	2.959100884
8707	B3GALT2	4.866224105	37.48632556	2.945489751
2313	FLI1	1.447654895	10.99271239	2.924757781
154	ADRB2	1.065976979	8.081360595	2.922421927
999	CDH1	274.0771002	2064.10589	2.912863286
5028	P2RY1	21.20025874	157.8220248	2.896144777
9211	LGI1	31.89809454	234.6990139	2.879269629

51286	CEND1	52.53733614	383.4874857	2.867764542
23316	CUX2	24.59310805	176.8682674	2.846349254
4907	NT5E	142.4661918	1021.04297	2.841352079
2254	FGF9	3.455184119	24.75819296	2.841071524
2766	GMPR	8.896413516	63.6601436	2.839094658
347365	ITIH6	3.799615277	27.08511888	2.833575169
84630	TTBK1	12.95707649	91.12347584	2.81408254
84166	NLRC5	5.185729531	36.19317635	2.803098858
84627	ZNF469	6.167187104	42.79375961	2.79471591
83857	TMTC1	78.94088098	547.630919	2.794359378
84141	EVA1A	2.447975912	16.92038035	2.789100732
83593	RASSF5	15.95886682	110.1346244	2.786837979
22822	PHLDA1	571.1322645	3848.757743	2.752496072
84969	TOX2	37.54893276	249.4368213	2.73183064
3239	HOXD13	8.212632444	54.13821607	2.720730712
10335	MRVI1	1.742359543	11.41021855	2.711212159
90293	KLHL13	7.203514812	46.65302649	2.695197757
4773	NFATC2	133.7496022	864.1725081	2.691784735
7045	TGFBI	7.200012414	46.05796544	2.677379384
7062	TCHH	1.018962625	6.511635991	2.675918918
83697	SLC4A9	2.415241103	15.27488429	2.660922332
144501	KRT80	2.079515461	13.00721059	2.644992292
22882	ZHX2	27.11586489	168.736743	2.637565067
80099	C7orf69	2.410387052	14.98510163	2.636192135
1435	CSF1	337.4525797	2094.554214	2.633886539
339761	CYP27C1	29.17554071	180.3085331	2.627636375
91683	SYT12	64.63471784	398.2361128	2.623242847
79971	WLS	4.097960801	25.10810382	2.61517499
166979	CDC20B	3.406691856	20.86567859	2.614688478
157638	FAM84B	35.87355582	218.8883116	2.60920226
5698	PSMB9	26.72256799	161.408025	2.594581749
3306	HSPA2	1491.351059	8994.59885	2.592439037
7262	PHLDA2	15.07445312	90.45374428	2.585074559
54885	TBC1D8B	3.448805939	20.52432168	2.573165685
728130	NUTM2D	10.23079932	60.85160472	2.572376443
3233	HOXD4	3.743280039	22.22269855	2.56965913
246	ALOX15	22.03178671	130.7498717	2.569151128
6710	SPTB	21.02670509	124.277968	2.563275857
9148	NEURL1	16.96720327	99.62354266	2.553737932
728392	LOC728392	104.0799682	608.7036289	2.548047541
100131137	BSPH1	1.348445512	7.823909921	2.536592534
3489	IGFBP6	78.80564939	455.3545912	2.530619469
2863	GPR39	6.45410466	37.23668775	2.528435871

54935	DUSP23	13.57903767	77.79216942	2.518243699
4940	OAS3	163.2751212	934.4217199	2.51676883
3106	HLA-B	110.7672794	632.3345318	2.513156234
1800	DPEP1	181.0663031	1033.472514	2.512910032
11043	MID2	96.88870938	552.9326563	2.512703319
3237	HOXD11	32.13037951	183.2893316	2.512112889
644019	CBWD6	2.057402839	11.72304144	2.510450707
11096	ADAMTS5	1.036462901	5.866923222	2.500935635
9394	HS6ST1	1583.537748	8944.628998	2.497870385
2890	GRIA1	6.45859658	36.26942695	2.489461337
7164	TPD52L1	107.2300608	593.8378904	2.469359743
55450	CAMK2N1	87.28223722	481.3269904	2.463257338
348174	CLEC18A	0.959595875	5.265622392	2.456105206
3167	HMX2	1.315992468	7.201577004	2.452161631
144535	CFAP54	11.20635949	61.20024518	2.449219754
84072	HORMAD1	19.65535603	106.075099	2.432091624
55531	ELMOD1	11.8252848	63.13686896	2.416607788
8714	ABCC3	5.765042001	30.73595579	2.414524325
28513	CDH19	11.17016659	59.50636589	2.413393312
55553	SOX6	3.374650161	17.97061883	2.412830235
5992	RFX4	4.060763756	21.55170823	2.407979222
1901	S1PR1	1.305432656	6.880591073	2.398004469
388697	HRNR	0.945677893	4.947088666	2.38715898
943	TNFRSF8	5.094464465	26.46561145	2.377116584
2312	FLG	1.699111011	8.822971575	2.376484523
388021	TMEM179	23.7104641	122.9088677	2.3739932
24141	LAMP5	114.1244046	586.6673635	2.361935403
57158	JPH2	35.85554891	184.0858153	2.360110155
800	CALD1	79.20350485	406.3712648	2.359162213
5457	POU4F1	19.2685599	98.53024792	2.354317936
4939	OAS2	3.392968671	17.28111747	2.348576498
84871	AGBL4	1.995156543	10.15842678	2.34810314
55170	PRMT6	73.70186932	373.500843	2.341338383
134	ADORA1	13.17971491	66.70223087	2.33941585
2641	GCG	88.60985996	448.1792133	2.338536591
51809	GALNT7	23.30143359	117.7491951	2.337226575
8605	PLA2G4C	28.04013111	141.6924004	2.337197382
80003	PCNX2	1.298521698	6.555050323	2.335736735
11240	PADI2	256.4674095	1281.646822	2.321151347
25945	NECTIN3	29.4931086	147.2882945	2.320192983
28996	HIPK2	211.0338908	1051.821882	2.317343805
64919	BCL11B	0.933327046	4.636337918	2.312531113
190	NR0B1	16.62733565	82.379139	2.308722036

1244	ABCC2	11.17261031	55.19902835	2.304676584
162494	RHBDL3	428.4418474	2111.983716	2.301427406
182	JAG1	1192.662347	5877.589229	2.301038875
5155	PDGFB	109.6993645	540.5925532	2.30098647
5359	PLSCR1	7.462581376	36.66176287	2.296529496
266727	MDGA1	33.80385609	165.9916281	2.295850748
131544	CRYBG3	7.47491479	36.30927241	2.280208982
79899	PRR5L	2.646060753	12.78275845	2.272281105
3755	KCNG1	116.0896588	559.4555514	2.268784051
54625	PARP14	85.27383426	409.704575	2.26440897
84674	CARD6	3.345355424	16.05753152	2.263018727
25884	CHRDL2	1.640990497	7.874096381	2.262547487
254778	C8orf46	12.14146104	58.18198479	2.260630474
64127	NOD2	1.649296968	7.889439047	2.258071536
6999	TDO2	15.18553827	72.3225573	2.251747645
55806	HR	116.2409231	553.187589	2.250650725
3235	HOXD9	54.10656628	256.7769404	2.246640056
4938	OAS1	0.914108226	4.329737251	2.24384259
167838	TXLNB	49.34655704	231.7911606	2.231804215
26064	RAI14	115.8437827	543.5854391	2.230326194
23551	RASD2	23.08054725	108.2775488	2.229984796
4135	MAP6	134.2541821	629.0111225	2.228118498
57822	GRHL3	15.21317382	71.21872786	2.226935502
10788	IQGAP2	2.365216049	11.06516455	2.225981029
4883	NPR3	1.61851829	7.570448626	2.225705128
120939	TMEM52B	1.974368303	9.201594711	2.220492775
80326	WNT10A	259.6814899	1206.581341	2.21611009
165	AEBP1	76.09529375	352.1229058	2.210199943
84709	MGARP	47.57552694	218.5905649	2.19993959
23220	DTX4	19.94888605	91.6185477	2.199331506
347902	AMIGO2	39.17473593	179.6897694	2.197512817
288	ANK3	20.60925766	94.3039159	2.194025139
284306	ZNF547	6.738262799	30.78208515	2.191642361
5950	RBP4	5.079761813	23.14105021	2.187621583
7042	TGFB2	1082.155215	4925.318745	2.18630965
2274	FHL2	5.046649102	22.91707305	2.183025113
59277	NTN4	248.3484042	1127.357531	2.182507744
10566	AKAP3	2.67774864	12.11076002	2.177196959
80833	APOL3	1.623354939	7.251207232	2.159242732
84632	AFAP1L2	4.023536237	17.94721519	2.157224072
2019	EN1	0.897982136	4.001053156	2.155621145
6252	RTN1	0.897982136	4.001053156	2.155621145
1141	CHRNB2	39.16009669	174.4415629	2.155287589

10344	CCL26	0.962397544	4.276918113	2.151866719
825	CAPN3	10.53201216	46.69404769	2.148457563
8061	FOSL1	38.80994685	171.8560381	2.146702179
3236	HOXD10	24.3067351	107.0539276	2.138909698
10046	MAMLD1	80.07431294	352.4137956	2.137858982
8651	SOCS1	44.2620067	194.6566565	2.136790916
81693	AMN	1.587658186	6.947090487	2.129508547
1839	HBEGF	610.1123119	2664.39935	2.126663586
8038	ADAM12	0.914316735	3.982000666	2.122727531
5271	SERPINB8	8.392477632	36.36196484	2.115261467
59352	LGR6	1.971518128	8.539579242	2.114858012
1001	CDH3	2863.457359	12336.09096	2.107055284
2247	FGF2	648.5499258	2789.117203	2.104519016
341640	FREM2	12.81379386	54.95355105	2.100515023
340061	TMEM173	28.65625468	122.7490664	2.098790083
55567	DNAH3	128.8394405	550.0531972	2.093996852
11309	SLCO2B1	3.385858371	14.39003354	2.087476422
203430	RTL3	0.873311171	3.707442818	2.085856741
8638	OASL	28.7350443	121.863596	2.084384039
4103	MAGEA4	12.11853517	51.08664541	2.075730881
389257	LRRC14B	8.78339576	36.86060343	2.069228974
1840	DTX1	101.205533	422.4774981	2.061586338
338596	ST8SIA6	7.7160616	32.13384793	2.058157184
4741	NEFM	1.267771815	5.269873307	2.055473179
5787	PTPRB	44.25331624	183.7092297	2.053566634
9478	CABP1	41.54903802	172.2954686	2.051997779
58489	ABHD17C	4.717999039	19.54407398	2.0504842
144811	LACC1	3.970037276	16.41804226	2.048057647
79819	WDR78	9.70422672	40.0012514	2.043359973
11077	HSF2BP	16.48671713	67.40600263	2.031572917
54453	RIN2	66.78098977	271.6973747	2.024491244
144347	RFLNA	60.90502387	245.5419747	2.011336529
5801	PTPRR	0.848450493	3.40648401	2.005381049
100287482	SMKR1	15.49466517	62.16638451	2.004363097
84541	KBTBD8	3.27971173	13.12857835	2.00106978
3754	KCNF1	3.295776198	13.11389671	1.992406253
10202	DHRS2	19.5085829	77.56121506	1.99122642
57096	RPGRIP1	1.979658396	7.867751999	1.990699978
642968	FAM163B	1.917940717	7.62022801	1.990276038
11023	VAX1	12.43852624	49.37557112	1.988981876
627	BDNF	34.06358642	135.1341425	1.988089983
861	RUNX1	19.93684813	78.8377514	1.983449281
9235	IL32	17.8808386	70.58374455	1.98092157

23052	ENDOD1	17.13283179	67.5268112	1.978696805
684	BST2	11.3984425	44.88583954	1.977423674
9619	ABCG1	91.516517	357.8260608	1.967154413
1825	DSC3	157.691294	613.7575872	1.960565942
7043	TGFB3	81.61519489	316.8135595	1.956724404
55509	BATF3	34.38101489	133.4536602	1.956654837
85444	LRRCC1	23.87444285	92.66516194	1.956559984
286133	SCARA5	6.669704025	25.88072431	1.956183347
84665	MYPN	19.5059903	75.6315616	1.955071163
23670	TMEM2	36.80653219	142.6761672	1.954710632
85463	ZC3H12C	3.992820975	15.4594962	1.953013012
8811	GALR2	5.671137233	21.95044041	1.952539913
3199	HOXA2	31.96598226	123.490159	1.949786746
10586	MAB21L2	1.549884433	5.987080021	1.949691907
50651	SLC45A1	97.20036095	375.4558392	1.949609653
2296	FOXC1	1492.844631	5762.867058	1.948722716
4674	NAP1L2	1.551618724	5.975705145	1.945334873
22998	LIMCH1	36.40623608	140.1969091	1.945197044
22861	NLRP1	1.557570913	5.972761321	1.939100223
57522	SRGAP1	750.4228505	2876.202341	1.938389509
80243	PREX2	100.6535686	385.7651208	1.938324385
3204	HOXA7	162.7263589	622.4476186	1.935504472
2342	FNTB	117.6925424	450.1436808	1.93536266
284076	TTLL6	154.9003312	591.6355947	1.933368623
100129654	TCF24	22.85280671	87.17629101	1.931564461
117581	TWIST2	2.257315958	8.608495317	1.931152723
51332	SPTBN5	14.1257135	53.79724433	1.929208534
55466	DNAJA4	40.45220359	153.7109374	1.925929625
54947	LPCAT2	102.9195833	390.8655828	1.925155035
26030	PLEKHG3	211.6861861	803.5582568	1.924475494
3965	LGALS9	11.74850662	44.55300693	1.92304542
79983	POF1B	2195.781474	8295.389688	1.917575272
2104	ESRRG	184.4026792	696.574502	1.917418047
2334	AFF2	499.4472375	1882.113015	1.913949078
24147	FJX1	0.82516551	3.103043541	1.910928514
339977	LRRC66	4.629443024	17.40763147	1.910809383
340156	MYLK4	5.000153453	18.69104503	1.902302957
4330	MN1	1031.405722	3851.027018	1.900631291
57144	PAK5	0.834009966	3.093293122	1.891007021
85352	SHISAL1	13.08445208	48.46517448	1.889094934
7476	WNT7A	1.191302922	4.387874437	1.880981938
27328	PCDH11X	1.96907076	7.250892976	1.880643724
9568	GABBR2	20.13055788	74.05090599	1.879130232

57549	IGSF9	2.250922173	8.251302383	1.874105677
6926	TBX3	5.932216478	21.72926446	1.872996189
63895	PIEZO2	1.546161096	5.661675031	1.872538301
84456	L3MBTL3	22.55878143	82.59831071	1.872423137
390205	LRRC10B	14.37146443	52.45398815	1.86784539
55190	NUDT11	5.589280938	20.39337775	1.867366151
140876	RIPOR3	428.0259364	1561.244439	1.866926309
5396	PRRX1	14.3971816	52.39696957	1.863696957
56937	PMEPA1	7.732425646	28.08128107	1.860615792
139716	GAB3	4.32194507	15.69401849	1.860462163
127294	MYOM3	94.10263935	341.2001121	1.858311028
91624	NEXN	243.9152992	881.4633971	1.853520411
10411	RAPGEF3	2.572785102	9.278523348	1.850564265
153572	IRX2	28.96954051	104.4017718	1.849537489
26230	TIAM2	78.39783432	281.9636501	1.84662348
94115	CGB8	0.850473716	3.058744638	1.846601113
29943	PADI1	11.32924617	40.70160282	1.845033739
255231	MCOLN2	8.038882715	28.84773633	1.843391208
9645	MICAL2	65.12731276	233.0687327	1.839420867
6840	SVIL	632.0435723	2258.934334	1.837546409
23708	GSPT2	98.21828638	350.8390776	1.836745892
163154	PRR22	12.6907466	45.24222494	1.833892935
8435	SOAT2	1.509783657	5.381406702	1.833641508
5507	PPP1R3C	73.81084259	261.9487301	1.827379804
9052	GPRC5A	33.69583176	119.4849376	1.826186718
401335	C7orf65	2.904846982	10.28423601	1.823900549
140862	ISM1	6.024245764	21.32682107	1.823816404
105372978	LINC01638	9.078043987	32.11964421	1.823002528
388585	HES5	204.3701972	722.2328738	1.821279262
2298	FOXD4	1.877813647	6.633763186	1.820773613
1066	CES1	0.795786388	2.808415105	1.819303066
4100	MAGEA1	0.794832369	2.802736902	1.81811379
80144	FRAS1	1.159019404	4.086499808	1.817960947
401387	LRRD1	1.882636533	6.632993252	1.816905562
26507	CNNM1	462.5827646	1625.741314	1.813314297
151742	PPM1L	19.80864944	69.54173645	1.811748571
198437	LKAAEAR1	24.68025169	86.32760032	1.806464779
27286	SRPX2	1.16919223	4.089415031	1.806382342
7104	TM4SF4	0.803025702	2.805996083	1.804994927
399939	TRIM49D1	1.599676646	5.586145878	1.804072937
25858	CATSPERZ	3.882553977	13.55696822	1.803956694
64135	IFIH1	32.946417	114.9223122	1.802465442
3009	HIST1H1B	1.535311982	5.337364547	1.797595705

3625	INHBB	1.833176234	6.368086577	1.796514462
102724428	SIK1B	323.3640074	1122.187566	1.795082822
389692	MAFA	42.76814141	148.3353696	1.794254222
79656	BEND5	0.805845942	2.793535257	1.793516067
57094	CPA6	40.06534716	138.854569	1.793147767
64759	TNS3	2784.571281	9650.306918	1.793119602
653149	NBPF6	19.83060574	68.17348105	1.781481905
375759	C9orf50	4.330196674	14.83017758	1.776031415
220963	SLC16A9	1.484419718	5.077804167	1.774305686
387723	C10orf143	3.882081778	13.21990118	1.767808978
8329	HIST1H2AI	12.87768172	43.81678016	1.766610575
84074	QRICH2	44.75929165	152.1988063	1.765697933
3918	LAMC2	7.33945037	24.90214225	1.762525925
7424	VEGFC	12.02456753	40.73809436	1.760393488
7771	ZNF112	45.07696898	152.5635723	1.758948115
79722	ANKRD55	1.498320268	5.058000968	1.755221278
83666	PARP9	104.8854732	354.0565673	1.755165
3973	LHCGR	41.70103078	140.4849093	1.752260216
4211	MEIS1	8.685024248	29.23065766	1.750880507
3202	HOXA5	20.81689287	70.0064746	1.749733609
6586	SLIT3	1.956545623	6.569883414	1.747559018
145946	SPATA8	7.942009088	26.64336232	1.746200241
5988	RFPL1	5.605949063	18.79791883	1.745542405
133396	IL31RA	8.65237322	28.88704448	1.739254803
644538	SMIM10	12.69311412	42.304851	1.736777041
27344	PCSK1N	709.1274566	2360.02821	1.734687243
146664	MGAT5B	269.9873263	897.5549534	1.733108586
102724265	LOC102724265	1.139015721	3.780684004	1.730859611
55715	DOK4	1274.275741	4227.646501	1.730177252
23705	CADM1	1207.566112	3997.002657	1.726816356
3207	HOXA11	24.15186229	79.93756364	1.726739166
2048	EPHB2	260.5216847	861.0767458	1.724738366
2172	FABP6	1.52899651	5.05070662	1.723900129
8357	HIST1H3H	44.31607524	146.2970795	1.722998947
203111	ERICH5	26.91584843	88.81024682	1.72227024
639	PRDM1	42.74272179	140.8437888	1.720345257
153571	C5orf38	19.41171601	63.90764307	1.719060822
340562	SATL1	2.536576788	8.336329467	1.716529462
57568	SIPA1L2	0.76349733	2.508252454	1.715987545
140733	MACROD2	41.78423317	136.8898241	1.711984642
84465	MEGF11	18.12498201	59.37666563	1.711916516
2919	CXCL1	1.154296809	3.778490965	1.710795935
3437	IFIT3	17.10183719	55.7250088	1.704173622

3756	KCNH1	3.868522429	12.58320892	1.701645336
3234	HOXD8	53.29267381	173.3272783	1.701489601
122402	TDRD9	0.768719919	2.498209572	1.70036455
57134	MAN1C1	124.842069	404.5660541	1.6962711
58191	CXCL16	237.1281752	767.9843282	1.695409779
118881	COMTD1	158.6111901	512.5095173	1.692084238
389432	SAMD5	55.87552545	180.1938575	1.689261435
23026	MYO16	0.779759207	2.510981275	1.687150683
83698	CALN1	95.15188121	304.7983714	1.679551109
729288	ZNF286B	20.83443797	66.7217333	1.679186585
146	ADRA1D	20.41621459	65.31359325	1.677667883
776	CACNA1D	2.20244727	7.037284936	1.675911449
3959	LGALS3BP	768.6863	2449.335556	1.671923574
2920	CXCL2	0.778571974	2.480819333	1.671914355
5139	PDE3A	999.6987413	3175.135467	1.667252835
494513	PJVK	10.66665182	33.87415913	1.667077738
2615	LRRC32	12.41654831	39.42341062	1.666788421
1956	EGFR	143.2788587	453.1442083	1.661144495
84867	PTPN5	2.843699093	8.987589043	1.660165345
51435	SCARA3	78.6678826	247.8163514	1.655424724
169355	IDO2	1.507473515	4.747316091	1.654979456
5328	PLAU	265.1475575	832.6047833	1.650836388
6421	SFPQ	1900.518036	5955.383993	1.647801818
961	CD47	8.622928296	26.95577391	1.644344542
27063	ANKRD1	28.51573757	89.11947047	1.643982311
401036	ASB18	2.959523504	9.213723766	1.638419431
286207	CFAP157	7.929666186	24.67246657	1.637569913
387849	REP15	3.527725395	10.96966632	1.636709474
10929	SRSF8	44.39668896	137.8655851	1.634738375
785	CACNB4	1.445068637	4.474389518	1.630552837
647310	TEX22	55.09103164	170.2350031	1.627638325
152485	ZNF827	8.222617394	25.36925188	1.62541137
59269	HIVEP3	31.59460514	97.35504623	1.623577525
7205	TRIP6	18.44877073	56.81235865	1.622680109
8483	CILP	2.823520571	8.675311319	1.619420388
388849	CCDC188	6.239987786	19.15999604	1.618482152
55068	ENOX1	3.480737287	10.68209032	1.617729154
360	AQP3	13.65181268	41.79096132	1.614098422
23569	PADI4	2.507206563	7.669675804	1.613084735
26115	TANC2	2025.682952	6158.791973	1.604239009
1991	ELANE	44.51999356	135.1985673	1.602554574
91050	CCDC149	62.9594576	191.0849984	1.601719503
221061	FAM171A1	148.1818474	448.1467061	1.596602367

83595	SOX7	27.46346221	83.02377018	1.596010935
25805	BAMBI	32.23712836	97.39361625	1.595103976
164633	CABP7	9.991012277	30.17827185	1.59480743
10257	ABCC4	809.4772145	2440.488802	1.592107756
94032	CAMK2N2	17.00278399	51.23459662	1.591347344
8022	LHX3	2.791334689	8.403085828	1.589964102
50801	KCNK4	18.0161097	54.17209503	1.588262369
3908	LAMA2	3130.495051	9405.966152	1.58718532
10892	MALT1	182.1298778	546.9385279	1.586411082
115708	TRMT61A	140.0445143	420.2810399	1.585468901
467	ATF3	116.612975	349.4101297	1.583193114
79922	MRM1	67.34604607	201.6056932	1.581871228
7851	MALL	6.611735516	19.79188329	1.581807978
219621	CABCOCO1	2.471147644	7.385036004	1.579423744
23600	AMACR	656.4730018	1954.262887	1.573816967
3215	HOXB5	89.68572926	266.6588368	1.57204479
6583	SLC22A4	15.63590057	46.30901241	1.566430674
116496	FAM129A	477.304309	1413.401091	1.566189662
23678	SGK3	104.2399383	308.3802945	1.564802445
64065	PERP	57.50689221	170.0316251	1.563996327
8382	NME5	6.167960509	18.23606556	1.563929068
51315	KRCC1	98.06105843	289.813163	1.563370883
161247	FITM1	4.842275492	14.29462688	1.561715897
84159	ARID5B	221.8389794	654.7027592	1.561327175
9573	GDF3	6.179770063	18.23226106	1.560868423
117248	GALNT15	4.838806725	14.26894895	1.56015585
145376	PPP1R36	29.14921609	85.92056529	1.5595464
2100	ESR2	5.185162175	15.27460022	1.558673603
116441	TM4SF18	4.616463981	13.5763933	1.556240129
4675	NAP1L3	11.69924951	34.38497517	1.555362318
8372	HYAL3	21.08355521	61.95020688	1.554990938
9540	TP53I3	434.0086386	1273.152394	1.552609454
27129	HSPB7	91.41368387	267.5359482	1.54925071
84225	ZMYND15	5.540746476	16.21324466	1.549020576
6441	SFTPD	16.38195785	47.87823165	1.547262081
23017	FAIM2	3.788416867	11.01639502	1.539985202
143941	TTC36	12.23628078	35.58202628	1.539983552
2780	GNAT2	5.139830626	14.94428705	1.539801347
10395	DLC1	2.44586975	7.110079559	1.539518125
124056	NOXO1	7.846311876	22.80444696	1.539228597
8970	HIST1H2BJ	16.06733429	46.60083731	1.536225283
1303	COL12A1	180.8801457	524.1448897	1.534931612
23362	PSD3	628.9012074	1817.277794	1.53087366

64067	NPAS3	486.8071221	1406.434444	1.530620128
117531	TMC1	17.3965762	50.20619109	1.52906188
24138	IFIT5	103.2181088	297.7741502	1.528522417
862	RUNX1T1	299.8781838	864.7015344	1.52782568
3623	INHA	3.138624079	9.038628801	1.525971682
3371	TNC	2.795838367	8.049252148	1.525573791
729262	NUTM2B	26.65012434	76.58889292	1.522992921
2944	GSTM1	9.682105436	27.82225383	1.522846585
695	BTK	12.25509267	35.21565855	1.522835669
2621	GAS6	355.2331889	1020.233634	1.522061286
9586	CREB5	2.468110821	7.075656403	1.519456817
84460	ZMAT1	82.91033159	236.9676042	1.515066048
10150	MBNL2	509.3878843	1455.649252	1.51482622
54437	SEMA5B	3.165263241	9.043639288	1.514577963
343702	XKR7	44.72688778	127.7858567	1.514513888
4313	MMP2	65.41218517	186.4409683	1.511087596
7475	WNT6	216.1573239	614.0292529	1.50622567
57562	CEP126	101.0256476	286.1011376	1.501803635
56912	IFT46	97.7156281	276.7190578	1.501760784
1769	DNAH8	20.0044669	56.49534068	1.497809707
10761	PLAC1	65.40491536	184.5279809	1.496368629
50632	CALY	597.8080249	1686.15876	1.495986211
55930	MYO5C	172.350097	484.4201	1.490916618
55106	SLFN12	26.80405075	75.33214439	1.490814553
1311	COMP	96.21158953	269.9429655	1.488372027
83849	SYT15	51.10265489	143.3657695	1.488230453
219670	ENKUR	278.9426427	782.3098772	1.48777168
4855	NOTCH4	46.72658744	130.814417	1.485205966
2257	FGF12	432.5855589	1210.702316	1.484786774
2202	EFEMP1	37.26270306	104.0001006	1.480780687
219348	PLAC9	8.175719424	22.80730516	1.480078401
161145	TMEM229B	26.1303335	72.88706609	1.479937291
10083	USH1C	7.837247253	21.82446199	1.477527172
6274	S100A3	3.141890246	8.736501125	1.475422827
124936	CYB5D2	165.1796943	459.0206845	1.47452282
7306	TYRP1	597.7686801	1660.841867	1.474255503
144448	TSPAN19	3.756578426	10.39525943	1.468434636
101928841	LOC101928841	21.11579134	58.4295603	1.468376118
2346	FOLH1	343.1932328	948.9360638	1.467289781
771	CA12	310.7219252	856.157075	1.462251461
7594	ZNF43	31.80151925	87.59593372	1.461768211
387885	CFAP73	16.25303776	44.74890925	1.461143128
6299	SALL1	6.881870632	18.8923374	1.456928529

2669	GEM	26.07757338	71.48807976	1.454893073
283948	NHLRC4	28.08202016	76.83641102	1.45214341
2042	EPHA3	2431.414304	6645.578844	1.450599122
5332	PLCB4	131.764001	360.0523727	1.450250506
9806	SPOCK2	7.866769529	21.49361848	1.45006516
284086	NEK8	72.66611869	198.2822842	1.448201029
56660	KCNK12	18.32553246	49.89990971	1.445182088
100130705	LOC100130705	24.38693444	66.35399264	1.444074862
54809	SAMD9	4.173048187	11.34329271	1.442665999
11219	TREX2	6.479820035	17.60276364	1.441776299
1050	CEBPA	78.40110521	212.8167423	1.440665755
1281	COL3A1	56145.41463	151869.3905	1.435591013
79822	ARHGAP28	64.09247132	173.2926246	1.43498345
122773	KLHDC1	32.80821192	88.59242451	1.433126372
1280	COL2A1	11.58355494	31.26523658	1.432481356
414152	C10orf105	23.3804152	63.1016476	1.432377125
3604	TNFRSF9	49.34802281	133.091881	1.431358377
161394	SAMD15	13.23316205	35.6292029	1.428902374
284098	PIGW	118.9050301	319.6159374	1.426529602
154214	RNF217	59.57348967	159.7284452	1.422878881
5739	PTGIR	5.474531379	14.64692486	1.419790421
166614	DCLK2	53.02490156	141.6307375	1.417392459
55824	PAG1	1937.733322	5175.465156	1.417318498
3592	IL12A	4.763319812	12.71933907	1.416984386
2006	ELN	30.52746895	81.41376933	1.415164835
2736	GLI2	265.0134373	706.0560766	1.413717258
54865	GPATCH4	207.1555138	551.0034487	1.411347128
219699	UNC5B	93.59714126	248.9216076	1.411155097
330	BIRC3	93.97731723	249.5942809	1.409200388
219595	FOLH1B	33.94538429	89.99711227	1.406663293
2878	GPX3	78.53395237	208.1527262	1.406254043
3399	ID3	1305.187829	3457.832007	1.40561034
11182	SLC2A6	141.3161645	374.3527192	1.405471736
79148	MMP28	12.55487964	33.25835199	1.405468484
79930	DOK3	17.92980454	47.39198942	1.402283463
84532	ACSS1	73.01528206	192.936862	1.401858451
5409	PNMT	5.795996014	15.30971319	1.401318748
114881	OSBPL7	26.78553352	70.64724111	1.39917919
9725	TMEM63A	257.7457721	679.2107784	1.39791059
8321	FZD1	97.29089544	256.1404239	1.396558248
4137	MAPT	913.7705993	2401.175272	1.393836786
81706	PPP1R14C	108.4762469	282.8711414	1.38276583
1602	DACH1	377.0358358	983.1851279	1.38276144

8819	SAP30	264.0914905	688.1133021	1.381608317
93183	PIGM	137.8318451	359.019303	1.381152162
255374	MBLAC1	37.29113544	97.0769948	1.380296723
8991	SELENBP1	38.88080359	101.1152229	1.378870269
55520	ELAC1	22.99716724	59.79518035	1.378573041
79884	MAP9	186.7003165	484.561285	1.375954769
342132	ZNF774	31.8868478	82.72119372	1.375295519
10475	TRIM38	5.414252061	14.03136289	1.373821188
1830	DSG3	25.37070271	65.69162541	1.372545983
57526	PCDH19	4.415466006	11.42858305	1.372008932
11155	LDB3	15.2804012	39.54680928	1.371878877
64072	CDH23	440.940533	1137.396055	1.367078699
53335	BCL11A	184.0430237	473.8373677	1.364348912
494143	CHAC2	32.41856781	83.39510506	1.363142349
347731	LRRTM3	39.21281207	100.6682017	1.360211037
85301	COL27A1	33.81892775	86.72138061	1.358556806
128434	VSTM2L	462.7751624	1184.583141	1.355996118
28951	TRIB2	270.6897627	692.1694436	1.354484927
729920	ISPD	38.86166294	99.36273935	1.354357311
375607	NAT16	5.750480459	14.69446836	1.353518759
54738	FEV	19.38365851	49.51188091	1.352933864
9464	HAND2	323.5746221	824.0697375	1.34866797
8407	TAGLN2	980.5501836	2491.830852	1.345542767
57596	BEGAIN	247.2560596	627.5859077	1.343807079
57125	PLXDC1	109.6648059	277.4935769	1.339353773
29118	DDX25	17.93660112	45.35504285	1.338356433
1647	GADD45A	9.561784105	24.09675226	1.333486978
348013	TMEM255B	15.19039773	38.22437314	1.331333198
9053	MAP7	4.404184923	11.07947217	1.330942201
388115	CCDC9B	29.79834422	74.94098435	1.33052276
2149	F2R	332.8364351	836.0490214	1.328774165
53342	IL17D	49.95601291	125.2949697	1.326598253
283130	SLC25A45	106.3850884	266.7577968	1.32623449
9454	HOMER3	216.8821761	543.4883559	1.325337632
643699	GOLGA8N	128.0392178	320.7080662	1.324674871
375519	GJB7	115.1522513	288.0091294	1.322571927
340075	ARSI	91.58620722	229.01565	1.322243938
80231	CXorf21	5.085041821	12.68643709	1.318955409
166348	KBTBD12	7.183507327	17.9142929	1.318350786
503542	SPRN	7.085236472	17.64962575	1.316749683
11067	DEPP1	6.810846842	16.9437776	1.314849463
7504	XK	6.437073808	16.00695277	1.314221774
408	ARRB1	1128.898332	2804.975823	1.313072773

9469	CHST3	654.3339686	1623.325345	1.310853099
150	ADRA2A	84.02192982	208.4175824	1.310639163
7781	SLC30A3	15.93860721	39.51986581	1.310052482
8787	RGS9	61.53408542	152.4403581	1.308787217
150082	LCA5L	12.15271379	30.04188828	1.305696982
26499	PLEK2	9.781330256	24.16974007	1.305099369
100133941	CD24	150.3227298	371.3094547	1.30455888
4060	LUM	16.9297828	41.79549615	1.303784023
257044	CATSPERE	7.416195403	18.30159927	1.303218561
56521	DNAJC12	16.54927244	40.82617397	1.302726579
286097	MICU3	43.8852527	108.0396892	1.299753275
23780	APOL2	191.4008141	471.1157107	1.299484478
63950	DMRTA2	71.27044006	174.7454201	1.293878907
22801	ITGA11	79.02267313	193.7310306	1.2937165
9734	HDAC9	4222.797732	10351.13834	1.293518383
85315	PAQR8	167.0341183	409.2646427	1.292891218
7957	EPM2A	33.13576453	81.18259654	1.292781277
441273	SPDYE2	7.852316352	19.20838686	1.290546163
3196	TLX2	172.3438823	421.2973495	1.289548751
159371	SLC35G1	84.06422826	205.1582949	1.287173558
133383	SETD9	103.3478646	252.113285	1.286563562
84875	PARP10	58.22026694	141.9557035	1.285847455
2022	ENG	264.1077157	643.809875	1.285508256
767	CA8	5.454602198	13.29589483	1.285434985
2537	IFI6	866.6032873	2109.518582	1.283470181
57189	KIAA1147	316.6883861	770.6727047	1.283054335
60529	ALX4	581.0988159	1412.119235	1.281006491
643008	SMIM5	6.150282565	14.94142852	1.280593488
4643	MYO1E	40.18739654	97.56519032	1.279623391
8365	HIST1H4H	8.080642987	19.60729769	1.278848715
79642	ARSJ	496.3734524	1203.957923	1.27828711
3707	ITPKB	107.0069787	259.5090269	1.278079836
116443	GRIN3A	8.468491071	20.53717978	1.278061245
4482	MSRA	25.88060467	62.71852557	1.27702032
158135	TTLL11	22.67168732	54.93022583	1.276708457
4254	KITLG	68.26063869	165.2291281	1.275342222
23641	LDOC1	157.7128915	381.1350404	1.27300166
1747	DLX3	24.41980634	58.98412514	1.272274964
389799	CFAP77	10.14076208	24.48225434	1.271570333
51298	THEG	9.476984336	22.8672212	1.270781103
83468	GLT8D2	211.2501517	508.871716	1.268349629
91768	CABLES1	1192.30918	2871.357207	1.267974426
84163	GTF2IRD2	68.21880343	164.1075882	1.266400594

10922	FASTK	775.9039307	1865.927209	1.265942767
221662	RBM24	401.5760097	964.8897692	1.264691051
3198	HOXA1	50.96375891	122.4177972	1.264269719
84168	ANTXR1	11.45842206	27.48886114	1.262438753
8029	CUBN	581.1530343	1390.878184	1.259006049
91010	FMNL3	744.8135679	1780.797193	1.257571965
6752	SSTR2	24.71017716	58.94426884	1.254246192
5971	RELB	160.5051095	382.7582778	1.253814355
151636	DTX3L	255.4597587	609.1469764	1.253694318
5920	RARRES3	30.11148035	71.55041769	1.248646553
339145	FAM92B	32.08561353	76.21401112	1.248129676
64116	SLC39A8	151.5374685	359.647059	1.246907254
2171	FABP5	126.7390218	300.751804	1.246712605
1007	CDH9	6.740811665	15.98669547	1.245877535
60677	CELF6	15.30863963	36.27894367	1.244786363
7399	USH2A	709.1993407	1680.430878	1.2445681
83445	GSG1	45.23216565	107.0431135	1.242771007
130752	MDH1B	14.81390134	35.03616046	1.241893048
155185	AMZ1	9.817039805	23.21627659	1.241776642
80830	APOL6	71.159016	168.1837793	1.240920104
283417	DPY19L2	59.43744841	140.4071578	1.240172396
55107	ANO1	11.52156612	27.18834749	1.238651631
1847	DUSP5	66.28423386	156.316387	1.237731365
91608	RASL10B	207.6632599	489.6318836	1.237451511
53637	S1PR5	672.5947841	1584.071111	1.235827605
2812	GP1BB	187.2844202	440.8202119	1.234959485
8814	CDKL1	53.77823324	126.3519372	1.232353519
55349	CHDH	14.18533427	33.32752904	1.232314211
54463	RETREG1	30.76996303	72.26102193	1.231694948
51296	SLC15A3	10.46978629	24.56401924	1.230314644
55231	CCDC87	11.45118842	26.85650928	1.22977447
79690	GAL3ST4	122.5564848	287.3689394	1.229457312
11142	PKIG	1212.43097	2841.126244	1.228560329
5805	PTS	121.1977423	283.7883583	1.227452584
55924	FAM212B	52.27397947	122.3348691	1.226670778
124976	SPNS2	32.72866846	76.55604323	1.225961358
51226	COPZ2	18.8864153	44.17561802	1.225901418
10325	RRAGB	104.9167766	245.351525	1.22560485
286319	TUSC1	63.77532319	149.082721	1.225042846
51764	GNG13	27.33758212	63.80913514	1.222877331
55332	DRAM1	229.4226422	535.360012	1.222501602
4862	NPAS2	9.132520702	21.26757674	1.219570636
27092	CACNG4	28.07069216	65.35665151	1.219269432

9310	ZNF235	42.84602787	99.68674578	1.218240236
57727	NCOA5	791.8104944	1841.060304	1.217309789
54505	DHX29	1166.256877	2711.170751	1.21703039
170302	ARX	28.34176042	65.81851716	1.215564152
91978	TPGS1	137.5356525	319.396531	1.215542995
4994	OR3A1	6.76671438	15.70289778	1.214503416
9423	NTN1	392.6250735	910.8532061	1.214066259
54898	ELOVL2	25.96389629	60.21249414	1.21355598
3675	ITGA3	479.9398059	1111.697056	1.21183832
25789	TMEM59L	739.3437764	1711.515553	1.21095716
497190	CLEC18B	14.48054728	33.38328653	1.205009864
11103	KRR1	681.312827	1569.083377	1.203532742
6330	SCN4B	11.10098812	25.54937099	1.202599674
59283	CACNG8	192.7466074	443.4251544	1.201985146
114898	C1QTNF2	7.102307233	16.31840679	1.200140535
51316	PLAC8	103.26603	237.1587571	1.199487392
80216	ALPK1	118.5685646	272.094546	1.198386471
55500	ETNK1	475.4366029	1089.744543	1.196665097
2905	GRIN2C	6.016354474	13.77953053	1.195565261
83743	GRWD1	333.4070523	761.1381101	1.190873637
4923	NTSR1	14.48984081	33.06504727	1.190265219
56413	LTB4R2	16.52445092	37.69442033	1.189748652
375057	STUM	48.56451211	110.6586836	1.188142293
60680	CELF5	145.7330542	332.0574209	1.188104603
57460	PPM1H	9.066838478	20.65031405	1.187492233
10855	HPSE	80.93092497	184.2493622	1.186896636
860	RUNX2	93.43033906	212.6647736	1.186618072
8347	HIST1H2BC	40.12427864	91.27744394	1.185782936
3205	HOXA9	16.53749994	37.6096471	1.185361618
29103	DNAJC15	719.1913668	1635.491535	1.185276684
10610	ST6GALNAC2	178.2538344	405.0281406	1.184089037
9379	NRXN2	67.84102178	154.1182332	1.183807748
55243	KIRREL1	1140.914235	2588.333839	1.181833361
200958	MUC20	33.05341853	74.97722462	1.181652934
285368	PRRT3	123.5652071	280.1528899	1.180941801
160857	CCDC122	60.44138796	137.0057961	1.180628233
3709	ITPR2	572.1418412	1293.503797	1.17683953
374618	TEX9	27.67351807	62.55386207	1.176592897
283008	NUTM2E	69.79357347	157.7307508	1.176297847
54149	C21orf91	12.47494397	28.16017344	1.174622884
2150	F2RL1	390.4959087	880.5587409	1.173111814
9263	STK17A	261.1598253	588.6051301	1.172367135
23466	CBX6	3880.489082	8741.311901	1.171611321

26519	TIMM10	111.9305206	252.1381096	1.171610716
3363	HTR7	17.17107837	38.64689498	1.170371862
84182	MINDY4	91.22584891	205.0358228	1.168361416
283576	ZDHHC22	39.18464285	87.98774101	1.167014185
9659	PDE4DIP	377.5068137	845.8391198	1.163880602
56134	PCDHAC2	121.1882245	271.4972916	1.163688282
399979	SNX19	211.9378827	474.7567169	1.163546928
137872	ADHFE1	8.061999027	18.04766095	1.162602356
201514	ZNF584	146.8447706	328.71764	1.162556988
54855	FAM46C	20.28726933	45.3968263	1.16201675
3751	KCND2	12.14156424	27.15775772	1.161410068
374882	TMEM205	929.330656	2078.662637	1.161391726
857	CAV1	691.3782186	1544.59025	1.159677111
727832	GOLGA6L6	8.072780174	17.99502262	1.156460404
141	ADPRH	209.2079471	466.1470479	1.155847475
80258	EFHC2	18.55924859	41.30780318	1.154276036
11247	NXPH4	27.68906553	61.55022952	1.152447871
55796	MBNL3	181.3350229	403.0257347	1.15221437
115207	KCTD12	25.02039915	55.58257332	1.151527824
84631	SLITRK2	321.3211048	713.7279399	1.151358508
4234	METTL1	151.8097008	337.032062	1.150621858
85369	STRIP1	761.7589761	1690.708522	1.150221461
362	AQP5	10.05974686	22.30589181	1.148830828
23057	NMNAT2	16.83394188	37.28781629	1.147331269
5731	PTGER1	278.9883808	617.7412182	1.146797558
169834	ZNF883	25.01768648	55.36631614	1.146060152
56147	PCDHA1	65.05112845	143.8808704	1.145228803
9331	B4GALT6	287.5256677	635.8172504	1.144921406
283212	KLHL35	59.66367194	131.9120529	1.144651716
363	AQP6	17.49581622	38.61366636	1.142101574
151195	CCNYL1	3152.723244	6949.963684	1.140406908
692312	PPAN-P2RY11	147.435087	324.8260697	1.139587523
5205	ATP8B1	51.62192259	113.6170698	1.138123822
6890	TAP1	446.6450277	981.746311	1.136221571
79815	NIPAL2	96.31274876	211.1720368	1.132620124
4995	OR3A2	33.77018691	74.02209069	1.13220572
11146	GLMN	288.6127798	632.1083298	1.131036643
84913	ATOH8	28.27195836	61.87060115	1.129882232
26609	VCX	89.95172529	196.4736059	1.127112657
7993	UBXN8	111.9918227	244.4480081	1.126134254
112849	L3HYPDH	122.3097744	266.900696	1.125763366
6041	RNASEL	13.10133827	28.56439123	1.124503596
57168	ASPHD2	33.03504319	72.0189129	1.124378592

4054	LTBP3	1615.631738	3521.426722	1.124061669
2762	GMDS	709.2997068	1545.770619	1.123858993
10808	HSPH1	1085.474319	2360.601379	1.120828849
340526	RTL5	695.7654458	1512.413878	1.120180057
1466	CSRP2	118.1016536	256.6865454	1.119978513
81831	NETO2	1118.360486	2427.837787	1.118286739
55970	GNG12	192.6477873	418.0780974	1.117806849
54482	TRMT13	200.8733682	435.6134672	1.116762252
197342	EME2	16.79608666	36.37230036	1.114715035
79132	DHX58	89.04885074	192.7598304	1.114135539
22846	VASH1	751.0012214	1624.588112	1.113186833
7041	TGFB1I1	614.717092	1329.026624	1.112375502
3958	LGALS3	101.4905657	219.3836217	1.112110199
10039	PARP3	135.7457537	293.3302134	1.111618609
348801	LNP1	34.05571264	73.56168794	1.111057762
3669	ISG20	18.93559478	40.88921891	1.110619766
54491	FAM105A	16.84616131	36.36989624	1.110324924
3176	HNMT	8.355060058	18.03726537	1.110258524
6352	CCL5	35.48547623	76.53808965	1.108949224
92154	MTSS1L	462.2877319	996.2027508	1.107648318
3875	KRT18	1199.029036	2582.501168	1.106902406
2116	ETV2	8.707062144	18.72506774	1.104713012
79841	AGBL2	10.38515448	22.32956192	1.104432271
91801	ALKBH8	180.2404187	387.3393646	1.103675557
4644	MYO5A	498.7374218	1070.244641	1.101588249
2113	ETS1	902.063875	1934.297299	1.100508053
55081	IFT57	180.2154634	386.2408689	1.09977802
5098	PCDHGC3	236.937112	507.0781279	1.097703858
114827	FHAD1	12.40339874	26.52519023	1.0966276
55638	SYBU	114.9929237	245.8464011	1.096212151
79080	CCDC86	425.172988	908.5903304	1.095580009
8701	DNAH11	520.008564	1109.072957	1.092746983
7168	TPM1	3424.153722	7280.69483	1.088328669
84698	CAPS2	92.09898193	195.8168889	1.088248087
91461	PKDCC	166.385813	353.7458679	1.08818087
133	ADM	81.61653687	173.4382321	1.087488555
55269	PSPC1	610.8434233	1297.857631	1.087257606
5954	RCN1	3096.951422	6576.072723	1.0863775
51776	MAP3K20	401.4319764	852.0857269	1.085843044
399697	CTXN2	11.7405939	24.91726648	1.085640419
83959	SLC4A11	73.2157508	155.2420019	1.08429299
79800	CARF	43.11343954	91.40944422	1.084205565
9289	ADGRG1	222.1137897	470.303814	1.082294068

153769	SH3RF2	255.8343569	541.4836622	1.081707787
5699	PSMB10	79.62263082	168.4704099	1.081244773
3663	IRF5	120.0980557	254.1099014	1.081239796
282973	JAKMIP3	12.11309192	25.6007834	1.079620792
123099	DEGS2	181.9737281	384.448873	1.079061569
10148	EBI3	302.5207936	638.9352233	1.078635366
148545	NBPF4	138.416828	292.1824481	1.077850167
57205	ATP10D	303.6185685	640.7653356	1.077536078
375341	C3orf62	83.25823536	175.4652795	1.075520697
79446	WDR25	140.8737194	296.740514	1.074799416
221	ALDH3B1	326.6608252	687.9915516	1.074597399
79183	TTPAL	1010.86775	2126.356265	1.072789071
91807	MYLK3	193.9405701	407.7774083	1.07216722
56924	PAK6	20.81464929	43.75820751	1.071954391
388677	NOTCH2NL	261.1647266	548.4461385	1.070389887
163702	IFNLR1	92.40254636	193.8865335	1.06920809
83982	IFI27L2	356.5749828	747.9444558	1.068725649
57458	TMCC3	347.0653022	727.9544692	1.068641079
114757	CYGB	250.8603377	526.0099326	1.068205651
150786	RAB6D	15.78898467	33.07829372	1.066966418
115416	MALSU1	897.8645212	1878.569909	1.065065127
3090	HIC1	27.89247219	58.34677572	1.064777124
54487	DGCR8	323.2097548	675.7188188	1.063952294
9830	TRIM14	420.4460232	877.4789529	1.061443922
79400	NOX5	27.60426058	57.59769075	1.061120014
57542	KLHL42	261.4529204	544.162183	1.057485514
1159	CKMT1B	188.6988587	392.3618444	1.056099055
10587	TXNRD2	513.6035215	1067.490668	1.055496459
389524	GTF2IRD2B	135.0369209	280.4636205	1.054459735
124565	SLC38A10	2647.849018	5495.248707	1.053363915
145553	MDP1	93.51616694	193.9404456	1.052326001
7779	SLC30A1	717.7881576	1487.868291	1.051616795
161357	MDGA2	24.51446386	50.79027419	1.050919052
7266	DNAJC7	694.9398958	1438.007673	1.049111262
146691	TOM1L2	3897.386038	8062.59137	1.048736767
57545	CC2D2A	89.9627579	186.1041248	1.048710237
65983	GRAMD2B	127.0869103	262.8233578	1.048278054
11167	FSTL1	1385.922299	2865.261964	1.047820671
7552	ZNF711	1290.878025	2667.208218	1.046977767
4884	NPTX1	831.3413836	1715.205378	1.0448684
51306	FAM13B	339.5729174	700.4498177	1.044560294
5996	RGS1	11.74606817	24.22868452	1.044538161
2651	GCNT2	1350.997063	2781.836611	1.042013148

4647	MYO7A	129.6635856	266.9749354	1.04193093
283377	SPRYD4	315.9274914	650.3930239	1.0417183
55094	GPATCH1	178.7956444	367.8326854	1.040738091
54839	LRRC49	39.69073927	81.57150311	1.039262803
144100	PLEKHA7	85.31455072	175.1766571	1.037946819
6604	SMARCD3	995.6344354	2044.208612	1.037854397
5054	SERPINE1	89.3352231	183.2710844	1.036678164
216	ALDH1A1	12957.82665	26563.21959	1.035606256
6367	CCL22	11.36237574	23.29160478	1.035545526
149840	C20orf196	24.83611835	50.8049484	1.032529311
399726	CASC10	582.8951951	1191.753743	1.031777742
23371	TNS2	365.9440267	747.8499002	1.031125742
1277	COL1A1	9760.270947	19935.22859	1.030327046
6939	TCF15	46.59093093	95.10287013	1.029439724
6376	CX3CL1	21.2204692	43.3040843	1.029046546
493861	EID3	83.71007732	170.77217	1.028599669
285600	KIAA0825	19.15315966	39.03819753	1.027304032
64478	CSMD1	193.787351	394.8391963	1.02679081
51421	AMOTL2	361.9820495	737.4040716	1.026537226
360200	TMPRSS9	14.04641454	28.59921082	1.025773419
55506	H2AFY2	299.1441557	608.8784591	1.025313397
57571	CARNS1	160.1612032	325.7246271	1.024128084
6595	SMARCA2	91.37650185	185.75982	1.023543361
388407	C17orf82	39.35110187	79.97129953	1.023078299
284307	ZIK1	209.0887907	424.7211975	1.022400394
60598	KCNK15	126.9041583	257.7553232	1.02226288
56252	YLPM1	1207.342718	2452.021837	1.022136568
116835	HSPA12B	31.99091347	64.80161827	1.018367654
5621	PRNP	579.3734516	1172.95355	1.0175804
8611	PLPP1	4282.624752	8660.484897	1.015952531
55117	SLC6A15	1788.070747	3614.737808	1.015487186
84858	ZNF503	1416.86532	2862.542642	1.014594555
9498	SLC4A8	117.3363567	237.0522571	1.014555027
6284	S100A13	16.07661866	32.47756648	1.014479535
57540	DISP3	89.48221642	180.7108148	1.014009952
162963	ZNF610	96.85920104	195.5051134	1.013245334
54970	TTC12	151.6815861	305.6527391	1.01084754
548596	CKMT1A	479.9433743	966.4604489	1.009846492
387712	ENO4	26.88328438	54.09498107	1.008785341
91754	NEK9	1441.430065	2900.122087	1.008612792
9570	GOSR2	455.0524651	914.6756338	1.007227329
11346	SYNPO	522.8883206	1050.988145	1.007171645
2159	F10	35.41294614	71.13284306	1.006238955

817	CAMK2D	1893.227122	3802.650874	1.006157994
3725	JUN	816.6622313	1639.516653	1.005459142
64838	FNDC4	168.5157884	338.2273737	1.00510966
766	CA7	35.67115671	71.46524608	1.002483823
22809	ATF5	1314.280596	2631.441468	1.001579984
126119	JOSD2	241.8489916	483.8233566	1.000373895
5657	PRTN3	585.4436846	292.6018527	-1.000591496
2810	SFN	22.74274502	11.36438281	-1.000887062
100506658	OCLN	2497.084111	1246.82191	-1.001989005
7349	UCN	51.05586575	25.46652052	-1.003474862
6513	SLC2A1	3352.057249	1671.601222	-1.00381607
94274	PPP1R14A	3170.72881	1580.786624	-1.004171845
51170	HSD17B11	950.0901779	473.5417297	-1.004572888
10472	ZBTB18	65.82458082	32.73446985	-1.007815815
54765	TRIM44	138.3508856	68.72913048	-1.009338266
5789	PTPRD	37.72369061	18.71739788	-1.011090942
55355	HJURP	680.5834791	337.615288	-1.011391897
7976	FZD3	1452.557962	720.3064362	-1.011913033
55803	ADAP2	1359.923237	674.2584968	-1.012151517
9071	CLDN10	151.2236759	74.94561341	-1.012768084
57706	DENND1A	3434.293826	1701.817498	-1.012937145
6676	SPAG4	972.0651508	481.5178463	-1.013463742
6490	PMEL	141.1209248	69.8843975	-1.013889622
8749	ADAM18	445.845773	220.7296823	-1.014264092
8565	YARS	3164.896916	1565.802145	-1.015256586
55814	BDP1	1430.743525	707.2032951	-1.016568175
4804	NGFR	1213.175565	598.3307206	-1.019773302
57369	GJD2	29.82225434	14.66696401	-1.023819047
255043	TMEM86B	112.4256743	55.26946579	-1.024416963
201294	UNC13D	103.9654248	51.09437625	-1.024867405
55220	KLHDC8A	134.5832574	66.11618248	-1.025423614
5360	PLTP	3182.953596	1562.630365	-1.026389571
83999	KREMEN1	1187.283477	582.4653883	-1.027420207
9537	TP53I11	904.621218	443.5637138	-1.028172486
26261	FBXO24	76.62886523	37.56636525	-1.028446407
6496	SIX3	9570.377151	4688.657913	-1.029400757
54084	TSPEAR	199.2547572	97.6088976	-1.0295296
27244	SESN1	1422.034807	696.1633643	-1.030458979
27293	SMPDL3B	573.0381661	280.4975441	-1.030643091
5783	PTPN13	958.7315157	468.4044885	-1.033371957
2597	GAPDH	112825.4872	55094.22617	-1.03411997
10420	TESK2	841.9930372	411.0146857	-1.03461836
493	ATP2B4	6278.942487	3059.095933	-1.037416247

5309	PITX3	61.20139813	29.80877816	-1.03782737
151887	CCDC80	71.18975099	34.67180069	-1.03790679
57167	SALL4	2960.794372	1441.533321	-1.038380114
2760	GM2A	1592.159993	773.4779269	-1.04155329
79990	PLEKHH3	1493.643336	725.0788593	-1.042625875
4124	MAN2A1	1995.248712	967.4007428	-1.044383042
27074	LAMP3	122.3516781	59.31493614	-1.044566547
92610	TIFA	75.64211557	36.65780206	-1.045069426
121227	LRIG3	173.7390517	84.14293944	-1.046007945
126410	CYP4F22	166.3797148	80.5240419	-1.046988054
23779	ARHGAP8	385.2428885	186.3807604	-1.047515383
79925	SPEF2	62.16200574	30.07304404	-1.047562148
29766	TMOD3	3666.338567	1773.521436	-1.04772325
53616	ADAM22	150.3079607	72.67984897	-1.048294094
3655	ITGA6	583.4782249	281.0019415	-1.054098718
8724	SNX3	12392.06373	5966.548545	-1.054447943
11123	RCAN3	967.2811047	464.9377474	-1.056897657
5330	PLCB2	76.32936809	36.68801017	-1.056929588
3004	GZMM	19.62043743	9.424521683	-1.057865901
126375	ZNF792	245.3579796	117.8488814	-1.057950127
80237	ELL3	189.6689118	91.06477651	-1.058518191
54557	SGTB	560.2497096	268.9203357	-1.058891141
348093	RBPMS2	1980.195997	950.4523804	-1.058956981
4323	MMP14	47.66833489	22.85476066	-1.06053652
3589	IL11	66.85529621	32.0536227	-1.060554434
283248	RCOR2	1404.464318	672.410038	-1.062606805
3064	HTT	2468.881289	1181.775289	-1.062901731
9783	RIMS3	1152.892276	551.4348123	-1.063995464
326624	RAB37	31.43807169	15.02065955	-1.065564568
6606	SMN1	967.4480383	462.1851162	-1.065713375
7697	ZNF138	346.979207	165.7411537	-1.065917341
5106	PCK2	1362.906716	650.8791858	-1.066225136
558	AXL	4427.235494	2108.983258	-1.069858473
1013	CDH15	78.9986852	37.59177637	-1.071411552
7490	WT1	2675.748422	1271.70399	-1.073179579
56929	FEM1C	667.868443	317.2022042	-1.074161152
10848	PPP1R13L	1434.312485	680.9187397	-1.074804826
730394	GTF2H2C_2	996.9892168	473.0302225	-1.075645539
56123	PCDHB13	94.11652955	44.6503449	-1.075776804
100996741	LOC100996741	252.517181	119.7851296	-1.075932731
89122	TRIM4	278.9022831	132.2938777	-1.076013446
285489	DOK7	47.6461707	22.58779985	-1.076816517
257313	UTS2B	53.07535472	25.16092852	-1.076856945

29106	SCG3	198.319008	93.95844749	-1.07772818
94031	HTRA3	303.5887716	143.8319323	-1.077734427
64787	EPS8L2	113.3212644	53.66653271	-1.078324018
79629	OCEL1	916.8634658	433.7825505	-1.079734892
64403	CDH24	2131.938174	1008.170171	-1.080426426
11148	HHLA2	139.7301894	65.88622683	-1.084594942
10481	HOXB13	552.8413318	260.591666	-1.085074533
8553	BHLHE40	1036.97144	488.6767653	-1.085423744
3099	HK2	1041.805864	490.6554539	-1.086304261
5884	RAD17	1141.276649	537.3129117	-1.086814136
3998	LMAN1	4808.484268	2263.205493	-1.087214616
342897	NCCRP1	1038.676088	488.1213347	-1.089434103
6938	TCF12	3248.302199	1524.921503	-1.090950877
2591	GALNT3	27.09290484	12.70216093	-1.092841131
688	KLF5	1398.475062	655.5429493	-1.093092318
3156	HMGCR	1546.683064	724.990307	-1.093143988
124739	USP43	137.6436264	64.4898738	-1.093793256
4902	NRTN	291.8649482	136.6312332	-1.095013645
51274	KLF3	2034.168634	951.823516	-1.095673281
7423	VEGFB	1517.901933	709.4265401	-1.097353377
55197	RPRD1A	1659.475372	775.2143417	-1.098060053
8744	TNFSF9	152.0975534	71.03300547	-1.098435513
1757	SARDH	571.7470074	266.9807882	-1.098640979
4324	MMP15	2598.66848	1212.328565	-1.099991846
129025	ZNF280A	231.3782879	107.9119162	-1.100399308
7305	TYROBP	591.3674741	275.5567605	-1.101705373
23338	JADE2	3717.462889	1731.993996	-1.101884411
8434	RECK	793.8154341	369.4733094	-1.103333464
10397	NDRG1	728.0196923	338.7709984	-1.103667101
55435	AP1AR	542.5935896	252.3330398	-1.104542882
8702	B4GALT4	745.7931083	346.4977488	-1.105929486
9306	SOCS6	1585.020352	735.6073553	-1.107493555
2125	EVPL	132.6280449	61.53904098	-1.107812007
85414	SLC45A3	251.5993568	116.6994413	-1.10833058
54361	WNT4	25.33713457	11.74962066	-1.108639197
3590	IL11RA	726.4020447	336.5683209	-1.109868872
6920	TCEA3	3203.658117	1482.841727	-1.111355578
26050	SLITRK5	2232.4208	1031.756923	-1.113505874
5865	RAB3B	919.6458592	424.9467724	-1.113796265
54507	ADAMTSL4	2566.164619	1185.756306	-1.113806181
151556	GPR155	278.8839068	128.754832	-1.115038109
783	CACNB2	23.32377419	10.76751002	-1.115116595
3383	ICAM1	990.3827111	457.0007972	-1.115789448

8310	ACOX3	154.8751819	71.43877994	-1.11632663
79958	DENND1C	178.1254054	82.15853331	-1.116410965
333929	SNAI3	31.11750771	14.35260298	-1.116414109
339456	TMEM52	272.0267152	125.4550489	-1.11657781
1139	CHRNA7	408.2494635	188.241558	-1.116865824
144195	SLC2A14	178.1200351	81.92630447	-1.120451157
89832	CHRFAM7A	630.0624054	289.4204118	-1.122328056
22902	RUFY3	1433.647873	658.4788175	-1.122481781
54020	SLC37A1	421.477756	193.5757307	-1.122558406
10620	ARID3B	2365.305529	1086.305546	-1.122596602
5010	CLDN11	34.14032487	15.66710842	-1.123737851
286336	FAM78A	345.8165659	158.65221	-1.124139363
132864	CPEB2	150.826024	69.14282406	-1.125233942
348751	FTCDNL1	37.81861938	17.33036759	-1.125794441
7456	WIPF1	662.00318	303.1831193	-1.126648718
51027	BOLA1	64.50967476	29.47358842	-1.130092823
7008	TEF	716.5540523	326.6527049	-1.13331795
26503	SLC17A5	3450.698327	1572.80116	-1.133552063
9846	GAB2	3560.765939	1622.26126	-1.134181426
7525	YES1	11252.83541	5124.589175	-1.13478031
57393	CLTRN	40.17526206	18.28187549	-1.135893354
1821	DRP2	36.03355404	16.39637621	-1.135963956
644596	SMIM10L2B	86.41723704	39.30610666	-1.136565636
121643	FOXN4	228.536566	103.8684733	-1.13766719
9444	QKI	4712.039592	2140.87976	-1.13814789
54836	BSPRY	17.26342325	7.837733185	-1.139210206
401024	FSIP2	26.71760674	12.08962998	-1.144020693
55274	PHF10	387.3070508	174.92758	-1.146719995
57333	RCN3	1284.6169	579.6846597	-1.147997968
5033	P4HA1	9711.673078	4382.26538	-1.148043004
6691	SPINK2	34.75563283	15.67766438	-1.148536168
134548	SOWAHA	113.961384	51.37747807	-1.149337067
9749	PHACTR2	362.5984452	163.2763253	-1.15105712
8092	ALX1	231.504403	104.1302551	-1.152650327
26140	TTLL3	65.8543393	29.61348568	-1.153024192
6659	SOX4	678.0522103	303.9883083	-1.157380529
317649	EIF4E3	10526.5716	4717.628409	-1.157901949
116039	OSR2	870.7336345	389.9108852	-1.159087021
57462	MYORG	921.5079506	411.5369497	-1.162974645
79144	PPDPF	28106.20442	12545.49941	-1.163718737
7378	UPP1	5200.336674	2317.265117	-1.166181916
7545	ZIC1	1473.793132	656.4277364	-1.166825932
56154	TEX15	239.1995019	106.4769677	-1.167672994

55876	GSDMB	29.36562768	13.05697485	-1.169307795
58526	MID1IP1	716.5670152	318.4813963	-1.169892534
1382	CRABP2	26113.68383	11605.70001	-1.169972449
23038	WDTC1	3500.373254	1554.551251	-1.171010588
80853	KDM7A	715.1407047	317.0457409	-1.173536125
7163	TPD52	4704.741798	2077.726063	-1.179110093
8864	PER2	195.1454256	86.10662088	-1.180353567
3910	LAMA4	6634.052937	2926.148815	-1.180887382
8673	VAMP8	33.63215173	14.81447884	-1.182833209
84614	ZBTB37	54.29391077	23.90383354	-1.183548398
2979	GUCA1B	37.45890108	16.475044	-1.185026258
79816	TLE6	743.780132	327.0346002	-1.18543293
22875	ENPP4	94.74309137	41.64932748	-1.185727546
8786	RGS11	35.81607214	15.71334998	-1.188616343
3040	HBA2	28.35365317	12.42960987	-1.189753614
23593	HEBP2	473.046943	207.3564392	-1.189870508
4948	OCA2	137.5293697	60.23672023	-1.191024616
160622	GRASP	250.6489008	109.7544882	-1.19138797
283659	PRTG	3141.274996	1372.951048	-1.194070058
92270	ATP6AP1L	71.19373162	31.0952076	-1.195057973
9467	SH3BP5	358.080676	156.3624203	-1.195390844
9369	NRXN3	18.85894963	8.221384653	-1.197796026
10493	VAT1	8421.360408	3665.586721	-1.200009171
93426	SYCE1	78.24889043	34.03366395	-1.201107819
900	CCNG1	3334.284571	1449.737674	-1.201585367
126668	TDRD10	31.7760579	13.79333226	-1.203969125
56605	ERO1B	133.7581318	58.06061875	-1.203994751
6658	SOX3	13137.54469	5698.053983	-1.205154477
5757	PTMA	47007.30393	20387.97879	-1.205166181
654254	ZNF732	41.75889491	18.07712743	-1.207918091
93	ACVR2B	1098.828943	475.2762668	-1.20912855
9381	OTOF	18.24123085	7.853424129	-1.215809363
64946	CENPH	668.792603	287.8802439	-1.216090105
57491	AHRR	516.2333148	222.0289075	-1.217275725
728498	GOLGA8H	279.1858383	120.0335852	-1.217787636
9543	IGDCC3	216.8188502	93.01984139	-1.220879805
2966	GTF2H2	1124.577514	482.0940629	-1.221996537
6303	SAT1	2292.459966	981.8465887	-1.22332701
54852	PAQR5	75.07164615	32.11290752	-1.225114825
23216	TBC1D1	413.059227	176.5701388	-1.226107281
342346	C16orf96	62.07960212	26.51598721	-1.227256849
79792	GSDMD	52.31550897	22.32022523	-1.228887113
23114	NFASC	221.5705131	94.35059045	-1.231662447

1373	CPS1	10298.07024	4384.222239	-1.231981179
1043	CD52	19.2699914	8.178091756	-1.236519773
347735	SERINC2	517.6901068	219.5654254	-1.237437855
22915	MMRN1	1285.725586	544.1002613	-1.240638333
340371	NRBP2	2191.141015	924.9264118	-1.244271842
55818	KDM3A	3934.390351	1659.309536	-1.245557063
5077	PAX3	62.36878845	26.29911209	-1.245810142
401	PHOX2A	48.18526652	20.30548573	-1.246722546
100507003	GFY	28.92755507	12.169455	-1.249179833
27165	GLS2	230.6386891	97.01452438	-1.249361883
2056	EPO	31.36342751	13.17859298	-1.250886883
226	ALDOA	47936.26647	20120.66588	-1.252439498
1122	CHML	796.623469	333.6855846	-1.25540862
1950	EGF	536.9959913	224.8580211	-1.25589697
6572	SLC18A3	30.86123193	12.91284832	-1.256988387
7226	TRPM2	14.12935336	5.910416248	-1.257363798
10675	CSPG5	1034.104752	431.8778666	-1.259687047
1628	DBP	2177.821832	909.0623445	-1.260434787
8436	CAVIN2	69.47171054	28.84141003	-1.268283922
3600	IL15	526.1548436	218.1799391	-1.26996898
2264	FGFR4	644.0028742	267.0364162	-1.270030629
51092	SIDT2	5034.377145	2085.932476	-1.271120841
57519	STARD9	359.8875113	148.9864787	-1.272364635
22936	ELL2	492.088939	203.5614962	-1.273454388
158219	TTC39B	91.24615689	37.73735391	-1.27377053
3159	HMGA1	10201.89191	4218.955755	-1.273878858
131405	TRIM71	705.1503983	291.2354404	-1.275745068
100526835	FPGT-TNNI3K	29.35976685	12.11754682	-1.276742855
158038	LINGO2	399.2051323	164.4847464	-1.279176468
90634	N4BP2L1	265.0922596	109.1891357	-1.27966523
1021	CDK6	999.7706136	411.7642881	-1.27977841
8204	NRIP1	1935.979986	797.1528006	-1.280135842
8728	ADAM19	51.4614309	21.16788228	-1.281614628
84695	LOXL3	590.5823361	242.7221872	-1.282832216
23025	UNC13A	692.2570771	284.1266286	-1.284773849
90355	C5orf30	316.3362737	129.4394755	-1.28918133
55314	TMEM144	686.0249848	280.1727756	-1.291944344
440	ASNS	2187.970576	893.1238988	-1.292661104
6619	SNAPC3	1461.606312	596.3277217	-1.293377459
26085	KLK13	13.78318309	5.623027306	-1.293490145
60675	PROK2	45.88503219	18.70925911	-1.294271191
93099	DMKN	179.4630047	73.1595497	-1.294568371
151306	GPBAR1	49.94324568	20.3545409	-1.294938899

79041	TMEM38A	649.0996971	264.3157462	-1.296177709
2911	GRM1	791.5995043	321.9612066	-1.297883842
6405	SEMA3F	436.1909161	177.1729747	-1.299801168
9120	SLC16A6	126.08401	51.19268798	-1.300375659
83543	AIF1L	550.4724836	223.1936197	-1.302374663
4440	MSI1	2095.9178	849.6335113	-1.302669562
23452	ANGPTL2	42.32970714	17.12111137	-1.305894154
146206	CARMIL2	182.3044774	73.73009916	-1.306024392
55630	SLC39A4	445.6735835	180.2228765	-1.3062053
11217	AKAP2	3246.60702	1311.830345	-1.307351614
2118	ETV4	1825.032679	735.3910796	-1.311338715
9497	SLC4A7	336.5819785	135.5043966	-1.312618271
10434	LYPLA1	6379.517693	2567.023862	-1.31335065
9717	SEC14L5	116.5879513	46.89280876	-1.313980101
1392	CRH	17.21497568	6.921243387	-1.314561
8497	PPFIA4	345.2891457	138.6875273	-1.315966937
389125	MUSTN1	15.53145057	6.236519632	-1.316379533
85442	KNDC1	202.5345113	80.98035086	-1.322523961
5214	PFKP	1672.240797	667.8624395	-1.324159721
482	ATP1B2	8496.346096	3392.149679	-1.324642705
129293	TRABD2A	682.9360142	272.324914	-1.32642144
85407	NKD1	74.36611505	29.64979215	-1.326623415
8577	TMEFF1	160.8134446	64.11063789	-1.326752358
338761	C1QL4	1961.653501	781.8787759	-1.32705338
388588	SMIM1	230.2688397	91.6419088	-1.329239783
158405	KIAA1958	725.3975472	287.8217133	-1.333596434
221303	FAM162B	64.70204876	25.62003973	-1.336538682
23090	ZNF423	169.2136534	66.96135106	-1.337445439
8905	AP1S2	816.5308094	323.0644284	-1.337685414
3613	IMPA2	1187.381283	469.2965136	-1.339211629
53826	FXYD6	20.88404741	8.249377474	-1.340044181
7179	TPTE	820.261296	323.5348898	-1.342162257
55344	PLCXD1	11859.67741	4677.44105	-1.342273392
84699	CREB3L3	44.15651593	17.38558931	-1.344734374
65124	SOWAHC	1709.007716	672.3116824	-1.345956787
2119	ETV5	1182.581129	464.9522154	-1.346784802
9687	GREB1	180.3314196	70.81385095	-1.348547303
10461	MERTK	1087.844719	426.8141961	-1.349792572
83604	TMEM47	4413.137021	1731.361515	-1.349897545
9262	STK17B	819.1983198	321.2530378	-1.35050266
345895	RSPH4A	127.2494759	49.86512202	-1.351556729
57715	SEMA4G	1035.415389	405.7438435	-1.351568556
146540	ZNF785	58.64365547	22.97273911	-1.352052151

23235	SIK2	4232.013664	1657.082359	-1.352698977
6442	SGCA	98.1302606	38.37340793	-1.354591196
5167	ENPP1	258.2442144	100.6686826	-1.359121088
4438	MSH4	24.55089011	9.570033527	-1.359179448
9509	ADAMTS2	186.8708834	72.81197809	-1.359794089
340485	ACER2	53.91953663	21.00709242	-1.359931605
81618	ITM2C	6876.955855	2674.730639	-1.362376474
25816	TNFAIP8	191.7010181	74.49260386	-1.363688902
116969	ART5	25.51924207	9.900854784	-1.365960491
64220	STRA6	85.58580644	33.1779546	-1.367146612
25802	LMOD1	22.20415474	8.591978191	-1.369767416
115727	RASGRP4	437.2887553	169.1267755	-1.370481171
55753	OGDHL	801.508935	309.9313618	-1.370769855
147798	TMC4	176.0436201	67.95244009	-1.373335682
3398	ID2	5937.573846	2285.816097	-1.377164214
54532	USP53	3974.55968	1529.219164	-1.377999855
5144	PDE4D	844.9849071	324.9233923	-1.378825961
7857	SCG2	87.18116665	33.47763663	-1.380818826
54845	ESRP1	104.4302043	40.08016498	-1.381578691
6935	ZEB1	258.2316038	99.03377739	-1.382673003
51411	BIN2	10.4244497	3.997805213	-1.382691141
1028	CDKN1C	500.0117599	191.3125007	-1.386030881
11248	NXPH3	107.2222632	41.02037202	-1.386192011
1132	CHRM4	335.4399687	128.2025033	-1.387630169
115362	GBP5	862.3472661	329.5660222	-1.387701447
7855	FZD5	7051.403201	2694.65585	-1.387809347
8626	TP63	2011.46621	767.4227043	-1.39015415
54800	KLHL24	2471.777875	942.6840297	-1.39070291
2245	FGD1	8779.66453	3340.283454	-1.394195282
55636	CHD7	2223.471433	843.7790353	-1.397876722
3050	HBZ	17.51846857	6.639196818	-1.399796036
140738	TMEM37	61.70189961	23.32931965	-1.403170671
90649	ZNF486	396.8208783	149.794957	-1.405498879
55093	WDYHV1	233.9786167	88.21575242	-1.407268486
81563	C1orf21	2993.889487	1126.36662	-1.410344483
1271	CNTFR	1238.18038	465.8135351	-1.410397038
2065	ERBB3	380.7726423	142.9081728	-1.413841403
1649	DDIT3	423.0747208	157.6920843	-1.423802243
6331	SCN5A	101.7389133	37.80792649	-1.428110955
5864	RAB3A	346.2616256	128.5971831	-1.429003469
2847	MCHR1	11.24716934	4.175016722	-1.429708072
1307	COL16A1	223.5553636	82.67410198	-1.435124785
220001	VWCE	49.88318126	18.44570893	-1.435268237

6515	SLC2A3	7875.522296	2910.418897	-1.436148789
339883	C3orf35	9.039420463	3.339499539	-1.436598366
79412	KREMEN2	3346.229652	1235.826575	-1.437060159
54587	MXRA8	284.5941688	105.0643894	-1.437632339
7101	NR2E1	4872.806952	1798.606236	-1.437873692
27231	NMRK2	24.24290139	8.934497111	-1.440103938
114793	FMNL2	1424.889054	524.5992885	-1.441561835
9060	PAPSS2	735.5721812	270.460196	-1.443450633
287	ANK2	6828.324221	2507.127104	-1.445496423
344558	SH3RF3	15.42110221	5.661900754	-1.445547518
3685	ITGAV	41429.04662	15184.42772	-1.448050084
7694	ZNF135	12.75012455	4.660592813	-1.451925962
154091	SLC2A12	65.66171509	23.99540004	-1.452294568
4661	MYT1	245.2163968	89.58832065	-1.452672881
23550	PSD4	301.2428655	109.9779538	-1.453712724
79012	CAMKV	23.79157361	8.684598035	-1.453919716
79924	ADM2	179.4162956	65.40937926	-1.455741501
1184	CLCN5	1711.733478	623.9271221	-1.456008658
1112	FOXN3	1481.573904	539.2928894	-1.457989675
25914	RTTN	1128.468288	410.4454373	-1.459103521
728215	FAM155A	65.24274713	23.71148746	-1.460231362
643246	MAP1LC3B2	63.54498709	23.07879401	-1.461210481
5230	PGK1	50081.27633	18128.75638	-1.465991369
4664	NAB1	219.3987247	79.39433949	-1.466447082
9622	KLK4	20.17731696	7.285008898	-1.469731708
1815	DRD4	63.54051185	22.80750471	-1.478168097
158326	FREM1	26.82508391	9.61253253	-1.480594203
604	BCL6	137.4439898	49.24598933	-1.480765683
84519	ACRBP	12.33943482	4.390349523	-1.490868611
57121	LPAR5	17.77251179	6.320938912	-1.491436815
8987	STBD1	700.3320897	249.0460748	-1.491626521
4885	NPTX2	168.4877191	59.73783345	-1.495926618
762	CA4	139.2250557	49.34368769	-1.496481426
8537	BCAS1	89.32544993	31.60532013	-1.498903848
3164	NR4A1	1973.818717	694.0458613	-1.507886591
6261	RYR1	286.4669676	100.5428246	-1.510558667
83854	ANGPTL6	74.11517332	25.99955322	-1.511282099
3547	IGSF1	39.6594048	13.88842733	-1.513779782
4601	MXI1	1480.662074	517.8120691	-1.51574192
441234	ZNF716	42.66747897	14.91867602	-1.516017363
347442	DCAF8L2	19.12485031	6.673504638	-1.518931949
645121	CCNI2	70.27143688	24.51650242	-1.519185223
130888	FBXO36	326.0315656	113.6516176	-1.520393431

51168	MYO15A	45.02275112	15.6679766	-1.522835336
22844	FRMPD1	43.91970837	15.27393158	-1.523797009
202915	TMEM184A	308.8040643	107.3788576	-1.523981779
132720	FAM241A	48.47353873	16.80715193	-1.528122136
717	C2	44.78405706	15.5243166	-1.52845547
146439	BICDL2	24.89361917	8.618720318	-1.530230409
730098	LOC730098	114.2569848	39.55369108	-1.530398128
143630	UBQLNL	9.714472348	3.360810647	-1.531326375
115330	GPR146	87.61397811	30.27987357	-1.532801877
7015	TERT	245.00711	84.64944569	-1.53325109
25803	SPDEF	7.958939193	2.748042625	-1.534171772
401720	FIGNL2	1185.707582	408.0183111	-1.539042454
23127	COLGALT2	11303.10609	3881.663702	-1.541972242
130367	SGPP2	116.182745	39.78877178	-1.54596255
100533952	RBAK-RBAKDN	82.90926033	28.38899727	-1.546201356
59353	TMEM35A	3018.591256	1033.171544	-1.546795603
9886	RHOBTB1	178.8542217	60.79047632	-1.556866944
79956	ERMP1	856.5920951	291.0150075	-1.557514809
64283	ARHGEF28	18.72978633	6.351913534	-1.560071263
3781	KCNN2	589.8156386	199.9742353	-1.56044994
282809	POC1B	1367.3772	463.3003544	-1.561391584
257407	C2orf72	249.9773893	84.69557288	-1.561439142
196385	DNAH10	94.76884783	32.08197047	-1.562650143
335	APOA1	67.65514995	22.86331107	-1.565165407
11036	GTF2A1L	18.3496723	6.183949191	-1.569153928
138715	ARID3C	11.0220424	3.710183845	-1.570829001
89	ACTN3	11.06042117	3.722612587	-1.571018935
26355	FAM162A	1892.20235	635.776903	-1.573473866
10507	SEMA4D	677.0696156	227.3105614	-1.574639461
27443	CECR2	2482.990275	833.4482245	-1.574914127
5454	POU3F2	4242.196806	1422.50896	-1.576373813
5172	SLC26A4	45.39793681	15.2161643	-1.577022004
120071	LARGE2	453.3446084	151.8723912	-1.5777485
5024	P2RX3	8.310223308	2.783131141	-1.578178355
6319	SCD	32859.12497	11000.52484	-1.578721705
402778	IFITM10	106.4158358	35.5163106	-1.583159227
881	CCIN	13.31660178	4.442845496	-1.583670096
28999	KLF15	16.09657536	5.366416607	-1.584722816
9456	HOMER1	719.4216698	239.8447648	-1.584736666
402573	C7orf61	6.569815951	2.18637687	-1.587310852
79258	MMEL1	8.307658341	2.763730277	-1.587825061
4255	MGMT	449.8534822	149.4277791	-1.590006816
6550	SLC9A3	1346.777431	447.2987008	-1.590200977

8313	AXIN2	268.4997413	88.98566051	-1.59327592
4638	MYLK	165.6570279	54.85892915	-1.594401044
10000	AKT3	729.4999608	240.82359	-1.598931181
84561	SLC12A8	33.28146358	10.94950528	-1.603853191
222223	KIAA1324L	545.6262595	178.8987221	-1.608769997
165215	FAM171B	6551.341426	2141.236981	-1.613345863
2827	GPR3	42.69611308	13.90454932	-1.618547753
10087	COL4A3BP	2028.081668	660.3849322	-1.618736641
81848	SPRY4	2525.374433	820.2687426	-1.622328751
7634	ZNF80	1005.34633	325.4640354	-1.627122543
4922	NTS	28.88596675	9.346288213	-1.627903348
83881	MIXL1	126.2074813	40.79393967	-1.629370686
115111	SLC26A7	13.69792337	4.419065241	-1.63214406
7380	UPK3A	2.306800265	0.74408038	-1.63236271
5163	PDK1	1954.619389	629.6096507	-1.634358148
55601	DDX60	36.20165523	11.6598841	-1.634502215
57576	KIF17	493.4394574	158.8734127	-1.634995373
9920	KBTBD11	117.9116286	37.94694669	-1.635650294
221301	CALHM4	68.13533723	21.89006651	-1.638126884
90625	ERVH48-1	44.3506836	14.23629898	-1.639382205
145501	ISM2	45.63405395	14.59626389	-1.644511684
5806	PTX3	2.306054253	0.7374646	-1.644780752
7478	WNT8A	2.302848415	0.732703158	-1.65211871
55760	DHX32	243.1256183	77.02613227	-1.658282029
5837	PYGM	2197.163135	696.038425	-1.658403134
83401	ELOVL3	46.25001599	14.64538289	-1.659007952
80303	EFHD1	507.2334324	160.5000749	-1.660075868
11320	MGAT4A	29.52733163	9.31927657	-1.663761115
220082	SPERT	7.933542477	2.502108066	-1.6648211
7570	ZNF22	19.37724214	6.096933827	-1.668207461
414332	LCN10	14.27139424	4.486806434	-1.669365434
23414	ZFPM2	1115.565866	350.3500427	-1.67090672
169026	SLC30A8	2351.642205	737.8760479	-1.672218184
7710	ZNF154	108.2461576	33.95438833	-1.672645866
63973	NEUROG2	7.909472856	2.479232197	-1.67368815
151531	UPP2	12.2702283	3.844076918	-1.674452983
230	ALDOC	620.9540085	194.3957333	-1.675489864
11045	UPK1A	1352.584017	421.466947	-1.682226813
4615	MYD88	101.6558505	31.67159337	-1.682431893
27134	TJP3	1086.811589	337.995265	-1.685026913
728780	ANKDD1B	8.943568786	2.780861191	-1.685318897
388611	CD164L2	8.198732951	2.540456663	-1.690313116
8633	UNC5C	292.7985186	90.70263424	-1.690691898

121256	TMEM132D	3.595302786	1.11131467	-1.6938459
10683	DLL3	3761.894205	1158.027856	-1.699789319
284422	SMIM24	120.9155916	37.20441217	-1.700454657
6340	SCNN1G	2323.985337	715.0538874	-1.700477092
91522	COL23A1	192.6900484	59.27349354	-1.700823068
9508	ADAMTS3	3.609732909	1.10497591	-1.707877176
388553	BHMG1	4.866935695	1.487999932	-1.709639252
7098	TLR3	310.3961602	94.79384668	-1.711245392
149461	CLDN19	10.27491381	3.132854989	-1.713576393
1510	CTSE	10.32331404	3.141040827	-1.716591586
22843	PPM1E	433.2891162	131.6075783	-1.719087432
10673	TNFSF13B	5.945375509	1.798943602	-1.724617976
4045	LSAMP	11.31901816	3.42254409	-1.725607789
5655	KLK10	71.58111292	21.50893851	-1.734642647
2695	GIP	168.8102519	50.6801404	-1.735910096
138255	C9orf135	328.8046669	98.52079969	-1.738730533
2173	FABP7	11.63191166	3.47522059	-1.742911757
80312	TET1	3002.579331	897.0378781	-1.742961553
10256	CNKSR1	11.8342742	3.534515175	-1.743386987
56099	PCDHGB7	215.3271806	64.26400202	-1.74444771
440836	ODF3B	330.3692719	98.56300396	-1.744961374
5137	PDE1C	23.78346319	7.095490139	-1.744984555
84898	PLXDC2	621.4802126	185.3948094	-1.745107606
114786	XKR4	5.963568568	1.774599728	-1.748682238
1010	CDH12	54.71681522	16.25666417	-1.75095301
55843	ARHGAP15	2955.719926	877.8180119	-1.751515793
728340	GTF2H2C	461.4050212	137.0265325	-1.751578434
154141	MBOAT1	1705.89853	506.3829338	-1.752231147
80125	CCDC33	2.60454584	0.772793442	-1.752877071
3587	IL10RA	19.96844036	5.914680534	-1.755349503
8277	TKTL1	6.245574597	1.844770645	-1.759392846
401562	LCNL1	2.602617636	0.767965343	-1.760850262
50515	CHST11	1671.606944	492.9152544	-1.761824123
55603	FAM46A	39.57688433	11.64927848	-1.76441744
8693	GALNT4	1652.903864	485.8801864	-1.76633031
84034	EMILIN2	2573.600264	755.3924182	-1.76848978
487	ATP2A1	102.3676486	30.02017556	-1.769755531
27019	DNAI1	447.4385195	130.7636636	-1.77472776
51778	MYOZ2	362.3776644	105.8279579	-1.775773223
117247	SLC16A10	9.61168777	2.80598183	-1.776284117
84628	NTNG2	93.89180744	27.40956182	-1.776320016
1848	DUSP6	6899.379793	2011.308673	-1.778332172
1268	CNR1	17.65715473	5.134344007	-1.78200102

1730	DIAPH2	6.247818763	1.815473112	-1.783007041
221914	GPC2	104.9224708	30.40765851	-1.786817053
25854	FAM149A	737.289052	213.6222781	-1.787168225
7087	ICAM5	1978.33004	573.118167	-1.787378594
126147	NTN5	5.236395038	1.516580667	-1.787751702
49	ACR	15.63557278	4.517804527	-1.791138315
9844	ELMO1	169.106665	48.78016285	-1.793567042
26051	PPP1R16B	87.41332996	25.13667691	-1.798059361
84000	TMPRSS13	5.227428524	1.502074437	-1.799145121
3109	HLA-DMB	101.3432028	29.06131546	-1.802077416
653220	XAGE1A	6.280743597	1.79921241	-1.803569858
653067	XAGE1B	6.280743597	1.79921241	-1.803569858
7137	TNNI3	31.84458795	9.105990937	-1.806160276
5179	PENK	2.870488238	0.819797077	-1.807957393
9473	THEMIS2	321.0972458	91.34225597	-1.813655963
83878	USHBP1	44.83364816	12.74165182	-1.815029578
7020	TFAP2A	209.0919877	59.23083935	-1.819717344
8654	PDE5A	144.9080962	41.02409467	-1.820594801
339834	CCDC36	6.571147677	1.85989838	-1.820921567
779	CACNA1S	5.251999422	1.477074442	-1.83012422
169166	SNX31	123.4719031	34.65870113	-1.832893289
255022	CALHM1	2.894549473	0.809248448	-1.838684217
126969	SLC44A3	78.88176378	21.94412416	-1.845857121
375033	PEAR1	22.08796035	6.140137097	-1.84691743
143872	ARHGAP42	104.8512883	29.13630136	-1.847454933
1645	AKR1C1	6.852968503	1.901408282	-1.849660707
25837	RAB26	5.524301783	1.528808765	-1.853384179
79094	CHAC1	249.700429	69.09922791	-1.853456804
8482	SEMA7A	60.11253826	16.60109408	-1.856387615
1295	COL8A1	650.6359062	179.5812895	-1.857213397
339390	CLEC4G	2.903653093	0.800750317	-1.858444733
29969	MDFIC	3481.167815	957.8919538	-1.861636523
1832	DSP	1075.273721	295.2957957	-1.864471236
586	BCAT1	3887.992777	1060.458155	-1.874337844
206338	LVRN	32.15340277	8.749596107	-1.877683098
5333	PLCD1	1776.176852	482.8184364	-1.879222565
83643	CCDC3	63.86970056	17.26384103	-1.887378202
54106	TLR9	46.55793118	12.54094193	-1.892381243
57188	ADAMTSL3	105.8144123	28.48397969	-1.893313506
5015	OTX2	4.536017653	1.220594345	-1.893842444
4585	MUC4	21.63776045	5.81942694	-1.894602187
388581	C1QTNF12	76.88228931	20.65943686	-1.895850366
116844	LRG1	198.0693411	53.07758346	-1.899830991

4131	MAP1B	2985.544731	799.7618158	-1.900351877
171024	SYNPO2	2338.276282	624.4229313	-1.904849979
149483	CCDC17	46.91520186	12.51348196	-1.906572188
664	BNIP3	19832.44194	5289.517692	-1.90665424
348738	C2orf48	18.00385738	4.793424895	-1.909177308
5797	PTPRM	372.1312987	99.04214632	-1.909697251
54551	MAGEL2	56.479312	15.02147829	-1.910695716
1272	CNTN1	24.35267816	6.474805413	-1.911171699
10630	PDPN	6.925519718	1.832466384	-1.918135608
10253	SPRY2	2705.028505	712.4658595	-1.924751008
79152	FA2H	2623.019809	690.5884087	-1.925330679
135932	TMEM139	22.0539519	5.803721685	-1.925986956
10144	FAM13A	14.87570398	3.897926321	-1.932179219
7903	ST8SIA4	86.04816042	22.54234867	-1.932506513
5125	PCSK5	11.15382605	2.92091131	-1.933048219
54768	HYDIN	5.859516155	1.532077801	-1.935291979
151835	CPNE9	15.98682496	4.16486494	-1.940541825
2902	GRIN1	80.61639482	20.99301985	-1.941163553
286046	XKR6	3.217849897	0.836308503	-1.943989894
154790	CLEC2L	50.33010371	13.05863328	-1.946417658
91351	DDX60L	148.2803091	38.29388741	-1.953141
7291	TWIST1	141.0095178	36.32932435	-1.956586105
9940	DLEC1	20.00233066	5.152563515	-1.956805823
3426	CFI	16.29497849	4.161990633	-1.969081824
8447	DOC2B	99.70015962	25.26401813	-1.980511703
9628	RGS6	4.843461264	1.227307744	-1.980541358
3046	HBE1	3.527514558	0.892938881	-1.982018701
56171	DNAH7	51.64275313	13.06416196	-1.982951333
79789	CLMN	65.08060095	16.33525775	-1.994238354
81031	SLC2A10	1008.562043	252.5621181	-1.997589666
2047	EPHB1	150.903013	37.67169412	-2.002068793
55203	LGI2	172.6499119	42.89656391	-2.008915603
6098	ROS1	62.62368187	15.51181426	-2.013340898
126393	HSPB6	54.06782375	13.39214497	-2.013383238
84101	USP44	1533.056384	379.100893	-2.015756998
338645	LUZP2	1507.782924	372.0839552	-2.018728652
84891	ZSCAN10	10.25643226	2.527385112	-2.020811553
629	CFB	6.513957193	1.596566537	-2.02856156
4897	NRCAM	64.1313644	15.70969909	-2.029374554
4237	MFAP2	582.6202637	142.3754619	-2.032855357
148229	ATP8B3	149.7161097	36.55701402	-2.034009325
340120	ANKRD34B	11.91381362	2.8954876	-2.040757071
80709	AKNA	760.0096756	184.4397304	-2.042868323

10321	CRISP3	3.522195126	0.852271097	-2.047090522
8711	TNK1	31.07917127	7.501485586	-2.050699796
6445	SGCG	129.2412026	31.15602507	-2.052482992
26166	RGS22	3.784281829	0.910492218	-2.055300944
27345	KCNMB4	261.9824976	62.99277414	-2.056212179
56479	KCNQ5	43.56276693	10.47418579	-2.05625749
3352	HTR1D	148.765819	35.67545886	-2.060039195
2045	EPHA7	28.94536249	6.911707405	-2.066218174
93986	FOXP2	2017.528403	479.4409	-2.073164091
1910	EDNRB	93.28069618	22.05424587	-2.080522126
6328	SCN3A	51.54392154	12.11392042	-2.089136465
8224	SYN3	5.175828297	1.21282658	-2.093416482
56944	OLFML3	20.93821529	4.872232706	-2.103483531
431707	LHX8	20.98860223	4.871132749	-2.107276886
81578	COL21A1	66.42973031	15.389472	-2.109885323
84889	SLC7A3	3087.246001	715.2044615	-2.109892804
30817	ADGRE2	28.89534819	6.63581591	-2.122491486
3060	HCRT	21.33688694	4.883421363	-2.127385531
2122	MECOM	89.75671823	20.53972666	-2.127602945
341405	ANKRD33	11.47638349	2.623500351	-2.129103195
79674	VEPH1	8.49615267	1.911577173	-2.152046246
26052	DNM3	43.84964586	9.8316597	-2.157058305
5443	POMC	31.09856409	6.953529999	-2.161030507
4828	NMB	10.1642197	2.253359907	-2.1733498
8174	MADCAM1	137.979781	30.53249539	-2.176039466
57502	NLGN4X	5.806226837	1.275663124	-2.186353542
27123	DKK2	44.93709076	9.865089365	-2.187502702
152831	KLB	113.7455535	24.93252819	-2.189709063
54549	SDK2	541.0910822	116.7369697	-2.21260994
10409	BASP1	316.1328598	67.87418084	-2.219596215
4038	LRP4	3579.893916	766.6922794	-2.223197279
126014	OSCAR	378.6610152	80.66025722	-2.230976986
11174	ADAMTS6	99.48468931	20.96367493	-2.246582869
4692	NDN	184.1485155	38.7371481	-2.249080118
152273	FGD5	92.05715228	19.34372397	-2.250664252
7145	TNS1	10.85855084	2.278082629	-2.252939595
64388	GREM2	12.49734735	2.606396118	-2.261493641
2348	FOLR1	95.61181296	19.75164346	-2.275216176
7148	TNXB	206.7048416	42.03331529	-2.297967026
79875	THSD4	16.23974118	3.28426432	-2.305886494
6480	ST6GAL1	1215.930796	244.8645627	-2.312005216
285141	ERICH2	57.59308012	11.49473479	-2.3249223
1004	CDH6	44157.32264	8790.754221	-2.328593846

22865	SLITRK3	215.9056415	42.69861258	-2.338139843
51050	PI15	1032.123474	203.1763497	-2.344811189
64218	SEMA4A	15.20022431	2.988209048	-2.346739629
400935	IL17REL	23.55051881	4.627015371	-2.347605046
148198	ZNF98	13.46000135	2.643471943	-2.348172635
83592	AKR1E2	48.93086075	9.532768516	-2.359777493
5652	PRSS8	44.48190916	8.634964727	-2.36495652
2	A2M	43.08231682	8.294969889	-2.376787186
5325	PLAGL1	555.2779852	106.7199755	-2.379379959
5087	PBX1	43.35241623	8.331488533	-2.379466223
284111	SLC13A5	6.784366519	1.301406685	-2.382142243
22837	COBLL1	990.2591736	189.7983827	-2.383338462
117166	WFIKKN1	374.312692	71.03443028	-2.397653596
2898	GRIK2	20.87665538	3.95771699	-2.399150242
494470	RNF165	113.2355443	21.39377958	-2.4040636
6866	TAC3	36.72266015	6.937728719	-2.404135236
23327	NEDD4L	80.22929545	15.114073	-2.408236631
131540	ZDHHC19	139.6159385	25.97183239	-2.426443939
2028	ENPEP	53.26431983	9.899547376	-2.427734969
254122	SNX32	5.137441405	0.948990812	-2.43658401
164832	LONRF2	23.49889613	4.329946769	-2.440171793
710	SERPING1	5866.325976	1077.977194	-2.444130584
168620	BHLHA15	27.62730018	4.996958499	-2.466972443
10251	SPRY3	1695.258663	305.7358519	-2.471147773
5210	PFKFB4	1566.211379	282.3172584	-2.471889704
1593	CYP27A1	29.34236357	5.288078713	-2.47216953
58494	JAM2	146.0489716	26.30502041	-2.473042125
7075	TIE1	35.07569699	6.277463364	-2.482218163
1404	HAPLN1	126.2225725	22.44098533	-2.491762004
136288	C7orf57	3774.30224	662.022825	-2.511257093
64123	ADGRL4	30.29140795	5.296019855	-2.5159282
283710	LOC283710	13.49059468	2.351374708	-2.520377579
146429	SLC22A31	136.5815561	23.73199009	-2.524857685
4857	NOVA1	254.1389952	44.00600932	-2.529845308
51557	LGSN	144.2870333	24.97291916	-2.530505278
10826	FAXDC2	935.1492692	160.4912351	-2.542702158
10194	TSHZ1	234.8235229	39.75221201	-2.562469888
256691	MAMDC2	1948.958946	328.3615365	-2.569346653
126326	GIPC3	32.03842184	5.352378673	-2.581550991
2158	F9	6.098937541	1.015885249	-2.585820492
3912	LAMB1	1044.282211	171.7587918	-2.604055791
9427	ECEL1	399.5560295	64.74574617	-2.625540511
130399	ACVR1C	14.50131174	2.346176427	-2.627799997

54514	DDX4	18.81828301	3.037687947	-2.631089423
254173	TTLL10	6.101726844	0.981226825	-2.636559016
5649	RELN	10.73907417	1.726857856	-2.636648383
57554	LRRC7	6.396874747	1.026050166	-2.640265965
3174	HNF4G	27.21886398	4.357895277	-2.642903422
391059	FRRS1	100.3792962	15.96673394	-2.652320597
402665	IGLON5	89.93735611	14.24204183	-2.658764479
23022	PALLD	786.3871742	123.504879	-2.670671756
1946	EFNA5	76.36776293	11.9228402	-2.679235814
1482	NKX2-5	60.62316809	9.269016206	-2.70938112
84634	KISS1R	747.325097	113.3758522	-2.720622578
116984	ARAP2	11.3926327	1.708589654	-2.737223319
79727	LIN28A	202.3385626	30.12926824	-2.747533762
167410	LIX1	1023.660518	150.4812796	-2.76608142
80059	LRRTM4	36.71847083	5.351293248	-2.778546483
9315	NREP	2540.886445	366.3526002	-2.794027141
255488	RNF144B	74.08827157	10.64599367	-2.798934564
460	ASTN1	138.4689927	19.83185941	-2.803671096
10351	ABCA8	35.56931591	5.072972983	-2.809729845
8076	MFAP5	1032.183224	146.54271	-2.816305982
50863	NTM	728.5151939	103.2258088	-2.819155338
3270	HRC	483.9779852	67.55431594	-2.840821576
23349	PHF24	22.73365813	3.102119201	-2.873503822
22925	PLA2R1	13.02125739	1.759456351	-2.887667139
4345	CD200	7.713714524	1.041368738	-2.888944752
144568	A2ML1	162.4134686	21.89030602	-2.891307249
92162	TMEM88	3023.580385	405.6608794	-2.897911849
3816	KLK1	52.56425415	7.049947457	-2.898397631
2566	GABRG2	503.9280383	67.43452105	-2.9016585
56979	PRDM9	849.8783943	113.1027808	-2.909622027
79729	SH3D21	292.9070471	38.93168109	-2.911426356
51305	KCNK9	61.20471773	7.943751347	-2.945750492
79632	FAM184A	85.51888612	11.01529262	-2.956735241
153579	BTNL9	39.04447217	5.023756487	-2.958279869
100271846	ERVV-2	92.9982875	11.95946635	-2.959051135
5156	PDGFRA	1829.560616	235.2470182	-2.959248873
63982	ANO3	29.5428112	3.759021273	-2.97437813
799	CALCR	1790.383051	227.2299797	-2.978043189
9068	ANGPTL1	8.671218989	1.093898331	-2.986756162
10468	FST	2938.991602	362.4059243	-3.019642792
441521	CT45A5	16.96185239	2.077265675	-3.029536085
9510	ADAMTS1	17.08924934	2.08273748	-3.036536117
91851	CHRDL1	314.1023062	36.93117377	-3.088323514

131149	OTOL1	76.73092509	9.010444065	-3.090138035
7078	TIMP3	440.6661364	51.71757496	-3.090959501
339453	TMEM240	23.53376319	2.755201045	-3.094502534
339479	BRINP3	346.3305852	40.27612716	-3.104152931
51309	ARMCX1	80.46391106	9.335663941	-3.10751733
1462	VCAN	20.81856416	2.406340966	-3.112957586
794	CALB2	38.60329382	4.426670528	-3.124430046
11075	STMN2	2083.444088	238.6937074	-3.125737945
57172	CAMK1G	18.79883968	2.15233661	-3.126667989
81704	DOCK8	21.75066363	2.462619268	-3.142793916
6857	SYT1	2153.495422	242.347306	-3.151532306
388531	RGS9BP	103.6913542	11.62951604	-3.156432641
6549	SLC9A2	15.76965058	1.755353129	-3.167317498
4118	MAL	232.1777328	25.76101172	-3.171968457
6326	SCN2A	73.29640953	8.030204273	-3.190233936
56884	FSTL5	95.12898174	10.37078625	-3.19735966
642475	MROH6	154.5849272	16.57980104	-3.220901057
1296	COL8A2	110.7931789	11.64993281	-3.249475523
9743	ARHGAP32	589.4445325	61.96433979	-3.249845965
83690	CRISPLD1	3668.569594	379.8809776	-3.271598277
3948	LDHC	14.34648849	1.464890513	-3.291832914
100131390	SP9	24.19840269	2.459007186	-3.298763964
79827	CLMP	77.31804335	7.757812453	-3.317083325
10840	ALDH1L1	1454.782332	143.492616	-3.341754906
8433	UTF1	35.8104431	3.491040133	-3.358651523
7425	VGF	3147.477425	306.563507	-3.359938155
3485	IGFBP2	1076.762284	103.1270891	-3.384204533
2888	GRB14	199.7667008	18.89667773	-3.402111601
1299	COL9A3	339.335324	30.58935787	-3.471609892
124602	KIF19	16.68394756	1.493826163	-3.481376506
1285	COL4A3	16.75201325	1.481230958	-3.499465976
170825	GSX2	415.5055285	36.16658174	-3.522138519
10417	SPON2	41.02592434	3.491763857	-3.554507939
148113	CILP2	248.7019314	20.89701374	-3.573049016
83482	SCRT1	271.3654482	20.92913434	-3.696652494
345557	PLCXD3	67.25083467	5.157375959	-3.70484305
79772	MCTP1	794.8710114	59.22094558	-3.746541333
7001	PRDX2	212.7829559	15.72738177	-3.758032173
646457	C19orf67	20.40048518	1.505774724	-3.760025611
7180	CRISP2	16.39307612	1.181434131	-3.794475497
6005	RHAG	21.38948216	1.533568099	-3.801937419
22871	NLGN1	92.85345153	6.465896588	-3.8440332
25928	SOSTDC1	255.0638705	17.68597526	-3.850180877

285220	EPHA6	22.03628992	1.524677179	-3.853305631
22797	TFEC	36.83115251	2.541488118	-3.857181151
8128	ST8SIA2	17.98184687	1.209022019	-3.894628779
4772	NFATC1	131.9762133	8.795723774	-3.907331822
114907	FBXO32	98.52112183	6.539057469	-3.913278448
7103	TSPAN8	43.30696866	2.867305986	-3.916831416
22999	RIMS1	385.00555	24.58226685	-3.969189378
30010	NXPH1	63.09527881	3.864651549	-4.029121811
760	CA2	157.8958783	9.46440068	-4.060318549
11030	RBPMS	96.18329304	5.529055513	-4.120681359
151254	C2CD6	44.99723229	2.567002724	-4.131679534
100288801	FRG2C	286.0858944	16.17485542	-4.144623644
448831	FRG2	101.1907678	5.540430556	-4.190935766
8784	TNFRSF18	49.40239853	2.623104056	-4.235234143
28954	REM1	24.39158217	1.238501368	-4.299715975
55103	RALGPS2	236.6834116	11.79173891	-4.327110204
4593	MUSK	26.04492028	1.25114203	-4.379684542
441581	FRG2B	67.86321321	3.250059092	-4.384091886
2904	GRIN2B	26.38766594	1.252000549	-4.397556646
56776	FMN2	155.0220723	7.221953937	-4.423940615
203328	SUSD3	49.68014098	2.264687883	-4.45528513
285313	IGSF10	36.88517583	1.630155577	-4.49995955
1286	COL4A4	54.05288841	2.293392806	-4.558816331
282890	ZNF311	46.97490418	1.947518439	-4.592181326
1902	LPAR1	113.9234965	4.596572557	-4.631363006
64849	SLC13A3	418.2755418	16.8412393	-4.634383429
1535	CYBA	318.2772339	12.22155319	-4.702784418
143662	MUC15	367.5309157	13.21477943	-4.79764136
6335	SCN9A	284.4864123	9.895068714	-4.845506212
100529239	RPS10-NUDT3	86.14388262	2.773687207	-4.956871342
80034	CSRNP3	770.2734183	22.88895289	-5.07264727
221002	RASGEF1A	422.8177547	12.55583557	-5.073606013
29951	PDZRN4	247.1067504	6.650616455	-5.21550254
9388	LIPG	166.3640431	4.366551819	-5.25170538
9074	CLDN6	1031.06785	23.16980603	-5.475749497
8492	PRSS12	195.4130034	3.757619783	-5.700563567
26002	MOXD1	845.9801119	15.9769953	-5.726555728
6332	SCN7A	188.1574123	3.458194638	-5.765777243
118427	OLFM3	135.1779913	2.457237233	-5.781679322
10752	CHL1	837.0839112	14.11589861	-5.889979372
429	ASCL1	197.4930523	2.233299275	-6.466481497

Table S1d

Gene ID	Gene symbol	ctrl Expression	E400K Expression	log2FoldChange (E400K/ctrl)
100137049	PLA2G4B	3.626972711	86.29739471	4.57247921
5592	PRKG1	11.26207259	117.0623127	3.377732426
1103	CHAT	5.783512376	58.17817717	3.33046027
6572	SLC18A3	24.58705691	238.5871722	3.278545519
57554	LRRC7	13.93565321	105.4218556	2.919321458
401335	C7orf65	7.187856282	49.17842364	2.774392024
431707	LHX8	15.5023933	94.92633864	2.614317479
130399	ACVR1C	16.39897286	80.84962824	2.301635685
2909	ARHGAP35	3197.769969	15219.44337	2.250777527
25893	TRIM58	32.77957419	142.0823671	2.115858503
51441	YTHDF2	3730.939366	13332.15565	1.837299246
91227	GGTLC2	1.991731651	6.618733144	1.732531821
56142	PCDHA6	11.08917335	34.73437472	1.647212303
9235	IL32	13.74418181	42.99169399	1.645236931
391195	OR2T33	2.069251621	6.209159672	1.585288944
26052	DNM3	48.18087548	139.7723409	1.536546385
100505767	SPDYE18	1.884118728	5.391184644	1.516712442
150221	RIMBP3C	13.19807288	37.25072467	1.496941203
343172	OR2T8	4.860808574	13.07298444	1.427320308
56171	DNAH7	45.57978059	122.379323	1.424893938
6549	SLC9A2	14.41061123	37.76411924	1.389884611
131149	OTOL1	63.14200587	163.364322	1.371420943
147664	ERVV-1	6.749500602	17.1595742	1.346161088
84419	C15orf48	7.428193084	18.45684433	1.313072687
55824	PAG1	1201.812534	2943.323261	1.292234126
51704	GPRC5B	788.4793279	1902.895661	1.271051622
55753	OGDHL	745.4940132	1754.599307	1.234872932
22865	SLITRK3	258.8124248	583.9883216	1.17403264
4916	NTRK3	28.32932153	63.66641238	1.16823642
56143	PCDHA5	20.37144582	44.76670802	1.135877855
6588	SLN	18.70694634	41.05809967	1.134092779
101059918	GOLGA8R	51.77708699	112.5894206	1.120685566
283008	NUTM2E	62.46525164	130.5625561	1.063615436
1373	CPS1	9264.086921	18816.378	1.022268253
8543	LMO4	368.808152	183.9699452	-1.00340045
170689	ADAMTS15	225.9082829	112.588351	-1.004679601
5443	POMC	64.90299701	32.1238979	-1.014638139
345557	PLCXD3	105.0603517	51.87234424	-1.018180844
288	ANK3	35.981245	17.71727146	-1.022088668
286499	FAM133A	197.1216455	96.97841775	-1.023350584

9900	SV2A	117.2779418	57.31550908	-1.032934212
26136	TES	456.7834735	222.8647649	-1.035341911
4753	NELL2	8891.079966	4328.762695	-1.038403955
8447	DOC2B	111.0986546	53.8298989	-1.045361725
79012	CAMKV	29.64381796	14.35971131	-1.045704525
153572	IRX2	40.89132287	19.79772985	-1.046459726
8614	STC2	26.28151358	12.71864833	-1.047103007
346689	KLRG2	132.4595175	63.82999039	-1.053245173
2515	ADAM2	80.96785122	38.94425497	-1.055938672
642475	MROH6	131.7386442	63.21265156	-1.05939337
57512	GPR158	804.1776315	385.2480755	-1.061726458
1824	DSC2	2667.371499	1274.22581	-1.065797806
27129	HSPB7	100.3640449	47.94170444	-1.065889414
57493	HEG1	438.3718065	208.742887	-1.070427977
5332	PLCB4	159.5559291	75.27119744	-1.083892393
57514	ARHGAP31	208.9989108	98.24640012	-1.089018973
64135	IFIH1	33.30521398	15.65312785	-1.089297081
5307	PITX1	190.7181368	89.53316628	-1.090947935
89846	FGD3	393.3565347	184.4485953	-1.092618749
5325	PLAGL1	823.5378621	385.2993596	-1.095855191
163782	KANK4	680.213741	317.7322725	-1.098176516
25945	NECTIN3	46.59275625	21.71051893	-1.101711468
9180	OSMR	59.86604835	27.81557257	-1.105845241
64478	CSMD1	223.9314591	103.9913196	-1.106594112
1602	DACH1	384.4361414	177.1508878	-1.117765277
728747	ANKRD20A4	28.51881447	13.13854497	-1.118108497
579	NKX3-2	104.7819886	48.1850052	-1.120734581
3198	HOXA1	54.4318287	24.92292462	-1.126977132
23452	ANGPTL2	60.56873154	27.72512119	-1.127379432
26999	CYFIP2	93.03323361	42.56524846	-1.128070121
347442	DCAF8L2	21.73376487	9.925884981	-1.130670468
23624	CBLC	71.21527629	32.42200777	-1.135213311
3973	LHCGR	74.21019894	33.74521245	-1.136934638
6768	ST14	37.84396332	17.18798638	-1.138662648
9856	KIAA0319	28.21485698	12.80197147	-1.140089038
771	CA12	320.9480936	145.5967134	-1.140362202
85450	ITPRIP	197.2634295	89.39399711	-1.141873659
8436	CAVIN2	85.87178663	38.7292319	-1.148761319
2330	FMO5	153.3823754	68.9389816	-1.153740826
6092	ROBO2	313.498841	140.2552722	-1.160405107
6691	SPINK2	63.79300981	28.52162435	-1.1613422
130367	SGPP2	144.2935561	64.4034649	-1.16379666
117245	HRASLS5	381.5591781	168.9290116	-1.175489714

130576	LYPD6B	27.10739536	11.92457427	-1.184748738
4313	MMP2	87.535408	38.46025786	-1.186498269
26064	RAI14	175.4995795	77.08219739	-1.186997969
2188	FANCF	24.07339005	10.57268283	-1.187097809
4921	DDR2	265.6228007	116.5639076	-1.188257843
3673	ITGA2	22.7269117	9.927197938	-1.19494319
84072	HORMAD1	26.48870961	11.5288142	-1.200133433
9435	CHST2	29.07826042	12.58210525	-1.208567628
23671	TMEFF2	38.04735093	16.31933461	-1.221213773
27010	TPK1	19.57548507	8.379802121	-1.224059975
79822	ARHGAP28	96.4548472	41.25885482	-1.225149962
4608	MYBPH	15.43821751	6.556000099	-1.235618408
55170	PRMT6	33.70566649	14.31065159	-1.235901791
286319	TUSC1	120.4570245	51.07291379	-1.237888253
3199	HOXA2	38.38700456	16.27231728	-1.238198273
10215	OLIG2	3.750105783	1.584410239	-1.242985362
3753	KCNE1	48.52309092	20.50064071	-1.243002454
258010	SVIP	30.9881703	13.0458031	-1.248131813
29944	PNMA3	75.77307166	31.87626163	-1.249202789
390	RND3	25.58113718	10.73979565	-1.252113855
2150	F2RL1	486.422742	204.2081163	-1.252170474
5342	PLGLB2	4.940441437	2.070862741	-1.254408022
124925	SEZ6	25.61529393	10.73268605	-1.254994264
56979	PRDM9	655.470528	274.4001732	-1.256249522
57216	VANGL2	285.3720411	118.6697235	-1.265892092
83690	CRISPLD1	4860.434211	2009.982045	-1.27390259
167410	LIX1	1199.122647	495.5227444	-1.274956042
1272	CNTN1	14.87335531	6.14553413	-1.275119834
200162	SPAG17	6.12609813	2.522835379	-1.279922407
9749	PHACTR2	450.6287052	185.1197582	-1.283480331
26166	RGS22	14.89603084	6.111042526	-1.285437539
6004	RGS16	9.007722404	3.682162313	-1.290609144
127833	SYT2	101.3031682	41.34436818	-1.292916567
653220	XAGE1A	13.0374053	5.31711408	-1.293941448
653067	XAGE1B	13.0374053	5.31711408	-1.293941448
3075	CFH	5.700716832	2.318972628	-1.29765755
54437	SEMA5B	8.318473414	3.378481038	-1.299944035
81578	COL21A1	79.69829541	32.32027766	-1.302109276
2898	GRIK2	19.711748	7.978884486	-1.304796752
340578	DCAF12L2	11.18756413	4.507405335	-1.311526855
8492	PRSS12	151.6720693	61.00381143	-1.313984147
100528021	ST20-MTHFS	8.822525715	3.54451468	-1.315603625
5787	PTPRB	71.63452742	28.70077876	-1.319565242

2028	ENPEP	55.01123969	22.00991445	-1.321572877
79802	HHIPL2	28.88432901	11.51016994	-1.327377847
5156	PDGFRA	3098.916087	1233.339484	-1.329193725
647042	GOLGA6L10	21.9783997	8.704272668	-1.336290688
84159	ARID5B	364.6643972	144.2604964	-1.33789306
55604	CARMIL1	40.64568546	16.0395229	-1.34147099
1829	DSG2	1499.442914	588.4557153	-1.349420844
4897	NRCAM	87.67745442	34.38818654	-1.350292875
9074	CLDN6	604.204931	236.3508074	-1.354108165
5168	ENPP2	6.234021041	2.436725296	-1.355219406
1040	CDS1	14.63780058	5.707885023	-1.358670617
2977	GUCY1A2	7.20324717	2.808507001	-1.35884401
10017	BCL2L10	6.257969336	2.422244186	-1.369350279
1902	LPAR1	101.8324195	38.88642596	-1.388858385
63895	PIEZO2	4.586933875	1.750551698	-1.389720442
153579	BTNL9	23.44386306	8.943910712	-1.390232625
11166	SOX21	335.4875365	127.8795159	-1.391473983
5396	PRRX1	27.55649582	10.47078785	-1.396022443
23327	NEDD4L	90.1894231	34.20219136	-1.39886949
4008	LMO7	23.45705864	8.895415712	-1.398888186
84333	PCGF5	465.2872912	176.2779258	-1.400269956
429	ASCL1	87.48706489	32.88714173	-1.411546101
55760	DHX32	308.0919202	115.1866177	-1.419387733
1307	COL16A1	180.0850261	67.31076693	-1.419769028
152273	FGD5	71.07079445	26.55955404	-1.420025904
613037	NPIPB13	85.67855788	31.96647187	-1.422374674
10893	MMP24	165.8563221	61.49144515	-1.431476388
56479	KCNQ5	77.32621893	28.4290159	-1.443593511
9844	ELMO1	176.268394	64.44703063	-1.45158802
7045	TGFBI	23.93485582	8.75089706	-1.451610294
57502	NLGN4X	10.50915869	3.839486044	-1.45266207
83857	TMTC1	190.3698829	69.31749814	-1.457513768
203447	NRK	13.46033682	4.880471051	-1.463622206
50863	NTM	598.7928691	216.4119702	-1.468276741
22797	TFEC	20.09019697	7.259889332	-1.468472247
6328	SCN3A	61.06405211	21.97422836	-1.474510823
29953	TRHDE	4.917954121	1.766246606	-1.477371488
3485	IGFBP2	1151.949959	412.4799646	-1.481682097
7180	CRISP2	6.151115909	2.18408033	-1.493822243
157638	FAM84B	197.4717008	69.88642795	-1.498561704
23150	FRMD4B	236.3322567	83.56004034	-1.49993146
121256	TMEM132D	5.450929253	1.919951679	-1.505432194
23237	ARC	42.07316357	14.62193522	-1.524766036

6546	SLC8A1	146.2314491	50.46742896	-1.53482912
367	AR	267.3653487	92.20683785	-1.535866854
1482	NKX2-5	52.36522987	18.01486229	-1.539421567
1404	HAPLN1	116.470313	39.98710876	-1.542355398
1803	DPP4	24.44483485	8.268713623	-1.563794849
79987	SVEP1	5.73271525	1.9314644	-1.569523534
59	ACTA2	28.71586805	9.631312361	-1.576043876
134429	STARD4	12.0195063	4.024014505	-1.578670228
51334	PRR16	638.0211886	213.5828364	-1.57880862
29951	PDZRN4	238.5711862	79.52535696	-1.584932962
112399	EGLN3	6.766382503	2.24061583	-1.594489425
4094	MAF	53.3757978	17.67214062	-1.594708925
4504	MT3	8.033152156	2.651292059	-1.5992706
80309	SPHKAP	5.778177373	1.901557722	-1.603432758
27123	DKK2	82.10424523	26.96007094	-1.606632526
1000	CDH2	929.1663691	303.459041	-1.614435133
2043	EPHA4	8.311840247	2.682989669	-1.631326427
5583	PRKCH	16.88314479	5.442003111	-1.633373971
79875	THSD4	24.29579335	7.772406339	-1.644273311
23657	SLC7A11	201.1857669	64.24440947	-1.646885422
64220	STRA6	158.7671514	50.31528803	-1.657843726
27063	ANKRD1	49.93083454	15.77834271	-1.661985342
56776	FMN2	132.316543	41.80242436	-1.662334927
341640	FREM2	37.63849466	11.71888972	-1.683373038
57188	ADAMTSL3	94.94353831	29.38986334	-1.691751166
2861	GPR37	6.653379538	2.053662657	-1.695888116
1730	DIAPH2	15.60763448	4.74252516	-1.718524564
5789	PTPRD	71.90222471	21.80511573	-1.721369762
5651	TMPRSS15	17.20789652	5.164790176	-1.73628911
91351	DDX60L	203.8856882	61.10411003	-1.73841918
4311	MME	21.81040766	6.52925077	-1.740027379
11170	FAM107A	18.25976908	5.43103862	-1.749368493
55796	MBNL3	219.9244133	65.22427875	-1.753526773
170692	ADAMTS18	672.3075315	199.349643	-1.753820289
3912	LAMB1	2053.857571	608.3398068	-1.755386823
114907	FBXO32	153.3189228	45.08021178	-1.765969569
54551	MAGEL2	49.28535023	14.45951547	-1.769139669
51473	DCDC2	45.47681506	13.2090381	-1.783605809
28316	CDH20	6.986290332	2.028928691	-1.783808438
9388	LIPG	231.2079809	66.99275455	-1.78711422
7020	TFAP2A	500.7868863	145.0164558	-1.787980164
55601	DDX60	51.51782057	14.83742184	-1.795831132
2706	GJB2	21.00897575	6.03647789	-1.799226898

6332	SCN7A	170.5903253	48.83730524	-1.804480327
190	NR0B1	24.67474519	7.046821404	-1.807990631
3872	KRT17	8.569005096	2.442798823	-1.810592655
25834	MGAT4C	11.94641473	3.404304012	-1.811145928
23057	NMNAT2	24.75162054	6.939490876	-1.834621258
116984	ARAP2	16.19432649	4.516179918	-1.842313602
26011	TENM4	13.85539848	3.819271249	-1.859078913
79953	SYNDIG1	8.898402118	2.447948181	-1.861973276
3730	ANOS1	108.0309117	29.45464517	-1.874877102
1946	EFNA5	67.23176534	18.15180205	-1.88903025
22837	COBLL1	939.3732652	251.1092979	-1.903383086
442213	PTCHD4	8.421514287	2.248195091	-1.905312437
10085	EDIL3	18.46310295	4.923365971	-1.906928146
6335	SCN9A	253.5467691	67.60591284	-1.907030555
23022	PALLD	1027.571729	270.1397219	-1.927461406
51481	VCX3A	10.10088191	2.640344033	-1.935683432
344758	GPR149	15.13524963	3.883438982	-1.962505767
118427	OLFM3	143.4464169	36.68705706	-1.967168847
24141	LAMP5	176.4207182	44.65100303	-1.982255506
9510	ADAMTS1	20.59595127	5.073603509	-2.021278076
2122	MECOM	162.5963426	38.093539	-2.093676576
169166	SNX31	104.6284868	24.10753525	-2.11771964
10202	DHRS2	129.1110166	29.73828421	-2.118218789
5456	POU3F4	11.69683476	2.691374725	-2.119703
10752	CHL1	806.7710357	184.6456129	-2.127400305
1825	DSC3	309.9919846	67.78281367	-2.193239483
5577	PRKAR2B	15.64597469	3.402465343	-2.201139164
22875	ENPP4	111.882344	24.2328925	-2.20694386
91851	CHRDL1	466.1250339	100.3364407	-2.21587133
9627	SNCAIP	13.16898441	2.825201709	-2.22071831
1293	COL6A3	11.61208738	2.403778621	-2.272251393
7504	XK	19.53622844	3.856355665	-2.340841955
2619	GAS1	19.55274855	3.814320202	-2.357873559
4747	NEFL	18.1930377	3.536087886	-2.363160414
2770	GNAI1	93.50841808	17.57532224	-2.411545108
58494	JAM2	312.7466928	54.97129838	-2.508244168
25928	SOSTDC1	298.0195151	52.03017373	-2.517986376
5087	PBX1	89.77340373	15.36475954	-2.546662907
4045	LSAMP	35.44037398	5.992630231	-2.564132566
84898	PLXDC2	916.4430156	153.2330813	-2.580317384
79937	CNTNAP3	36.39300994	6.04687975	-2.589398582
794	CALB2	60.58637293	9.185640403	-2.721541128
7837	PXDN	68.80044565	9.246388351	-2.895456047

2202	EFEMP1	225.4936186	29.51684403	-2.933476226
6662	SOX9	89.50965629	9.651814882	-3.213171178
2173	FABP7	98.47977919	9.999509538	-3.299898287
2641	GCG	195.3356012	18.7202724	-3.383281581
7412	VCAM1	33.78048417	2.96707213	-3.509078102
220382	FAM181B	218.6937093	8.813520021	-4.633049581

Table S1e

Gene ID	Gene symbol	ctrl Expression	R1284W Expression	log2FoldChange (R1284W/ctrl)
101060389	TBC1D3D	4.29039374	37.6660002	3.134080881
2909	ARHGAP35	3171.226686	14130.78132	2.155728325
285220	EPHA6	6.129605096	19.78484139	1.690529464
4747	NEFL	23.77327305	66.4669869	1.483297411
6005	RHAG	7.191501438	19.0718432	1.407079367
129293	TRABD2A	268.3775459	671.6035705	1.323345929
11075	STMN2	820.4942867	2053.009344	1.323175
1392	CRH	6.286422685	15.06475817	1.26086633
646457	C19orf67	8.111357761	18.83442465	1.21535663
56146	PCDHA2	4.473690879	10.17714351	1.185795208
150221	RIMBP3C	13.77036932	30.59413909	1.15168805
1910	EDNRB	41.36083943	90.38232554	1.127775212
638	BIK	21.63115848	46.22840696	1.095668715
100131390	SP9	10.66347542	22.6633358	1.087682511
429	ASCL1	91.79870105	193.7925516	1.077967477
3753	KCNE1	47.91004948	23.62388895	-1.020081823
1381	CRABP1	121.4493733	59.61266805	-1.026664196
58494	JAM2	312.3208698	147.6039866	-1.081297288
80309	SPHKAP	5.432180831	2.54559931	-1.093526156
130574	LYPD6	6.122901311	2.853373165	-1.101546995
112399	EGLN3	6.386202777	2.916476661	-1.130731833
2619	GAS1	19.44105452	8.836989057	-1.137479673
2641	GCG	195.1424834	88.63500301	-1.13857944
7091	TLE4	15.59428152	7.017957216	-1.151894026
3983	ABLIM1	9.190071177	4.110728473	-1.160681955
4311	MME	21.36985553	9.522944707	-1.166097494
5796	PTPRK	24.6568302	10.64174019	-1.212253257
134429	STARD4	11.19723012	4.780226486	-1.227991015
7504	XK	19.42485303	8.200474539	-1.244124381
28316	CDH20	6.552283881	2.766113908	-1.244137296
7020	TFAP2A	498.4424309	210.4166331	-1.244178134
55553	SOX6	11.48656752	4.816259178	-1.253962813
83857	TMTC1	188.9410166	79.18560069	-1.25462591
4045	LSAMP	35.30115115	14.61654264	-1.272113129
79937	CNTNAP3	36.31131235	14.94961459	-1.280310781
79674	VEPH1	30.63881984	12.22753026	-1.325227693
11170	FAM107A	17.61318414	7.025352764	-1.326013172
579	NKX3-2	102.9175525	40.88790074	-1.331743155
341640	FREM2	36.65179731	14.54184956	-1.33367317
55885	LMO3	6.97179688	2.688138494	-1.37492307

729408	GAGE2D	6.72847573	2.589744124	-1.377470152
7045	TGFBI	23.11372311	8.819461698	-1.389987157
26011	TENM4	13.27256068	5.008029729	-1.406131705
127833	SYT2	99.81612398	37.57210264	-1.409611027
203447	NRK	12.56854373	4.724000478	-1.411736484
79953	SYNDIG1	8.262407022	3.048601363	-1.438414617
100528021	ST20-MTHFS	7.79560555	2.86642286	-1.443409639
57381	RHOJ	12.64977468	4.618318485	-1.453672116
2770	GNAI1	92.85880849	33.88172337	-1.454531511
1823	DSC1	100.6854463	36.04498875	-1.481984559
5651	TMPRSS15	16.22643439	5.744639936	-1.498057641
1293	COL6A3	11.06338987	3.894146279	-1.506414518
130576	LYPD6B	25.77636456	8.850699707	-1.542185384
257019	FRMD3	177.7183144	60.38500349	-1.557330155
9627	SNCAIP	12.46875355	4.136020541	-1.592001996
6422	SFRP1	36.2207718	10.37148974	-1.804194153
4921	DDR2	262.8074097	74.52096293	-1.818287731
6662	SOX9	88.46565932	23.9616762	-1.884388704
3730	ANOS1	106.4798119	27.63786305	-1.945861952
10085	EDIL3	17.11888398	4.196929793	-2.028182418
7837	PXDN	67.67708123	16.44576802	-2.040950965
2045	EPHA7	160.8888781	33.6885051	-2.255736282
157638	FAM84B	194.8103723	38.38658697	-2.343396295
2202	EFEMP1	223.6460056	40.22947669	-2.474892115
10202	DHRS2	126.9398716	22.47714452	-2.497614615
2173	FABP7	96.92718736	16.51008121	-2.553554171
7412	VCAM1	32.63304282	5.295827041	-2.623405605
220382	FAM181B	216.6086404	23.00691091	-3.234951598

Table S1f

Gene ID	Gene symbol	E400K Expression	R1284W Expression	log2FoldChange (R1284W/E400K)
4747	NEFL	4.676169113	66.1382624	3.822086132
429	ASCL1	34.24518101	193.6068988	2.49915747
285220	EPHA6	3.624712968	20.03560157	2.466627166
6005	RHAG	3.911443611	19.44513703	2.31363633
169166	SNX31	24.64707847	120.0346561	2.283962445
25928	SOSTDC1	52.56052224	250.2811335	2.251498028
7180	CRISP2	3.128769282	14.59604506	2.221910328
11075	STMN2	450.3626411	2057.246401	2.191555538
2641	GCG	18.53206809	83.4475446	2.170845712
55170	PRMT6	15.59472981	68.78317746	2.140997206
10752	CHL1	186.9476622	823.0901913	2.138416094
9074	CLDN6	238.9374461	1016.980692	2.089587414
22797	TFEC	8.314279851	34.66954522	2.060005698
6335	SCN9A	68.41272862	279.5050064	2.030537447
84898	PLXDC2	153.342346	612.526293	1.998015616
794	CALB2	9.248647688	36.40080863	1.97665616
1946	EFNA5	18.7033547	73.58195023	1.976054854
124925	SEZ6	11.6345895	45.3792142	1.963611317
22837	COBLL1	251.8960702	977.0808829	1.955649378
2904	GRIN2B	6.458728023	24.53563232	1.925556478
153579	BTNL9	9.735461099	36.97557848	1.925251503
22875	ENPP4	24.18044333	91.48439505	1.919684981
6332	SCN7A	49.73793563	184.0929147	1.888015567
54551	MAGEL2	15.04383936	54.07660311	1.845831723
118427	OLFM3	37.16574416	132.2258011	1.830958316
56776	FMN2	42.65054131	151.6000336	1.829634116
57188	ADAMTSL3	30.02454722	102.8625746	1.776503774
50863	NTM	217.63579	718.8538729	1.723782706
152273	FGD5	27.24908984	89.37894951	1.713727046
1307	COL16A1	68.12603533	219.3177337	1.686744316
1272	CNTN1	6.96662353	22.32998804	1.68045097
5655	KLK10	21.80490347	68.83430038	1.658475041
1482	NKX2-5	18.53767105	58.46275372	1.65705778
9510	ADAMTS1	5.030621138	15.62865427	1.635385111
8492	PRSS12	61.91990006	191.9873799	1.632536432
91851	CHRDL1	100.2810667	309.007234	1.623591365
8128	ST8SIA2	5.403605432	16.58045823	1.61748964
1404	HAPLN1	40.3962465	123.5632931	1.612957072
56979	PRDM9	276.0236946	838.7054581	1.603372128
29951	PDZRN4	80.10242515	243.1626323	1.602003715

131368	ZPLD1	5.658887023	16.3236675	1.528374989
23022	PALLD	269.891907	775.2762174	1.522328692
1902	LPAR1	39.33192368	111.5481905	1.503894457
26136	TES	224.3484606	635.143458	1.501341201
148113	CILP2	88.15276721	244.8064046	1.473563539
5087	PBX1	15.03336288	41.57025283	1.467383754
24	ABCA4	3.470012619	9.544172175	1.45967916
1392	CRH	5.626394113	15.43029445	1.455483074
26085	KLK13	4.50528153	12.14984539	1.431248789
117245	HRASLS5	170.2171469	458.2880945	1.428878433
55796	MBNL3	65.53021035	175.569832	1.421812902
51309	ARMCX1	29.73439155	78.59632291	1.402329264
126326	GIPC3	11.56703119	30.51605189	1.399549694
58494	JAM2	54.41438204	142.938782	1.39333748
57172	CAMK1G	6.660852258	17.40585395	1.385793909
55203	LGI2	65.2052794	168.998046	1.373945883
9900	SV2A	58.20496327	150.3639426	1.369244564
79987	SVEP1	1.95987684	5.042434876	1.363357549
3485	IGFBP2	413.9560293	1063.188689	1.360848224
9844	ELMO1	64.69788626	165.946862	1.358930865
728747	ANKRD20A4	14.6490382	37.23680458	1.345923329
1832	DSP	425.2743157	1060.836853	1.318737168
2158	F9	2.018917013	5.021442235	1.314520177
4593	MUSK	10.06762216	24.92170509	1.307679799
170689	ADAMTS15	113.4796503	280.3012968	1.304544809
57348	TTYH1	309.0217338	758.4127906	1.295274988
23657	SLC7A11	64.43164531	158.0122846	1.294195384
24141	LAMP5	44.45383024	108.975987	1.293630635
100131390	SP9	9.314349376	22.75791315	1.288841369
9388	LIPG	66.98910996	163.4718423	1.287043667
51334	PRR16	214.3383896	519.0792376	1.276064513
54413	NLGN3	87.59973037	211.3135422	1.270386892
442213	PTCHD4	1.863609829	4.469658028	1.262064611
91351	DDX60L	60.65388982	145.4690944	1.262040603
647042	GOLGA6L10	9.033926962	21.62864254	1.259517966
1462	VCAN	8.256821281	19.7459973	1.257901852
116984	ARAP2	4.325277066	10.32194889	1.254850936
2898	GRIK2	8.261754202	19.70032866	1.253699655
642475	MROH6	63.81757278	152.0753613	1.252760788
55601	DDX60	14.66971235	34.58670909	1.237377166
57514	ARHGAP31	99.17901164	233.7642341	1.236947461
5167	ENPP1	108.371671	254.1228638	1.229538506
2122	MECOM	37.3758829	87.47237303	1.226719774

26002	MOXD1	357.6385213	835.5811427	1.224277796
2028	ENPEP	22.22371589	51.77487812	1.220152191
56243	KIAA1217	4.504633472	10.49327331	1.219983159
646457	C19orf67	8.327193199	19.31231409	1.213618843
1910	EDNRB	39.64864901	91.25923626	1.202698872
59336	PRDM13	10.01310323	23.00886642	1.200300749
23150	FRMD4B	83.39149164	191.0778224	1.196188241
23327	NEDD4L	34.31508034	78.32205413	1.19057587
57216	VANGL2	118.7916132	271.0513457	1.190133185
220382	FAM181B	7.227286643	16.44700601	1.186298962
1825	DSC3	67.03119212	152.5011635	1.185915753
23114	NFASC	96.57228258	217.3871415	1.170585525
6328	SCN3A	22.02417516	49.56308783	1.170178083
11230	PRAF2	185.4925407	417.3322283	1.169835164
84675	TRIM55	11.31328258	25.32724676	1.162672663
129293	TRABD2A	302.0472157	674.1895678	1.158380216
6480	ST6GAL1	540.4588638	1201.39037	1.152448286
2	A2M	18.82875987	41.84181465	1.15200744
10409	BASP1	140.5223516	312.2503879	1.15190374
222967	RSPH10B	2.284981592	5.076915309	1.151769651
57512	GPR158	386.6501489	858.5138655	1.150812666
51481	VCX3A	2.264826431	5.024900449	1.149694524
54511	HMGCLL1	143.6852867	318.7577513	1.149548086
9435	CHST2	12.8541658	28.48348928	1.147889903
6340	SCNN1G	1037.973625	2296.067598	1.145395331
653	BMP5	222.673819	491.3625725	1.141856028
30817	ADGRE2	12.55095454	27.69432414	1.141793241
64073	C19orf33	23.07633845	50.90222794	1.141314475
4638	MYLK	74.23083482	162.5944714	1.131187704
548596	CKMT1A	215.5808649	471.1458251	1.127944531
2888	GRB14	90.30527816	196.80156	1.123859439
80854	SETD7	177.4789419	385.9473998	1.120756381
114907	FBXO32	44.86367643	96.71033948	1.10812229
387104	SOGA3	146.1100988	313.720825	1.102425402
8612	PLPP2	34.79700822	74.53234212	1.098903325
6550	SLC9A3	624.6583878	1331.825761	1.092266019
30010	NXPH1	29.39089013	61.51843485	1.065649746
388585	HES5	94.93920262	198.2819021	1.062477165
6338	SCNN1B	2673.368086	5579.364132	1.06144222
2330	FMO5	69.21015798	144.2549911	1.059565533
771	CA12	146.0199232	304.1316112	1.058530551
55760	DHX32	115.1876299	239.5105568	1.056103454
1602	DACH1	177.6559316	369.1154621	1.054986314

1159	CKMT1B	88.17433014	182.5685759	1.050007851
221935	SDK1	36.59064737	75.69131692	1.048652868
10769	PLK2	1492.428521	3084.983532	1.047600951
57608	JCAD	63.31180567	130.8150126	1.046981669
80059	LRRTM4	17.30234897	35.718274	1.045694456
4843	NOS2	81.81141805	168.7041927	1.044121716
10893	MMP24	61.42969196	126.4610383	1.041684916
7903	ST8SIA4	40.95540329	84.29035198	1.041313707
167410	LIX1	495.4057606	1011.562817	1.029903364
9180	OSMR	28.08554374	57.23921702	1.027176204
23025	UNC13A	335.5607117	683.2841273	1.025911802
6546	SLC8A1	50.1834663	101.680018	1.018752161
23213	SULF1	369.2907946	746.3823351	1.015157541
9249	DHRS3	14.35360107	28.97673261	1.013482198
8676	STX11	81.83770378	164.8693584	1.010485722
57556	SEMA6A	1007.934222	2029.833827	1.009960134
81578	COL21A1	32.29620977	64.96353151	1.008265199
638	BIK	23.34286696	46.9378231	1.00776917
203328	SUSD3	24.26540278	48.7197666	1.005606414
5624	PROC	23.97526561	47.98236143	1.000957363
85450	ITPRIP	89.53709713	179.1424111	1.000549478
284076	TTLL6	308.8910961	153.9474261	-1.004660539
140767	NRSN1	73.97815271	36.41998279	-1.022369035
6549	SLC9A2	38.26842003	18.58826004	-1.041762605
131149	OTOL1	163.5883997	79.02848836	-1.04962573
56143	PCDHA5	44.6513278	21.5272005	-1.052542361
643699	GOLGA8N	267.8231417	128.3252429	-1.061475633
144347	RFLNA	130.6011687	62.36289452	-1.066408011
388662	SLC6A17	29.30327987	13.79748726	-1.086656599
55753	OGDHL	1755.911694	798.8137621	-1.1362892
84419	C15orf48	18.3452507	8.211043917	-1.159769063
56171	DNAH7	122.5991593	54.1454035	-1.179038311
9235	IL32	43.33508524	18.7694008	-1.207152948
57381	RHOJ	8.898152127	3.701665788	-1.265331119
3983	ABLIM1	9.173341205	3.788366591	-1.275871359
113457	TUBA3D	19.5702673	7.987274102	-1.292888332
115701	ALPK2	12.7575792	5.155533286	-1.307161026
27092	CACNG4	72.81869875	29.18920265	-1.318874159
728392	LOC728392	269.6402964	104.3266699	-1.369928103
23768	FLRT2	14.13240763	5.444312065	-1.376185599
22865	SLITRK3	583.6143861	218.0584432	-1.420300594
391195	OR2T33	6.041675702	2.250369018	-1.424787151
51704	GPRC5B	1903.742345	659.5256074	-1.529337654

26052	DNM3	139.344524	47.03527383	-1.5668413
6588	SLN	40.21165074	12.29713723	-1.709291066
1356	CP	27.22353865	7.965634999	-1.772993329
431707	LHX8	94.91935294	24.62671883	-1.946477845
51441	YTHDF2	13340.96738	3444.421462	-1.953529695
4916	NTRK3	62.37883575	15.68284271	-1.991869537
343172	OR2T8	12.2622678	3.013214721	-2.02485043
25893	TRIM58	141.6203023	34.2890549	-2.046208056
130399	ACVR1C	80.57300936	18.43034414	-2.128213631
257019	FRMD3	300.2314693	60.73350847	-2.305510585
1103	CHAT	57.84858219	9.817670922	-2.558828886
6572	SLC18A3	238.4275385	35.30207164	-2.755726125
2045	EPHA7	271.7021246	33.79503401	-3.007142676
401335	C7orf65	48.64103076	5.46154675	-3.154792306
57554	LRRC7	104.8420434	10.96646686	-3.257046675
5592	PRKG1	116.7840265	10.98104878	-3.410755203

Table S2a

Gene ID	Gene Symbol	Idctrl Expression	Idp190A Expression	log2FoldChange (Idp190A/Idctrl)
388436	LOC388436	0.01	9.31	9.862637358
55170	PRMT6	0.36	4.41	3.614709844
1800	DPEP1	1.49	15.95	3.420172188
2303	FOXC2	0.41	3.7	3.173829456
2022	ENG	0.94	7.28	2.953205789
26289	AK5	0.52	3.97	2.932555479
4222	MEOX1	3.18	23.91	2.910515459
10568	SLC34A2	4.11	23.27	2.501260912
800	CALD1	1.78	9.98	2.487162574
89927	C16orf45	3.58	19.99	2.481246979
2909	ARHGAP35	8.96	49.38	2.462356199
55567	DNAH3	0.71	3.74	2.39714734
9211	LGI1	1.06	5.48	2.370111628
3237	HOXD11	1.76	8.56	2.282035368
6588	SLN	2.44	11.86	2.281150957
84709	MGARP	2.19	10.55	2.268240224
57522	SRGAP1	1.62	7.6	2.230005605
56892	TCIM	3.5	15.94	2.187224802
11098	PRSS23	6.13	27.06	2.14220286
7164	TPD52L1	3.31	14.51	2.132144397
64073	C19orf33	2.57	11.15	2.117203446
11346	SYNPO	1.73	7.47	2.110336205
55824	PAG1	2.77	11.83	2.094492192
5327	PLAT	2.88	11.76	2.029747343
1001	CDH3	21.62	87.43	2.015761876
1277	COL1A1	33.94	134.25	1.983863618
22822	PHLDA1	10.9	43.01	1.980343996
548596	CKMT1A	13.98	54.02	1.950129279
80326	WNT10A	3.45	13.17	1.932587079
11240	PADI2	3.6	13.68	1.925999419
11010	GLIPR1	2.87	10.84	1.917242115
5155	PDGFB	5.4	19.86	1.87883431
7784	ZP3	2.83	10.38	1.874932486
140706	CCM2L	5.48	19.77	1.851065073
26115	TANC2	4.51	15.36	1.767978877
7042	TGFB2	8.35	27.88	1.739382459
51232	CRIM1	22.3	74.33	1.736900898
81029	WNT5B	23.51	76.59	1.703881499
6277	S100A6	42.75	137.27	1.683020038
1435	CSF1	7.61	23.83	1.646810593

4330	MN1	4.25	13.27	1.642633624
130576	LYPD6B	3.34	10.35	1.63171076
6275	S100A4	2.7	8.25	1.611434712
4137	MAPT	3.26	9.81	1.589381172
83853	ROPN1L	4.24	12.6	1.571287564
2042	EPHA3	9.46	27.61	1.545278799
221662	RBM24	5.43	15.7	1.531740456
4884	NPTX1	6.15	17.65	1.521009868
10536	P3H3	4.26	12.09	1.504888909
51286	CEND1	3.98	11.28	1.502926732
4907	NT5E	7.19	20.14	1.486000008
1159	CKMT1B	6.5	18.14	1.480662833
169611	OLFML2A	5.42	15.05	1.47339873
11230	PRAF2	10.17	28.09	1.465736945
2641	GCG	21.83	59.57	1.448273829
51316	PLAC8	7.58	20.39	1.427592022
3908	LAMA2	5.27	13.81	1.389838453
100528064	NEDD8-MDP1	5.02	13.15	1.38930353
64759	TNS3	16.2	42.16	1.379881054
6939	TCF15	6.78	17.37	1.357240576
3897	L1CAM	25.86	65.31	1.336581633
3875	KRT18	45.07	111.9	1.311970681
5106	PCK2	8.01	19.53	1.285817801
100133941	CD24	9.09	21.74	1.257999741
26509	MYOF	9.22	21.88	1.246774082
3306	HSPA2	76.23	174.86	1.197769523
6320	CLEC11A	26.68	60.92	1.191157275
6282	S100A11	125.17	285.2	1.188085155
283869	NPW	33.89	76.04	1.165898895
5836	PYGL	39.1	86.96	1.153183333
8407	TAGLN2	195.93	421.65	1.10570764
2296	FOXC1	20.43	43.8	1.100241666
1508	CTSB	72.93	155.6	1.093257761
1278	COL1A2	270.39	562.63	1.057144684
5101	PCDH9	52.6	25.71	-1.032733189
1490	CTGF	791.49	363.87	-1.121148013
91419	ATP23	34.17	15.01	-1.186806271
9353	SLIT2	21.46	9.19	-1.223513309
81848	SPRY4	30.72	13.02	-1.238448768
442247	RFPL4B	207.5	87.03	-1.253526635
116039	OSR2	63.59	26.65	-1.254664376
8905	AP1S2	33.65	13.97	-1.268274484
8490	RGS5	86.9	35.26	-1.301323702

256691	MAMDC2	31.17	12.1	-1.365151107
55843	ARHGAP15	37.45	14.31	-1.387942047
8749	ADAM18	13.2	4.94	-1.417954983
7980	TFPI2	14.76	5.37	-1.458698728
10409	BASP1	11.23	4.05	-1.471364115
169026	SLC30A8	12.04	4.3	-1.485426827
138255	C9orf135	18.85	6.72	-1.488031385
3485	IGFBP2	24.58	8.62	-1.511725141
93986	FOXP2	15.33	5.37	-1.513363704
154141	MBOAT1	14.28	4.97	-1.522678222
5454	POU3F2	24.74	8.4	-1.558384267
171392	ZNF675	16.32	5.52	-1.563900885
287	ANK2	10.6	3.52	-1.590416931
1021	CDK6	7.74	2.54	-1.607505069
10253	SPRY2	46.71	15.29	-1.611143038
1005	CDH7	36.07	11.75	-1.618138666
4237	MFAP2	15.64	5.03	-1.636610208
1004	CDH6	215.08	65.9	-1.706523006
55630	SLC39A4	18	5.49	-1.713118852
9315	NREP	14.57	4.44	-1.714369296
10631	POSTN	79.21	23.68	-1.742013496
710	SERPING1	39.52	11.7	-1.756074417
117166	WFIKKN1	8.25	2.26	-1.868071347
119710	C11orf74	6.32	1.54	-2.036994207
6857	SYT1	7.46	1.81	-2.043185933
3208	HPCA	10.12	2.44	-2.052256237
171024	SYNPO2	8.94	2.11	-2.083031832
165215	FAM171B	48.5	11.27	-2.105497232
1373	CPS1	24.08	5.59	-2.106915204
7098	TLR3	4.53	1.05	-2.109121722
1000	CDH2	5.88	1.36	-2.112209504
10468	FST	44.47	10.28	-2.11299214
50863	NTM	10.99	2.42	-2.183112434
100526737	RBM14-RBM4	5.27	1.16	-2.183678157
3912	LAMB1	10.95	2.41	-2.183825818
2202	EFEMP1	4.82	0.99	-2.283532716
2047	EPHB1	7.4	1.44	-2.361456459
55760	DHX32	5.71	1.09	-2.389162611
9427	ECEL1	11.34	2.1	-2.432959407
10251	SPRY3	6.67	1.22	-2.450805614
7078	TIMP3	4.18	0.7	-2.578076115
5325	PLAGL1	13.94	2.28	-2.612124832
3486	IGFBP3	12.05	1.84	-2.711255475

83643	CCDC3	3.85	0.54	-2.833827133
22837	COBLL1	5.33	0.74	-2.848538357
4664	NAB1	3.86	0.5	-2.948600847
10840	ALDH1L1	4.72	0.61	-2.951905712
23022	PALLD	10.83	1.32	-3.036423408
167410	LIX1	3.61	0.41	-3.138303022
51050	PI15	10.7	1.2	-3.156504486
83690	CRISPLD1	21.97	1.7	-3.691928218
799	CALCR	4.68	0.36	-3.700439718
9074	CLDN6	10.88	0.56	-4.280107919
25928	SOSTDC1	3.76	0.13	-4.854149134
7001	PRDX2	5.61	0.19	-4.883929447
339479	BRINP3	6.24	0.2	-4.963474124
64849	SLC13A3	3.09	0.07	-5.464108106
1535	CYBA	19.02	0.38	-5.645374017
441521	CT45A5	3.86	0.01	-8.592457037
79999	LOC79999	8.66	0.01	-9.758223215

Table S2b

Gene ID	Gene Symbol	Idecadkd Expression	Idp190A Expression	log2FoldChange (Idp190A/Idecadkd)
5098	PCDHGC3	0.18	6.91	5.262616899
84419	C15orf48	0.49	4.34	3.146841388
2022	ENG	0.83	7.28	3.132755209
257019	FRMD3	0.47	3.82	3.022839976
7164	TPD52L1	2.18	14.51	2.734647479
26289	AK5	0.6	3.97	2.726104602
64073	C19orf33	1.88	11.15	2.568239143
51700	CYB5R2	1.29	7.26	2.492598483
4222	MEOX1	4.57	23.91	2.38734806
89927	C16orf45	3.88	19.99	2.365149915
1277	COL1A1	26.76	134.25	2.326772067
150763	GPAT2	1.53	7.28	2.250406798
728340	GTF2H2C	1.69	7.99	2.241172257
2641	GCG	12.75	59.57	2.224088712
81035	COLEC12	10.78	47.23	2.131346357
9235	IL32	1.12	4.87	2.12042304
8839	WISP2	1.01	4.39	2.119865647
9289	ADGRG1	1.46	6.16	2.076961982
10568	SLC34A2	5.52	23.27	2.075731039
4313	MMP2	2.54	10.36	2.028123601
6820	SULT2B1	1.12	4.47	1.996776099
54738	FEV	1.06	4.23	1.996593399
6588	SLN	3.03	11.86	1.968714311
9084	VCY	6.7	26.02	1.957387961
5328	PLAU	4.75	17.18	1.854730618
1382	CRABP2	61.15	207.83	1.764979517
2537	IFI6	37.3	124	1.733092585
4907	NT5E	6.41	20.14	1.651667421
7134	TNNC1	7.4	22.75	1.620269369
4062	LY6H	3.33	10.16	1.60930632
140706	CCM2L	6.48	19.77	1.609247153
283869	NPW	25.86	76.04	1.556036257
654364	NME1-NME2	6.48	18.81	1.537434131
6320	CLEC11A	21.44	60.92	1.506611036
3875	KRT18	40.8	111.9	1.455568979
6939	TCF15	6.44	17.37	1.431465161
163732	CITED4	31.18	83.29	1.417522364
3281	HSBP1	17.08	45.57	1.415776396
2301	FOXE3	5.01	13.12	1.388885211
5176	SERPINF1	12.65	31.93	1.335775168

6720	SREBF1	43.46	108.53	1.32033381
100	ADA	17.3	40.85	1.239564041
548596	CKMT1A	22.99	54.02	1.232487174
6319	SCD	65.78	150.82	1.197106841
4232	MEST	14.63	33.14	1.179643833
151195	CCNYL1	19.04	43.12	1.179323699
6277	S100A6	62.74	137.27	1.129558928
11010	GLIPR1	25.41	10.84	-1.229031619
347475	CCDC160	16.26	6.33	-1.361049853
3218	HOXB8	18.82	7.22	-1.382195886
631	BFSP1	16.42	6.23	-1.398150059
23414	ZFPM2	13.15	4.87	-1.433069122
165215	FAM171B	31.56	11.27	-1.48560969
10253	SPRY2	43.26	15.29	-1.500445259
54532	USP53	22.43	7.89	-1.507332415
143903	LAYN	44.65	15.01	-1.572736198
102724594	U2AF1L5	8.21	2.59	-1.664430124
256691	MAMDC2	41.43	12.1	-1.775668773
10468	FST	37.04	10.28	-1.849243834
171024	SYNPO2	8.2	2.11	-1.958380911
5796	PTPRK	6.89	1.76	-1.968928554
258010	SVIP	9.08	2.24	-2.019193565
79839	CCDC102B	4.99	1.2	-2.05600541
18	ABAT	8.3	1.96	-2.082257682
119710	C11orf74	6.64	1.54	-2.108252891
3912	LAMB1	10.94	2.41	-2.182507687
5325	PLAGL1	11.57	2.28	-2.343283135
4237	MFAP2	27.44	5.03	-2.447650176
55760	DHX32	6.27	1.09	-2.524137308
2846	LPAR4	6.91	1.2	-2.525651305
1410	CRYAB	54.42	8.45	-2.68711371
100526737	RBM14-RBM4	8.69	1.16	-2.905231372
7980	TFPI2	40.76	5.37	-2.924160058
167410	LIX1	4.09	0.41	-3.318405028
4664	NAB1	5.77	0.5	-3.528571319
1368	CPM	7.89	0.64	-3.62388149
11166	SOX21	6.23	0.34	-4.195625512
8631	SKAP1	5.32	0.27	-4.300394933
170825	GSX2	3.24	0.08	-5.339850003
4857	NOVA1	24.95	0.61	-5.354086763
5156	PDGFRA	5.48	0.12	-5.513069582
799	CALCR	17.62	0.36	-5.613073207
794	CALB2	10.79	0.11	-6.616047531

220382	FAM181B	26.48	0.18	-7.200762405
79999	LOC79999	4.94	0.01	-8.948367232

Table S2c

Gene ID	Gene Symbol	ldctrl Expression	ldecadkd Expression	log2FoldChange (ldecadkd/ldctrl)
388436	LOC388436	0.01	3.69	8.527477006
2846	LPAR4	0.02	6.91	8.4325419
59277	NTN4	0.17	4.5	4.72631835
220382	FAM181B	1.86	26.48	3.831528596
5796	PTPRK	0.49	6.89	3.813650329
8631	SKAP1	0.46	5.32	3.531720479
2303	FOXC2	0.41	4.4	3.423807709
800	CALD1	1.78	15.99	3.167220793
11010	GLIPR1	2.87	25.41	3.146273733
1368	CPM	1.01	7.89	2.965670007
4857	NOVA1	3.49	24.95	2.837740874
11166	SOX21	1.07	6.23	2.541621367
23670	TMEM2	0.94	5.42	2.52756019
2909	ARHGAP35	8.96	51.62	2.526359504
1800	DPEP1	1.49	8.3	2.477799006
28951	TRIB2	1.65	8.28	2.327164743
57522	SRGAP1	1.62	8.06	2.314786026
51196	PLCE1	1.89	9.08	2.264306063
1956	EGFR	1.2	5.74	2.258016331
122060	SLAIN1	1.14	5.28	2.211504105
143903	LAYN	9.75	44.65	2.195186051
794	CALB2	2.39	10.79	2.174612342
64065	PERP	1.7	7.38	2.11808607
1410	CRYAB	12.97	54.42	2.068958477
23213	SULF1	1.73	7.25	2.067208957
5327	PLAT	2.88	11.68	2.019899557
55796	MBNL3	1.96	7.83	1.998158653
799	CALCR	4.68	17.62	1.912633489
51232	CRIM1	22.3	80.65	1.854630823
347902	AMIGO2	2.98	10.59	1.829318354
26115	TANC2	4.51	15.85	1.813283502
85016	C11orf70	2.51	8.8	1.80981616
170689	ADAMTS15	2.55	8.93	1.808162928
3959	LGALS3BP	2.31	7.57	1.712400449
56892	TCIM	3.5	11.46	1.711180217
55824	PAG1	2.77	8.9	1.68391936
7042	TGFB2	8.35	26.13	1.645859022
2171	FABP5	17.95	55.25	1.62199062
6340	SCNN1G	4.31	13.15	1.609303025
51375	SNX7	27.71	82.4	1.572237627

9659	PDE4DIP	5.76	16.87	1.550319257
26609	VCX	4.82	13.93	1.531090206
3306	HSPA2	76.23	213.4	1.485129395
22822	PHLDA1	10.9	30.13	1.466872538
7980	TFPI2	14.76	40.76	1.46546133
3218	HOXB8	6.9	18.82	1.447598361
102724428	SIK1B	4.88	13.29	1.445388052
8651	SOCS1	7.77	20.38	1.391167548
11098	PRSS23	6.13	15.89	1.374160146
29761	USP25	15.27	39.56	1.373342364
5106	PCK2	8.01	20.64	1.365568823
1827	RCAN1	5.16	13.21	1.356187496
23705	CADM1	15.14	37.81	1.320402644
2273	FHL1	15.08	36.56	1.277629642
81029	WNT5B	23.51	56.09	1.254469044
5376	PMP22	26.8	63.7	1.249060372
23231	SEL1L3	13.94	31.44	1.173370656
23089	PEG10	159.9	342.62	1.099439431
2719	GPC3	275.55	570.27	1.049331012
1278	COL1A2	270.39	542.03	1.003330907
10933	MORF4L1	140.14	69.6	-1.00970959
8724	SNX3	226.12	105.77	-1.096158114
2537	IFI6	82.56	37.3	-1.14626734
163732	CITED4	69.29	31.18	-1.152026228
497661	C18orf32	21.9	9.19	-1.252794103
411	ARSB	16.82	6.73	-1.321499296
1004	CDH6	215.08	85.09	-1.337811879
50861	STMN3	22.8	8.89	-1.3587785
10681	GNB5	15.15	5.44	-1.477639237
3281	HSBP1	50.55	17.08	-1.565403117
8749	ADAM18	13.2	4.23	-1.641808361
9427	ECEL1	11.34	3.58	-1.663389148
9060	PAPSS2	12.04	3.79	-1.667565639
11013	TMSB15A	9.59	2.93	-1.710630151
3486	IGFBP3	12.05	3.68	-1.711255475
7134	TNNC1	24.37	7.4	-1.719509075
23022	PALLD	10.83	3.25	-1.73652162
93986	FOXP2	15.33	4.59	-1.739791638
27122	DKK3	8.37	2.39	-1.808217005
9022	CLIC3	8.07	2.1	-1.942179346
3208	HPCA	10.12	2.43	-2.058181071
9084	VCY	28.91	6.7	-2.109335608
339479	BRINP3	6.24	1.44	-2.115477217

10409	BASP1	11.23	2.57	-2.127517663
4062	LY6H	15.04	3.33	-2.175210485
3485	IGFBP2	24.58	4.81	-2.353376117
81035	COLEC12	57.74	10.78	-2.421213929
2047	EPHB1	7.4	1.37	-2.433349378
83690	CRISPLD1	21.97	4.06	-2.435983237
1382	CRABP2	335.41	61.15	-2.455501299
2202	EFEMP1	4.82	0.84	-2.520571913
103344718	HOTS	4.29	0.63	-2.767553914
710	SERPING1	39.52	5.76	-2.77844223
728340	GTF2H2C	11.77	1.69	-2.800019169
9315	NREP	14.57	2.09	-2.80142603
50863	NTM	10.99	1.35	-3.025160074
2888	GRB14	3.47	0.4	-3.116863758
22837	COBLL1	5.33	0.57	-3.225101709
9074	CLDN6	10.88	0.51	-4.415037499
5098	PCDHGC3	3.96	0.18	-4.459431619
1535	CYBA	19.02	0.8	-4.571373436
7078	TIMP3	4.18	0.14	-4.90000421
80712	ESX1	3.45	0.06	-5.845490051
7001	PRDX2	5.61	0.05	-6.809928866
441521	CT45A5	3.86	0.01	-8.592457037

Table S2d

Gene ID	Gene Symbol	hdctrl Expression	hdp190A Expression	log2FoldChange (hdp190A/hdctrl)
8293	SERF1A	0.01	4.703333333	8.877539772
24150	TP53TG3	0.013333333	1.076666667	6.335390355
2846	LPAR4	0.03	2.033333333	6.082740431
6588	SLN	0.69	29.99666667	5.44206202
3815	KIT	0.04	1.5	5.22881869
4071	TM4SF1	0.113333333	4.163333333	5.19909492
55024	BANK1	0.043333333	1.236666667	4.834835658
94240	EPSTI1	0.136666667	3.556666667	4.701792456
23544	SEZ6L	0.053333333	1.18	4.46760555
56892	TCIM	1.463333333	32.13333333	4.456740302
5999	RGS4	0.19	3.916666667	4.365555027
2303	FOXC2	0.07	1.38	4.301169535
10873	ME3	0.176666667	3.286666667	4.217523382
3777	KCNK3	0.166666667	3.07	4.203201156
11098	PRSS23	4.07	74.70666667	4.198136292
221981	THSD7A	0.06	1.003333333	4.063694675
51702	PADI3	0.3	4.933333333	4.039528364
4222	MEOX1	6.223333333	101.3666667	4.025751984
140706	CCM2L	4.583333333	66.3	3.854539752
999	CDH1	2.673333333	38.07333333	3.832069243
388436	LOC388436	0.613333333	8.676666667	3.82239764
26289	AK5	0.196666667	2.75	3.80560726
165	AEBP1	0.256666667	3.47	3.756967813
4916	NTRK3	0.173333333	2.176666667	3.650499463
5796	PTPRK	0.33	4.083333333	3.629209414
4629	MYH11	0.34	3.876666667	3.51121004
246	ALOX15	0.376666667	4.146666667	3.460591808
55170	PRMT6	0.45	4.936666667	3.455540328
11010	GLIPR1	0.686666667	7.476666667	3.444713378
55806	HR	0.92	9.696666667	3.397783124
1800	DPEP1	2.963333333	31.03333333	3.388525844
84419	C15orf48	0.143333333	1.5	3.387516437
286	ANK1	1.046666667	9.996666667	3.255645058
23316	CUX2	0.2	1.81	3.177917792
4005	LMO2	0.49	4.33	3.143513371
167838	TXLNB	0.223333333	1.963333333	3.136034633
89927	C16orf45	5.19	45.38333333	3.128356133
59277	NTN4	1.873333333	16.24333333	3.116168187
9148	NEURL1	0.14	1.203333333	3.103537604
960	CD44	0.356666667	3.03	3.086669498

117154	DACH2	0.22	1.85	3.071949842
51286	CEND1	1.713333333	14.07333333	3.038086314
22822	PHLDA1	5.543333333	44.95333333	3.019602429
9211	LGI1	0.63	5.09	3.014241923
22882	ZHX2	0.316666667	2.543333333	3.005683639
182	JAG1	8.463333333	66.42333333	2.972392229
57574	4-Mar	0.193333333	1.51	2.965386245
84709	MGARP	1.73	13.43666667	2.957331339
388662	SLC6A17	0.253333333	1.956666667	2.94928918
5155	PDGFB	1.256666667	9.69	2.946894643
7042	TGFB2	6.013333333	46.18333333	2.941135467
51232	CRIM1	5.103333333	37.26333333	2.868244952
23551	RASD2	0.36	2.62	2.863498
4135	MAP6	2.046666667	14.68666667	2.843158934
8839	WISP2	0.25	1.793333333	2.842643672
80326	WNT10A	3.553333333	24.86	2.806581359
4907	NT5E	2.066666667	14.36666667	2.797347749
340061	TMEM173	0.423333333	2.883333333	2.767871636
55466	DNAJA4	0.356666667	2.426666667	2.766327654
9394	HS6ST1	21.29666667	143.5533333	2.752887287
10046	MAMLD1	0.836666667	5.596666667	2.741842961
57158	JPH2	0.276666667	1.846666667	2.738702735
11240	PADI2	2.72	17.90666667	2.718818247
3306	HSPA2	27.18666667	178.62	2.715922448
9235	IL32	0.4	2.586666667	2.693022247
127294	MYOM3	0.44	2.843333333	2.692007812
339761	CYP27C1	0.41	2.613333333	2.672195339
3489	IGFBP6	3.446666667	21.84	2.663699171
55450	CAMK2N1	2.033333333	12.82	2.656477615
9619	ABCG1	0.996666667	6.253333333	2.649442438
84072	HORMAD1	0.4	2.473333333	2.628384781
83593	RASSF5	0.403333333	2.49	2.626101196
84665	MYPN	0.18	1.11	2.624490865
4608	MYBPH	0.503333333	3.1	2.622682167
1381	CRABP1	6.37	39.05333333	2.616080415
8605	PLA2G4C	0.5	3.053333333	2.610385098
91683	SYT12	1.14	6.906666667	2.598955773
80243	PREX2	0.266666667	1.593333333	2.578938713
1823	DSC1	0.56	3.306666667	2.561878888
2909	ARHGAP35	13.88666667	81.28666667	2.54931839
55531	ELMOD1	0.283333333	1.62	2.515421567
388585	HES5	6.846666667	38.96666667	2.508766843
1956	EGFR	0.99	5.586666667	2.496487313

79836	LONRF3	0.273333333	1.533333333	2.487938046
10568	SLC34A2	6.713333333	37.37333333	2.476908061
5950	RBP4	0.26	1.446666667	2.476149014
1001	CDH3	31.83	175.8866667	2.466187059
4773	NFATC2	1.246666667	6.883333333	2.465031606
1435	CSF1	5.55	30.60333333	2.463129124
57822	GRHL3	0.303333333	1.67	2.460872153
100533105	C8orf44-SGK3	0.24	1.27	2.403722186
1839	HBEGF	15.04333333	78.4	2.381729377
641517	DEFB109B	0.353333333	1.84	2.380604002
8638	OASL	0.74	3.836666667	2.374256252
3106	HLA-B	3.91	20.08666667	2.36099766
8061	FOSL1	1.08	5.516666667	2.352765499
3875	KRT18	22.75	114.88	2.336189204
102724428	SIK1B	2.556666667	12.88333333	2.333169931
4940	OAS3	1.303333333	6.496666667	2.317493578
8991	SELENBP1	0.783333333	3.9	2.315775868
55567	DNAH3	0.416666667	2.066666667	2.310340121
7043	TGFB3	1.226666667	6.05	2.302191877
6236	RRAD	0.353333333	1.74	2.299985542
4330	MN1	6.373333333	31.36	2.298805537
9478	CABP1	1.273333333	6.26	2.29755252
2247	FGF2	4.12	20.21333333	2.29459101
283948	NHLRC4	0.383333333	1.873333333	2.288936269
51809	GALNT7	0.293333333	1.43	2.285402219
639	PRDM1	0.313333333	1.526666667	2.284614936
51435	SCARA3	0.926666667	4.463333333	2.267999173
8651	SOCS1	1.37	6.596666667	2.267561313
627	BDNF	0.603333333	2.886666667	2.258377328
728118	NUTM2A	0.246666667	1.176666667	2.254071008
11346	SYNPO	2.283333333	10.86333333	2.250253459
3233	HOXD4	0.306666667	1.456666667	2.247927513
9052	GPRC5A	0.55	2.576666667	2.22800239
266727	MDGA1	0.273333333	1.273333333	2.219876823
2022	ENG	2.453333333	11.19666667	2.190254124
54453	RIN2	0.83	3.766666667	2.182105125
4060	LUM	0.23	1.04	2.176877762
57094	CPA6	0.78	3.526666667	2.176759193
5328	PLAU	4.79	21.63333333	2.175158416
140876	RIPOR3	4.506666667	20.26333333	2.168738867
4921	DDR2	0.933333333	4.19	2.166485917
24141	LAMP5	2.163333333	9.69	2.163240688
387723	C10orf143	0.26	1.163333333	2.161681007

23708	GSPT2	1.15	5.136666667	2.159198595
219595	FOLH1B	0.5	2.23	2.15704371
146	ADRA1D	0.393333333	1.753333333	2.15627594
5698	PSMB9	1.48	6.58	2.152490408
1991	ELANE	2.19	9.716666667	2.149530608
162494	RHBDL3	5.486666667	24.29666667	2.146756565
3237	HOXD11	1.613333333	7.133333333	2.144531844
9573	GDF3	0.236666667	1.04	2.135655099
50651	SLC45A1	2.423333333	10.61666667	2.131266103
57522	SRGAP1	3.553333333	15.53666667	2.128432075
2346	FOLH1	2.77	12.07666667	2.124264424
771	CA12	3.48	15.16	2.123110542
54625	PARP14	0.643333333	2.8	2.12178848
26115	TANC2	6.943333333	30.15333333	2.118617157
1840	DTX1	2.173333333	9.416666667	2.115306998
5327	PLAT	3.82	16.52	2.112569143
57540	DISP3	0.6	2.59	2.109917692
800	CALD1	0.966666667	4.16	2.105493129
2334	AFF2	1.62	6.936666667	2.098248746
9729	KIAA0408	0.286666667	1.226666667	2.097297201
53335	BCL11A	1.52	6.48	2.091922489
4313	MMP2	0.91	3.833333333	2.074661005
2983	GUCY1B1	2.103333333	8.853333333	2.073543236
2048	EPHB2	1.14	4.793333333	2.071995446
64759	TNS3	13.72	57.65666667	2.07120695
11043	MID2	1.036666667	4.323333333	2.060191994
84969	TOX2	1.673333333	6.97	2.058433793
64135	IFIH1	0.356666667	1.473333333	2.046435573
26230	TIAM2	0.743333333	3.066666667	2.044590151
124925	SEZ6	0.4	1.65	2.044394119
161145	TMEM229B	0.386666667	1.583333333	2.033802708
7262	PHLDA2	1.003333333	4.103333333	2.03199537
3437	IFIT3	0.373333333	1.526666667	2.031848866
25945	NECTIN3	0.436666667	1.783333333	2.02997208
1303	COL12A1	0.853333333	3.446666667	2.01402047
134	ADORA1	0.26	1.05	2.0138058
28996	HIPK2	0.883333333	3.566666667	2.013546532
146664	MGAT5B	3.54	14.27333333	2.01150103
2669	GEM	0.453333333	1.826666667	2.010569242
330	BIRC3	0.483333333	1.94	2.004966253
83666	PARP9	1.243333333	4.953333333	1.99418658
28951	TRIB2	2.273333333	9.056666667	1.994170921
26064	RAI14	1.78	7.036666667	1.983014932

3965	LGALS9	0.46	1.813333333	1.978938384
221061	FAM171A1	1.686666667	6.646666667	1.97845612
84913	ATOH8	0.286666667	1.126666667	1.974614682
143941	TTC36	0.756666667	2.966666667	1.971113039
9423	NTN1	3.303333333	12.91666667	1.967239348
23220	DTX4	0.27	1.053333333	1.963930745
83849	SYT15	0.696666667	2.713333333	1.961525852
6752	SSTR2	0.546666667	2.12	1.955330951
1277	COL1A1	39.85666667	153.6866667	1.947099044
10761	PLAC1	1.59	6.073333333	1.933461788
3959	LGALS3BP	15.04	57.26333333	1.928807086
5787	PTPRB	0.273333333	1.04	1.927850214
55824	PAG1	6.143333333	23.26	1.920757527
58985	IL22RA1	0.413333333	1.563333333	1.919247802
347902	AMIGO2	0.963333333	3.64	1.917831458
56112	PCDHGA3	3.356666667	12.64333333	1.913275688
9469	CHST3	4.79	17.97333333	1.907760435
26030	PLEKHG3	2.413333333	9.033333333	1.904231249
2621	GAS6	5.95	22.21666667	1.900680801
2766	GMPR	0.55	2.05	1.898120386
1141	CHRNB2	0.34	1.26	1.889817082
342132	ZNF774	0.71	2.606666667	1.876315177
360	AQP3	0.35	1.283333333	1.874469118
3707	ITPKB	0.556666667	2.04	1.87368355
91624	NEXN	5.02	18.36333333	1.871068693
6422	SFRP1	0.393333333	1.436666667	1.86890101
27129	HSPB7	1.433333333	5.216666667	1.863754092
153571	C5orf38	1.086666667	3.946666667	1.860725211
440689	HIST2H2BF	1.216666667	4.413333333	1.858934753
284076	TTLL6	4.073333333	14.74	1.85545474
221662	RBM24	7.686666667	27.81333333	1.855346645
5457	POU4F1	0.343333333	1.24	1.852658284
3235	HOXD9	1.856666667	6.7	1.851446269
169611	OLFML2A	6.853333333	24.71666667	1.850606428
53637	S1PR5	10.88	39.01333333	1.842288712
219699	UNC5B	0.806666667	2.876666667	1.834353512
647310	TEX22	0.883333333	3.143333333	1.831265411
7481	WNT11	0.313333333	1.113333333	1.829115441
10257	ABCC4	6.41	22.77	1.82873803
4137	MAPT	6.653333333	23.48333333	1.819487986
26507	CNNM1	4.393333333	15.48333333	1.817328226
10150	MBNL2	4.536666667	15.96666667	1.815358589
8329	HIST1H2AI	1.69	5.926666667	1.810197672

56912	IFT46	1.893333333	6.616666667	1.805176173
9289	ADGRG1	3.796666667	13.14666667	1.791891805
2104	ESRRG	1.713333333	5.923333333	1.789603417
140733	MACROD2	0.303333333	1.046666667	1.786826109
728392	LOC728392	5.98	20.57333333	1.782558172
3236	HOXD10	0.933333333	3.196666667	1.776103988
54947	LPCAT2	1.336666667	4.54	1.764052562
2342	FNTB	1.796666667	6.083333333	1.759539286
414060	TBC1D3C	0.496666667	1.68	1.758111403
825	CAPN3	0.41	1.386666667	1.757925213
145376	PPP1R36	0.91	3.06	1.749593202
5205	ATP8B1	0.52	1.746666667	1.748020783
644538	SMIM10	0.3	1.006666667	1.746551643
84532	ACSS1	0.9	3.013333333	1.743363365
5139	PDE3A	7.016666667	23.43	1.739499222
494513	PJVK	0.323333333	1.073333333	1.731004036
6840	SVIL	5.743333333	19.05	1.729830797
60529	ALX4	3.996666667	13.25	1.729123202
684	BST2	0.473333333	1.566666667	1.726769827
343702	XKR7	0.58	1.916666667	1.72447465
27344	PCSK1N	44.08	145.15	1.719348443
56147	PCDHA1	0.343333333	1.13	1.718640936
122773	KLHDC1	0.506666667	1.663333333	1.714968492
79132	DHX58	1.19	3.903333333	1.713745096
25858	CATSPERZ	0.31	1.016666667	1.713506621
2042	EPHA3	18.87333333	61.77	1.710557082
1847	DUSP5	1.076666667	3.503333333	1.702156599
283130	SLC25A45	1.966666667	6.396666667	1.701567852
23600	AMACR	7.716666667	25.07666667	1.700295897
4101	MAGEA2	1.533333333	4.96	1.69366876
862	RUNX1T1	2.126666667	6.856666667	1.688913465
7475	WNT6	5.883333333	18.90666667	1.684189349
3675	ITGA3	4.45	14.19333333	1.673336208
408	ARRB1	8.496666667	27.06333333	1.671370671
50801	KCNK4	0.94	2.993333333	1.671020282
221	ALDH3B1	4.243333333	13.50666667	1.670401755
5098	PCDHGC3	5.993333333	18.97	1.662289159
123099	DEGS2	5.193333333	16.43333333	1.661892413
50944	SHANK1	0.593333333	1.873333333	1.658692889
83698	CALN1	0.523333333	1.646666667	1.653746483
7771	ZNF112	0.73	2.286666667	1.647277707
23213	SULF1	3.16	9.886666667	1.645559634
1281	COL3A1	458.9633333	1434.223333	1.643818888

57168	ASPHD2	0.43	1.34	1.639824436
55332	DRAM1	2.47	7.693333333	1.639097776
257019	FRMD3	1.71	5.273333333	1.624718869
8372	HYAL3	0.61	1.88	1.623851514
2307	FOXS1	1.696666667	5.213333333	1.619502951
83595	SOX7	0.456666667	1.393333333	1.609327049
79690	GAL3ST4	1.613333333	4.92	1.608613769
25840	METTL7A	1.62	4.93	1.605593833
4103	MAGEA4	0.386666667	1.176666667	1.605543378
130576	LYPD6B	1.763333333	5.34	1.59853452
8701	DNAH11	1.3	3.936666667	1.598462936
55859	BEX1	1.206666667	3.65	1.596869267
10855	HPSE	0.89	2.69	1.595728932
283212	KLHL35	1.17	3.523333333	1.590432441
5507	PPP1R3C	1.876666667	5.65	1.590078446
84858	ZNF503	18.45333333	55.46666667	1.587739585
2878	GPX3	2.22	6.666666667	1.586405918
857	CAV1	10.66333333	31.93666667	1.58255525
169834	ZNF883	0.436666667	1.306666667	1.581286843
81706	PPP1R14C	3.076666667	9.17	1.575553587
5920	RARRES3	1.3	3.87	1.573821943
91050	CCDC149	0.823333333	2.45	1.573233208
4254	KITLG	0.61	1.813333333	1.571763003
8357	HIST1H3H	7.073333333	21.00666667	1.570385098
128434	VSTM2L	10.90333333	32.38	1.570333727
83468	GLT8D2	3.716666667	10.99333333	1.564547689
85315	PAQR8	1.726666667	5.086666667	1.558730959
57571	CARNS1	1.67	4.9	1.552933647
91608	RASL10B	3.72	10.90666667	1.551835721
11077	HSF2BP	0.566666667	1.653333333	1.544805374
23466	CBX6	24.20666667	70.56	1.543446131
283417	DPY19L2	0.726666667	2.116666667	1.542428457
2296	FOXC1	27.44333333	79.90333333	1.541799964
2257	FGF12	3.406666667	9.896666667	1.538581897
2171	FABP5	6.696666667	19.45	1.538255092
1311	COMP	2.423333333	7.033333333	1.53721573
4884	NPTX1	5.38	15.59333333	1.535251283
58191	CXCL16	5.816666667	16.75666667	1.526470653
100287482	SMKR1	0.92	2.646666667	1.52447074
7164	TPD52L1	4.463333333	12.83333333	1.523702485
3908	LAMA2	15.94	45.67	1.51859516
118881	COMTD1	5.936666667	16.89666667	1.509013647
23670	TMEM2	0.4	1.136666667	1.506737333

202658	TRIM39-RPP21	0.46	1.306666667	1.506185387
8365	HIST1H4H	1.516666667	4.296666667	1.502313813
79983	POF1B	30.07333333	85.19333333	1.502255754
8348	HIST1H2BO	0.536666667	1.516666667	1.498805857
54463	RETREG1	0.553333333	1.563333333	1.498404681
23780	APOL2	3.343333333	9.44	1.497499659
5054	SERPINE1	1.006666667	2.84	1.496304881
148545	NBPF4	3.08	8.676666667	1.494210555
8029	CUBN	2.193333333	6.163333333	1.490585736
817	CAMK2D	12.62333333	35.45	1.489692707
4647	MYO7A	0.626666667	1.75	1.481584761
9498	SLC4A8	0.65	1.813333333	1.480132528
57134	MAN1C1	1.953333333	5.433333333	1.475899395
3755	KCNG1	4.666666667	12.97333333	1.475084883
148932	MOB3C	5.136666667	14.26666667	1.473743935
153572	IRX2	0.996666667	2.76	1.469485283
729262	NUTM2B	0.433333333	1.2	1.469485283
1130	LYST	4.06	11.24	1.469090403
56134	PCDHAC2	0.973333333	2.693333333	1.468386924
994	CDC25B	49.91666667	137.7433333	1.464388993
91947	ARRDC4	2.16	5.96	1.464281018
23362	PSD3	2.79	7.69	1.462718476
57596	BEGAIN	4.143333333	11.40666667	1.461013463
94032	CAMK2N2	1.09	2.996666667	1.45903048
54935	DUSP23	2.056666667	5.633333333	1.453680852
388849	CCDC188	0.6	1.64	1.450661409
57699	CPNE5	0.38	1.036666667	1.447880756
85444	LRRCC1	0.513333333	1.4	1.447458977
3572	IL6ST	16.1	43.86333333	1.445954763
3669	ISG20	0.51	1.386666667	1.443051875
79642	ARSJ	6.183333333	16.77	1.439426003
29118	DDX25	0.473333333	1.283333333	1.438967516
6282	S100A11	102.8066667	278.18	1.436084878
3204	HOXA7	6.35	17.16	1.434221056
9645	MICAL2	0.593333333	1.603333333	1.434159653
3199	HOXA2	1.476666667	3.99	1.434044548
54738	FEV	0.456666667	1.23	1.429444923
51226	COPZ2	0.906666667	2.44	1.428236997
242	ALOX12B	0.43	1.156666667	1.427564597
55638	SYBU	1.903333333	5.116666667	1.426676005
1050	CEBPA	1.673333333	4.493333333	1.425061227
91768	CABLES1	13.52333333	36.23666667	1.421999449
3560	IL2RB	0.863333333	2.31	1.419903254

548596	CKMT1A	8.26	22.09	1.419179732
55924	FAM212B	0.5	1.336666667	1.418639736
83844	USP26	0.846666667	2.263333333	1.418583077
2159	F10	0.603333333	1.61	1.416033492
9659	PDE4DIP	4.476666667	11.94	1.415306033
150094	SIK1	0.393333333	1.046666667	1.4119777
101927322	LOC101927322	0.756666667	2.01	1.409465705
3897	L1CAM	25.83666667	68.20666667	1.400492806
80216	ALPK1	0.996666667	2.63	1.399879816
151195	CCNYL1	37.51	98.92333333	1.399035591
7424	VEGFC	0.53	1.393333333	1.394476177
79148	MMP28	0.446666667	1.173333333	1.393342428
4995	OR3A2	1.363333333	3.58	1.392821245
100526760	ABHD14A-ACY1	0.503333333	1.32	1.390951881
83857	TMTC1	1.02	2.67	1.38827059
8787	RGS9	1.313333333	3.436666667	1.387776798
348013	TMEM255B	0.806666667	2.106666667	1.384917511
9900	SV2A	1.45	3.786666667	1.384875529
9734	HDAC9	38.74	101.0233333	1.382792691
84698	CAPS2	0.776666667	2.023333333	1.381366562
4054	LTBP3	14.59333333	37.99333333	1.380436838
23371	TNS2	3.943333333	10.24666667	1.377667091
10628	TXNIP	94.12666667	243.4833333	1.371147611
653149	NBPF6	0.643333333	1.663333333	1.370438968
51363	CHST15	0.82	2.116666667	1.368098276
6441	SFTPD	0.74	1.903333333	1.362931069
388021	TMEM179	0.443333333	1.14	1.362570079
151636	DTX3L	2.126666667	5.45	1.357662307
2149	F2R	4.34	11.1	1.354792729
10267	RAMP1	8.866666667	22.64333333	1.352622608
92154	MTSS1L	5.133333333	13.07666667	1.349026984
3198	HOXA1	1.216666667	3.09	1.344672875
399979	SNX19	2.186666667	5.54	1.341152662
283576	ZDHHC22	0.613333333	1.553333333	1.340624189
389692	MAFA	1.113333333	2.813333333	1.337394896
8330	HIST1H2AK	0.756666667	1.91	1.335842841
389337	ARHGEF37	1.03	2.596666667	1.33401649
51196	PLCE1	1.49	3.75	1.331578265
7399	USH2A	1.74	4.373333333	1.329646008
124936	CYB5D2	6.68	16.77666667	1.328536089
4643	MYO1E	0.523333333	1.313333333	1.327431071
145946	SPATA8	0.706666667	1.773333333	1.327361981
860	RUNX2	0.903333333	2.266666667	1.327241895

284307	ZIK1	1.753333333	4.386666667	1.323024784
643699	GOLGA8N	1.29	3.223333333	1.321182323
23268	DNMBP	2	4.996666667	1.320965977
51764	GNG13	1.66	4.146666667	1.320768838
198437	LKAAEAR1	1.52	3.793333333	1.319394828
90853	SPOCD1	1.636666667	4.076666667	1.316629474
8407	TAGLN2	34.68	86.3	1.315256661
115811	IQCD	4.203333333	10.45333333	1.314357284
9263	STK17A	3.226666667	8.023333333	1.314157189
139411	PTCHD1	0.72	1.79	1.313890776
2150	F2RL1	5.263333333	13.07333333	1.312578364
190	NR0B1	1.1	2.726666667	1.309634819
5332	PLCB4	1.61	3.986666667	1.308122295
8347	HIST1H2BC	5.846666667	14.42333333	1.302718373
9464	HAND2	8.223333333	20.28333333	1.302499547
116496	FAM129A	4.916666667	12.12333333	1.302033971
387104	SOGA3	0.893333333	2.2	1.300233024
2944	GSTM1	0.47	1.156666667	1.2992405
1806	DPYD	13.56	33.34666667	1.298185378
6284	S100A13	0.98	2.41	1.298179492
1565	CYP2D6	0.563333333	1.383333333	1.29608809
284086	NEK8	1.436666667	3.526666667	1.295579853
94235	GNG8	0.543333333	1.333333333	1.295128036
3234	HOXD8	1.44	3.533333333	1.294961047
222183	SRRM3	4.186666667	10.26666667	1.294093887
84074	QRICH2	0.576666667	1.413333333	1.293292227
7779	SLC30A1	12.9	31.55666667	1.290573756
203111	ERICH5	1.16	2.83	1.286677248
2113	ETS1	8.56	20.82	1.282287367
378108	TRIM74	0.53	1.286666667	1.279574082
24138	IFIT5	1.35	3.273333333	1.277801117
3399	ID3	89.73333333	217.3633333	1.276392686
141	ADPRH	2.21	5.353333333	1.276391117
4603	MYBL1	1.646666667	3.983333333	1.274427671
8336	HIST1H2AM	2.756666667	6.666666667	1.274040765
65983	GRAMD2B	2.01	4.86	1.273760812
56137	PCDHA12	0.656666667	1.58	1.266691429
374882	TMEM205	52.73	126.87	1.266655064
90139	TSPAN18	3.016666667	7.24	1.263034406
146691	TOM1L2	28.43	67.98666667	1.257837738
23705	CADM1	13.9	33.21	1.25653284
387885	CFAP73	0.48	1.146666667	1.256339753
83982	IFI27L2	35.91333333	85.68	1.254438916

6398	SECTM1	3.396666667	8.103333333	1.254395842
55244	SLC47A1	0.81	1.93	1.252607034
144347	RFLNA	2.263333333	5.383333333	1.250050685
11103	KRR1	9.4	22.28333333	1.245232398
4493	MT1E	8.23	19.47	1.242288548
3202	HOXA5	0.696666667	1.646666667	1.2410081
340075	ARSI	1.473333333	3.473333333	1.237237003
2906	GRIN2D	4.35	10.25333333	1.237005697
22801	ITGA11	1.033333333	2.433333333	1.235628248
101059918	GOLGA8R	0.623333333	1.466666667	1.234465254
389813	AJM1	2.833333333	6.66	1.233021837
59271	EVA1C	3.356666667	7.89	1.232996023
3604	TNFRSF9	0.456666667	1.073333333	1.232884795
59284	CACNG7	22.45333333	52.70666667	1.231055817
284613	CYB561D1	2.723333333	6.386666667	1.229689578
51315	KRCC1	3.546666667	8.313333333	1.228963314
4214	MAP3K1	0.9	2.106666667	1.226965151
221178	SPATA13	0.543333333	1.27	1.224919033
216	ALDH1A1	221.01	515.9666667	1.223166217
147685	C19orf18	0.523333333	1.22	1.221079089
124976	SPNS2	0.626666667	1.46	1.220198208
10039	PARP3	2.35	5.473333333	1.219758964
9542	NRG2	2.85	6.636666667	1.219496896
56108	PCDHGA7	1.126666667	2.613333333	1.213830408
2569	GABRR1	0.533333333	1.236666667	1.213347282
1675	CFD	109.9166667	254.59	1.211765594
133383	SETD9	3.13	7.246666667	1.211154877
79873	NUDT18	1.786666667	4.133333333	1.210035215
159371	SLC35G1	1.14	2.636666667	1.20968137
6999	TDO2	0.576666667	1.333333333	1.209227962
389799	CFAP77	0.526666667	1.216666667	1.207971906
9379	NRXN2	0.66	1.523333333	1.206693735
9830	TRIM14	3.9	9	1.206450877
55509	BATF3	2.95	6.796666667	1.204112415
8970	HIST1H2BJ	2.426666667	5.586666667	1.203011794
91010	FMNL3	4.16	9.573333333	1.202437815
1159	CKMT1B	3.153333333	7.24	1.199112014
1825	DSC3	1.75	4.013333333	1.197446064
79680	RTL10	4.936666667	11.32	1.197264818
166614	DCLK2	0.85	1.94	1.190521906
56521	DNAJC12	0.993333333	2.266666667	1.190222416
144100	PLEKHA7	0.916666667	2.09	1.189033824
3973	LHCGR	1	2.28	1.189033824

85369	STRIP1	11.91666667	27.16	1.188502738
27092	CACNG4	0.553333333	1.26	1.187202993
64478	CSMD1	0.546666667	1.243333333	1.185479816
11230	PRAF2	15.78333333	35.88333333	1.184911989
9540	TP53I3	19.21	43.65333333	1.184234302
84631	SLITRK2	3.476666667	7.9	1.184147901
84460	ZMAT1	1.79	4.066666667	1.183887154
10929	SRSF8	0.77	1.746666667	1.18167396
5971	RELB	3.916666667	8.88	1.180933326
54972	TMEM132A	35.26666667	79.90666667	1.180010652
158866	ZDHHC15	2.023333333	4.58	1.178613583
60680	CELF5	1.843333333	4.17	1.177730404
53840	TRIM34	0.796666667	1.8	1.175948789
55106	SLFN12	0.476666667	1.076666667	1.175519018
9788	MTSS1	0.783333333	1.766666667	1.173331603
5045	FURIN	17.82	40.17333333	1.172740835
53342	IL17D	1.376666667	3.103333333	1.172639386
7454	WAS	0.763333333	1.72	1.172023467
6604	SMARCD3	28.28333333	63.72333333	1.171869576
8819	SAP30	11.62333333	26.18666667	1.171808566
54997	TESC	0.476666667	1.073333333	1.171045541
100129654	TCF24	0.57	1.283333333	1.170862121
55783	CMTR2	1.643333333	3.693333333	1.16829833
10148	EBI3	11.14666667	25.04333333	1.167814255
2049	EPHB3	2.503333333	5.623333333	1.167575161
56135	PCDHAC1	0.516666667	1.16	1.166819091
7781	SLC30A3	0.493333333	1.106666667	1.165586066
402682	UFSP1	1.043333333	2.336666667	1.163251787
387496	RASL11A	3.16	7.06	1.159743625
954	ENTPD2	19.42666667	43.39	1.159324209
9241	NOG	7.833333333	17.48666667	1.158557051
64073	C19orf33	2.11	4.71	1.158484061
23382	AHCYL2	21.17666667	47.20666667	1.156515097
55715	DOK4	26.13666667	58.23333333	1.155770043
2537	IFI6	59.08333333	131.6	1.155336362
7168	TPM1	110.8	246.7833333	1.155287083
348487	FAM131C	2.586666667	5.756666667	1.154139525
7552	ZNF711	11.27	25.06	1.152898899
10677	AVIL	0.59	1.31	1.150779952
3958	LGALS3	5.673333333	12.59333333	1.150391665
1602	DACH1	4.03	8.913333333	1.145185221
3215	HOXB5	3.216666667	7.11	1.144283118
6595	SMARCA2	0.89	1.966666667	1.143875213

4482	MSRA	1.15	2.54	1.143194636
25789	TMEM59L	34.27666667	75.57	1.140586806
112849	L3HYPDH	2.453333333	5.406666667	1.139996148
84159	ARID5B	2.48	5.463333333	1.139441328
5224	PGAM2	2.72	5.986666667	1.138146293
56660	KCNK12	0.673333333	1.48	1.136204384
6277	S100A6	80.03	175.7433333	1.134857149
85446	ZFHX2	2.54	5.573333333	1.133711945
50509	COL5A3	0.693333333	1.52	1.132450296
5493	PPL	11.91666667	26.10333333	1.131253306
389432	SAMD5	0.683333333	1.496666667	1.131091535
57143	ADCK1	3.156666667	6.893333333	1.126799855
55796	MBNL3	0.856666667	1.87	1.126232411
51316	PLAC8	2.98	6.496666667	1.124387354
51375	SNX7	30.57666667	66.52666667	1.12150161
6890	TAP1	8.83	19.20666667	1.121121817
81839	VANGL1	2.29	4.973333333	1.118865531
10892	MALT1	2.546666667	5.53	1.118669343
23641	LDOC1	7.02	15.22	1.116425423
29899	GPSM2	15.38	33.33	1.115765814
126119	JOSD2	13.98666667	30.30666667	1.115583006
84700	MYO18B	1.303333333	2.823333333	1.115193362
7035	TFPI	18.53	40.1	1.113739355
5731	PTGER1	9.023333333	19.50666667	1.112234882
5630	PRPH	14.97333333	32.34666667	1.111221623
4920	ROR2	5.043333333	10.89333333	1.110995996
339145	FAM92B	0.606666667	1.31	1.110590862
9310	ZNF235	0.666666667	1.436666667	1.107687869
8549	LGR5	0.583333333	1.256666667	1.107209601
1947	EFNB1	4.626666667	9.966666667	1.107137917
151963	MB21D2	3.866666667	8.323333333	1.106071016
8814	CDKL1	1.593333333	3.42	1.101948208
50632	CALY	32.94333333	70.57333333	1.099136613
219348	PLAC9	0.756666667	1.62	1.098264016
9895	TECPR2	4.51	9.643333333	1.096404484
89796	NAV1	0.946666667	2.023333333	1.095805587
64711	HS3ST6	3.8	8.113333333	1.094295344
84696	ABHD1	1.396666667	2.973333333	1.090093466
64065	PERP	0.623333333	1.326666667	1.089730161
494143	CHAC2	1.716666667	3.65	1.088286532
100533496	TVP23C-CDRT4	0.83	1.76	1.084392187
153769	SH3RF2	5.473333333	11.60333333	1.084047687
3207	HOXA11	0.59	1.25	1.083141235

170689	ADAMTS15	1.74	3.683333333	1.081924658
7957	EPM2A	0.6	1.27	1.081794091
3090	HIC1	0.516666667	1.093333333	1.081427599
91754	NEK9	10.46	22.10333333	1.079381103
55970	GNG12	2.86	6.04	1.078533403
10553	HTATIP2	3.306666667	6.973333333	1.076470826
8871	SYNJ2	0.683333333	1.44	1.075407403
3620	IDO1	0.976666667	2.056666667	1.074369825
5578	PRKCA	0.853333333	1.796666667	1.074141463
11142	PKIG	67.56	142.1533333	1.073206696
64116	SLC39A8	2.8	5.886666667	1.07202411
79815	NIPAL2	1.086666667	2.28	1.069124361
5129	CDK18	12.29	25.78333333	1.068953875
27239	GPR162	14.95333333	31.36666667	1.068765103
10536	P3H3	4.47	9.36	1.066233698
7766	ZNF223	1.2	2.51	1.064652958
9725	TMEM63A	4.263333333	8.913333333	1.063983201
65268	WNK2	1.903333333	3.973333333	1.061821585
54505	DHX29	13.63333333	28.43666667	1.06061402
1278	COL1A2	1125.693333	2347.783333	1.060485419
200958	MUC20	0.973333333	2.03	1.060473859
3693	ITGB5	3.14	6.546666667	1.059995965
26094	DCAF4	2.77	5.77	1.058685343
3856	KRT8	0.853333333	1.776666667	1.057991723
25876	SPEF1	0.54	1.123333333	1.056754778
651746	ANKRD33B	17.58666667	36.57333333	1.056310058
135	ADORA2A	1.02	2.12	1.055495113
10763	NES	17.55333333	36.45333333	1.054305722
7204	TRIO	8.586666667	17.77666667	1.049814735
6583	SLC22A4	0.586666667	1.213333333	1.048363022
8321	FZD1	1.41	2.913333333	1.046975616
3206	HOXA10	2.733333333	5.646666667	1.04673806
8503	PIK3R3	4.9	10.12	1.046355636
80328	ULBP2	0.93	1.92	1.04580369
1465	CSRP1	42.26333333	87.22	1.045252432
79822	ARHGAP28	0.653333333	1.346666667	1.043501639
57727	NCOA5	13.02666667	26.84333333	1.043095866
6352	CCL5	2.26	4.656666667	1.042974842
4784	NFIX	18.19333333	37.48666667	1.042967652
115708	TRMT61A	2.996666667	6.173333333	1.042691078
51306	FAM13B	2.75	5.66	1.041370434
51065	RPS27L	20.74666667	42.55666667	1.036505594
2487	FRZB	49.33	100.93	1.032817866

389524	GTF2IRD2B	2.15	4.393333333	1.030979305
21	ABCA3	12.16333333	24.85333333	1.030900709
647024	C6orf132	2.026666667	4.14	1.030521945
467	ATF3	4.526666667	9.246666667	1.030484308
23732	FRRS1L	1.873333333	3.826666667	1.030480606
2736	GLI2	2.346666667	4.793333333	1.030416342
55243	KIRREL1	7.846666667	16.01	1.028821488
1519	CTSO	6.993333333	14.23666667	1.02555922
284297	SSC5D	1.126666667	2.293333333	1.025385318
649	BMP1	4.506666667	9.166666667	1.024336467
10379	IRF9	16.75333333	34.05666667	1.023489066
1466	CSRP2	5.343333333	10.86	1.023212178
80017	DGLUCY	3.94	8.003333333	1.022405368
4929	NR4A2	7.07	14.35	1.021268617
57205	ATP10D	2.166666667	4.396666667	1.020932941
245806	VGLL2	1.076666667	2.183333333	1.019960742
51700	CYB5R2	1.013333333	2.053333333	1.018859027
56144	PCDHA4	2.146666667	4.346666667	1.017811276
348327	ZNF530	0.683333333	1.383333333	1.017487427
6281	S100A10	503.78	1019.103333	1.016434589
6320	CLEC11A	23.54	47.55	1.014331021
7263	TST	14.08666667	28.44	1.013591198
57562	CEP126	1.066666667	2.153333333	1.01346226
7975	MAFK	7.223333333	14.56333333	1.011603952
26268	FBXO9	13.18666667	26.55333333	1.009813054
114804	RNF157	1.046666667	2.106666667	1.009159999
81831	NETO2	18.64333333	37.48666667	1.007717717
57594	HOMEZ	4.526666667	9.096666667	1.006888915
25805	BAMBI	1.59	3.193333333	1.00603639
55665	URGCP	8.156666667	16.37	1.005002721
79683	ZDHHC14	2.436666667	4.886666667	1.003941792
22821	RASA3	4.176666667	8.373333333	1.00345005
2812	GP1BB	10.85333333	21.75333333	1.003098288
7041	TGFB1I1	22.73	45.51333333	1.001691567
55930	MYO5C	1.673333333	3.35	1.001436232
92610	TIFA	1.48	0.74	-1
2827	GPR3	1.083333333	0.54	-1.004445905
347735	SERINC2	12.90666667	6.426666667	-1.005973901
146206	CARMIL2	1.346666667	0.67	-1.007159792
57688	ZSWIM6	3.613333333	1.796666667	-1.008007579
5783	PTPN13	5.623333333	2.79	-1.011160446
6319	SCD	261.5733333	129.77	-1.011258567
10000	AKT3	4.22	2.093333333	-1.011440941

1373	CPS1	77.93333333	38.65666667	-1.011523284
55803	ADAP2	27.26666667	13.52333333	-1.011687541
100631383	FAM47E-STBD1	1.54	0.763333333	-1.012545253
6303	SAT1	91.66333333	45.43	-1.012699445
28984	RGCC	9.706666667	4.81	-1.012939056
348093	RBPMS2	47.40666667	23.49	-1.013043242
5077	PAX3	1.07	0.53	-1.013546532
5230	PGK1	587.1366667	290.5733333	-1.014794047
2695	GIP	7.436666667	3.676666667	-1.016257723
6228	RPS23	117.7533333	58.21666667	-1.016263758
7169	TPM2	113.53	56.10666667	-1.016829467
347688	TUBB8	4.283333333	2.116666667	-1.016939862
900	CCNG1	52.5	25.89	-1.019922458
9832	JAKMIP2	2.313333333	1.14	-1.020939338
127602	DNAH14	8.833333333	4.35	-1.021942553
121643	FOXN4	2.973333333	1.463333333	-1.02282277
730098	LOC730098	3.78	1.86	-1.023083613
8543	LMO4	3.313333333	1.63	-1.023411387
25816	TNFAIP8	3.253333333	1.6	-1.023846742
27165	GLS2	4.663333333	2.293333333	-1.023915492
6496	SIX3	196.3366667	96.51333333	-1.024529457
10793	ZNF273	3.583333333	1.76	-1.025726825
9306	SOCS6	11.70333333	5.746666667	-1.026122222
220134	SKA1	7.34	3.6	-1.027783157
23150	FRMD4B	1.726666667	0.846666667	-1.028123601
58526	MID1IP1	15.84333333	7.756666667	-1.030367191
9156	EXO1	6.526666667	3.193333333	-1.031283204
51092	SIDT2	58.36	28.53666667	-1.032163058
158038	LINGO2	1.03	0.503333333	-1.033058289
284273	ZADH2	1.27	0.62	-1.034488376
2596	GAP43	177.9533333	86.77	-1.036230723
3655	ITGA6	3.983333333	1.94	-1.03791956
10381	TUBB3	176.7233333	86.04	-1.038413106
9846	GAB2	23.30333333	11.34333333	-1.038691684
25914	RTTN	6.153333333	2.993333333	-1.039615203
57393	CLTRN	1.213333333	0.59	-1.04018909
6917	TCEA1	141.57	68.81333333	-1.040755542
100861412	FSBP	1.983333333	0.963333333	-1.041820176
55876	GSDMB	1.313333333	0.636666667	-1.044622991
3590	IL11RA	19.24	9.32	-1.045706939
7163	TPD52	50.31333333	24.37	-1.045834522
100526693	ARPC4-TTLL3	3.143333333	1.52	-1.048223947
54532	USP53	27.95	13.49666667	-1.050245141

5837	PYGM	22.63333333	10.90333333	-1.053679815
317649	EIF4E3	85.89333333	41.36	-1.054309974
7644	ZNF91	8.716666667	4.196666667	-1.054532663
55753	OGDHL	9.706666667	4.67	-1.0555534
54765	TRIM44	1.22	0.586666667	-1.05626822
93349	SP140L	2.786666667	1.34	-1.056307441
10461	MERTK	14.56333333	6.986666667	-1.059664389
83543	AIF1L	6.22	2.983333333	-1.059989399
338761	C1QL4	42.27	20.27333333	-1.060050797
5570	PKIB	13.67666667	6.54	-1.064354113
54549	SDK2	2.363333333	1.13	-1.064500354
9543	IGDCC3	1.903333333	0.91	-1.064589794
10675	CSPG5	17.01	8.12	-1.066831508
115362	GBP5	8.186666667	3.903333333	-1.068569485
27443	CECR2	8.913333333	4.243333333	-1.070767046
221120	ALKBH3	4.7	2.233333333	-1.073462162
283659	PRTG	10.12333333	4.806666667	-1.074575744
27255	CNTN6	12.02333333	5.7	-1.076803098
10087	COL4A3BP	13.42	6.356666667	-1.078042329
6902	TBCA	303.06	143.3933333	-1.079625496
7101	NR2E1	66.07	31.25333333	-1.079985276
116039	OSR2	21.19	10.02	-1.080501079
6658	SOX3	246.5666667	116.44	-1.08239103
55194	EVA1B	30.35666667	14.32	-1.083981891
133418	EMB	14.91	7.033333333	-1.083999759
22902	RUFY3	20.04	9.45	-1.084496274
23338	JADE2	33.18	15.63	-1.085996108
85442	KNDC1	2.69	1.266666667	-1.086569255
8092	ALX1	10.61333333	4.996666667	-1.086839953
126969	SLC44A3	1.12	0.526666667	-1.088536675
256126	SYCE2	2.63	1.236666667	-1.088606113
3398	ID2	226.4333333	106.3	-1.090944757
339456	TMEM52	14.79666667	6.943333333	-1.091571869
8626	TP63	15.15	7.096666667	-1.094104345
56929	FEM1C	5.92	2.77	-1.0957112
6935	ZEB1	1.81	0.846666667	-1.096123701
3685	ITGAV	195.49	91.38	-1.097144463
4062	LY6H	6.583333333	3.073333333	-1.099013998
8724	SNX3	331.02	154.3366667	-1.100837534
5864	RAB3A	14.21333333	6.62	-1.102341815
3038	HAS3	11.59666667	5.396666667	-1.103569693
56853	CELF4	1.083333333	0.503333333	-1.105891169
7448	VTN	1.033333333	0.48	-1.106199404

51027	BOLA1	3.72	1.726666667	-1.107313024
144195	SLC2A14	2.506666667	1.163333333	-1.107505625
134359	POC5	4.686666667	2.17	-1.110867146
353322	ANKRD37	2.426666667	1.123333333	-1.111189859
8702	B4GALT4	13.02	6.026666667	-1.111297271
64105	CENPK	7.266666667	3.363333333	-1.111401961
56605	ERO1B	1.42	0.656666667	-1.112657801
134548	SOWAHA	1.04	0.48	-1.115477217
93	ACVR2B	3.693333333	1.703333333	-1.116562685
4605	MYBL2	99.01333333	45.64666667	-1.117113303
1936	EEF1D	150.63	69.32666667	-1.119526831
94031	HTRA3	5.57	2.563333333	-1.11965623
9497	SLC4A7	1.923333333	0.883333333	-1.122578959
56992	KIF15	5.02	2.296666667	-1.128145882
1749	DLX5	1.38	0.63	-1.131244533
8497	PPFIA4	2.056666667	0.936666667	-1.134700359
643246	MAP1LC3B2	5.893333333	2.68	-1.136850868
131405	TRIM71	2.676666667	1.216666667	-1.137503524
7015	TERT	2.606666667	1.18	-1.143419247
100996741	LOC100996741	3.81	1.723333333	-1.144589218
2264	FGFR4	9.793333333	4.423333333	-1.146666025
2245	FGD1	109.0233333	49.17333333	-1.148688876
79412	KREMEN2	66.28666667	29.79666667	-1.15356776
284254	DYNAP	1.3	0.583333333	-1.156119202
8490	RGS5	40.04	17.89333333	-1.162019803
23550	PSD4	2.243333333	1	-1.165644004
3491	CYR61	228.3833333	101.7766667	-1.166050525
29057	FAM156A	1.22	0.543333333	-1.166971684
2966	GTF2H2	27.41	12.2	-1.16782118
83463	MXD3	7.476666667	3.326666667	-1.1683179
29944	PNMA3	1.126666667	0.5	-1.172060746
55435	AP1AR	4.16	1.84	-1.176877762
100533106	ZHX1-C8orf76	2.536666667	1.12	-1.179435221
5757	PTMA	1898.693333	837.1133333	-1.181512046
8174	MADCAM1	1.826666667	0.803333333	-1.185142747
3050	HBZ	1.656666667	0.726666667	-1.188917717
55314	TMEM144	9.493333333	4.163333333	-1.189175669
3613	IMPA2	31.14	13.64333333	-1.190572778
90355	C5orf30	4.05	1.763333333	-1.199616686
10683	DLL3	60.08666667	26.14	-1.200785749
8310	ACOX3	2.713333333	1.18	-1.201279434
91057	CCDC34	34.05333333	14.80666667	-1.201549132
2515	ADAM2	1.226666667	0.533333333	-1.201633861

55636	CHD7	10.12333333	4.396666667	-1.203202344
6876	TAGLN	3.97	1.723333333	-1.203937228
51441	YTHDF2	77.95333333	33.83	-1.204307538
730394	GTF2H2C_2	26.44666667	11.47666667	-1.204382217
64946	CENPH	23.01666667	9.983333333	-1.205085411
26227	PHGDH	71.70666667	31.1	-1.205192674
26355	FAM162A	81.28333333	35.2	-1.207384138
6662	SOX9	1.433333333	0.62	-1.209034038
2118	ETV4	44.81666667	19.36333333	-1.21070802
79956	ERMP1	5.096666667	2.2	-1.212050477
1122	CHML	4.616666667	1.986666667	-1.21650174
160622	GRASP	5.043333333	2.17	-1.216682539
27074	LAMP3	2.676666667	1.15	-1.218803626
23025	UNC13A	2.693333333	1.156666667	-1.21941963
7980	TFPI2	34.73666667	14.91333333	-1.219856564
9060	PAPSS2	8.8	3.766666667	-1.224215157
604	BCL6	2.453333333	1.05	-1.224353938
7634	ZNF80	11.82	5.056666667	-1.224971451
3164	NR4A1	32.93333333	14.08	-1.225901207
6606	SMN1	27.68666667	11.82	-1.227961336
3589	IL11	1.383333333	0.59	-1.229361976
25854	FAM149A	10.63666667	4.5	-1.241049201
29097	CNIH4	76.00666667	32.11	-1.2431033
7855	FZD5	31.61333333	13.33333333	-1.245495663
8905	AP1S2	14.34666667	6.05	-1.24570853
642819	ZNF487	1.906666667	0.803333333	-1.246982001
131616	TMEM42	11.38333333	4.793333333	-1.247821903
6405	SEMA3F	6.25	2.626666667	-1.250623061
56099	PCDHGB7	1.42	0.596666667	-1.250893843
5214	PFKP	25.98666667	10.91	-1.252120488
79924	ADM2	2.396666667	1.003333333	-1.256228284
171024	SYNPO2	11.07	4.633333333	-1.25653284
23593	HEBP2	21.57666667	9.016666667	-1.25880591
3600	IL15	12.63333333	5.263333333	-1.263186677
130888	FBXO36	5.79	2.41	-1.264530202
10434	LYPLA1	104.3433333	43.42	-1.264906796
5144	PDE4D	5.136666667	2.126666667	-1.272238533
1184	CLCN5	7.666666667	3.173333333	-1.272600383
6340	SCNN1G	18.64	7.7	-1.275471509
7098	TLR3	4.54	1.873333333	-1.277084668
10507	SEMA4D	6.883333333	2.833333333	-1.280607035
8436	CAVIN2	1.62	0.666666667	-1.280956314
4615	MYD88	1.216666667	0.5	-1.282933963

6490	PMEL	3.393333333	1.393333333	-1.284162714
440836	ODF3B	14.49	5.933333333	-1.288142854
9456	HOMER1	7.59	3.09	-1.296493048
27134	TJP3	10.35	4.213333333	-1.296596805
80312	TET1	13.75	5.596666667	-1.296791889
51361	HOOK1	1.36	0.553333333	-1.297385911
388581	C1QTNF12	2.163333333	0.88	-1.297680549
90649	ZNF486	3.83	1.553333333	-1.301976938
93099	DMKN	5.43	2.183333333	-1.314419792
8565	YARS	66.77666667	26.81666667	-1.316214159
7137	TNNI3	1.216666667	0.486666667	-1.321928095
11075	STMN2	20.01	7.97	-1.328069538
5454	POU3F2	42.17666667	16.78333333	-1.329415802
84900	RNFT2	1.78	0.706666667	-1.332775477
7179	TPTE	13.79	5.46	-1.336649601
338645	LUZP2	11.30666667	4.443333333	-1.347459389
6515	SLC2A3	101.7533333	39.92	-1.349892431
23127	COLGALT2	102.4766667	40.1	-1.353621312
388730	TMEM81	2.136666667	0.833333333	-1.358396262
1028	CDKN1C	9.796666667	3.82	-1.358718315
1382	CRABP2	1559.673333	607.9933333	-1.359116485
80303	EFHD1	12.63333333	4.856666667	-1.379196971
165215	FAM171B	74.65666667	28.59666667	-1.384424104
4661	MYT1	1.95	0.746666667	-1.384937892
59353	TMEM35A	52.61333333	20.09	-1.388950891
728340	GTF2H2C	11.29666667	4.306666667	-1.391253567
222643	UNC5CL	1.146666667	0.436666667	-1.392841753
4638	MYLK	1.326666667	0.503333333	-1.398219881
4900	NRGN	14.19333333	5.373333333	-1.401324206
22843	PPM1E	2.52	0.953333333	-1.402371087
11166	SOX21	3.506666667	1.323333333	-1.405923792
282809	POC1B	19.57	7.38	-1.406951035
81031	SLC2A10	8.036666667	3.03	-1.407279451
83604	TMEM47	43.02333333	16.2	-1.409125492
5309	PITX3	2.32	0.873333333	-1.409520494
5210	PFKFB4	8.426666667	3.17	-1.410479217
3781	KCNN2	15.64333333	5.856666667	-1.417396269
120071	LARGE2	8.063333333	2.99	-1.431230879
117166	WFIKKN1	5.826666667	2.156666667	-1.433867568
6424	SFRP4	384.5333333	140.9266667	-1.448164034
154790	CLEC2L	1.183333333	0.433333333	-1.449307401
8313	AXIN2	2.236666667	0.816666667	-1.453531017
6445	SGCG	3.19	1.163333333	-1.455291888

8693	GALNT4	9.776666667	3.563333333	-1.456115218
5443	POMC	1.25	0.453333333	-1.463283944
57576	KIF17	5.63	2.036666667	-1.466925043
57685	CACHD1	1.603333333	0.58	-1.466949588
7378	UPP1	204.8033333	73.92333333	-1.47013748
53833	IL20RB	1.503333333	0.533333333	-1.495055528
287	ANK2	47.04	16.65666667	-1.497788343
9473	THEMIS2	5.97	2.113333333	-1.498210592
55093	WDYHV1	7.65	2.703333333	-1.500720334
401	PHOX2A	1.17	0.413333333	-1.50113091
51778	MYOZ2	6.12	2.16	-1.502500341
4255	MGMT	13.5	4.756666667	-1.504936573
140738	TMEM37	1.913333333	0.673333333	-1.506695444
1021	CDK6	4.38	1.54	-1.508000519
3109	HLA-DMB	3.916666667	1.376666667	-1.50844707
8987	STBD1	10.30333333	3.606666667	-1.514373155
83881	MIXL1	3.17	1.106666667	-1.5182621
4885	NPTX2	4.576666667	1.593333333	-1.522249102
50515	CHST11	12.96666667	4.496666667	-1.527879807
79152	FA2H	55.49666667	19.18	-1.5327984
8749	ADAM18	15.89666667	5.493333333	-1.53297054
664	BNIP3	340.3633333	117.45	-1.535028913
222223	KIAA1324L	3.72	1.283333333	-1.535406676
7148	TNXB	1.13	0.386666667	-1.547160468
5106	PCK2	34.13333333	11.63666667	-1.552503453
5163	PDK1	13.45666667	4.576666667	-1.555951961
54020	SLC37A1	8.556666667	2.91	-1.556029737
91522	COL23A1	2.6	0.88	-1.562936194
23090	ZNF423	1.273333333	0.43	-1.566201573
57369	GJD2	1.93	0.65	-1.570089224
284422	SMIM24	3.383333333	1.136666667	-1.573636083
7697	ZNF138	7.76	2.596666667	-1.579395825
221303	FAM162B	2.683333333	0.896666667	-1.58138261
51309	ARMCX1	1.053333333	0.35	-1.589535231
2119	ETV5	15.34666667	5.093333333	-1.59124329
116844	LRG1	3.416666667	1.133333333	-1.592017258
169026	SLC30A8	23.21666667	7.693333333	-1.593480129
335	APOA1	3.866666667	1.276666667	-1.598708508
402778	IFITM10	1.356666667	0.446666667	-1.602795794
29968	PSAT1	51.30666667	16.82	-1.608968593
7373	COL14A1	1.906666667	0.623333333	-1.612976877
257407	C2orf72	3.04	0.99	-1.618570893
8537	BCAS1	1.94	0.63	-1.622632919

8633	UNC5C	1.41	0.456666667	-1.62648177
103344718	HOTS	2.86	0.916666667	-1.641546029
4664	NAB1	2.896666667	0.926666667	-1.644271294
9262	STK17B	7.723333333	2.47	-1.644712597
9743	ARHGAP32	2.16	0.69	-1.646363045
1848	DUSP6	113.9133333	36.30666667	-1.649630235
146429	SLC22A31	2.703333333	0.846666667	-1.674873417
106865373	WRB-SH3BGR	2.486666667	0.773333333	-1.685050825
440585	FAM183A	1.533333333	0.476666667	-1.685618714
23414	ZFPM2	12.04333333	3.74	-1.687124579
10409	BASP1	7.936666667	2.456666667	-1.691831096
83643	CCDC3	1.453333333	0.446666667	-1.702095134
7291	TWIST1	3.986666667	1.216666667	-1.71224902
642475	MROH6	1.356666667	0.413333333	-1.714688674
27345	KCNMB4	2.433333333	0.74	-1.717336787
84898	PLXDC2	2.613333333	0.793333333	-1.719892081
147798	TMC4	2.073333333	0.626666667	-1.726181918
149483	CCDC17	1.116666667	0.336666667	-1.729805803
4131	MAP1B	15.00333333	4.52	-1.730888386
168620	BHLHA15	1.716666667	0.506666667	-1.760501109
3816	KLK1	2.04	0.596666667	-1.773572065
586	BCAT1	16.04666667	4.68	-1.777693206
10253	SPRY2	61.92	18	-1.782408565
80709	AKNA	7.18	2.076666667	-1.789714182
4237	MFAP2	14.48333333	4.16	-1.799738243
8447	DOC2B	2.443333333	0.7	-1.80342387
1649	DDIT3	42.41	12.12	-1.807014784
3352	HTR1D	2.03	0.576666667	-1.81567019
10826	FAXDC2	10.7	3.013333333	-1.82817862
29969	MDFIC	27	7.55	-1.838410858
84101	USP44	15.11	4.2	-1.847042427
55630	SLC39A4	25.67	7.1	-1.854192366
6866	TAC3	1.703333333	0.47	-1.857628129
5797	PTPRM	5.1	1.406666667	-1.858216749
22837	COBLL1	3.76	1.03	-1.868088324
710	SERPING1	109.19	29.79333333	-1.873779286
256691	MAMDC2	12.09666667	3.29	-1.878450067
1295	COL8A1	7.98	2.166666667	-1.880911529
50863	NTM	8.656666667	2.35	-1.881150851
221914	GPC2	1.903333333	0.51	-1.899959093
2047	EPHB1	1.21	0.32	-1.918863237
2911	GRM1	4.636666667	1.213333333	-1.934112064
126014	OSCAR	5.753333333	1.493333333	-1.945861827

27019	DNAI1	8.62	2.23	-1.950644159
79094	CHAC1	12.44666667	3.216666667	-1.95212108
138255	C9orf135	24.45333333	6.306666667	-1.95508155
5333	PLCD1	40.20666667	10.26333333	-1.969935368
64220	STRA6	1.556666667	0.396666667	-1.972460976
9886	RHOBTB1	2.33	0.59	-1.981543095
2770	GNAI1	3	0.743333333	-2.012881291
93986	FOXP2	13.72	3.373333333	-2.024033692
4118	MAL	6.063333333	1.463333333	-2.050852698
345895	RSPH4A	2.273333333	0.546666667	-2.056075924
84889	SLC7A3	61.87	14.84	-2.059748942
84634	KISS1R	13.40666667	3.166666667	-2.081913663
55843	ARHGAP15	62.28666667	14.47	-2.105858445
3270	HRC	6.396666667	1.466666667	-2.124779283
2348	FOLR1	3.176666667	0.726666667	-2.128148079
10194	TSHZ1	2.263333333	0.516666667	-2.131143359
79999	LOC79999	17.21666667	3.923333333	-2.133654029
91351	DDX60L	1.306666667	0.296666667	-2.138976413
10003	NAALAD2	1.293333333	0.293333333	-2.140481224
285141	ERICH2	3.34	0.753333333	-2.148487831
6691	SPINK2	3.94	0.876666667	-2.168095331
154141	MBOAT1	28.46666667	6.283333333	-2.179671546
83592	AKR1E2	1.513333333	0.333333333	-2.182692298
53826	FXYD6	1.126666667	0.246666667	-2.191426071
56979	PRDM9	7.713333333	1.67	-2.207506356
8076	MFAP5	11.60333333	2.48	-2.226127288
9427	ECEL1	6.483333333	1.38	-2.232067482
645121	CCNI2	1.146666667	0.243333333	-2.236440196
440	ASNS	109.24	23.12333333	-2.24007993
55760	DHX32	6.06	1.253333333	-2.273547633
100506243	KRBOX1	1.48	0.303333333	-2.286621226
1004	CDH6	233.33	47.8	-2.287289288
4038	LRP4	19.21666667	3.91	-2.297117595
6857	SYT1	17.49666667	3.54	-2.305258831
2122	MECOM	1.04	0.203333333	-2.354664881
7837	PXDN	1.063333333	0.206666667	-2.363216303
51050	PI15	5.723333333	1.103333333	-2.374986917
169166	SNX31	1.983333333	0.38	-2.383855844
60675	PROK2	1.75	0.33	-2.406816992
79727	LIN28A	1.22	0.226666667	-2.428236997
131540	ZDHHC19	4.573333333	0.836666667	-2.450521212
7857	SCG2	2.736666667	0.5	-2.452419721
4857	NOVA1	10.37	1.846666667	-2.489420513

144568	A2ML1	1.316666667	0.233333333	-2.496425826
136288	C7orf57	113.3266667	20.01333333	-2.501453997
10251	SPRY3	9.166666667	1.606666667	-2.512326567
4692	NDN	4.903333333	0.853333333	-2.522581531
388531	RGS9BP	1.42	0.246666667	-2.525256255
3485	IGFBP2	36.95	6.383333333	-2.533192473
391059	FRRS1	1.456666667	0.25	-2.542670779
487	ATP2A1	2.846666667	0.486666667	-2.548267701
7020	TFAP2A	8.72	1.47	-2.56851198
131149	OTOL1	1.746666667	0.29	-2.590479506
799	CALCR	9.403333333	1.54	-2.610241909
1404	HAPLN1	1.123333333	0.18	-2.641717279
5325	PLAGL1	13.02	2.083333333	-2.643763854
83482	SCRT1	3.336666667	0.516666667	-2.691101854
56884	FSTL5	1.166666667	0.18	-2.69632361
23022	PALLD	9.79	1.453333333	-2.751943226
9315	NREP	44.07333333	6.423333333	-2.778511943
22865	SLITRK3	2.226666667	0.31	-2.844545481
1299	COL9A3	3.9	0.526666667	-2.888512066
1482	NKX2-5	1.063333333	0.143333333	-2.891147859
7425	VGF	51.29666667	6.86	-2.902584599
27123	DKK2	1.256666667	0.166666667	-2.914564523
402665	IGLON5	1.95	0.256666667	-2.925506274
58494	JAM2	4.663333333	0.613333333	-2.926618291
92162	TMEM88	144.9	18.76666667	-2.948813268
2566	GABRG2	4.81	0.616666667	-2.963474124
79729	SH3D21	6.636666667	0.843333333	-2.976283931
2888	GRB14	3.333333333	0.42	-2.988504361
79632	FAM184A	1.176666667	0.146666667	-3.004092755
79827	CLMP	1.186666667	0.14	-3.083416008
80712	ESX1	1.003333333	0.113333333	-3.146156836
4772	NFATC1	1.93	0.213333333	-3.177419538
10468	FST	50.97666667	5.553333333	-3.19841114
7078	TIMP3	5.326666667	0.58	-3.199108197
167410	LIX1	9.07	0.983333333	-3.205350097
152831	KLB	2.02	0.216666667	-3.22080617
79772	MCTP1	4.516666667	0.48	-3.234152135
91851	CHRDL1	3.776666667	0.393333333	-3.263289096
339479	BRINP3	8.63	0.896666667	-3.266716887
25928	SOSTDC1	5.52	0.556666667	-3.309782665
220382	FAM181B	4.3	0.413333333	-3.37895904
10417	SPON2	1.17	0.11	-3.410933101
55103	RALGPS2	1.193333333	0.11	-3.439421658

3060	HCRT	1.406666667	0.123333333	-3.511645823
794	CALB2	3.386666667	0.283333333	-3.579293751
148113	CILP2	1.573333333	0.13	-3.59724083
407977	TNFSF12-TNFSF13	1.3	0.103333333	-3.653134003
10840	ALDH1L1	17.19333333	1.316666667	-3.706887215
3912	LAMB1	13.04333333	0.983333333	-3.729488251
100271846	ERVV-2	1.023333333	0.076666667	-3.738532889
83690	CRISPLD1	43.89	3.223333333	-3.767266976
2045	EPHA7	1.16	0.08	-3.857980995
2173	FABP7	4.023333333	0.256666667	-3.97042342
56776	FMN2	1.373333333	0.086666667	-3.986060809
8433	UTF1	2.153333333	0.133333333	-4.01346226
8784	TNFRSF18	1.693333333	0.093333333	-4.181329765
22999	RIMS1	3.873333333	0.213333333	-4.182394353
653220	XAGE1A	1.446666667	0.076666667	-4.237989276
653067	XAGE1B	1.446666667	0.076666667	-4.237989276
7001	PRDX2	8.78	0.443333333	-4.307757195
760	CA2	5.656666667	0.28	-4.336453427
5156	PDGFRA	13.24333333	0.623333333	-4.409118618
64849	SLC13A3	4.366666667	0.2	-4.448460501
441581	FRG2B	2.94	0.133333333	-4.462706751
448831	FRG2	2.346666667	0.09	-4.704544116
143662	MUC15	5.44	0.206666667	-4.718229032
1535	CYBA	20.83333333	0.723333333	-4.848089242
11030	RBPMS	3.693333333	0.123333333	-4.9042888
9074	CLDN6	13.81	0.433333333	-4.994092292
26002	MOXD1	8.086666667	0.25	-5.015545145
80034	CSRNP3	2.506666667	0.076666667	-5.031026896
100288801	FRG2C	6.66	0.2	-5.057450272
114907	FBXO32	1.24	0.036666667	-5.079727192
221002	RASGEF1A	5.063333333	0.146666667	-5.109474536
10752	CHL1	3.016666667	0.083333333	-5.177917792
29951	PDZRN4	2.73	0.066666667	-5.355791547
118427	OLFM3	2.07	0.05	-5.371558863
1902	LPAR1	1.296666667	0.026666667	-5.603626345
9388	LIPG	3.103333333	0.046666667	-6.055282436
429	ASCL1	1.313333333	0.013333333	-6.622051819
203328	SUSD3	1.063333333	0.01	-6.732450113
170825	GSX2	27.17	0.243333333	-6.802936792
8492	PRSS12	1.236666667	0.01	-6.950312876
8363	HIST1H4J	1.246666667	0.01	-6.961931959
100137049	PLA2G4B	1.806666667	0.01	-7.497186541

Table S2e

Gene ID	Gene Symbol	hdecadkd Expression	hdp190A Expression	log2FoldChange (hdp190A/hdecadkd)
353513	VCY1B	0.01	1.473333333	7.202940059
24150	TP53TG3	0.01	1.076666667	6.750427854
23544	SEZ6L	0.026666667	1.18	5.46760555
257019	FRMD3	0.13	5.273333333	5.342131666
999	CDH1	1.286666667	38.07333333	4.887070632
6422	SFRP1	0.06	1.436666667	4.581619058
4608	MYBPH	0.13	3.1	4.575684687
3777	KCNK3	0.136666667	3.07	4.489505341
140706	CCM2L	3.16	66.3	4.391012407
165	AEBP1	0.173333333	3.47	4.323314635
26289	AK5	0.156666667	2.75	4.133661457
340061	TMEM173	0.173333333	2.883333333	4.056116604
960	CD44	0.193333333	3.03	3.970155489
4005	LMO2	0.283333333	4.33	3.933794779
4222	MEOX1	6.673333333	101.3666667	3.925031937
10046	MAMLD1	0.41	5.596666667	3.77087201
83593	RASSF5	0.193333333	2.49	3.686983438
81035	COLEC12	2.386666667	30.56333333	3.678732905
9573	GDF3	0.083333333	1.04	3.641546029
8638	OASL	0.333333333	3.836666667	3.524815928
3489	IGFBP6	2.013333333	21.84	3.439314902
84419	C15orf48	0.14	1.5	3.421463768
286	ANK1	0.966666667	9.996666667	3.370356717
3815	KIT	0.146666667	1.5	3.354349573
10267	RAMP1	2.233333333	22.64333333	3.341815854
51702	PADI3	0.496666667	4.933333333	3.31221294
79836	LONRF3	0.156666667	1.533333333	3.290901199
4103	MAGEA4	0.123333333	1.176666667	3.254071008
190	NR0B1	0.293333333	2.726666667	3.216525414
27063	ANKRD1	0.25	2.316666667	3.212050477
1141	CHRNB2	0.136666667	1.26	3.20469042
2022	ENG	1.23	11.19666667	3.186339074
4907	NT5E	1.58	14.36666667	3.184728905
3875	KRT18	12.74666667	114.88	3.171935727
9235	IL32	0.293333333	2.586666667	3.140481224
10568	SLC34A2	4.28	37.37333333	3.126326542
23220	DTX4	0.123333333	1.053333333	3.094327383
246	ALOX15	0.486666667	4.146666667	3.090946211
90139	TSPAN18	0.866666667	7.24	3.062440575
221662	RBM24	3.376666667	27.81333333	3.042104984

51435	SCARA3	0.543333333	4.463333333	3.038212091
91683	SYT12	0.843333333	6.906666667	3.033814713
3560	IL2RB	0.286666667	2.31	3.010446787
4629	MYH11	0.483333333	3.876666667	3.003726291
389432	SAMD5	0.186666667	1.496666667	3.003216713
51700	CYB5R2	0.256666667	2.053333333	3
57699	CPNE5	0.13	1.036666667	2.995368551
89927	C16orf45	5.756666667	45.38333333	2.978856994
57158	JPH2	0.236666667	1.846666667	2.963995047
1277	COL1A1	21.12333333	153.6866667	2.863082587
9394	HS6ST1	19.78666667	143.5533333	2.858986334
729262	NUTM2B	0.166666667	1.2	2.847996907
55450	CAMK2N1	1.806666667	12.82	2.826994006
343702	XKR7	0.276666667	1.916666667	2.792378714
219699	UNC5B	0.423333333	2.876666667	2.764532062
2113	ETS1	3.08	20.82	2.756967813
56892	TCIM	4.806666667	32.13333333	2.740961982
23551	RASD2	0.393333333	2.62	2.735742453
6588	SLN	4.54	29.99666667	2.72403799
7481	WNT11	0.17	1.113333333	2.711278951
29118	DDX25	0.196666667	1.283333333	2.706071586
55959	SULF2	2.496666667	16.02	2.681799025
1381	CRABP1	6.126666667	39.05333333	2.672271427
5155	PDGFB	1.523333333	9.69	2.669265001
9211	LGI1	0.803333333	5.09	2.663595011
57540	DISP3	0.41	2.59	2.659256283
219348	PLAC9	0.256666667	1.62	2.658025963
7262	PHLDA2	0.653333333	4.103333333	2.650905202
7164	TPD52L1	2.043333333	12.83333333	2.650899467
11098	PRSS23	12.14	74.70666667	2.62146857
286499	FAM133A	0.26	1.593333333	2.615464589
388662	SLC6A17	0.32	1.956666667	2.612254192
388021	TMEM179	0.19	1.14	2.584962501
4199	ME1	0.293333333	1.743333333	2.571235518
54738	FEV	0.21	1.23	2.550197083
182	JAG1	11.48333333	66.42333333	2.532148641
9289	ADGRG1	2.346666667	13.14666667	2.486012218
5328	PLAU	3.883333333	21.63333333	2.477888523
60529	ALX4	2.42	13.25	2.452913407
85444	LRRCC1	0.256666667	1.4	2.447458977
102724594	U2AF1L5	0.946666667	5.09	2.426737227
54596	L1TD1	2.53	13.53333333	2.419307937
4313	MMP2	0.716666667	3.833333333	2.419225296

6236	RRAD	0.326666667	1.74	2.413196152
653689	GSTT2B	0.4	2.106666667	2.396890153
151195	CCNYL1	18.86	98.92333333	2.390981178
3707	ITPKB	0.393333333	2.04	2.374744793
27129	HSPB7	1.006666667	5.216666667	2.373542202
89796	NAV1	0.393333333	2.023333333	2.362909657
339761	CYP27C1	0.513333333	2.613333333	2.347923303
8706	B3GALNT1	0.286666667	1.456666667	2.345224715
84894	LINGO1	2.006666667	10.17333333	2.34191957
8362	HIST1H4K	0.996666667	5.026666667	2.334419039
26507	CNNM1	3.08	15.48333333	2.32971384
9535	GMFG	0.56	2.81	2.327071398
141	ADPRH	1.07	5.353333333	2.32282669
1187	CLCNKA	0.393333333	1.966666667	2.321928095
771	CA12	3.053333333	15.16	2.311812751
3976	LIF	0.313333333	1.513333333	2.271959636
162494	RHBDL3	5.036666667	24.29666667	2.27021724
5731	PTGER1	4.053333333	19.50666667	2.266786541
7424	VEGFC	0.29	1.393333333	2.264415636
1991	ELANE	2.033333333	9.716666667	2.256614736
978	CDA	0.7	3.333333333	2.251538767
11240	PADI2	3.793333333	17.90666667	2.238958747
91624	NEXN	3.993333333	18.36333333	2.201162555
4884	NPTX1	3.413333333	15.59333333	2.191676146
169834	ZNF883	0.286666667	1.306666667	2.188445089
58189	WFDC1	2.75	12.52	2.186731039
6999	TDO2	0.293333333	1.333333333	2.184424571
54935	DUSP23	1.24	5.633333333	2.18364872
3437	IFIT3	0.336666667	1.526666667	2.180992305
9331	B4GALT6	1.243333333	5.636666667	2.180629124
5999	RGS4	0.87	3.916666667	2.170539045
163782	KANK4	1.773333333	7.963333333	2.166908703
4773	NFATC2	1.533333333	6.883333333	2.166436015
5950	RBP4	0.323333333	1.446666667	2.16163839
11013	TMSB15A	0.313333333	1.393333333	2.15277028
9241	NOG	3.94	17.48666667	2.149987772
1136	CHRNA3	1.143333333	5.073333333	2.149687877
10873	ME3	0.743333333	3.286666667	2.144543936
55806	HR	2.206666667	9.696666667	2.135620174
50944	SHANK1	0.426666667	1.873333333	2.13442632
9052	GPRC5A	0.59	2.576666667	2.126719054
1465	CSRP1	19.99	87.22	2.125380518
51334	PRR16	6.196666667	26.99666667	2.123217016

2342	FNTB	1.396666667	6.083333333	2.122874315
8651	SOCS1	1.516666667	6.596666667	2.120833162
91461	PKDCC	1.416666667	6.113333333	2.109458893
8605	PLA2G4C	0.71	3.053333333	2.104494168
94032	CAMK2N2	0.7	2.996666667	2.097931788
360	AQP3	0.3	1.283333333	2.096861539
10266	RAMP2	1.096666667	4.666666667	2.089267338
1823	DSC1	0.78	3.306666667	2.083831591
146664	MGAT5B	3.376666667	14.27333333	2.079650622
627	BDNF	0.683333333	2.886666667	2.078743115
63827	BCAN	1.243333333	5.236666667	2.074435645
53637	S1PR5	9.323333333	39.01333333	2.065049516
55466	DNAJA4	0.58	2.426666667	2.064851144
11131	CAPN11	0.67	2.783333333	2.054580696
8991	SELENBP1	0.94	3.9	2.052741462
79883	PODNL1	0.32	1.326666667	2.05166212
25945	NECTIN3	0.433333333	1.783333333	2.041027268
9900	SV2A	0.923333333	3.786666667	2.036004953
136227	COL26A1	6.266666667	25.67333333	2.034500461
83849	SYT15	0.663333333	2.713333333	2.032260364
2134	EXTL1	0.936666667	3.82	2.027965009
338773	TMEM119	0.97	3.943333333	2.023359015
153572	IRX2	0.68	2.76	2.021061616
1435	CSF1	7.55	30.60333333	2.019140251
10581	IFITM2	0.736666667	2.983333333	2.017841313
408	ARRB1	6.723333333	27.06333333	2.009090961
54625	PARP14	0.696666667	2.8	2.006886386
1464	CSPG4	1.05	4.18	1.993113614
140733	MACROD2	0.263333333	1.046666667	1.990840001
58985	IL22RA1	0.393333333	1.563333333	1.990801063
3090	HIC1	0.276666667	1.093333333	1.982512573
1366	CLDN7	6.426666667	25.31	1.977564957
4917	NTN3	0.366666667	1.44	1.973527789
9469	CHST3	4.596666667	17.97333333	1.96719804
283948	NHLRC4	0.48	1.873333333	1.964501319
1001	CDH3	45.19333333	175.8866667	1.960464246
93099	DMKN	0.563333333	2.183333333	1.95447166
64073	C19orf33	1.216666667	4.71	1.952793097
343637	RSPO4	1.023333333	3.96	1.952224275
84631	SLITRK2	2.05	7.9	1.946228744
80326	WNT10A	6.503333333	24.86	1.934575019
100533105	C8orf44-SGK3	0.333333333	1.27	1.929790998
3563	IL3RA	1.3	4.95	1.928916902

84709	MGARP	3.533333333	13.43666667	1.927073518
653	BMP5	1.046666667	3.97	1.923336949
127294	MYOM3	0.75	2.843333333	1.92262074
2150	F2RL1	3.45	13.07333333	1.921958768
5054	SERPINE1	0.756666667	2.84	1.908161133
4940	OAS3	1.733333333	6.496666667	1.906150562
51286	CEND1	3.76	14.07333333	1.904159511
3620	IDO1	0.55	2.056666667	1.902804465
9788	MTSS1	0.473333333	1.766666667	1.90010143
28	ABO	0.793333333	2.946666667	1.893084796
4651	MYO10	11.98333333	44.49666667	1.892667995
84969	TOX2	1.896666667	6.97	1.877692504
9148	NEURL1	0.33	1.203333333	1.866498407
57125	PLXDC1	0.45	1.64	1.865698908
2837	UTS2R	0.56	2.033333333	1.86034801
5698	PSMB9	1.816666667	6.58	1.856793855
5730	PTGDS	10.75333333	38.89333333	1.854738948
4162	MCAM	5.82	21.01	1.851985104
388610	TRNP1	1.88	6.776666667	1.849843147
81706	PPP1R14C	2.553333333	9.17	1.844539842
57822	GRHL3	0.466666667	1.67	1.839383776
9087	TMSB4Y	0.343333333	1.226666667	1.837061429
27439	TMEM121B	0.49	1.72	1.811554911
2303	FOXC2	0.393333333	1.38	1.810843908
684	BST2	0.45	1.566666667	1.79970135
10677	AVIL	0.376666667	1.31	1.79820654
2878	GPX3	1.92	6.666666667	1.795859283
51764	GNG13	1.196666667	4.146666667	1.792930736
9645	MICAL2	0.463333333	1.603333333	1.790952011
1159	CKMT1B	2.096666667	7.24	1.787892181
26064	RAI14	2.046666667	7.036666667	1.781616018
55332	DRAM1	2.24	7.693333333	1.780110086
2049	EPHB3	1.64	5.623333333	1.777729753
50509	COL5A3	0.443333333	1.52	1.777607579
647024	C6orf132	1.21	4.14	1.77462372
91608	RASL10B	3.213333333	10.90666667	1.763067697
330	BIRC3	0.573333333	1.94	1.758610588
1839	HBEGF	23.28	78.4	1.751762596
388585	HES5	11.60333333	38.96666667	1.747701211
53840	TRIM34	0.536666667	1.8	1.745898719
2983	GUCY1B1	2.643333333	8.853333333	1.743862376
55024	BANK1	0.37	1.236666667	1.74085951
140876	RIPOR3	6.07	20.26333333	1.739103096

55170	PRMT6	1.48	4.936666667	1.737940059
55531	ELMOD1	0.486666667	1.62	1.734987945
150209	AIFM3	0.463333333	1.54	1.732807969
7475	WNT6	5.696666667	18.90666667	1.730705136
10202	DHRS2	0.466666667	1.543333333	1.725585366
26030	PLEKHG3	2.736666667	9.033333333	1.722838724
83468	GLT8D2	3.34	10.99333333	1.71870889
54511	HMGCLL1	0.85	2.79	1.714730376
7052	TGM2	2.206666667	7.226666667	1.711461635
1840	DTX1	2.876666667	9.416666667	1.710818403
3705	ITPK1	8.583333333	28.05666667	1.708733685
56912	IFT46	2.04	6.616666667	1.697535449
7043	TGFB3	1.866666667	6.05	1.696470816
11346	SYNPO	3.353333333	10.86333333	1.695799047
23498	HAAO	0.833333333	2.68	1.685267407
994	CDC25B	42.94666667	137.7433333	1.68136443
5129	CDK18	8.04	25.78333333	1.681171384
766	CA7	0.816666667	2.616666667	1.679910905
3856	KRT8	0.56	1.776666667	1.6656743
56147	PCDHA1	0.356666667	1.13	1.663674477
23769	FLRT1	0.543333333	1.72	1.662499101
283152	CCDC153	1.476666667	4.67	1.661078352
10148	EBI3	7.94	25.04333333	1.657215689
4647	MYO7A	0.556666667	1.75	1.65246932
6441	SFTPD	0.606666667	1.903333333	1.649552295
3383	ICAM1	1.82	5.693333333	1.645335119
23586	DDX58	1.356666667	4.233333333	1.641727797
7042	TGFB2	14.80666667	46.18333333	1.641125413
10232	MSLN	0.716666667	2.223333333	1.633350102
83844	USP26	0.73	2.263333333	1.632480705
23231	SEL1L3	4.376666667	13.53333333	1.628612811
51063	CALHM2	0.436666667	1.35	1.628355096
4071	TM4SF1	1.353333333	4.163333333	1.621221844
2301	FOXE3	0.613333333	1.88	1.615989396
83857	TMTC1	0.873333333	2.67	1.612235431
26230	TIAM2	1.003333333	3.066666667	1.611870374
3750	KCND1	1.01	3.086666667	1.6116944
79930	DOK3	0.62	1.893333333	1.610588308
79690	GAL3ST4	1.623333333	4.92	1.599699044
83743	GRWD1	4.813333333	14.50333333	1.591276275
58477	SRPRB	3.713333333	11.13333333	1.58409887
3237	HOXD11	2.383333333	7.133333333	1.58159565
83666	PARP9	1.656666667	4.953333333	1.580116359

23180	RFTN1	0.593333333	1.77	1.57683462
94240	EPSTI1	1.196666667	3.556666667	1.571504427
55890	GPRC5C	5.49	16.27	1.567336197
51127	TRIM17	0.943333333	2.793333333	1.566148191
133022	TRAM1L1	1.283333333	3.796666667	1.564837396
151963	MB21D2	2.816666667	8.323333333	1.563172575
167838	TXLNB	0.666666667	1.963333333	1.558267634
1158	CKM	3.396666667	9.993333333	1.556846332
348487	FAM131C	1.956666667	5.756666667	1.556835674
8372	HYAL3	0.64	1.88	1.554588852
548596	CKMT1A	7.523333333	22.09	1.553949501
286319	TUSC1	0.95	2.783333333	1.550814278
7779	SLC30A1	10.77666667	31.55666667	1.550033815
3106	HLA-B	6.863333333	20.08666667	1.549256843
10763	NES	12.45666667	36.45333333	1.549132679
3965	LGALS9	0.62	1.813333333	1.54830403
9478	CABP1	2.143333333	6.26	1.54630642
50861	STMN3	13.87333333	40.47333333	1.544657208
84734	FAM167B	0.47	1.366666667	1.539928747
23708	GSPT2	1.766666667	5.136666667	1.539802597
221	ALDH3B1	4.65	13.50666667	1.538369052
401027	C2orf66	0.49	1.416666667	1.531646686
4232	MEST	13.44	38.84666667	1.531257672
1102	RCBTB2	1.983333333	5.723333333	1.528928466
140576	S100A16	2.5	7.21	1.528071165
10257	ABCC4	7.923333333	22.77	1.522954889
55715	DOK4	20.26666667	58.23333333	1.522736379
51363	CHST15	0.736666667	2.116666667	1.522710222
2048	EPHB2	1.67	4.793333333	1.521181167
111	ADCY5	1.36	3.9	1.519867472
5205	ATP8B1	0.61	1.746666667	1.517723163
22837	COBLL1	0.36	1.03	1.516575526
9699	RIMS2	0.52	1.483333333	1.512259307
23316	CUX2	0.636666667	1.81	1.50737956
3755	KCNG1	4.57	12.97333333	1.505283139
51259	TMEM216	4.69	13.31	1.504850743
4995	OR3A2	1.263333333	3.58	1.50272424
5818	NECTIN1	1.366666667	3.856666667	1.49669305
4101	MAGEA2	1.76	4.96	1.494764692
22979	EFR3B	5.636666667	15.85	1.491568681
3281	HSBP1	11.64	32.66666667	1.48872819
22882	ZHX2	0.906666667	2.543333333	1.488076406
85315	PAQR8	1.82	5.086666667	1.482782106

102723680	CT45A9	3.636666667	10.13	1.477945573
11167	FSTL1	17.07666667	47.5	1.475901122
1831	TSC22D3	8.126666667	22.58333333	1.474522821
374872	PEAK3	0.493333333	1.37	1.473541218
595	CCND1	9.863333333	27.33	1.470338264
9084	VCY	9.026666667	25.00666667	1.470047524
80017	DGLUCY	2.9	8.003333333	1.464548097
8061	FOSL1	2.006666667	5.516666667	1.458995825
163732	CITED4	13.27666667	36.49333333	1.458739955
8329	HIST1H2AI	2.156666667	5.926666667	1.458417707
388591	RNF207	0.673333333	1.85	1.458132478
344022	NOTO	1.086666667	2.976666667	1.453788211
163154	PRR22	0.746666667	2.043333333	1.452388342
54937	SOHLH2	4.116666667	11.24666667	1.449948932
7168	TPM1	90.43	246.7833333	1.448371596
6533	SLC6A6	2.666666667	7.276666667	1.448240225
79642	ARSJ	6.146666667	16.77	1.448006534
23371	TNS2	3.776666667	10.24666667	1.439969304
64759	TNS3	21.25666667	57.65666667	1.439572052
7771	ZNF112	0.843333333	2.286666667	1.439071191
2330	FMO5	0.67	1.816666667	1.439060728
283212	KLHL35	1.3	3.523333333	1.438429348
7134	TNNC1	4.793333333	12.98333333	1.437559653
283008	NUTM2E	0.796666667	2.156666667	1.436755094
79132	DHX58	1.443333333	3.903333333	1.435302146
4921	DDR2	1.55	4.19	1.434682028
151636	DTX3L	2.016666667	5.45	1.434283588
113791	PIK3IP1	4.643333333	12.52666667	1.431769809
4914	NTRK1	0.46	1.24	1.430634354
9891	NUAK1	1.556666667	4.193333333	1.429637467
197259	MLKL	1.38	3.7	1.422857004
800	CALD1	1.553333333	4.16	1.421216074
55924	FAM212B	0.5	1.336666667	1.418639736
92154	MTSS1L	4.896666667	13.07666667	1.41712294
6320	CLEC11A	17.81333333	47.55	1.416487834
4330	MN1	11.77	31.36	1.413811239
745	MYRF	1.65	4.393333333	1.41284994
84913	ATOH8	0.423333333	1.126666667	1.41219475
254295	PHYHD1	3.92	10.42333333	1.410891159
169611	OLFML2A	9.3	24.71666667	1.410181571
59283	CACNG8	1.033333333	2.723333333	1.398067863
6764	ST5	14.54333333	38.26	1.395478901
2766	GMPR	0.78	2.05	1.394077881

467	ATF3	3.526666667	9.246666667	1.39062816
4060	LUM	0.396666667	1.04	1.390584456
8407	TAGLN2	32.99	86.3	1.387331782
151473	SLC16A14	0.793333333	2.073333333	1.385953007
1303	COL12A1	1.32	3.446666667	1.38466385
117854	TRIM6	3.866666667	10.09333333	1.3842404
161145	TMEM229B	0.606666667	1.583333333	1.383989063
6913	TBX15	0.836666667	2.183333333	1.383807542
54947	LPCAT2	1.74	4.54	1.383604991
30832	ZNF354C	0.526666667	1.37	1.379213836
27254	CSDC2	4.213333333	10.94333333	1.377018286
4493	MT1E	7.496666667	19.47	1.376931724
2569	GABRR1	0.476666667	1.236666667	1.37540404
114880	OSBPL6	5.333333333	13.83	1.374691752
57758	SCUBE2	2.283333333	5.906666667	1.371202711
3306	HSPA2	69.15	178.62	1.369092471
91807	MYLK3	0.956666667	2.47	1.368422806
59285	CACNG6	1.183333333	3.053333333	1.367528574
65983	GRAMD2B	1.89	4.86	1.362570079
26609	VCX	3.01	7.736666667	1.36194863
266727	MDGA1	0.496666667	1.273333333	1.358260308
220323	OAF	0.543333333	1.39	1.355175419
6398	SECTM1	3.186666667	8.103333333	1.34646737
65009	NDRG4	1.036666667	2.63	1.34311072
56137	PCDHA12	0.623333333	1.58	1.341848789
85456	TNKS1BP1	6.18	15.64333333	1.339869216
57552	NCEH1	2.62	6.63	1.339442059
115948	CCDC151	7.89	19.93666667	1.337327012
440804	RIMBP3B	0.94	2.373333333	1.336182079
2678	GGT1	6.853333333	17.30333333	1.336172223
153571	C5orf38	1.563333333	3.946666667	1.336009253
23529	CLCF1	0.42	1.06	1.335603032
84875	PARP10	0.643333333	1.623333333	1.335320925
22809	ATF5	21.04333333	53.09	1.335076891
57514	ARHGAP31	0.693333333	1.746666667	1.332983283
4135	MAP6	5.83	14.68666667	1.332939206
57165	GJC2	0.75	1.886666667	1.330877052
57523	NYNRIN	6.633333333	16.67333333	1.329736627
2318	FLNC	5.843333333	14.68333333	1.329316023
1847	DUSP5	1.396666667	3.503333333	1.32674052
3049	HBQ1	1.446666667	3.626666667	1.325911609
10855	HPSE	1.073333333	2.69	1.325507985
56135	PCDHAC1	0.463333333	1.16	1.324002423

388564	TMEM238	1.083333333	2.696666667	1.315699985
2517	FUCA1	20.81666667	51.70666667	1.312611232
85300	ATCAY	8.97	22.26333333	1.311489723
83595	SOX7	0.563333333	1.393333333	1.306479696
54762	GRAMD1C	1.393333333	3.44	1.303868123
79158	GNPTAB	6.813333333	16.77666667	1.300023402
133383	SETD9	2.943333333	7.246666667	1.299866597
1917	EEF1A2	90.32666667	222.3233333	1.299435495
1318	SLC31A2	1.296666667	3.19	1.298748769
3604	TNFRSF9	0.436666667	1.073333333	1.297493877
5097	PCDH1	4.023333333	9.88	1.296119772
710	SERPING1	12.13333333	29.79333333	1.296013594
57542	KLHL42	1.626666667	3.986666667	1.293264337
4920	ROR2	4.456666667	10.89333333	1.289408518
400451	FAM174B	0.5	1.22	1.286881148
9423	NTN1	5.296666667	12.91666667	1.286077186
10761	PLAC1	2.496666667	6.073333333	1.282485335
24141	LAMP5	3.986666667	9.69	1.281313682
25992	SNED1	0.49	1.19	1.280107919
253461	ZBTB38	3.39	8.21	1.276096949
3207	HOXA11	0.516666667	1.25	1.27462238
4584	MUC3A	0.596666667	1.443333333	1.274407437
222183	SRRM3	4.246666667	10.26666667	1.273565073
151176	ERFE	2.14	5.173333333	1.273483355
79443	FYCO1	2.27	5.486666667	1.273237632
85376	RIMBP3	1.09	2.63	1.270734665
56108	PCDHGA7	1.083333333	2.613333333	1.270413936
84152	PPP1R1B	0.656666667	1.583333333	1.269731884
9454	HOMER3	7.51	18.09333333	1.268573407
7290	HIRA	6.593333333	15.87333333	1.267525195
494513	PJVK	0.446666667	1.073333333	1.264827688
3233	HOXD4	0.606666667	1.456666667	1.263694829
79815	NIPAL2	0.95	2.28	1.263034406
8828	NRP2	0.45	1.08	1.263034406
857	CAV1	13.34333333	31.93666667	1.259094627
6615	SNAI1	4.023333333	9.61	1.256145161
23624	CBLC	0.67	1.6	1.255838904
342132	ZNF774	1.093333333	2.606666667	1.253472793
79148	MMP28	0.493333333	1.173333333	1.249978253
5332	PLCB4	1.676666667	3.986666667	1.249587084
57216	VANGL2	0.95	2.256666667	1.248193915
284086	NEK8	1.486666667	3.526666667	1.246224012
283869	NPW	23.82666667	56.47	1.244907498

3675	ITGA3	5.99	14.19333333	1.244585541
57583	TMEM181	3.633333333	8.603333333	1.243602006
2159	F10	0.68	1.61	1.243454037
79183	TTPAL	6.58	15.54666667	1.240445799
148932	MOB3C	6.04	14.26666667	1.240027841
148545	NBPF4	3.676666667	8.676666667	1.238742521
10039	PARP3	2.32	5.473333333	1.238294916
128434	VSTM2L	13.75	32.38	1.235671367
3931	LCAT	1.29	3.033333333	1.233532979
5920	RARRES3	1.646666667	3.87	1.232785025
23600	AMACR	10.67333333	25.07666667	1.232334783
231	AKR1B1	26.21	61.56666667	1.232032107
132430	PABPC4L	0.516666667	1.213333333	1.231670235
106821730	BUB1B-PAK6	0.86	2.016666667	1.229564077
100528064	NEDD8-MDP1	4.586666667	10.74666667	1.228371274
51306	FAM13B	2.416666667	5.66	1.227783559
51760	SYT17	3.656666667	8.556666667	1.22651977
641517	DEFB109B	0.786666667	1.84	1.225881407
10481	HOXB13	1.766666667	4.126666667	1.22394705
134	ADORA1	0.45	1.05	1.222392421
5176	SERPINF1	31.63333333	73.8	1.22217523
145376	PPP1R36	1.313333333	3.06	1.220298524
83877	TM2D2	10.58666667	24.65666667	1.219729363
91977	MYOZ3	0.943333333	2.196666667	1.219476412
130576	LYPD6B	2.293333333	5.34	1.219393678
3487	IGFBP4	13.34666667	31.03333333	1.217339194
728392	LOC728392	8.85	20.57333333	1.217026201
100271715	ARHGEF33	1.196666667	2.78	1.21606354
8970	HIST1H2BJ	2.406666667	5.586666667	1.214951407
122773	KLHDC1	0.716666667	1.663333333	1.214703156
8293	SERF1A	2.03	4.703333333	1.212203855
246329	STAC3	0.73	1.69	1.211054877
51087	YBX2	12.62	29.19	1.209762301
91010	FMNL3	4.143333333	9.573333333	1.208229453
2042	EPHA3	26.75	61.77	1.207367439
9673	SLC25A44	5.153333333	11.89	1.206170897
51029	DESI2	5.603333333	12.92666667	1.205993079
4792	NFKBIA	43.60666667	100.5633333	1.205483757
9310	ZNF235	0.623333333	1.436666667	1.204649599
84450	ZNF512	6.313333333	14.53666667	1.20322266
27122	DKK3	3.59	8.253333333	1.200993065
51440	HPCAL4	0.91	2.09	1.199564492
4851	NOTCH1	3.84	8.816666667	1.199127006

79697	RIOX1	5.32	12.21333333	1.198958852
728118	NUTM2A	0.513333333	1.176666667	1.196737833
641	BLM	2.89	6.616666667	1.195035109
94059	LENG9	2.116666667	4.843333333	1.194206206
2852	GPER1	2.256666667	5.163333333	1.194109405
29993	PACSIN1	2.11	4.823333333	1.192787517
283149	BCL9L	22.66333333	51.72333333	1.190455184
221061	FAM171A1	2.913333333	6.646666667	1.189960225
8365	HIST1H4H	1.883333333	4.296666667	1.189929491
57458	TMCC3	2.733333333	6.23	1.188570754
114990	VASN	1.796666667	4.093333333	1.187953383
6939	TCF15	1.296666667	2.953333333	1.187536544
114625	ERMAP	1.866666667	4.25	1.186998515
2149	F2R	4.876666667	11.1	1.186592408
51421	AMOTL2	3.203333333	7.276666667	1.183703794
5971	RELB	3.91	8.88	1.183391069
7993	UBXN8	3.446666667	7.81	1.180120769
284307	ZIK1	1.936666667	4.386666667	1.17954942
57571	CARNS1	2.163333333	4.9	1.179525772
8353	HIST1H3E	0.996666667	2.256666667	1.179010349
8287	USP9Y	2.34	5.296666667	1.178576189
80216	ALPK1	1.163333333	2.63	1.176798264
139411	PTCHD1	0.793333333	1.79	1.173960515
148170	CDC42EP5	6.78	15.28333333	1.172602055
7070	THY1	44.24666667	99.71	1.172169427
122786	FRMD6	2.92	6.573333333	1.170656777
1602	DACH1	3.96	8.913333333	1.170464629
253152	EPHX4	1.27	2.856666667	1.169504207
9118	INA	24.06666667	54.10333333	1.168681146
5139	PDE3A	10.42666667	23.43	1.168078942
79922	MRM1	2.336666667	5.246666667	1.166949191
56971	CEACAM19	0.45	1.01	1.166358386
6277	S100A6	78.35333333	175.7433333	1.165403406
283130	SLC25A45	2.853333333	6.396666667	1.16467201
100302652	GPR75-ASB3	1.093333333	2.45	1.164048435
23780	APOL2	4.213333333	9.44	1.163824802
219833	C11orf45	0.583333333	1.306666667	1.163498732
56164	STK31	1.036666667	2.32	1.162172726
79081	LBHD1	0.883333333	1.976666667	1.162039745
4776	NFATC4	1.61	3.6	1.160936218
55092	TMEM51	3.263333333	7.296666667	1.16089119
494143	CHAC2	1.633333333	3.65	1.160077215
402682	UFSP1	1.046666667	2.336666667	1.158649885

3227	HOXC11	2.293333333	5.113333333	1.156818013
5753	PTK6	1.15	2.563333333	1.156387236
198437	LKAAEAR1	1.703333333	3.793333333	1.155105361
57605	PITPNM2	1.126666667	2.506666667	1.153709415
4234	METTL1	5.073333333	11.28666667	1.153613614
57556	SEMA6A	8.986666667	19.98	1.152698587
5311	PKD2	2.483333333	5.52	1.152390342
8187	ZNF239	0.95	2.11	1.15124358
51474	LIMA1	16.73	37.15666667	1.151183638
65251	ZNF649	0.893333333	1.983333333	1.150656668
55179	FAIM	6.543333333	14.52333333	1.15027494
100	ADA	24.8	55.03333333	1.149965594
27132	CPNE7	1.036666667	2.3	1.149681782
200844	C3orf67	0.9	1.996666667	1.149596596
115727	RASGRP4	1.07	2.373333333	1.149303944
8996	NOL3	12.37333333	27.41333333	1.147643554
200035	NUDT17	2.5	5.53	1.145351386
4062	LY6H	1.39	3.073333333	1.144719367
2316	FLNA	64.2	141.8633333	1.143856549
100037417	DDTL	2.106666667	4.65	1.142268659
91050	CCDC149	1.11	2.45	1.142222073
817	CAMK2D	16.1	35.45	1.138724939
83786	FRMD8	6.566666667	14.45666667	1.138501814
825	CAPN3	0.63	1.386666667	1.138197294
64115	VSIR	1.03	2.266666667	1.137927908
51816	ADA2	4.993333333	10.96666667	1.13504996
9619	ABCG1	2.85	6.253333333	1.133663503
9873	FCHSD2	5.59	12.26333333	1.133430987
64711	HS3ST6	3.7	8.113333333	1.132769491
1747	DLX3	0.536666667	1.176666667	1.132607495
169200	TMEM64	7.54	16.52	1.131577258
8741	TNFSF13	0.676666667	1.48	1.129079949
26509	MYOF	20.40333333	44.60333333	1.128346663
268	AMH	0.82	1.79	1.126263773
647310	TEX22	1.44	3.143333333	1.126226459
4591	TRIM37	23.40333333	51.03333333	1.12472585
10384	BTN3A3	1.773333333	3.866666667	1.124626655
7568	ZNF20	0.733333333	1.596666667	1.122522132
493861	EID3	2.03	4.416666667	1.121478226
57596	BEGAIN	5.243333333	11.40666667	1.121321089
43	ACHE	2.15	4.676666667	1.121143943
643699	GOLGA8N	1.483333333	3.223333333	1.119710554
2307	FOXS1	2.4	5.213333333	1.119171701

100130519	TMEM221	2.523333333	5.476666667	1.117967275
9540	TP53I3	20.12666667	43.65333333	1.116983565
55244	SLC47A1	0.89	1.93	1.116723606
10893	MMP24	0.643333333	1.393333333	1.114902095
9022	CLIC3	14.06333333	30.44666667	1.114345702
1138	CHRNA5	1.56	3.373333333	1.112628855
158880	USP51	0.646666667	1.396666667	1.110893592
9498	SLC4A8	0.84	1.813333333	1.110182918
5630	PRPH	15.02	32.34666667	1.106732237
90853	SPOCD1	1.893333333	4.076666667	1.106461569
51226	COPZ2	1.133333333	2.44	1.106308902
5961	PRPH2	1.673333333	3.6	1.105272043
1281	COL3A1	667.0233333	1434.223333	1.104460559
245806	VGLL2	1.016666667	2.183333333	1.102685664
25840	METTL7A	2.296666667	4.93	1.102046164
388849	CCDC188	0.766666667	1.64	1.097024454
113	ADCY7	6.326666667	13.53333333	1.096999735
55930	MYO5C	1.566666667	3.35	1.09646284
651746	ANKRD33B	17.10666667	36.57333333	1.096233452
100533496	TVP23C-CDRT4	0.823333333	1.76	1.096026888
374882	TMEM205	59.35666667	126.87	1.095868985
100129543	ZNF730	0.863333333	1.843333333	1.094327383
54510	PCDH18	18.53666667	39.57333333	1.094146755
440689	HIST2H2BF	2.07	4.413333333	1.092237949
29984	RHOD	8.623333333	18.37	1.091034073
9263	STK17A	3.773333333	8.023333333	1.088362184
4790	NFKB1	5.076666667	10.79333333	1.088187044
10900	RUNDC3A	1.556666667	3.306666667	1.086917571
8356	HIST1H3J	0.626666667	1.33	1.085656085
2296	FOXC1	37.67333333	79.90333333	1.084712001
100170841	EPOP	4.196666667	8.896666667	1.084021022
11142	PKIG	67.09666667	142.1533333	1.083134928
55500	ETNK1	3.433333333	7.273333333	1.083006764
90427	BMF	5.746666667	12.17333333	1.082926991
114757	CYGB	7.21	15.26333333	1.0819989
7022	TFAP2C	17.27	36.54	1.081208551
85461	TANC1	2.983333333	6.31	1.080714824
7132	TNFRSF1A	16.48333333	34.82666667	1.079184376
8835	SOCS2	4.81	10.16	1.078791603
203111	ERICH5	1.343333333	2.83	1.074984715
7041	TGFB1I1	21.61666667	45.51333333	1.074145177
4603	MYBL1	1.893333333	3.983333333	1.073047783
55742	PARVA	1.48	3.113333333	1.072862873

27235	COQ2	5.77	12.12333333	1.071143201
7544	ZFY	2.82	5.923333333	1.070714113
135	ADORA2A	1.01	2.12	1.069708972
81542	TMX1	7.956666667	16.69333333	1.069035996
6528	SLC5A5	3.07	6.44	1.068822033
57664	PLEKHA4	16.34333333	34.27	1.068243931
2317	FLNB	15.80666667	33.13333333	1.067750189
10825	NEU3	1.503333333	3.15	1.067186896
8459	TPST2	37.11333333	77.70666667	1.066100794
10241	CALCOCO2	53.77	112.48	1.064795123
79671	NLRX1	3.883333333	8.123333333	1.064776296
84072	HORMAD1	1.183333333	2.473333333	1.063600162
8826	IQGAP1	38.88333333	81.26666667	1.063511819
2624	GATA2	3.486666667	7.28	1.062090005
339768	ESPNL	0.7	1.46	1.060541542
56981	PRDM11	0.546666667	1.14	1.06030051
53335	BCL11A	3.11	6.48	1.059079233
8839	WISP2	0.863333333	1.793333333	1.054654075
6525	SMTN	7.416666667	15.4	1.054087516
157506	RDH10	45.31333333	93.95333333	1.052008725
27239	GPR162	15.13333333	31.36666667	1.051502425
102724428	SIK1B	6.216666667	12.88333333	1.051292784
25847	ANAPC13	21.98333333	45.53666667	1.05061853
1288	COL4A6	5.136666667	10.64	1.05059379
11182	SLC2A6	4.07	8.423333333	1.049362464
26095	PTPN20	1.976666667	4.086666667	1.047854969
56301	SLC7A10	5.146666667	10.63666667	1.047335856
26049	FAM169A	2.226666667	4.6	1.046748259
6275	S100A4	4.463333333	9.21	1.045079601
2669	GEM	0.886666667	1.826666667	1.042749647
8749	ADAM18	2.666666667	5.493333333	1.042644337
123688	HYKK	3.746666667	7.703333333	1.039875224
85369	STRIP1	13.22666667	27.16	1.038033955
924	CD7	50.86666667	104.41	1.037467433
100526783	C15orf38-AP3S2	1.24	2.543333333	1.036380436
2257	FGF12	4.826666667	9.896666667	1.035915491
9378	NRXN1	6.723333333	13.77666667	1.03497828
84074	QRICH2	0.69	1.413333333	1.034433497
9516	LITAF	37.55	76.90333333	1.034233225
375341	C3orf62	1.156666667	2.366666667	1.032883362
110599564	EEF1AKMT4	2.133333333	4.363333333	1.032321287
51704	GPRC5B	8.183333333	16.72666667	1.031389446
386724	AMIGO3	1.373333333	2.806666667	1.031175896

11043	MID2	2.116666667	4.323333333	1.030349983
90233	ZNF551	0.946666667	1.933333333	1.030161971
1800	DPEP1	15.21333333	31.03333333	1.028482376
200895	DHFR2	1.57	3.196666667	1.025803755
55243	KIRREL1	7.863333333	16.01	1.02576039
526	ATP6V1B2	12.17333333	24.77666667	1.025257843
6583	SLC22A4	0.596666667	1.213333333	1.023978863
57727	NCOA5	13.21	26.84333333	1.022933366
7511	XPNPEP1	13.89333333	28.22	1.022325211
122769	LRR1	3.246666667	6.593333333	1.022048749
91801	ALKBH8	1.916666667	3.89	1.0211707
6778	STAT6	34.96	70.91666667	1.020419541
2537	IFI6	64.88	131.6	1.020313764
388335	TMEM220	3.666666667	7.436666667	1.02018699
4843	NOS2	0.6	1.216666667	1.019899557
5027	P2RX7	0.603333333	1.223333333	1.019790366
115708	TRMT61A	3.046666667	6.173333333	1.018818028
147949	ZNF583	1.566666667	3.173333333	1.018300817
7204	TRIO	8.776666667	17.77666667	1.018239807
389524	GTF2IRD2B	2.17	4.393333333	1.017620922
79134	TMEM185B	6.89	13.94333333	1.016999609
100507588	TGFBR3L	6.35	12.84	1.015816705
54463	RETREG1	0.773333333	1.563333333	1.015463117
8357	HIST1H3H	10.39333333	21.00666667	1.015188826
55915	LANCL2	5.02	10.14333333	1.014772564
4784	NFIX	18.56333333	37.48666667	1.013921754
84812	PLCD4	4.683333333	9.456666667	1.01379602
53635	PTOV1	85.28333333	172.0933333	1.012855478
6385	SDC4	8.963333333	18.08666667	1.012819292
2944	GSTM1	0.573333333	1.156666667	1.012527098
7957	EPM2A	0.63	1.27	1.011404763
400720	ZNF772	1.053333333	2.123333333	1.011368814
116238	TLCD1	12.32	24.8	1.009337865
221468	TMEM217	1.276666667	2.566666667	1.007514054
55620	STAP2	10.06333333	20.22666667	1.007150329
51099	ABHD5	1.766666667	3.55	1.006789166
102723631	CT45A10	2.913333333	5.84	1.00329759
126017	ZNF813	0.96	1.923333333	1.002502507
53342	IL17D	1.55	3.103333333	1.001550452
389813	AJM1	3.326666667	6.66	1.001444862
57493	HEG1	1.666666667	3.336666667	1.001441974
125950	RAVER1	27.82	55.67333333	1.000864044
4148	MATN3	11.19666667	22.40666667	1.000858747

2826	CCR10	2.116666667	4.233333333	1
7275	TUB	0.643333333	1.286666667	1
5662	PSD	0.976666667	1.953333333	1
5167	ENPP1	1.4	0.7	-1
6474	SHOX2	1.596666667	0.796666667	-1.003015038
347475	CCDC160	11.92666667	5.94	-1.005656051
147111	NOTUM	7.17	3.563333333	-1.008745672
59	ACTA2	1.576666667	0.783333333	-1.009179427
16	AARS	46.63666667	23.16	-1.009829423
27253	PCDH17	21.79	10.81666667	-1.010410219
145788	C15orf65	5.63	2.79	-1.0128698
23593	HEBP2	18.2	9.016666667	-1.013272357
147798	TMC4	1.266666667	0.626666667	-1.015266757
4753	NELL2	188.8933333	93.42	-1.015768435
9467	SH3BP5	5.446666667	2.686666667	-1.01955624
5783	PTPN13	5.66	2.79	-1.020536931
1299	COL9A3	1.07	0.526666667	-1.022648739
90557	CCDC74A	38.73333333	19.06333333	-1.022775258
5454	POU3F2	34.10666667	16.78333333	-1.023024486
6006	RHCE	1.186666667	0.583333333	-1.024522319
221120	ALKBH3	4.55	2.233333333	-1.02666795
3998	LMAN1	43.87	21.52666667	-1.027109766
79096	C11orf49	50.14333333	24.54666667	-1.030530778
6885	MAP3K7	49.53	24.2	-1.033295573
8076	MFAP5	5.09	2.48	-1.037325536
727936	GXYLT2	4.616666667	2.246666667	-1.03906548
728340	GTF2H2C	8.886666667	4.306666667	-1.04507071
64762	GAREM1	3.51	1.696666667	-1.048767875
10683	DLL3	54.10666667	26.14	-1.049547223
1123	CHN1	17.59666667	8.496666667	-1.050333293
1950	EGF	4.993333333	2.41	-1.050970072
91409	CCDC74B	26.99333333	13.02333333	-1.051504387
25914	RTTN	6.206666667	2.993333333	-1.052065723
1848	DUSP6	75.51333333	36.30666667	-1.056496921
26355	FAM162A	73.38666667	35.2	-1.05994254
57167	SALL4	34.15333333	16.36333333	-1.061559725
114793	FMNL2	13.61333333	6.513333333	-1.063552399
3589	IL11	1.233333333	0.59	-1.06377591
3955	LFNG	20.60666667	9.856666667	-1.063939411
10434	LYPLA1	91.11	43.42	-1.069249683
113451	AZIN2	1.54	0.733333333	-1.070389328
5230	PGK1	611.95	290.5733333	-1.074511472
2827	GPR3	1.14	0.54	-1.078002512

6609	SMPD1	32.31333333	15.29	-1.079541175
970	CD70	17.74	8.383333333	-1.08141011
57639	CCDC146	1.996666667	0.943333333	-1.08175395
3592	IL12A	1.143333333	0.54	-1.082214763
9120	SLC16A6	1.503333333	0.71	-1.082274003
26227	PHGDH	66.16666667	31.1	-1.089190021
144406	WDR66	7.59	3.566666667	-1.089523495
28231	SLCO4A1	2.476666667	1.163333333	-1.090135174
100505385	IQCJ-SCHIP1	14.55	6.826666667	-1.091765939
152503	SH3D19	13.83333333	6.48	-1.094083118
64946	CENPH	21.38	9.983333333	-1.098668351
5309	PITX3	1.876666667	0.873333333	-1.103568111
23576	DDAH1	203.2933333	94.52666667	-1.104769618
6338	SCNN1B	99.40333333	46.20666667	-1.105193213
57211	ADGRG6	49.53333333	22.95	-1.109905556
54843	SYTL2	46.48666667	21.44	-1.116512075
26118	WSB1	78.76	36.27666667	-1.118421213
1263	PLK3	2.473333333	1.136666667	-1.121647448
308	ANXA5	489.2233333	224.72	-1.122364685
100996741	LOC100996741	3.753333333	1.723333333	-1.122970642
6319	SCD	282.8033333	129.77	-1.123842223
27345	KCNMB4	1.613333333	0.74	-1.124447371
144195	SLC2A14	2.543333333	1.163333333	-1.128456021
57688	ZSWIM6	3.933333333	1.796666667	-1.130429681
51778	MYOZ2	4.73	2.16	-1.130808871
8490	RGS5	39.23666667	17.89333333	-1.132780312
9832	JAKMIP2	2.503333333	1.14	-1.134816583
51135	IRAK4	10.63333333	4.84	-1.135514971
163486	DENND1B	2.756666667	1.253333333	-1.137154667
1740	DLG2	3.836666667	1.743333333	-1.138004982
79665	DHX40	72.26666667	32.7	-1.144039715
256979	SUN3	35.72	16.09666667	-1.149970118
55876	GSDMB	1.413333333	0.636666667	-1.150491627
2191	FAP	3.396666667	1.53	-1.150587993
201134	CEP112	9.51	4.283333333	-1.150711388
127602	DNAH14	9.666666667	4.35	-1.152003093
113263	GLCCI1	6.656666667	2.99	-1.154654443
6550	SLC9A3	16.86333333	7.563333333	-1.15679563
401478	LOC401478	1.503333333	0.673333333	-1.158772141
157574	FBXO16	9.28	4.143333333	-1.163332915
55435	AP1AR	4.143333333	1.84	-1.171086124
10631	POSTN	37.00666667	16.39	-1.174969338
84216	TMEM117	14.65666667	6.49	-1.175266648

29097	CNIH4	72.73333333	32.11	-1.179594031
282809	POC1B	16.75666667	7.38	-1.183042467
5756	TWF1	92.48	40.66666667	-1.185294656
57142	RTN4	338.23	148.3	-1.18948603
89765	RSPH1	3.943333333	1.723333333	-1.194213888
4661	MYT1	1.713333333	0.746666667	-1.198269627
9249	DHRS3	2.9	1.263333333	-1.198817553
8844	KSR1	4.73	2.056666667	-1.201532195
7107	GPR137B	3.09	1.343333333	-1.2017895
631	BFSP1	26.14	11.33	-1.20611128
347731	LRRTM3	1.446666667	0.626666667	-1.206962381
6902	TBCA	331.2866667	143.3933333	-1.208102189
3050	HBZ	1.68	0.726666667	-1.209095599
128344	PIFO	4.786666667	2.066666667	-1.211715629
441054	C4orf47	4.666666667	2.013333333	-1.212806373
6876	TAGLN	4.006666667	1.723333333	-1.21720071
3638	INSIG1	23.22	9.983333333	-1.21777447
8565	YARS	62.48	26.81666667	-1.220264533
53833	IL20RB	1.243333333	0.533333333	-1.221103725
7456	WIPF1	8.676666667	3.716666667	-1.223131602
3768	KCNJ12	10.27333333	4.4	-1.223328932
22843	PPM1E	2.226666667	0.953333333	-1.223832956
23440	OTP	17.99	7.693333333	-1.225514463
56660	KCNK12	3.466666667	1.48	-1.227951947
7423	VEGFB	53.67	22.76	-1.23761533
4885	NPTX2	3.76	1.593333333	-1.238684544
26018	LRIG1	3.06	1.296666667	-1.238723998
5228	PGF	28.35333333	11.97666667	-1.24329192
10793	ZNF273	4.166666667	1.76	-1.24331826
55093	WDYHV1	6.403333333	2.703333333	-1.244083699
55521	TRIM36	2.526666667	1.066666667	-1.244125943
3910	LAMA4	52.56666667	22.19	-1.244238587
83604	TMEM47	38.45666667	16.2	-1.247239906
8862	APLN	3.39	1.426666667	-1.248636977
51655	RASD1	4.05	1.703333333	-1.249561118
10924	SMPDL3A	2.11	0.886666667	-1.250779254
5144	PDE4D	5.066666667	2.126666667	-1.252442995
84978	FRMD5	1.826666667	0.766666667	-1.252542032
221935	SDK1	2.273333333	0.953333333	-1.253756592
7477	WNT7B	1.106666667	0.463333333	-1.256098359
23338	JADE2	37.41666667	15.63	-1.259363261
129804	FBLN7	1.893333333	0.79	-1.261003871
8553	BHLHE40	31.93333333	13.28333333	-1.265445932

94031	HTRA3	6.163333333	2.563333333	-1.265689721
25994	HIGD1A	47.75666667	19.83	-1.268017465
134728	IRAK1BP1	13	5.396666667	-1.268371138
83643	CCDC3	1.076666667	0.446666667	-1.269301164
6917	TCEA1	165.8833333	68.81333333	-1.269408908
4692	NDN	2.06	0.853333333	-1.271463028
5547	PRCP	143.4766667	59.39666667	-1.272362259
29057	FAM156A	1.316666667	0.543333333	-1.276980689
131540	ZDHHC19	2.036666667	0.836666667	-1.283485016
79867	TCTN2	13.96333333	5.696666667	-1.293453487
284716	RIMKLA	1.423333333	0.58	-1.295148764
347902	AMIGO2	8.963333333	3.64	-1.300096899
5106	PCK2	28.68333333	11.63666667	-1.301534835
136259	KLF14	1.013333333	0.41	-1.305413008
55605	KIF21A	34.39	13.9	-1.306904233
29968	PSAT1	41.70333333	16.82	-1.309984997
3212	HOXB2	36.77333333	14.81	-1.312088316
653519	GPR89A	26.78333333	10.75666667	-1.316104446
79400	NOX5	2.806666667	1.126666667	-1.316796987
1525	CXADR	141.64	56.85666667	-1.316827323
29969	MDFIC	18.83333333	7.55	-1.318739817
23235	SIK2	38.76	15.49333333	-1.322921003
3784	KCNQ1	7.056666667	2.82	-1.323291701
27244	SESN1	21.19333333	8.466666667	-1.323744519
84059	ADGRV1	16.10666667	6.433333333	-1.324019607
8531	YBX3	37.25	14.87	-1.324835778
81563	C1orf21	16.47	6.57	-1.325875279
9473	THEMIS2	5.306666667	2.113333333	-1.32828559
26511	CHIC2	50.19666667	19.96666667	-1.329998062
2119	ETV5	12.81	5.093333333	-1.330588433
1832	DSP	4.43	1.753333333	-1.3372064
221303	FAM162B	2.276666667	0.896666667	-1.344279406
53616	ADAM22	1.38	0.543333333	-1.344758803
4974	OMG	1.456666667	0.573333333	-1.345224715
2348	FOLR1	1.853333333	0.726666667	-1.350756748
1295	COL8A1	5.543333333	2.166666667	-1.355276545
7306	TYRP1	67.95	26.4	-1.363935621
7846	TUBA1A	2001.226667	768.6833333	-1.380423288
644596	SMIM10L2B	1.59	0.61	-1.382145618
11148	HHLA2	5.153333333	1.973333333	-1.384871238
287	ANK2	43.55666667	16.65666667	-1.386793833
50507	NOX4	1.216666667	0.463333333	-1.392811581
79729	SH3D21	2.22	0.843333333	-1.396384792

51012	PRELID3B	111.8333333	42.41333333	-1.398760489
284273	ZADH2	1.636666667	0.62	-1.400420403
8744	TNFSF9	5.863333333	2.22	-1.4011614
22875	ENPP4	1.506666667	0.57	-1.402326448
55803	ADAP2	35.81	13.52333333	-1.404911717
2596	GAP43	230.83	86.77	-1.411562503
2791	GNG11	79.79	29.61333333	-1.429961054
5163	PDK1	12.38333333	4.576666667	-1.436030585
6496	SIX3	261.3566667	96.51333333	-1.43721979
92610	TIFA	2.013333333	0.74	-1.443988873
728591	CCDC169	4.676666667	1.716666667	-1.445870672
117166	WFIKKN1	5.886666667	2.156666667	-1.448647726
317649	EIF4E3	113.0133333	41.36	-1.450184901
55753	OGDHL	12.79333333	4.67	-1.453897756
10000	AKT3	5.753333333	2.093333333	-1.458596
29942	PURG	5.136666667	1.856666667	-1.468117629
84900	RNFT2	1.96	0.706666667	-1.47175189
165215	FAM171B	79.48333333	28.59666667	-1.474805386
2778	GNAS	2704.573333	971.1533333	-1.477630013
57393	CLTRN	1.643333333	0.59	-1.477838286
3351	HTR1B	10.11333333	3.616666667	-1.483526043
6546	SLC8A1	4.196666667	1.496666667	-1.487490933
55636	CHD7	12.33	4.396666667	-1.487690736
6857	SYT1	9.94	3.54	-1.489496491
143903	LAYN	23.88333333	8.446666667	-1.49955018
80709	AKNA	5.903333333	2.076666667	-1.507260144
64744	SMAP2	58.75333333	20.65666667	-1.508063237
487	ATP2A1	1.386666667	0.486666667	-1.510615159
222223	KIAA1324L	3.66	1.283333333	-1.511947703
9936	CD302	6.623333333	2.313333333	-1.517584305
1004	CDH6	136.9533333	47.8	-1.518601857
57576	KIF17	5.836666667	2.036666667	-1.518934799
6938	TCF12	50.05	17.45666667	-1.519591965
604	BCL6	3.016666667	1.05	-1.522565964
51092	SIDT2	82.12	28.53666667	-1.524916802
5080	PAX6	55.88	19.41666667	-1.525036472
8447	DOC2B	2.02	0.7	-1.528928466
9355	LHX2	83.87333333	29.05	-1.52967403
79794	C12orf49	44.76666667	15.50333333	-1.52984646
56099	PCDHGB7	1.73	0.596666667	-1.535774951
119710	C11orf74	6.546666667	2.256666667	-1.536567191
11341	SCRG1	3.076666667	1.06	-1.537303882
3214	HOXB4	56.43333333	19.43333333	-1.538014185

6658	SOX3	338.4833333	116.44	-1.539498054
10610	ST6GALNAC2	29.47333333	10.07666667	-1.548391755
56929	FEM1C	8.123333333	2.77	-1.552185869
514	ATP5F1E	724.7266667	247.11	-1.552283585
170692	ADAMTS18	12.85333333	4.373333333	-1.555337332
57706	DENND1A	50.93	17.3	-1.557743679
140628	GATA5	11.68	3.966666667	-1.558041201
56548	CHST7	42.42	14.39333333	-1.559343878
2590	GALNT2	71.57666667	24.10333333	-1.570256683
55630	SLC39A4	21.09333333	7.1	-1.570896169
5210	PFKFB4	9.476666667	3.17	-1.579896853
55437	STRADB	101.4366667	33.79666667	-1.585626376
5727	PTCH1	30.16666667	10.05	-1.58575979
664	BNIP3	355.5433333	117.45	-1.597978692
158038	LINGO2	1.536666667	0.503333333	-1.610218201
230	ALDOC	13.71333333	4.49	-1.610791943
84210	ANKRD20A1	2.646666667	0.863333333	-1.616186909
83881	MIXL1	3.396666667	1.106666667	-1.617898905
7976	FZD3	6.88	2.24	-1.618909833
27019	DNAI1	6.896666667	2.23	-1.628855529
22915	MMRN1	15.65666667	5.046666667	-1.633374388
2719	GPC3	1389.65	447.5166667	-1.634708251
51050	PI15	3.436666667	1.103333333	-1.639141211
79094	CHAC1	10.02666667	3.216666667	-1.64020372
25841	ABTB2	6.016666667	1.923333333	-1.645355613
167359	NIM1K	9.006666667	2.853333333	-1.658344973
6092	ROBO2	3.53	1.116666667	-1.660469589
3490	IGFBP7	100.72	31.73666667	-1.666127675
84101	USP44	13.34666667	4.2	-1.66801824
5797	PTPRM	4.473333333	1.406666667	-1.669069767
7291	TWIST1	3.87	1.216666667	-1.669399603
2483	FRG1	76.06666667	23.65666667	-1.68501758
6611	SMS	582.37	179.9233333	-1.694553745
7697	ZNF138	8.42	2.596666667	-1.697159406
64065	PERP	4.303333333	1.326666667	-1.697648665
1368	CPM	1.73	0.533333333	-1.697662633
353322	ANKRD37	3.646666667	1.123333333	-1.698792242
1649	DDIT3	39.41666667	12.12	-1.701416079
441425	ANKRD20A3	2.05	0.626666667	-1.709853749
2770	GNAI1	2.443333333	0.743333333	-1.716769488
134548	SOWAHA	1.593333333	0.48	-1.730941807
138255	C9orf135	20.97	6.306666667	-1.733377273
127700	OSCP1	8.436666667	2.523333333	-1.741342302

57462	MYORG	10.43666667	3.093333333	-1.754426798
7161	TP73	3.726666667	1.096666667	-1.764760699
91351	DDX60L	1.04	0.296666667	-1.809668788
10251	SPRY3	5.64	1.606666667	-1.811624517
5352	PLOD2	3.88	1.103333333	-1.814187936
84634	KISS1R	11.13666667	3.166666667	-1.814280564
4131	MAP1B	15.93333333	4.52	-1.81765344
83592	AKR1E2	1.176666667	0.333333333	-1.819668183
23127	COLGALT2	144.1366667	40.1	-1.845763245
8637	EIF4EBP3	3.573333333	0.986666667	-1.856635825
116039	OSR2	36.32666667	10.02	-1.858146482
1411	CRYBA1	1.27	0.35	-1.85940167
56605	ERO1B	2.386666667	0.656666667	-1.861763958
3109	HLA-DMB	5.076666667	1.376666667	-1.882702255
11075	STMN2	29.39333333	7.97	-1.882837347
579	NKX3-2	4.046666667	1.096666667	-1.883608933
3084	NRG1	82.32666667	22.17333333	-1.892534146
51364	ZMYND10	2.41	0.646666667	-1.897938995
440	ASNS	86.51666667	23.12333333	-1.903628698
3491	CYR61	380.9033333	101.7766667	-1.904018066
59307	SIGIRR	1.226666667	0.326666667	-1.908852112
120071	LARGE2	11.24666667	2.99	-1.911280083
7179	TPTE	20.68	5.46	-1.921263329
26999	CYFIP2	3.306666667	0.86	-1.942969055
9743	ARHGAP32	2.66	0.69	-1.946757979
9518	GDF15	4.443333333	1.143333333	-1.958396299
85442	KNDC1	4.923333333	1.266666667	-1.958598502
91851	CHRDL1	1.533333333	0.393333333	-1.962847002
6340	SCNN1G	30.03666667	7.7	-1.963794367
3218	HOXB8	57.64666667	14.75666667	-1.965870316
136288	C7orf57	78.31666667	20.01333333	-1.968357886
402665	IGLON5	1.006666667	0.256666667	-1.971618199
55843	ARHGAP15	57.29	14.47	-1.985218416
222663	SCUBE3	19.57333333	4.94	-1.986306521
54020	SLC37A1	11.60666667	2.91	-1.995862644
6662	SOX9	2.49	0.62	-2.005805622
127003	C1orf194	1.426666667	0.353333333	-2.013546532
55259	CASC1	1.23	0.303333333	-2.019682366
92521	SPECC1	25.36333333	6.206666667	-2.03085379
79772	MCTP1	1.976666667	0.48	-2.041963293
26038	CHD5	2.973333333	0.703333333	-2.079800711
23022	PALLD	6.186666667	1.453333333	-2.08979667
23414	ZFPM2	15.93	3.74	-2.090636092

154141	MBOAT1	26.86666667	6.283333333	-2.096215315
577	ADGRB3	1.633333333	0.38	-2.103747925
6423	SFRP2	167.3766667	38.86333333	-2.106616869
1910	EDNRB	1.196666667	0.276666667	-2.112800602
4237	MFAP2	18.25333333	4.16	-2.133504512
89944	GLB1L2	11.87666667	2.7	-2.13709867
961	CD47	1.466666667	0.333333333	-2.137503524
143282	FGFBP3	78.55333333	17.72666667	-2.14775122
1021	CDK6	6.836666667	1.54	-2.150362735
221895	JAZF1	1.413333333	0.316666667	-2.158064846
50515	CHST11	20.58	4.496666667	-2.194315135
30813	VSX1	1.063333333	0.23	-2.208888157
100113407	TMEM170B	1.186666667	0.256666667	-2.20894689
10253	SPRY2	83.58666667	18	-2.215275922
2566	GABRG2	2.866666667	0.616666667	-2.216811389
83690	CRISPLD1	15.09333333	3.223333333	-2.227286163
26051	PPP1R16B	1.34	0.283333333	-2.241660755
348751	FTCDNL1	1.566666667	0.326666667	-2.261807102
51361	HOOK1	2.706666667	0.553333333	-2.290296486
9844	ELMO1	3.573333333	0.73	-2.291302131
100526761	CCDC169-SOHLH2	1.87	0.376666667	-2.311677998
1268	CNR1	1.013333333	0.203333333	-2.317190176
10472	ZBTB18	1.866666667	0.37	-2.334867151
7857	SCG2	2.533333333	0.5	-2.341036918
23671	TMEFF2	1.48	0.29	-2.351472371
58494	JAM2	3.15	0.613333333	-2.360608563
222389	BEND7	1.29	0.25	-2.367371066
18	ABAT	68.43666667	13.19	-2.375324928
10098	TSPAN5	1.32	0.25	-2.40053793
7020	TFAP2A	8.063333333	1.47	-2.455560208
285141	ERICH2	4.14	0.753333333	-2.458270496
25928	SOSTDC1	3.076666667	0.556666667	-2.466482545
93649	MYOCD	1.106666667	0.2	-2.468148836
388531	RGS9BP	1.376666667	0.246666667	-2.480544606
57369	GJD2	3.69	0.65	-2.505109193
120224	TMEM45B	3.643333333	0.636666667	-2.516648858
54842	MFSD6	5.976666667	1.026666667	-2.541373232
339479	BRINP3	5.283333333	0.896666667	-2.558804762
55203	LGI2	3.853333333	0.65	-2.567595369
5602	MAPK10	1.536666667	0.256666667	-2.5818364
9427	ECEL1	8.43	1.38	-2.610864364
1410	CRYAB	7.99	1.306666667	-2.61230435
728215	FAM155A	1.826666667	0.293333333	-2.638600464

63027	SLC22A23	22.11	3.493333333	-2.662022809
133418	EMB	44.6	7.033333333	-2.664763212
92162	TMEM88	123.09	18.76666667	-2.713469233
27123	DKK2	1.133333333	0.166666667	-2.765534746
221002	RASGEF1A	1.003333333	0.146666667	-2.774188058
258010	SVIP	9.34	1.323333333	-2.819246043
2171	FABP5	145.0633333	19.45	-2.898840845
7373	COL14A1	4.683333333	0.623333333	-2.909459955
2591	GALNT3	1.723333333	0.226666667	-2.926557629
79971	WLS	4.68	0.603333333	-2.955481333
1026	CDKN1A	96.72333333	12.19666667	-2.987377049
2911	GRM1	9.696666667	1.213333333	-2.998512941
55760	DHX32	10.26333333	1.253333333	-3.0336573
5325	PLAGL1	17.17666667	2.083333333	-3.043484498
760	CA2	2.323333333	0.28	-3.052697423
256691	MAMDC2	27.84	3.29	-3.080999722
8631	SKAP1	1.643333333	0.193333333	-3.087462841
3352	HTR1D	5.03	0.576666667	-3.124748863
23670	TMEM2	9.926666667	1.136666667	-3.12650011
10194	TSHZ1	4.626666667	0.516666667	-3.162667447
1803	DPP4	1.483333333	0.163333333	-3.182951682
28514	DLL1	3.123333333	0.333333333	-3.228049048
27022	FOXD3	1.106666667	0.116666667	-3.245756414
132720	FAM241A	3.076666667	0.316666667	-3.280331229
391059	FRRS1	2.44	0.25	-3.286881148
7980	TFPI2	146.81	14.91333333	-3.29927558
4038	LRP4	39.45666667	3.91	-3.335028568
645121	CCNI2	2.456666667	0.243333333	-3.33569625
2173	FABP7	2.626666667	0.256666667	-3.355265279
11166	SOX21	13.62666667	1.323333333	-3.364184284
100131390	SP9	2.1	0.19	-3.466318004
6572	SLC18A3	4.326666667	0.386666667	-3.484093673
167410	LIX1	11.18666667	0.983333333	-3.507955856
54836	BSPRY	1.403333333	0.123333333	-3.508223057
3060	HCRT	1.413333333	0.123333333	-3.518467089
407977	TNFSF12-TNFSF13	1.196666667	0.103333333	-3.533643723
84889	SLC7A3	173.65	14.84	-3.548619414
347735	SERINC2	77.46333333	6.426666667	-3.591371033
2628	GATM	2.773333333	0.23	-3.591915261
4664	NAB1	11.49	0.926666667	-3.632184511
64849	SLC13A3	2.516666667	0.2	-3.653442239
4897	NRCAM	1.653333333	0.13	-3.668794092
10468	FST	76.79666667	5.553333333	-3.789617793

152831	KLB	3.353333333	0.216666667	-3.952046777
7103	TSPAN8	1.536666667	0.093333333	-4.041268018
79632	FAM184A	2.59	0.146666667	-4.14233917
23639	LRRC6	1.65	0.093333333	-4.143929793
22999	RIMS1	3.9	0.213333333	-4.192292814
345895	RSPH4A	10.47666667	0.546666667	-4.260374552
10840	ALDH1L1	26.38666667	1.316666667	-4.324847054
7044	LEFTY2	15.20333333	0.71	-4.420424835
30010	NXPH1	1.126666667	0.05	-4.493988841
79097	TRIM48	4.223333333	0.183333333	-4.525841096
80034	CSRNP3	1.913333333	0.076666667	-4.641344971
56944	OLFML3	1.036666667	0.04	-4.695808269
3912	LAMB1	25.6	0.983333333	-4.702319451
5592	PRKG1	1.503333333	0.056666667	-4.729520782
143662	MUC15	5.88	0.206666667	-4.830438535
8433	UTF1	3.843333333	0.133333333	-4.849248703
4772	NFATC1	6.263333333	0.213333333	-4.875749351
375061	FAM89A	1.086666667	0.036666667	-4.889296536
4857	NOVA1	56.15666667	1.846666667	-4.926461922
60675	PROK2	10.47666667	0.33	-4.988569937
5577	PRKAR2B	1.603333333	0.05	-5.003002488
114088	TRIM9	4.94	0.136666667	-5.175777728
63973	NEUROG2	1.446666667	0.04	-5.176588732
64123	ADGRL4	2.46	0.063333333	-5.279549493
441581	FRG2B	7.056666667	0.133333333	-5.725877459
799	CALCR	82.88333333	1.54	-5.750079769
6335	SCN9A	2.556666667	0.043333333	-5.882643049
5156	PDGFRA	38.99666667	0.623333333	-5.967203137
4990	SIX6	2.09	0.03	-6.122396631
448831	FRG2	6.876666667	0.09	-6.255640604
2824	GPM6B	8.8	0.11	-6.321928095
114907	FBXO32	3.08	0.036666667	-6.392317423
26002	MOXD1	21.27666667	0.25	-6.411200242
10215	OLIG2	1.14	0.013333333	-6.417852515
146713	RBFOX3	2.223333333	0.023333333	-6.574188029
100288801	FRG2C	19.13666667	0.2	-6.580195745
8492	PRSS12	1.403333333	0.01	-7.132713922
1286	COL4A4	3.753333333	0.026666667	-7.136991112
116448	OLIG1	1.58	0.01	-7.303780748
3075	CFH	1.766666667	0.01	-7.464886049
220382	FAM181B	74.42666667	0.413333333	-7.492370099
170825	GSX2	44.83333333	0.243333333	-7.525493993
100137049	PLA2G4B	2.02	0.01	-7.658211483

1285	COL4A3	2.373333333	0.01	-7.89077093
5375	PMP2	6.956666667	0.02	-8.442252384
8363	HIST1H4J	3.85	0.01	-8.588714636
794	CALB2	154.3466667	0.283333333	-9.08945827

Table S2f

Gene ID	Gene Symbol	hdctrl Expression	hdecadkd Expression	log2FoldChange (hdecadkd/hdctrl)
5375	PMP2	0.016666667	6.956666667	8.70528679
8293	SERF1A	0.01	2.03	7.665335917
2846	LPAR4	0.03	1.606666667	5.742964335
794	CALB2	3.386666667	154.3466667	5.51016452
114088	TRIM9	0.156666667	4.94	4.978740881
2824	GPM6B	0.28	8.8	4.974004791
1285	COL4A3	0.076666667	2.373333333	4.952171475
7044	LEFTY2	0.513333333	15.20333333	4.888347914
93649	MYOCD	0.04	1.106666667	4.790076931
23670	TMEM2	0.4	9.926666667	4.633237443
4990	SIX6	0.086666667	2.09	4.591881915
146713	RBFOX3	0.1	2.223333333	4.474652356
3075	CFH	0.08	1.766666667	4.464886049
2171	FABP5	6.696666667	145.0633333	4.437095937
5592	PRKG1	0.083333333	1.503333333	4.173127433
961	CD47	0.083333333	1.466666667	4.137503524
5796	PTPRK	0.33	5.74	4.120512807
220382	FAM181B	4.3	74.42666667	4.113411059
79971	WLS	0.276666667	4.68	4.080287789
1286	COL4A4	0.223333333	3.753333333	4.070901922
23639	LRRC6	0.11	1.65	3.906890596
388436	LOC388436	0.613333333	8.69	3.824612911
117154	DACH2	0.22	3.01	3.774188058
4071	TM4SF1	0.113333333	1.353333333	3.577873076
79097	TRIM48	0.37	4.223333333	3.512784943
1026	CDKN1A	8.483333333	96.72333333	3.511160809
27022	FOXD3	0.1	1.106666667	3.468148836
11010	GLIPR1	0.686666667	7.373333333	3.424635143
63973	NEUROG2	0.143333333	1.446666667	3.335286478
347902	AMIGO2	0.963333333	8.963333333	3.217928357
799	CALCR	9.403333333	82.88333333	3.13983786
94240	EPSTI1	0.136666667	1.196666667	3.130288029
64123	ADGRL4	0.3	2.46	3.03562391
4916	NTRK3	0.173333333	1.406666667	3.020659471
120224	TMEM45B	0.453333333	3.643333333	3.006614844
258010	SVIP	1.176666667	9.34	2.988716867
2346	FOLH1	2.77	21.23333333	2.93837299
1268	CNR1	0.136666667	1.013333333	2.890375509
1803	DPP4	0.203333333	1.483333333	2.866924188
2628	GATM	0.396666667	2.773333333	2.805621955

221935	SDK1	0.326666667	2.273333333	2.798918085
64065	PERP	0.623333333	4.303333333	2.787378825
63027	SLC22A23	3.27	22.11	2.757336484
5577	PRKAR2B	0.243333333	1.603333333	2.720068525
6588	SLN	0.69	4.54	2.718024031
219595	FOLH1B	0.5	3.23	2.691534165
80243	PREX2	0.266666667	1.616666667	2.599912842
347735	SERINC2	12.90666667	77.46333333	2.585397132
60675	PROK2	1.75	10.47666667	2.581752944
100131390	SP9	0.36	2.1	2.544320516
11341	SCRG1	0.543333333	3.076666667	2.501458683
116448	OLIG1	0.29	1.58	2.445799753
4857	NOVA1	10.37	56.15666667	2.437041409
10610	ST6GALNAC2	5.443333333	29.47333333	2.436847943
26038	CHD5	0.553333333	2.973333333	2.425860469
1410	CRYAB	1.5	7.99	2.413233002
54842	MFSD6	1.126666667	5.976666667	2.407280337
11023	VAX1	0.306666667	1.626666667	2.407175382
2909	ARHGAP35	13.88666667	72.91666667	2.392548272
375061	FAM89A	0.21	1.086666667	2.371448231
56660	KCNK12	0.673333333	3.466666667	2.36415633
1800	DPEP1	2.963333333	15.21333333	2.360043467
5457	POU4F1	0.343333333	1.736666667	2.338639035
127003	C1orf194	0.296666667	1.426666667	2.265733555
51196	PLCE1	1.49	7.036666667	2.239579842
51232	CRIM1	5.103333333	23.83333333	2.223468959
345895	RSPH4A	2.273333333	10.47666667	2.204298628
94235	GNG8	0.543333333	2.486666667	2.194303666
57094	CPA6	0.78	3.54	2.182203331
59277	NTN4	1.873333333	8.47	2.17675434
347731	LRRTM3	0.32	1.446666667	2.176588732
28514	DLL1	0.696666667	3.123333333	2.164546106
6572	SLC18A3	0.986666667	4.326666667	2.132621302
221895	JAZF1	0.33	1.413333333	2.098563834
1740	DLG2	0.896666667	3.836666667	2.097209756
10215	OLIG2	0.266666667	1.14	2.09592442
22822	PHLDA1	5.543333333	23.66	2.093624406
7980	TFPI2	34.73666667	146.81	2.079419016
124925	SEZ6	0.4	1.673333333	2.064652958
3592	IL12A	0.273333333	1.143333333	2.064512762
51809	GALNT7	0.293333333	1.226666667	2.064130337
6423	SFRP2	40.65666667	167.3766667	2.041534579
6474	SHOX2	0.39	1.596666667	2.033517126

79400	NOX5	0.686666667	2.806666667	2.031175896
222663	SCUBE3	4.89	19.57333333	2.000983097
55203	LGI2	0.963333333	3.853333333	2
4664	NAB1	2.896666667	11.49	1.987913217
8862	APLN	0.86	3.39	1.978876708
55567	DNAH3	0.416666667	1.623333333	1.961993677
11166	SOX21	3.506666667	13.62666667	1.958260492
5352	PLOD2	1.003333333	3.88	1.951255666
84532	ACSS1	0.9	3.48	1.9510904
6546	SLC8A1	1.086666667	4.196666667	1.949334413
7306	TYRP1	17.65333333	67.95	1.94453293
2334	AFF2	1.62	6.103333333	1.913603572
3955	LFNG	5.47	20.60666667	1.913498415
55859	BEX1	1.206666667	4.506666667	1.901033549
54836	BSPRY	0.376666667	1.403333333	1.897497461
8631	SKAP1	0.443333333	1.643333333	1.890161401
5327	PLAT	3.82	14	1.873782284
132720	FAM241A	0.846666667	3.076666667	1.861502151
4897	NRCAM	0.463333333	1.653333333	1.835255238
50507	NOX4	0.346666667	1.216666667	1.811312936
23213	SULF1	3.16	11.08	1.809961418
26999	CYFIP2	0.943333333	3.306666667	1.809538068
8549	LGR5	0.583333333	2.01	1.78480308
5228	PGF	8.416666667	28.35333333	1.752197467
1956	EGFR	0.99	3.33	1.750021747
30813	VSX1	0.32	1.063333333	1.732450113
55170	PRMT6	0.45	1.48	1.717600269
29970	SCHIP1	24.82666667	81.59	1.716501768
56892	TCIM	1.463333333	4.806666667	1.71577832
5602	MAPK10	0.47	1.536666667	1.709071588
4772	NFATC1	1.93	6.263333333	1.698329813
100505385	IQCJ-SCHIP1	4.496666667	14.55	1.694091306
7477	WNT7B	0.346666667	1.106666667	1.674599713
387885	CFAP73	0.48	1.526666667	1.669278787
55638	SYBU	1.903333333	6.006666667	1.65803636
146	ADRA1D	0.393333333	1.24	1.656515762
51364	ZMYND10	0.766666667	2.41	1.652361786
22801	ITGA11	1.033333333	3.243333333	1.65017159
55824	PAG1	6.143333333	19.24	1.647015229
2247	FGF2	4.12	12.86	1.6421744
2487	FRZB	49.33	153.85	1.640987251
8363	HIST1H4J	1.246666667	3.85	1.626782676
84879	MFSD2A	1.53	4.666666667	1.608860768

2791	GNG11	26.47666667	79.79	1.591486444
84978	FRMD5	0.606666667	1.826666667	1.590237443
133418	EMB	14.91	44.6	1.580763453
11098	PRSS23	4.07	12.14	1.576667722
3215	HOXB5	3.216666667	9.593333333	1.576465745
7161	TP73	1.25	3.726666667	1.575957687
9659	PDE4DIP	4.476666667	13.29666667	1.570567819
1910	EDNRB	0.403333333	1.196666667	1.568976797
92521	SPECC1	8.563333333	25.36333333	1.566499972
57522	SRGAP1	3.553333333	10.52333333	1.566346823
84072	HORMAD1	0.4	1.183333333	1.564784619
5156	PDGFRA	13.24333333	38.99666667	1.558084519
55796	MBNL3	0.856666667	2.516666667	1.554708285
448831	FRG2	2.346666667	6.876666667	1.551096487
9249	DHRS3	0.99	2.9	1.55055247
8788	DLK1	3.51	10.25666667	1.547019007
50651	SLC45A1	2.423333333	7.036666667	1.537899309
18	ABAT	23.70333333	68.43666667	1.529679537
6335	SCN9A	0.886666667	2.556666667	1.527800332
143903	LAYN	8.286666667	23.88333333	1.527140408
100288801	FRG2C	6.66	19.13666667	1.522745472
113263	GLCCI1	2.323333333	6.656666667	1.518603772
9619	ABCG1	0.996666667	2.85	1.515778936
4753	NELL2	66.19666667	188.8933333	1.512741308
56944	OLFML3	0.363333333	1.036666667	1.512586445
4135	MAP6	2.046666667	5.83	1.510219729
2591	GALNT3	0.606666667	1.723333333	1.50622583
89765	RSPH1	1.39	3.943333333	1.504330785
84210	ANKRD20A1	0.933333333	2.646666667	1.50371218
100287482	SMKR1	0.92	2.603333333	1.500654281
84889	SLC7A3	61.87	173.65	1.488870472
143941	TTC36	0.756666667	2.123333333	1.488601075
10439	OLFM1	4.873333333	13.66	1.486976673
1368	CPM	0.633333333	1.73	1.44973512
28951	TRIB2	2.273333333	6.203333333	1.448234411
2736	GLI2	2.346666667	6.386666667	1.444450227
100113407	TMEM170B	0.446666667	1.186666667	1.409644241
84698	CAPS2	0.776666667	2.053333333	1.402600396
166929	SGMS2	0.696666667	1.833333333	1.395928676
26002	MOXD1	8.086666667	21.27666667	1.395655097
83698	CALN1	0.523333333	1.376666667	1.395377223
26115	TANC2	6.943333333	18.19333333	1.389709555
4643	MYO1E	0.523333333	1.366666667	1.384859351

8531	YBX3	14.27333333	37.25	1.383918131
2104	ESRRG	1.713333333	4.42	1.367240511
389799	CFAP77	0.526666667	1.356666667	1.365104236
143282	FGFBP3	30.59	78.55333333	1.360612389
112937	GLB1L3	0.43	1.103333333	1.359460151
23705	CADM1	13.9	35.66	1.35922182
170692	ADAMTS18	5.013333333	12.85333333	1.358300485
283417	DPY19L2	0.726666667	1.85	1.348159636
5080	PAX6	21.95	55.88	1.348111081
3306	HSPA2	27.18666667	69.15	1.346829977
1411	CRYBA1	0.5	1.27	1.344828497
9464	HAND2	8.223333333	20.86666667	1.343404942
28996	HIPK2	0.883333333	2.236666667	1.340320407
127700	OSCP1	3.346666667	8.436666667	1.333948238
9844	ELMO1	1.426666667	3.573333333	1.324622204
89944	GLB1L2	4.766666667	11.87666667	1.317077336
56126	PCDHB10	0.876666667	2.18	1.314227836
114907	FBXO32	1.24	3.08	1.31259023
3352	HTR1D	2.03	5.03	1.309078673
144100	PLEKHA7	0.916666667	2.263333333	1.303979956
7042	TGFB2	6.013333333	14.80666667	1.300010054
7373	COL14A1	1.906666667	4.683333333	1.296483078
577	ADGRB3	0.666666667	1.633333333	1.292781749
23418	CRB1	6.68	16.26333333	1.283702975
55584	CHRNA9	6.193333333	15.07333333	1.28321049
79983	POF1B	30.07333333	73.14	1.282175841
102724428	SIK1B	2.556666667	6.216666667	1.281877148
3218	HOXB8	23.81666667	57.64666667	1.275265678
2195	FAT1	7.866666667	18.95666667	1.268880945
441581	FRG2B	2.94	7.056666667	1.263170708
55806	HR	0.92	2.206666667	1.26216295
8643	PTCH2	2.656666667	6.333333333	1.253347789
8787	RGS9	1.313333333	3.13	1.252929528
64744	SMAP2	24.71	58.75333333	1.249575694
728591	CCDC169	1.976666667	4.676666667	1.242410999
152007	GLIPR2	7.496666667	17.71	1.240243052
116931	MED12L	3.27	7.723333333	1.239933003
160335	TMTC2	15.83333333	37.22666667	1.233371428
23440	OTP	7.663333333	17.99	1.231151221
1036	CDO1	10.64333333	24.98333333	1.231015925
56122	PCDHB14	0.74	1.736666667	1.230723696
59307	SIGIRR	0.523333333	1.226666667	1.228941207
3216	HOXB6	7.016666667	16.39	1.223958122

348013	TMEM255B	0.806666667	1.883333333	1.22324382
3351	HTR1B	4.34	10.11333333	1.220491637
1278	COL1A2	1125.693333	2621.17	1.21939707
341019	DCDC1	0.56	1.303333333	1.218707375
136259	KLF14	0.436666667	1.013333333	1.214504512
157574	FBXO16	4.006666667	9.28	1.211722315
256691	MAMDC2	12.09666667	27.84	1.202549655
5756	TWF1	40.29333333	92.48	1.198600238
8637	EIF4EBP3	1.56	3.573333333	1.195724471
3202	HOXA5	0.696666667	1.593333333	1.193507676
54453	RIN2	0.83	1.896666667	1.19228291
122060	SLAIN1	1.69	3.833333333	1.181576209
10472	ZBTB18	0.823333333	1.866666667	1.180915785
23111	SPART	8.66	19.61	1.179150605
79794	C12orf49	19.80333333	44.76666667	1.176681611
140628	GATA5	5.186666667	11.68	1.171160715
2353	FOS	1.876666667	4.223333333	1.170209697
57211	ADGRG6	22.04333333	49.53333333	1.168057309
30819	KCNIP2	0.64	1.436666667	1.166581558
157638	FAM84B	1.39	3.113333333	1.163375166
3214	HOXB4	25.22666667	56.43333333	1.161597979
284119	CAVIN1	104.4733333	232.6033333	1.154737028
5547	PRCP	64.73333333	143.4766667	1.148235433
124936	CYB5D2	6.68	14.79	1.146702045
170302	ARX	0.59	1.303333333	1.143419247
114804	RNF157	1.046666667	2.31	1.142090793
79632	FAM184A	1.176666667	2.59	1.138246415
728215	FAM155A	0.83	1.826666667	1.138030151
6833	ABCC8	0.616666667	1.356666667	1.137503524
51286	CEND1	1.713333333	3.76	1.133926803
8701	DNAH11	1.3	2.85	1.132450296
25841	ABTB2	2.746666667	6.016666667	1.131282595
727936	GXYLT2	2.12	4.616666667	1.122787306
8437	RASAL1	0.56	1.216666667	1.119435231
23283	CSTF2T	1.546666667	3.36	1.119298928
5530	PPP3CA	4.17	9.046666667	1.117338931
51375	SNX7	30.57666667	66.26	1.115707064
9379	NRXN2	0.66	1.43	1.115477217
6611	SMS	268.9333333	582.37	1.114687456
9518	GDF15	2.06	4.443333333	1.108998037
8892	EIF2B2	41.50333333	89.19666667	1.103762586
6092	ROBO2	1.646666667	3.53	1.100119642
645121	CCNI2	1.146666667	2.456666667	1.099256054

7103	TSPAN8	0.72	1.536666667	1.093735438
100526761	CCDC169-SOHLH2	0.876666667	1.87	1.092937971
101927322	LOC101927322	0.756666667	1.613333333	1.09231475
25789	TMEM59L	34.27666667	73.07333333	1.092118202
56112	PCDHGA3	3.356666667	7.146666667	1.090241222
79822	ARHGAP28	0.653333333	1.39	1.089193729
27092	CACNG4	0.553333333	1.176666667	1.088484942
219670	ENKUR	6.01	12.78	1.08845094
2621	GAS6	5.95	12.62666667	1.085512257
3663	IRF5	2.44	5.17	1.083283133
4254	KITLG	0.61	1.29	1.080489918
55220	KLHDC8A	1.453333333	3.073333333	1.080438616
6840	SVIL	5.743333333	12.12333333	1.077826224
23002	DAAM1	2.32	4.886666667	1.074725892
55437	STRADB	48.16333333	101.4366667	1.074572095
284076	TTLL6	4.073333333	8.566666667	1.072524074
58191	CXCL16	5.816666667	12.21333333	1.070192467
230	ALDOC	6.533333333	13.71333333	1.06968814
51655	RASD1	1.93	4.05	1.069321061
9734	HDAC9	38.74	81.28	1.069076448
339184	CCDC144NL	0.49	1.026666667	1.067114196
26053	AUTS2	4.606666667	9.646666667	1.066307306
2911	GRM1	4.636666667	9.696666667	1.064400877
56987	BBX	5.286666667	11.04333333	1.062745432
3198	HOXA1	1.216666667	2.54	1.061894534
6282	S100A11	102.8066667	214.57	1.061514559
638	BIK	1.376666667	2.87	1.059871456
6692	SPINT1	10.70333333	22.27	1.057041394
10186	LHFPL6	17.04666667	35.26	1.048542815
25978	CHMP2B	13.35333333	27.61333333	1.048165132
23007	PLCH1	3.723333333	7.696666667	1.047638987
3959	LGALS3BP	15.04	31.07666667	1.047027199
27344	PCSK1N	44.08	90.52333333	1.038165486
4038	LRP4	19.21666667	39.45666667	1.037910974
3201	HOXA4	4.23	8.683333333	1.037591303
55815	TSNAXIP1	0.68	1.393333333	1.03493379
4086	SMAD1	1.04	2.13	1.034269902
53335	BCL11A	1.52	3.11	1.032843257
10194	TSHZ1	2.263333333	4.626666667	1.031524088
84709	MGARP	1.73	3.533333333	1.030257821
11043	MID2	1.036666667	2.116666667	1.029842012
308	ANXA5	239.9933333	489.2233333	1.027498884
54843	SYTL2	22.91666667	46.48666667	1.020419768

83891	SNX25	5.533333333	11.22333333	1.02028048
89801	PPP1R3F	0.943333333	1.91	1.017733086
23345	SYNE1	1.35	2.73	1.015941544
860	RUNX2	0.903333333	1.826666667	1.015883042
64072	CDH23	6.913333333	13.96666667	1.01453435
653333	FAM86B2	0.566666667	1.143333333	1.01267383
85016	C11orf70	4.47	9.006666667	1.010718437
3199	HOXA2	1.476666667	2.966666667	1.006498637
5507	PPP1R3C	1.876666667	3.766666667	1.005115945
79739	TTLL7	1.84	3.683333333	1.001306198
514	ATP5F1E	362.23	724.7266667	1.000530944
80328	ULBP2	0.93	1.86	1
6752	SSTR2	0.546666667	1.093333333	1
167359	NIM1K	4.503333333	9.006666667	1
10635	RAD51AP1	6.38	3.186666667	-1.001508307
64115	VSIR	2.066666667	1.03	-1.004661377
8835	SOCS2	9.67	4.81	-1.007478996
5757	PTMA	1898.693333	942.55	-1.010365851
4584	MUC3A	1.206666667	0.596666667	-1.01603011
84517	ACTRT3	1.496666667	0.74	-1.016155768
90427	BMF	11.62666667	5.746666667	-1.016640266
9170	LPAR2	4.233333333	2.09	-1.018291149
51816	ADA2	10.13333333	4.993333333	-1.0210337
5026	P2RX5	1.88	0.923333333	-1.025809186
4255	MGMT	13.5	6.626666667	-1.026604151
28	ABO	1.616666667	0.793333333	-1.027023174
51083	GAL	12.69	6.213333333	-1.03025271
93349	SP140L	2.786666667	1.363333333	-1.031402099
111	ADCY5	2.78	1.36	-1.031478231
1366	CLDN7	13.16	6.426666667	-1.034016938
27132	CPNE7	2.123333333	1.036666667	-1.034378792
200916	RPL22L1	4.116666667	2.006666667	-1.03667565
487	ATP2A1	2.846666667	1.386666667	-1.037652541
6623	SNCG	1.02	0.496666667	-1.038219322
113	ADCY7	13.00333333	6.326666667	-1.039364006
6683	SPAST	8.073333333	3.92	-1.042310805
11339	OIP5	10.36666667	5.02	-1.04619281
141	ADPRH	2.21	1.07	-1.046435573
200844	C3orf67	1.86	0.9	-1.047305715
23129	PLXND1	44.72666667	21.64	-1.047434744
554282	FAM72C	4.243333333	2.046666667	-1.051921859
999	CDH1	2.673333333	1.286666667	-1.055001389
338645	LUZP2	11.30666667	5.433333333	-1.057264205

55032	SLC35A5	6.58	3.153333333	-1.061209901
54762	GRAMD1C	2.916666667	1.393333333	-1.065780075
5052	PRDX1	455.1066667	216.9966667	-1.068531839
253152	EPHX4	2.68	1.27	-1.077404504
345630	FBLL1	4.023333333	1.903333333	-1.079863025
1465	CSRP1	42.26333333	19.99	-1.080128086
80055	PGAP1	41.50333333	19.62333333	-1.080657084
2825	GPR1	12.37666667	5.846666667	-1.081936567
57615	ZNF492	2.86	1.35	-1.08305574
5730	PTGDS	22.79333333	10.75333333	-1.083827984
51087	YBX2	26.89666667	12.62	-1.091715479
7511	XPNPEP1	29.66666667	13.89333333	-1.094450058
2774	GNAL	1.063333333	0.496666667	-1.098244093
5798	PTPRN	1.82	0.85	-1.098403704
126433	FBXO27	169.36	78.65666667	-1.106452221
90649	ZNF486	3.83	1.776666667	-1.10817136
2199	FBLN2	9.766666667	4.53	-1.108355209
7052	TGM2	4.766666667	2.206666667	-1.111112025
253970	SFTA3	50.00666667	23.09	-1.114852268
148203	ZNF738	8.19	3.776666667	-1.116749997
6405	SEMA3F	6.25	2.88	-1.117787378
2678	GGT1	14.88	6.853333333	-1.118496763
1140	CHRNB1	13.94	6.406666667	-1.121584726
93986	FOXP2	13.72	6.296666667	-1.123620281
7275	TUB	1.403333333	0.643333333	-1.125219386
7164	TPD52L1	4.463333333	2.043333333	-1.127196982
154790	CLEC2L	1.183333333	0.54	-1.131825212
83543	AIF1L	6.22	2.836666667	-1.132717949
58477	SRPRB	8.156666667	3.713333333	-1.135264869
4638	MYLK	1.326666667	0.603333333	-1.136778733
23240	TMEM131L	7.36	3.346666667	-1.136980903
150280	HORMAD2	2.046666667	0.93	-1.137973534
3487	IGFBP4	29.37333333	13.34666667	-1.138027521
7644	ZNF91	8.716666667	3.956666667	-1.139491011
493	ATP2B4	23.00666667	10.43666667	-1.140390965
136853	SSC4D	1.17	0.53	-1.142444265
339768	ESPNL	1.55	0.7	-1.146841388
170261	ZCCHC12	19.47333333	8.783333333	-1.148659396
147741	ZNF560	1.37	0.616666667	-1.151613123
5731	PTGER1	9.023333333	4.053333333	-1.154551659
4232	MEST	30.02333333	13.44	-1.159551023
1013	CDH15	1.176666667	0.526666667	-1.159743625
9262	STK17B	7.723333333	3.45	-1.162627276

131540	ZDHHC19	4.573333333	2.036666667	-1.167036196
3674	ITGA2B	2.223333333	0.99	-1.16722383
1749	DLX5	1.38	0.613333333	-1.169925001
132430	PABPC4L	1.163333333	0.516666667	-1.170958821
57583	TMEM181	8.183333333	3.633333333	-1.17139489
286319	TUSC1	2.146666667	0.95	-1.176098769
3750	KCND1	2.283333333	1.01	-1.176786194
83401	ELOVL3	1.17	0.516666667	-1.179202815
51127	TRIM17	2.136666667	0.943333333	-1.179522304
64714	PDIA2	1.05	0.463333333	-1.180266946
5837	PYGM	22.63333333	9.983333333	-1.180855571
221662	RBM24	7.686666667	3.376666667	-1.186758339
8076	MFAP5	11.60333333	5.09	-1.188801752
84958	SYTL1	3.15	1.38	-1.190683562
133022	TRAM1L1	2.93	1.283333333	-1.19100472
389383	CLPSL2	1.34	0.586666667	-1.191620073
79772	MCTP1	4.516666667	1.976666667	-1.192188842
51062	ATL1	5.03	2.2	-1.193054876
766	CA7	1.87	0.816666667	-1.195219022
11173	ADAMTS7	15.31333333	6.686666667	-1.195429251
401027	C2orf66	1.123333333	0.49	-1.196932436
57514	ARHGAP31	1.59	0.693333333	-1.197405738
7137	TNNI3	1.216666667	0.53	-1.198869698
6659	SOX4	5.703333333	2.48	-1.201465233
1028	CDKN1C	9.796666667	4.25	-1.204828112
3049	HBQ1	3.34	1.446666667	-1.207115561
745	MYRF	3.816666667	1.65	-1.209847168
163732	CITED4	30.74333333	13.27666667	-1.211380618
116844	LRG1	3.416666667	1.47	-1.216773349
79785	RERGL	1.61	0.69	-1.222392421
2157	F8	2.326666667	0.996666667	-1.223081552
140576	S100A16	5.84	2.5	-1.224040274
4162	MCAM	13.61666667	5.82	-1.226282519
9891	NUAK1	3.656666667	1.556666667	-1.232069071
338773	TMEM119	2.28	0.97	-1.232977172
10681	GNB5	21.02	8.93	-1.235030589
7643	ZNF90	1.75	0.743333333	-1.235273713
10893	MMP24	1.516666667	0.643333333	-1.237265698
6442	SGCA	2.216666667	0.94	-1.237659178
3486	IGFBP3	1.016666667	0.43	-1.241438177
10003	NAALAD2	1.293333333	0.546666667	-1.242360838
220323	OAF	1.29	0.543333333	-1.247461602
257407	C2orf72	3.04	1.28	-1.247927513

4692	NDN	4.903333333	2.06	-1.251118503
11131	CAPN11	1.603333333	0.67	-1.258841393
136227	COL26A1	15.04	6.266666667	-1.263034406
54509	RHOF	4.94	2.056666667	-1.264203053
26609	VCX	7.233333333	3.01	-1.26489715
10220	GDF11	20.57666667	8.546666667	-1.267575529
106821730	BUB1B-PAK6	2.076666667	0.86	-1.271861098
84812	PLCD4	11.31	4.683333333	-1.2719913
5333	PLCD1	40.20666667	16.57	-1.278861132
8187	ZNF239	2.31	0.95	-1.281893433
760	CA2	5.656666667	2.323333333	-1.283756004
10409	BASP1	7.936666667	3.24	-1.292539402
6935	ZEB1	1.81	0.736666667	-1.296905828
388581	C1QTNF12	2.163333333	0.88	-1.297680549
4843	NOS2	1.476666667	0.6	-1.299309792
5292	PIM1	30.76666667	12.49333333	-1.300209695
91851	CHRDL1	3.776666667	1.533333333	-1.300442095
57165	GJC2	1.856666667	0.75	-1.307752326
5176	SERPINF1	78.34666667	31.63333333	-1.308426309
10826	FAXDC2	10.7	4.3	-1.315202232
54587	MXRA8	3.443333333	1.383333333	-1.315657013
8693	GALNT4	9.776666667	3.926666667	-1.316037532
51441	YTHDF2	77.95333333	31.28	-1.317370202
54549	SDK2	2.363333333	0.946666667	-1.319894698
9456	HOMER1	7.59	3.04	-1.320028562
91522	COL23A1	2.6	1.03	-1.335867286
100316904	SAP25	1.38	0.546666667	-1.335934953
9754	STARD8	1.63	0.643333333	-1.341233618
4914	NTRK1	1.166666667	0.46	-1.342686655
8645	KCNK5	1.386666667	0.543333333	-1.351711564
27122	DKK3	9.166666667	3.59	-1.352413369
2134	EXTL1	2.393333333	0.936666667	-1.353413714
10062	NR1H3	9.313333333	3.64	-1.355359164
440585	FAM183A	1.533333333	0.596666667	-1.361674274
7568	ZNF20	1.89	0.733333333	-1.365845211
7134	TNNC1	12.36333333	4.793333333	-1.366966592
254295	PHYHD1	10.12333333	3.92	-1.368758848
117854	TRIM6	10.05	3.866666667	-1.378033197
151473	SLC16A14	2.063333333	0.793333333	-1.378977836
7087	ICAM5	20.93666667	8.04	-1.380764362
100129543	ZNF730	2.253333333	0.863333333	-1.384071149
51334	PRR16	16.27333333	6.196666667	-1.392945525
85300	ATCAY	23.58333333	8.97	-1.394587757

3563	IL3RA	3.42	1.3	-1.395484702
497661	C18orf32	20.50666667	7.766666667	-1.400725548
1132	CHRM4	5.536666667	2.093333333	-1.403215609
1187	CLCNKA	1.043333333	0.393333333	-1.407375798
9509	ADAMTS2	1.42	0.533333333	-1.412781525
7305	TYROBP	30.99333333	11.63666667	-1.413280069
126969	SLC44A3	1.12	0.416666667	-1.426533138
3038	HAS3	11.59666667	4.296666667	-1.432420415
284422	SMIM24	3.383333333	1.25	-1.436517227
84734	FAM167B	1.273333333	0.47	-1.437877476
100302652	GPR75-ASB3	2.973333333	1.093333333	-1.443347895
1136	CHRNA3	3.116666667	1.143333333	-1.446757789
11217	AKAP2	17.55666667	6.36	-1.464920288
388610	TRNP1	5.196666667	1.88	-1.46685386
2113	ETS1	8.56	3.08	-1.474680446
151176	ERFE	5.996666667	2.14	-1.486549984
8436	CAVIN2	1.62	0.576666667	-1.490184276
102724594	U2AF1L5	2.66	0.946666667	-1.490497817
168620	BHLHA15	1.716666667	0.61	-1.492728784
6691	SPINK2	3.94	1.4	-1.492768803
3039	HBA1	6.846666667	2.42	-1.500394728
8784	TNFRSF18	1.693333333	0.596666667	-1.50486891
1464	CSPG4	2.983333333	1.05	-1.506535854
51029	DESI2	15.97	5.603333333	-1.511007089
9331	B4GALT6	3.566666667	1.243333333	-1.520363261
58189	WFDC1	7.976666667	2.75	-1.536354372
83690	CRISPLD1	43.89	15.09333333	-1.539980813
1592	CYP26A1	17.87333333	6.133333333	-1.543065453
342897	NCCRP1	15.53666667	5.32	-1.546178861
22865	SLITRK3	2.226666667	0.76	-1.550814278
3383	ICAM1	5.36	1.82	-1.55829455
3485	IGFBP2	36.95	12.54333333	-1.558653576
8174	MADCAM1	1.826666667	0.616666667	-1.566650622
51063	CALHM2	1.303333333	0.436666667	-1.577601796
79729	SH3D21	6.636666667	2.22	-1.579899139
586	BCAT1	16.04666667	5.34	-1.587361994
169166	SNX31	1.983333333	0.656666667	-1.594694039
80303	EFHD1	12.63333333	4.086666667	-1.628238869
3281	HSBP1	36.19333333	11.64	-1.636632925
27439	TMEM121B	1.53	0.49	-1.642677999
1000	CDH2	6.823333333	2.18	-1.646148562
4118	MAL	6.063333333	1.936666667	-1.646535474
9535	GMFG	1.763333333	0.56	-1.654806489

50861	STMN3	44.12666667	13.87333333	-1.669336306
4900	NRGN	14.19333333	4.46	-1.670097834
57216	VANGL2	3.053333333	0.95	-1.684385679
146206	CARMIL2	1.346666667	0.406666667	-1.727474145
2695	GIP	7.436666667	2.243333333	-1.729012104
4615	MYD88	1.216666667	0.366666667	-1.73039294
978	CDA	2.323333333	0.7	-1.730769328
1158	CKM	11.31666667	3.396666667	-1.736257523
54511	HMGCLL1	2.833333333	0.85	-1.736965594
28984	RGCC	9.706666667	2.883333333	-1.751238318
10481	HOXB13	5.996666667	1.766666667	-1.763130922
4846	NOS3	1.416666667	0.416666667	-1.765534746
6480	ST6GAL1	11.63666667	3.35	-1.796444855
90139	TSPAN18	3.016666667	0.866666667	-1.799406169
79999	LOC79999	17.21666667	4.936666667	-1.802196708
23769	FLRT1	1.896666667	0.543333333	-1.803556688
440836	ODF3B	14.49	4.143333333	-1.806193799
8362	HIST1H4K	3.496666667	0.996666667	-1.810797288
115727	RASGRP4	3.776666667	1.07	-1.819502659
10752	CHL1	3.016666667	0.853333333	-1.821773982
3816	KLK1	2.04	0.576666667	-1.822759615
29944	PNMA3	1.126666667	0.316666667	-1.831023828
10202	DHRS2	1.673333333	0.466666667	-1.842260537
79727	LIN28A	1.22	0.336666667	-1.857488356
2837	UTS2R	2.04	0.56	-1.86507042
1299	COL9A3	3.9	1.07	-1.865863327
653	BMP5	3.873333333	1.046666667	-1.887773605
10581	IFITM2	2.73	0.736666667	-1.889817082
9886	RHOBTB1	2.33	0.623333333	-1.902254185
190	NR0B1	1.1	0.293333333	-1.906890596
54596	L1TD1	9.546666667	2.53	-1.915859702
401	PHOX2A	1.17	0.31	-1.916168409
30832	ZNF354C	2.006666667	0.526666667	-1.929838929
2047	EPHB1	1.21	0.316666667	-1.93397013
140738	TMEM37	1.913333333	0.5	-1.936088236
144568	A2ML1	1.316666667	0.336666667	-1.96749736
4651	MYO10	46.96333333	11.98333333	-1.970505541
4199	ME1	1.15	0.293333333	-1.971020933
51700	CYB5R2	1.013333333	0.256666667	-1.981140973
10267	RAMP1	8.866666667	2.233333333	-1.989193245
9699	RIMS2	2.076666667	0.52	-1.997686134
163782	KANK4	7.15	1.773333333	-2.011479497
10417	SPON2	1.17	0.29	-2.012383724

102723360	LOC102723360	1.01	0.25	-2.014355293
63827	BCAN	5.023333333	1.243333333	-2.014431881
3976	LIF	1.273333333	0.313333333	-2.022839976
8537	BCAS1	1.94	0.476666667	-2.025004006
8633	UNC5C	1.41	0.343333333	-2.038013326
56164	STK31	4.326666667	1.036666667	-2.061303898
91461	PKDCC	5.92	1.416666667	-2.063096835
1382	CRABP2	1559.673333	371.9	-2.068257242
23180	RFTN1	2.533333333	0.593333333	-2.094122177
55959	SULF2	10.78333333	2.496666667	-2.110728088
2122	MECOM	1.04	0.236666667	-2.135655099
2301	FOXE3	2.713333333	0.613333333	-2.145323028
23025	UNC13A	2.693333333	0.6	-2.166358386
9060	PAPSS2	8.8	1.936666667	-2.183927861
11013	TMSB15A	1.443333333	0.313333333	-2.203634363
84894	LINGO1	9.28	2.006666667	-2.209323819
4062	LY6H	6.583333333	1.39	-2.243733365
159963	SLC5A12	1.403333333	0.286666667	-2.291411668
5443	POMC	1.25	0.25	-2.321928095
221002	RASGEF1A	5.063333333	1.003333333	-2.335286478
653689	GSTT2B	2.033333333	0.4	-2.345774837
54765	TRIM44	1.22	0.24	-2.345774837
2515	ADAM2	1.226666667	0.24	-2.353636955
131149	OTOL1	1.746666667	0.34	-2.36099766
343637	RSPO4	5.263333333	1.023333333	-2.36270061
83482	SCRT1	3.336666667	0.646666667	-2.367313417
126014	OSCAR	5.753333333	1.11	-2.373838382
2330	FMO5	3.556666667	0.67	-2.40829277
8706	B3GALNT1	1.573333333	0.286666667	-2.456378295
9084	VCY	49.72	9.026666667	-2.461561058
27063	ANKRD1	1.43	0.25	-2.516015147
53826	FXYD6	1.126666667	0.196666667	-2.518236387
10266	RAMP2	6.446666667	1.096666667	-2.555428306
8749	ADAM18	15.89666667	2.666666667	-2.575614878
335	APOA1	3.866666667	0.646666667	-2.579996248
106865373	WRB-SH3BGR	2.486666667	0.413333333	-2.58883551
9641	IKBKE	1.063333333	0.176666667	-2.589492159
9315	NREP	44.07333333	6.853333333	-2.68502825
56979	PRDM9	7.713333333	1.133333333	-2.766782213
50863	NTM	8.656666667	1.213333333	-2.834835658
2888	GRB14	3.333333333	0.446666667	-2.899695094
3270	HRC	6.396666667	0.843333333	-2.923145422
79827	CLMP	1.186666667	0.15	-2.983880335

7425	VGF	51.29666667	5.99	-3.098237172
103344718	HOTS	2.86	0.326666667	-3.130123993
710	SERPING1	109.19	12.13333333	-3.16979288
84898	PLXDC2	2.613333333	0.286666667	-3.188445089
93099	DMKN	5.43	0.563333333	-3.268891452
203328	SUSD3	1.063333333	0.106666667	-3.317412614
81035	COLEC12	23.82333333	2.386666667	-3.319306296
56884	FSTL5	1.166666667	0.113333333	-3.363748271
22837	COBLL1	3.76	0.36	-3.38466385
11030	RBPMS	3.693333333	0.34	-3.441316824
9388	LIPG	3.103333333	0.283333333	-3.453246421
286499	FAM133A	2.99	0.26	-3.523561956
29951	PDZRN4	2.73	0.226666667	-3.5902568
257019	FRMD3	1.71	0.13	-3.717412797
100506243	KRBOX1	1.48	0.103333333	-3.840219556
57685	CACHD1	1.603333333	0.106666667	-3.909893084
429	ASCL1	1.313333333	0.08	-4.037089319
7837	PXDN	1.063333333	0.06	-4.147487612
118427	OLFM3	2.07	0.116666667	-4.149166441
1535	CYBA	20.83333333	1.14	-4.19178796
9074	CLDN6	13.81	0.713333333	-4.274993119
64220	STRA6	1.556666667	0.056666667	-4.779815898
100271846	ERVV-2	1.023333333	0.036666667	-4.802663227
1482	NKX2-5	1.063333333	0.033333333	-4.995484519
653220	XAGE1A	1.446666667	0.043333333	-5.061111514
653067	XAGE1B	1.446666667	0.043333333	-5.061111514
7078	TIMP3	5.326666667	0.13	-5.356649474
56776	FMN2	1.373333333	0.033333333	-5.364572432
2045	EPHA7	1.16	0.023333333	-5.635588574
80712	ESX1	1.003333333	0.01	-6.648657176
101060389	TBC1D3D	1.05	0.01	-6.714245518
7001	PRDX2	8.78	0.083333333	-6.71918344
